# Supplementary material for: Epigenetic regulation by polycomb repressive complex 1 promotes cerebral cavernous malformations
Source: EMBO Mol Med. 2024 Oct 14;16(11):10. doi: 10.1038/s44321-024-00152-9 (PMC11555420; doi:10.1038/s44321-024-00152-9)
Supplement: Supplementary file 1 — Appendix [file 44321_2024_152_MOESM1_ESM.pdf]

APPENDIX

		page
1	Table of contents	1
2	APPENDIX FIGURES	2
2.1	Appendix Figure S1	3
2.2	Appendix Figure S1 legend	4
2.3	Appendix Figure S2	5
2.4	Appendix Figure S2 legend	6

APPENDIX FIGURES

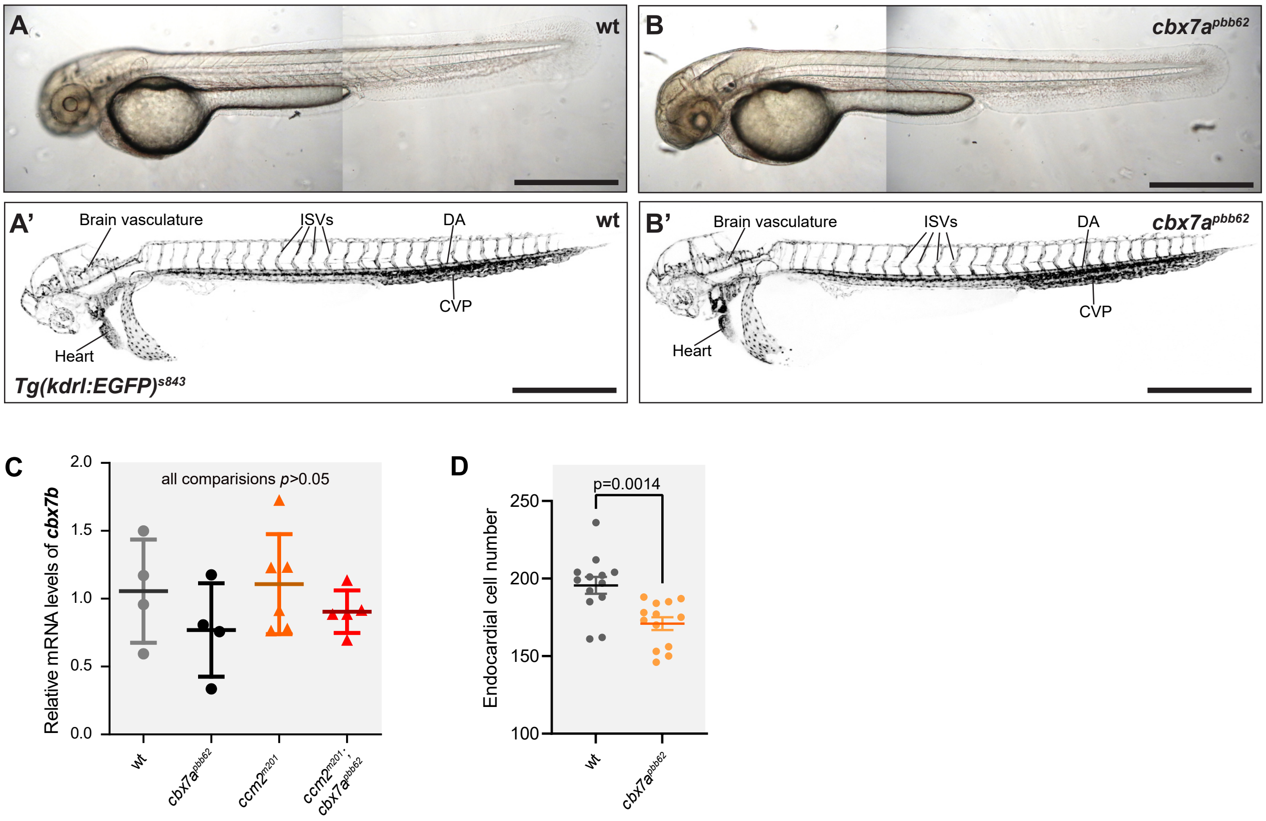

Pham, Rödel et *al*. Appendix Figure S1

Appendix Figure S1. The cardiovascular system of *cbx7a^pbb62^* mutants is phenotypically normal and embryos develop normally. (**A, B**) Shown are bright field images of wild-type (A) and *cbx7a*^{pbb62} mutant (B) embryos at 56 hpf. (**A'**, **B'**). Shown are maximum projections of confocal z-scan projections. The embryonic cardiovascular system is marked by *Tg(kdrl:EGFP)*^{s843} expression. There are no gross morphological phenotypes in mutants. (**C**) Quantifications of *cbx7b* mRNA expression levels based on qRT-PCR reveal that expression is unaltered in *cbx7a*^{pbb62}, *ccm2*^{m201}, or *ccm2*^{m201};*cbx7a*^{pbb62} double mutants (whole embryos compared with wild-type siblings) (One-way ANOVA statistic test). (**D**) Quantifications of endocardial cell numbers in *cbx7a*^{pbb62} mutants at 56hpf, which are slightly reduced compared with wild-type (Two tailed t-test). ISVs, intersegmental vessels; DA, dorsal aorta; CVP, caudal vein plexus. Scale bars are 500 µm.

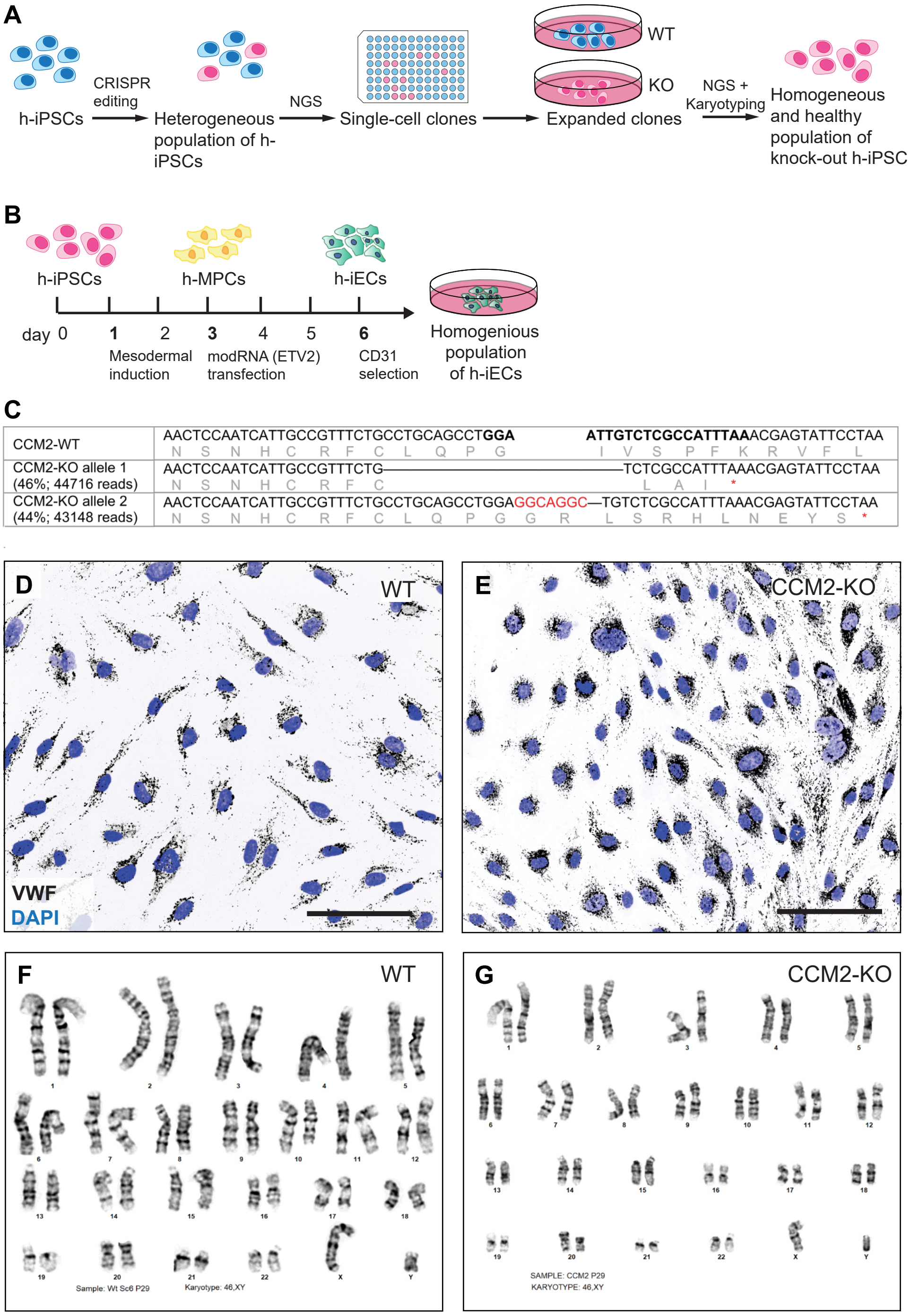

Pham, Rödel et *al*. Appendix Figure S2

Appendix Figure S2. Generation and characterization of *CCM2* knock out (KO) human induced pluripotent stem cell-derived endothelial cells (iPSC-derived ECs). (**A**) Experimental scheme of generating *CCM2*-KO iPSC-ECs (see details in Materials and Methods section) using CRISPR/Cas9 genome editing and later next generation sequencing (NGS) to screen KO clones. (**B**) Experimental scheme of differentiating h-iPSCs into iECs. (**C**) Alignment of *CCM2*-KO alleles with wild-type (WT) allele, in which target sequence of single-guide RNA is in bold in WT, deleted nucleotides in mutated alleles are represented in strike lines, replaced or inserted nucleotides are represented in red. Indels result in amino acid changes and premature stop codons, which are represented in red asterisks. Mutations were sequenced by NGS with frequencies and number of reads indicated. (**D, E**) Representative fluorescence microscopy images of WT and CCM2-KO iECs immunostained for von Willebrand factor as endothelial cell marker. (**F,G**) Karyotyping results of WT and *CCM2*-KO iECs indicate that there are no chromosomal anomalies associated with the knockout procedure and that healthy clones were generated and utilized.
