# Supplementary figures and images for: Epigenetic regulation by polycomb repressive complex 1 promotes cerebral cavernous malformations (part 1 of 2)
Source: EMBO Mol Med. 2024 Oct 14;16(11):10. doi: 10.1038/s44321-024-00152-9 (PMC11555420; doi:10.1038/s44321-024-00152-9)

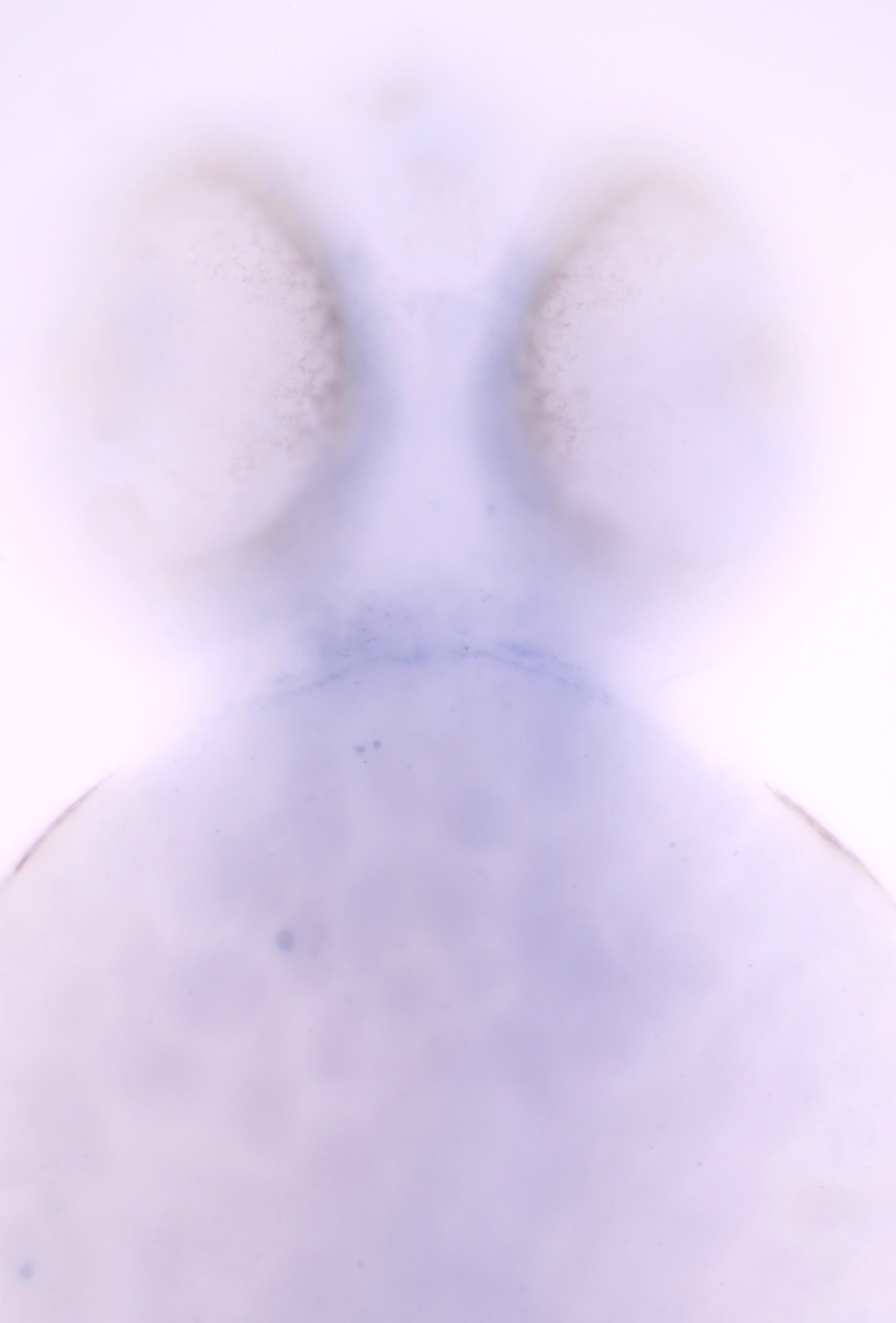

Supplement: Supplementary file 6 — Source data Fig. 1 [file 44321_2024_152_MOESM6_ESM.zip › Figure 1/1C/cbx7a-wish-wt-56hpf-1-1.tif]

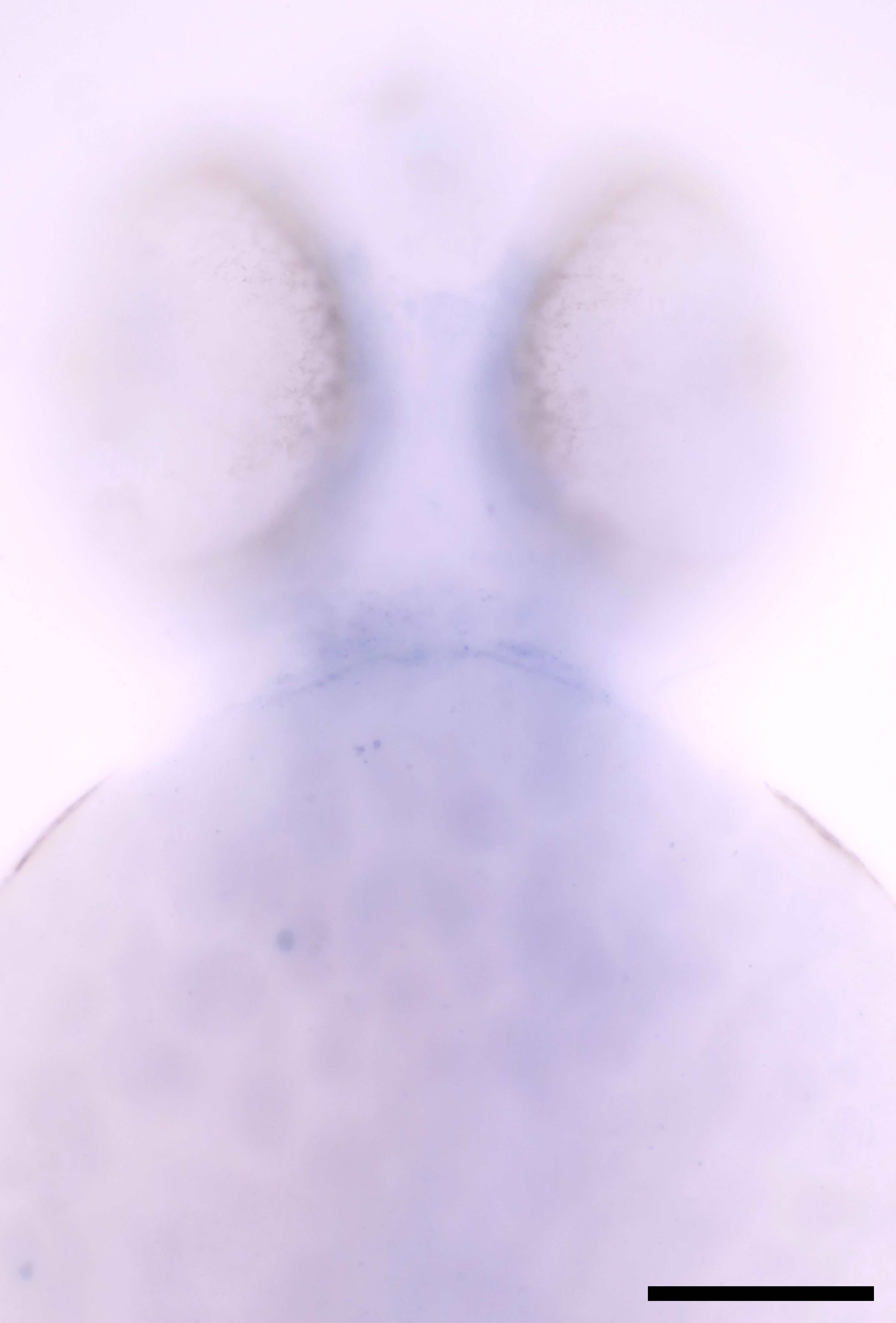

Supplement: Supplementary file 6 — Source data Fig. 1 [file 44321_2024_152_MOESM6_ESM.zip › Figure 1/1C/cbx7a-wish-wt-56hpf-1-scalebar.png]

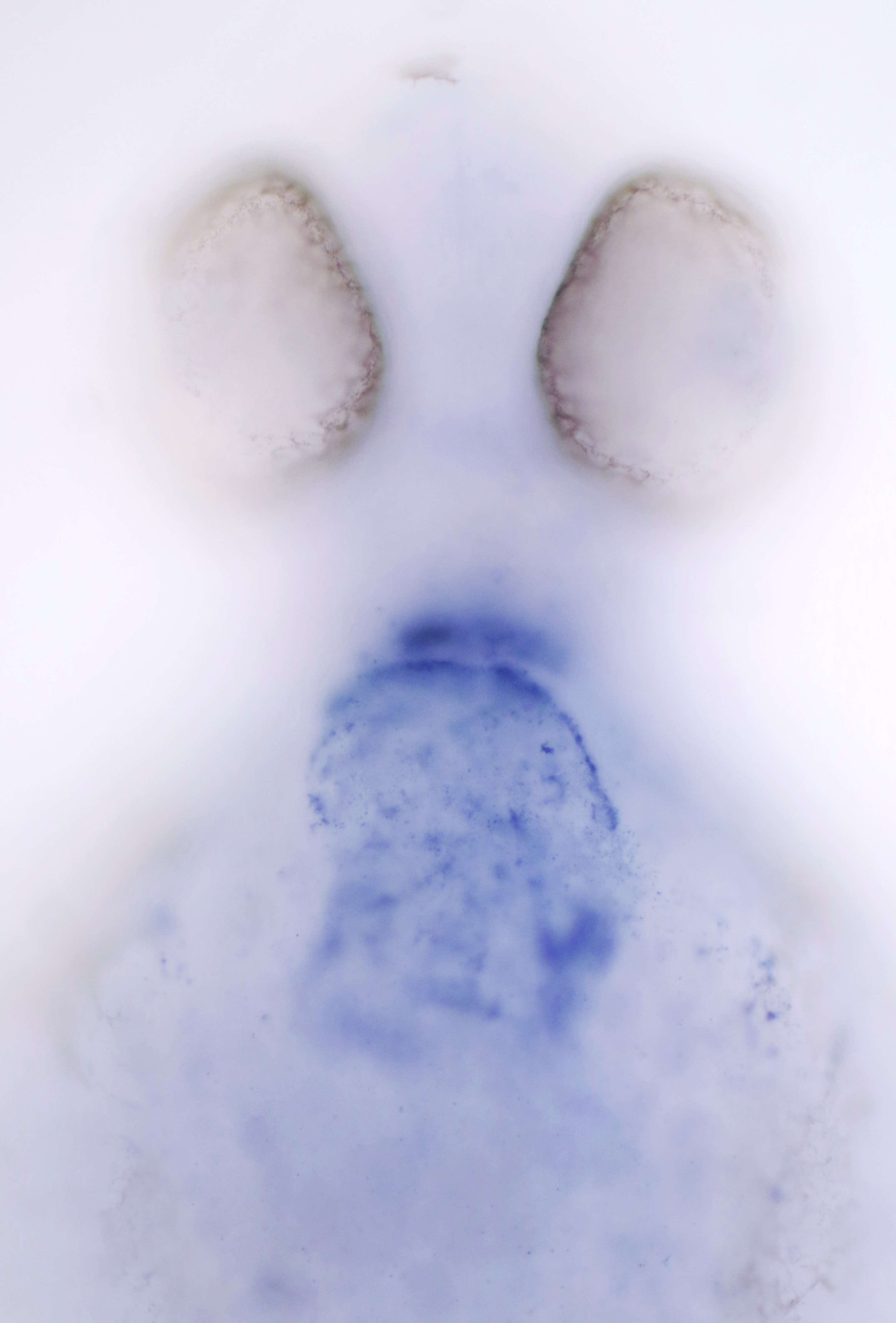

Supplement: Supplementary file 6 — Source data Fig. 1 [file 44321_2024_152_MOESM6_ESM.zip › Figure 1/1D/cbx7a-wish-ccm2-56hpf-1-1.tif]

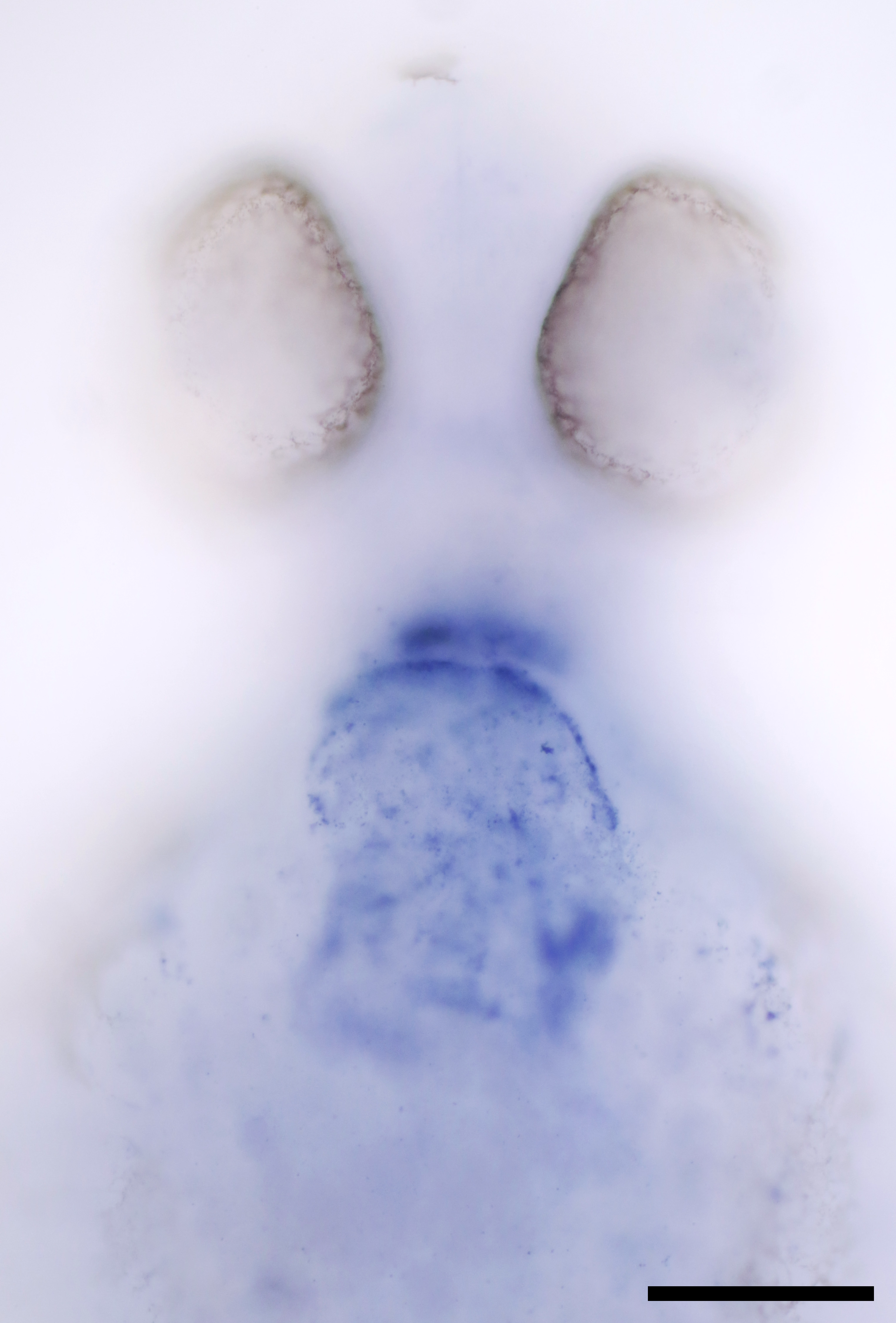

Supplement: Supplementary file 6 — Source data Fig. 1 [file 44321_2024_152_MOESM6_ESM.zip › Figure 1/1D/cbx7a-wish-ccm2-56hpf-1-scalebar.png]

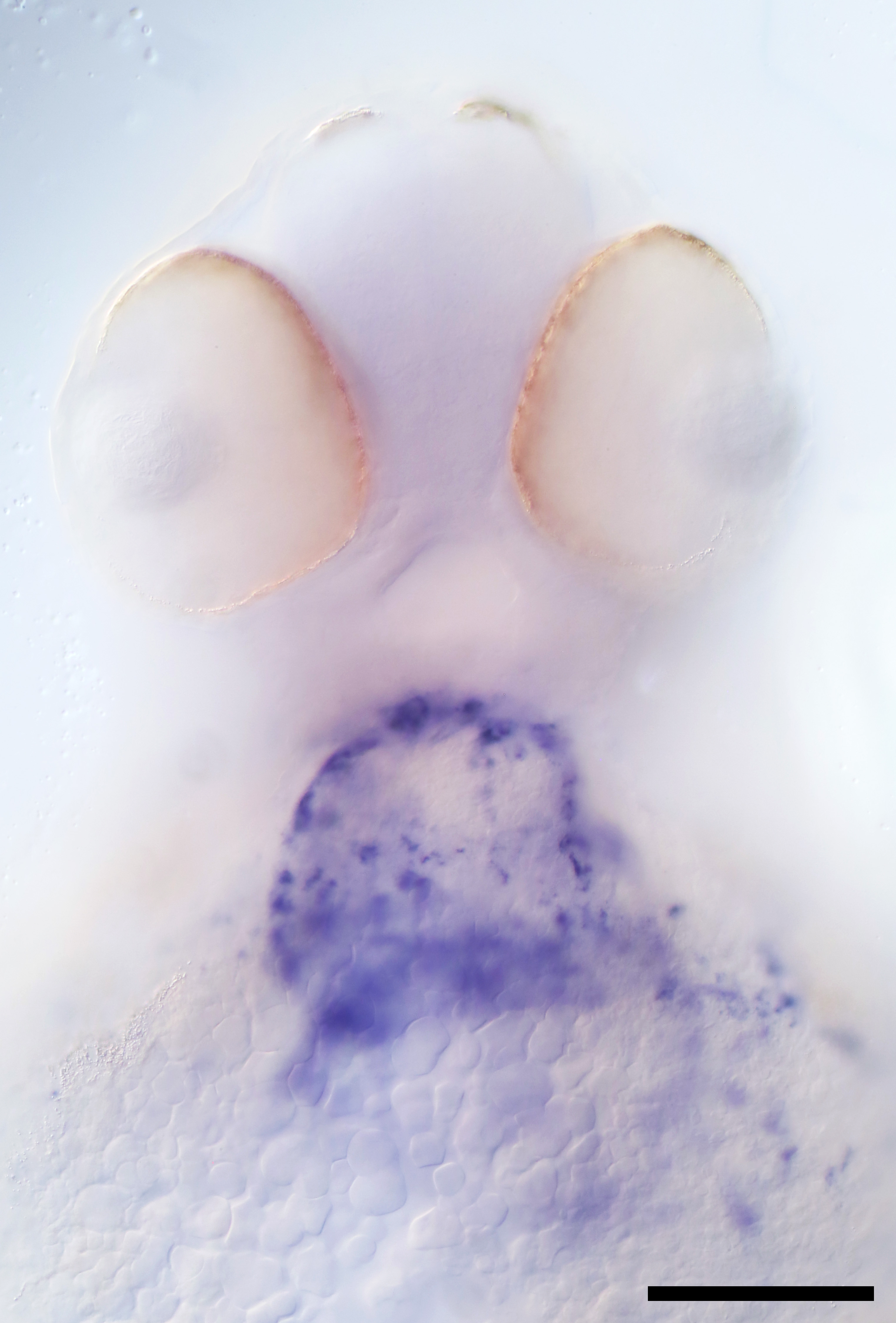

Supplement: Supplementary file 6 — Source data Fig. 1 [file 44321_2024_152_MOESM6_ESM.zip › Figure 1/1E/cbx7a-WISH-krit1-56hpf-1-scalebar.png]

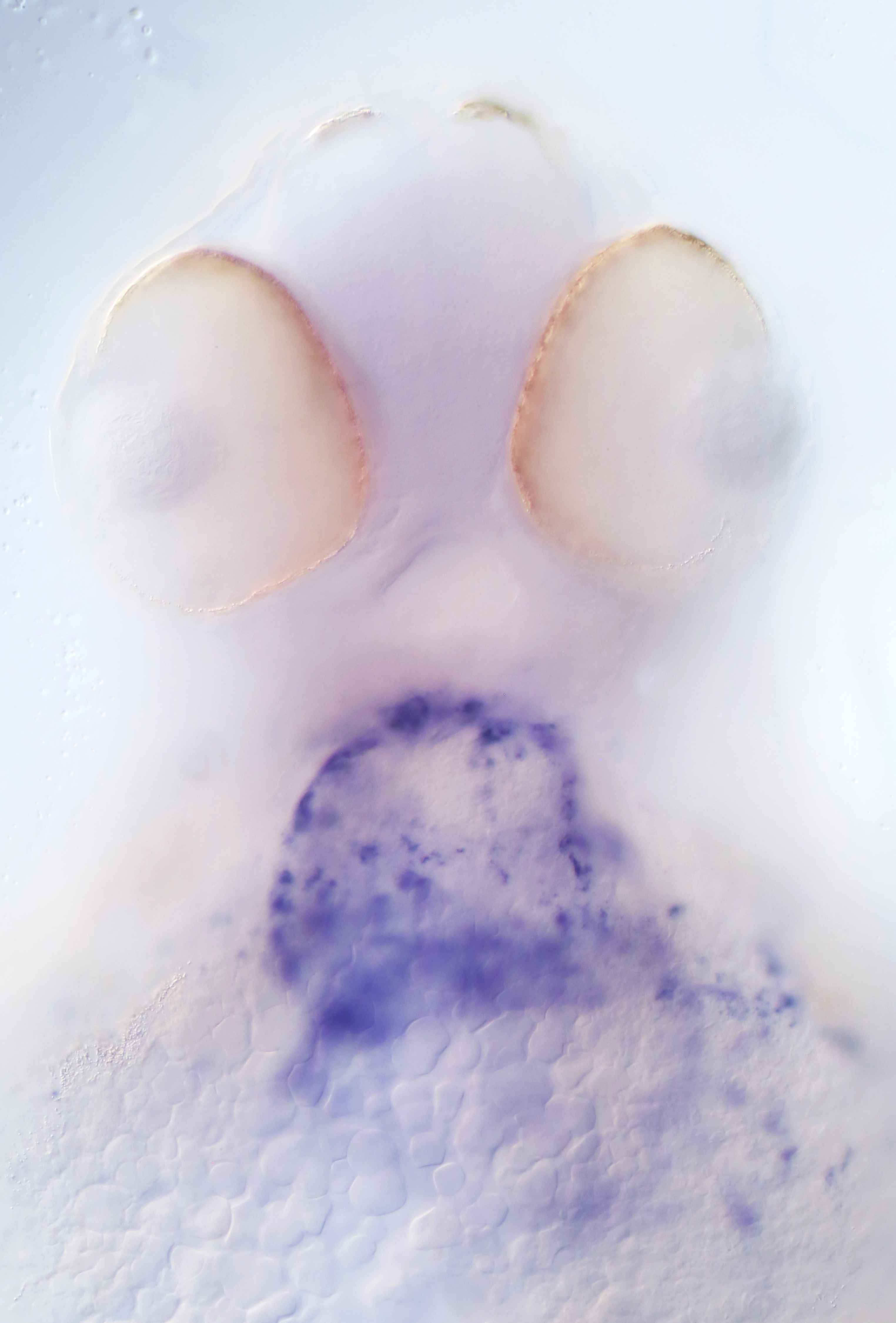

Supplement: Supplementary file 6 — Source data Fig. 1 [file 44321_2024_152_MOESM6_ESM.zip › Figure 1/1E/cbx7a-WISH-krit1-56hpf-1.tif]

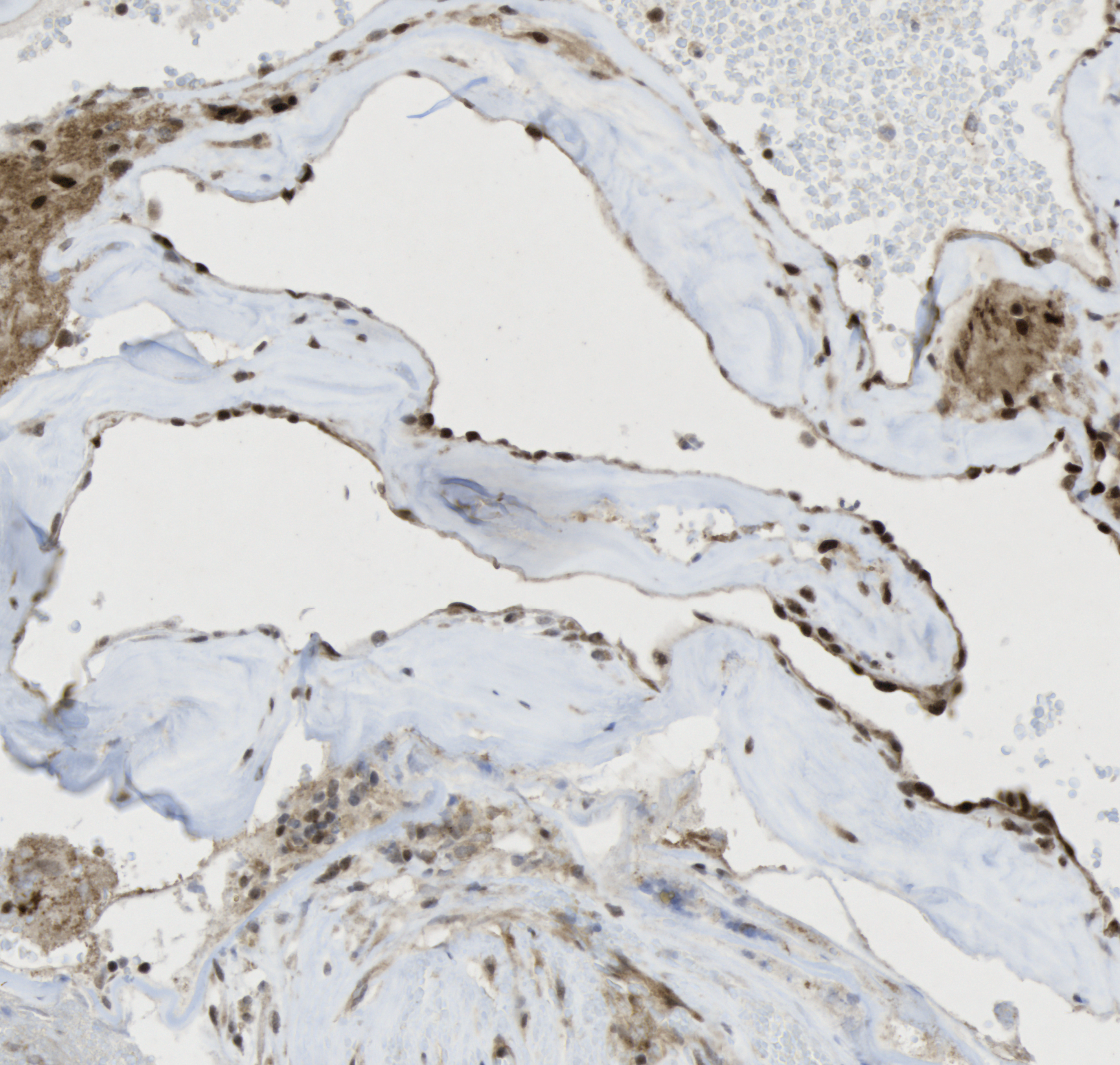

Supplement: Supplementary file 6 — Source data Fig. 1 [file 44321_2024_152_MOESM6_ESM.zip › Figure 1/1G/AA386_CBX7z35.tif]

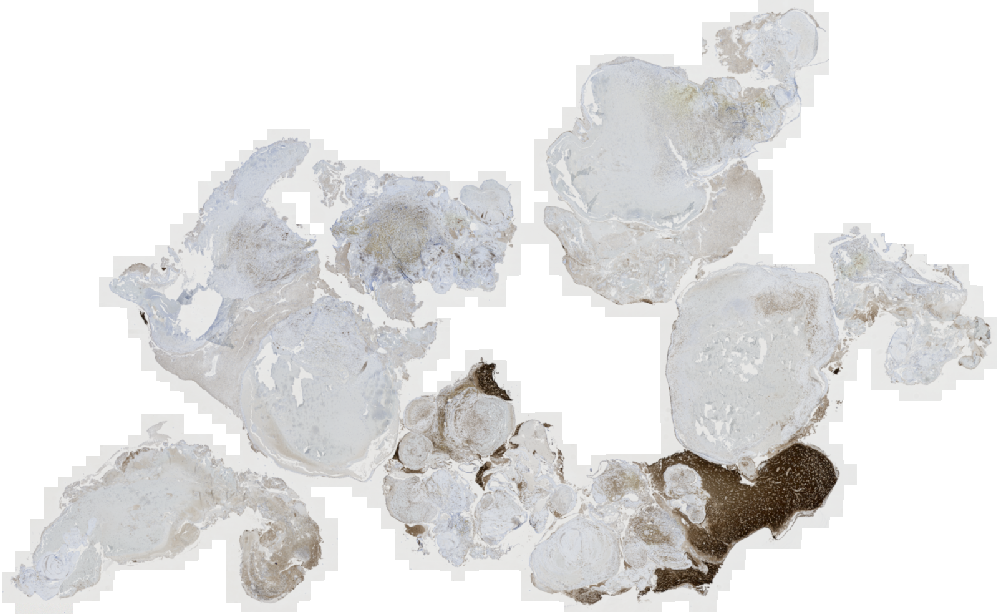

Supplement: Supplementary file 6 — Source data Fig. 1 [file 44321_2024_152_MOESM6_ESM.zip › Figure 1/1G/AA386_CBX7_overview2.tif]

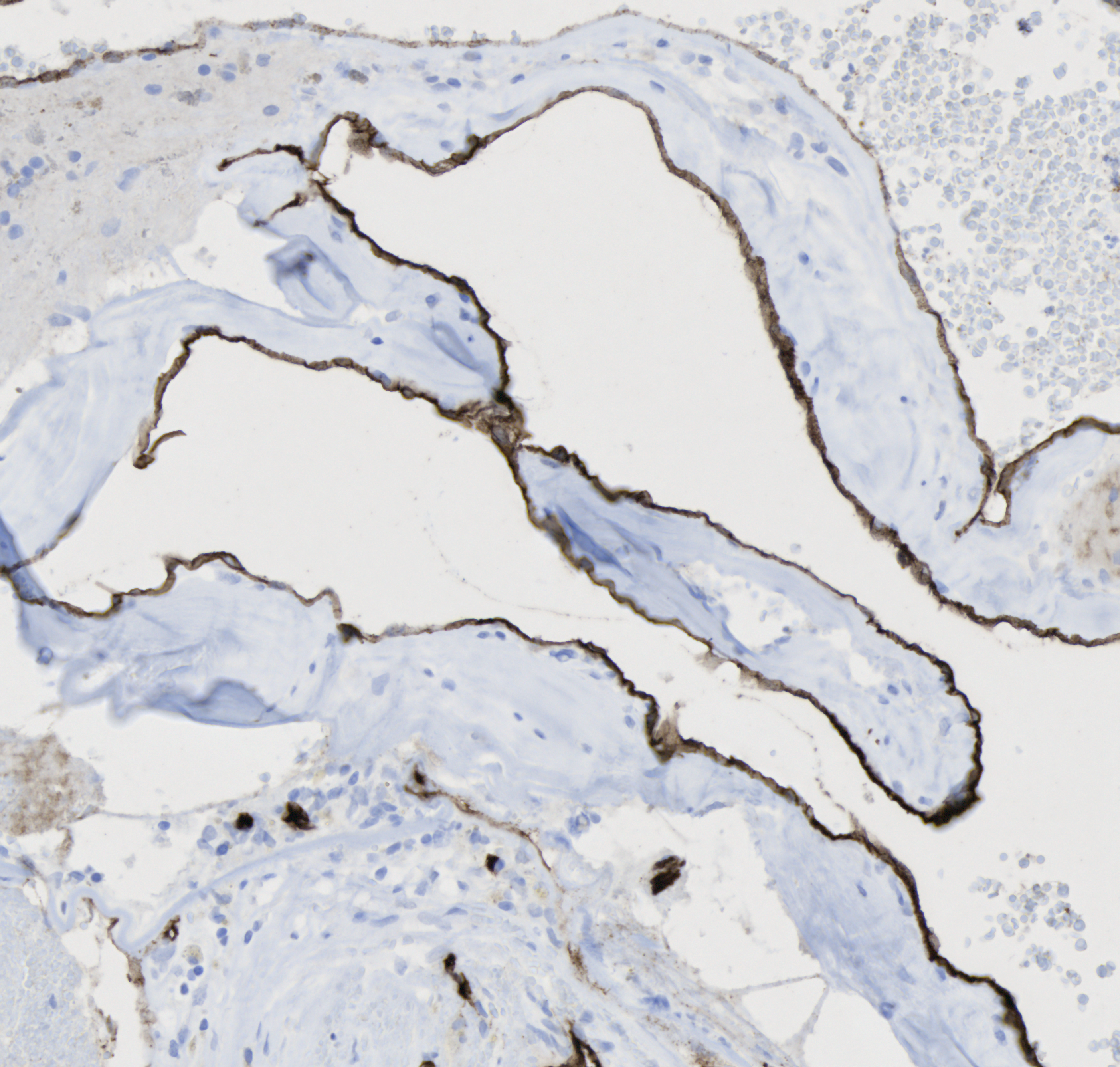

Supplement: Supplementary file 6 — Source data Fig. 1 [file 44321_2024_152_MOESM6_ESM.zip › Figure 1/1G/AA386_CD34z35.tif]

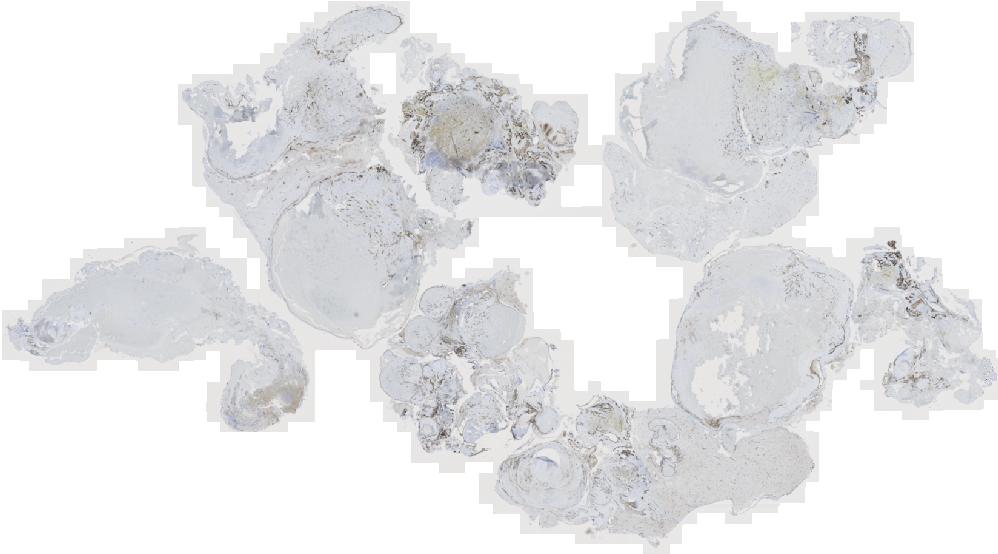

Supplement: Supplementary file 6 — Source data Fig. 1 [file 44321_2024_152_MOESM6_ESM.zip › Figure 1/1G/AA386_CD34_overview.tif]

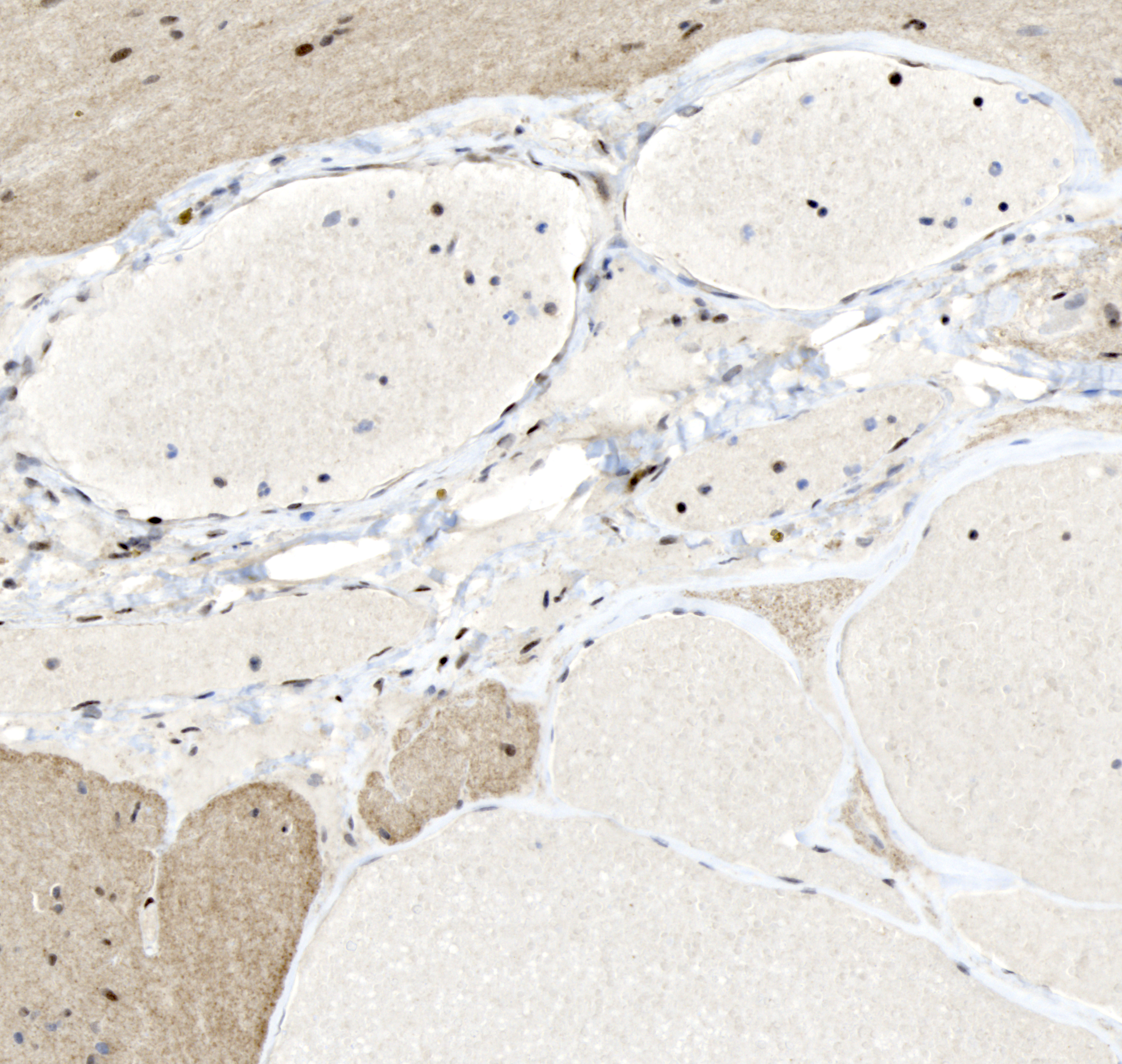

Supplement: Supplementary file 6 — Source data Fig. 1 [file 44321_2024_152_MOESM6_ESM.zip › Figure 1/1H/AA1134_CBX7z35.tif]

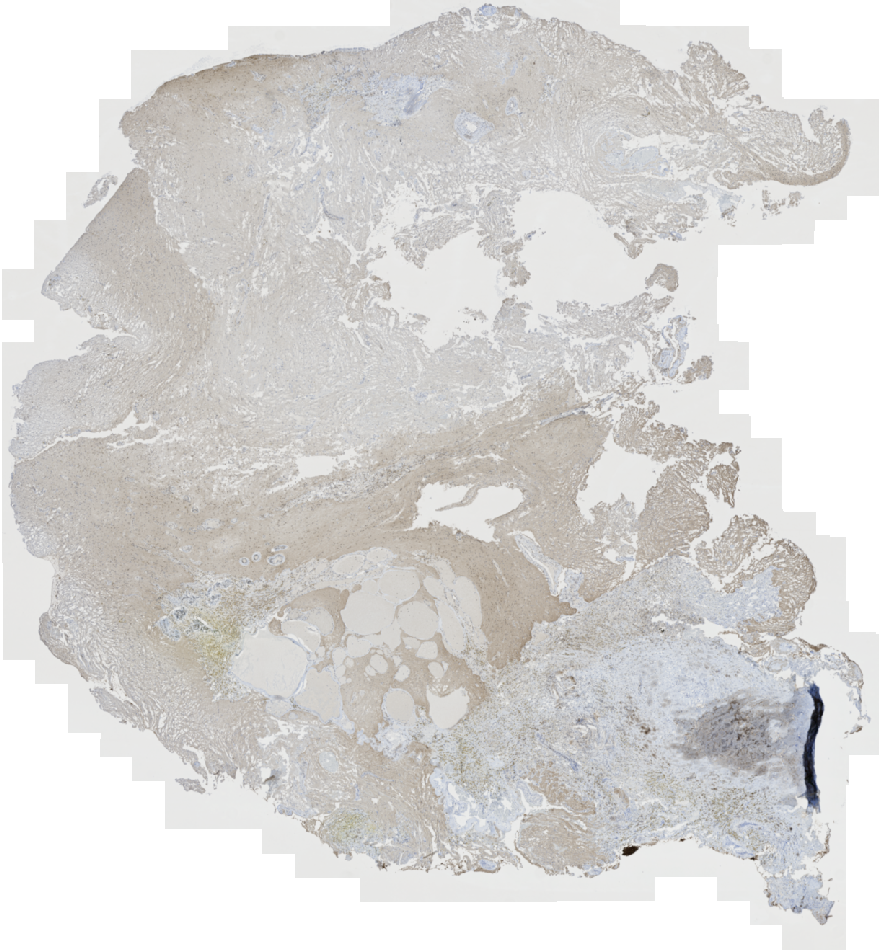

Supplement: Supplementary file 6 — Source data Fig. 1 [file 44321_2024_152_MOESM6_ESM.zip › Figure 1/1H/AA1134_CBX7_overview.tif]

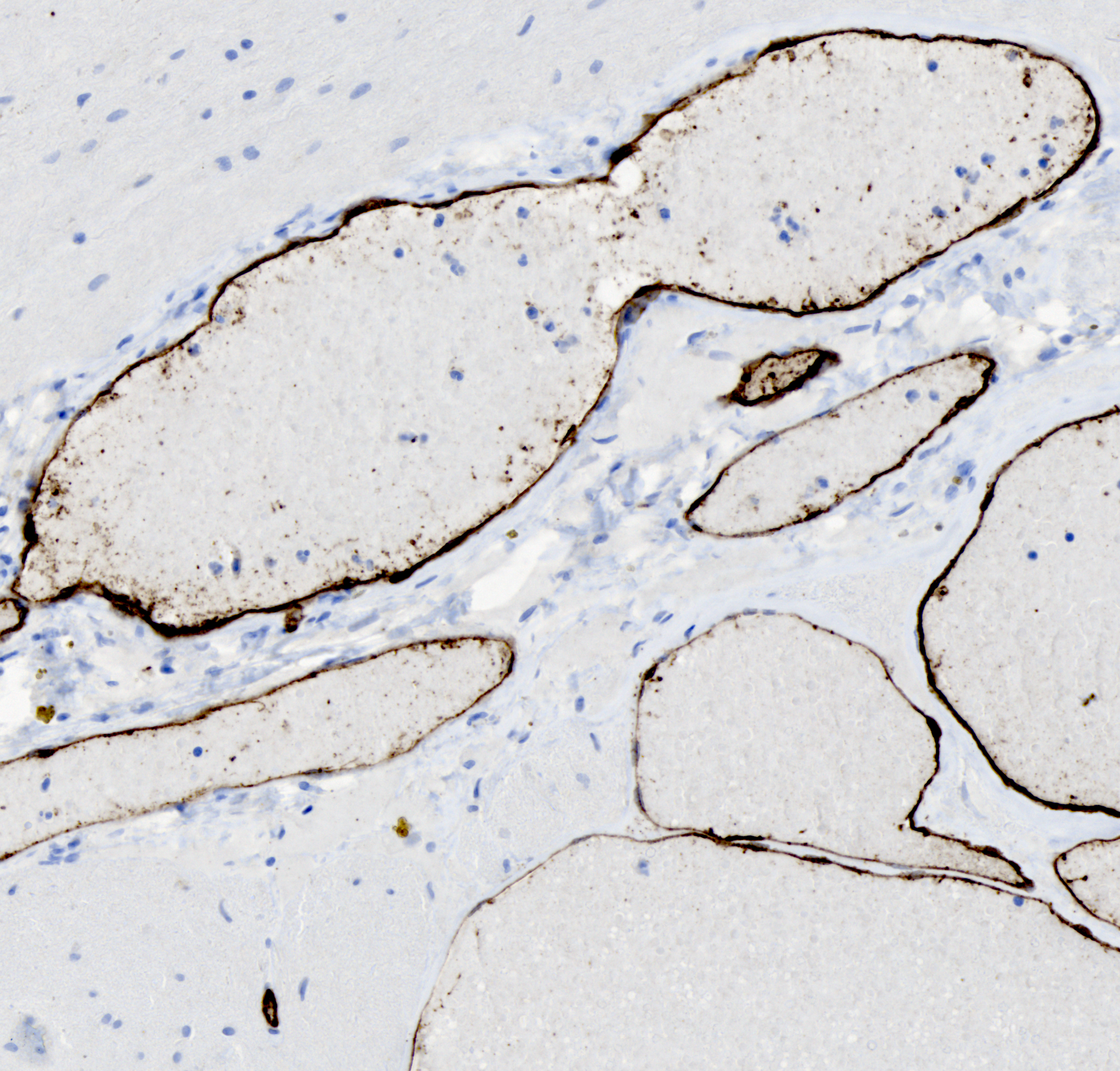

Supplement: Supplementary file 6 — Source data Fig. 1 [file 44321_2024_152_MOESM6_ESM.zip › Figure 1/1H/AA1134_CD34z35.tif]

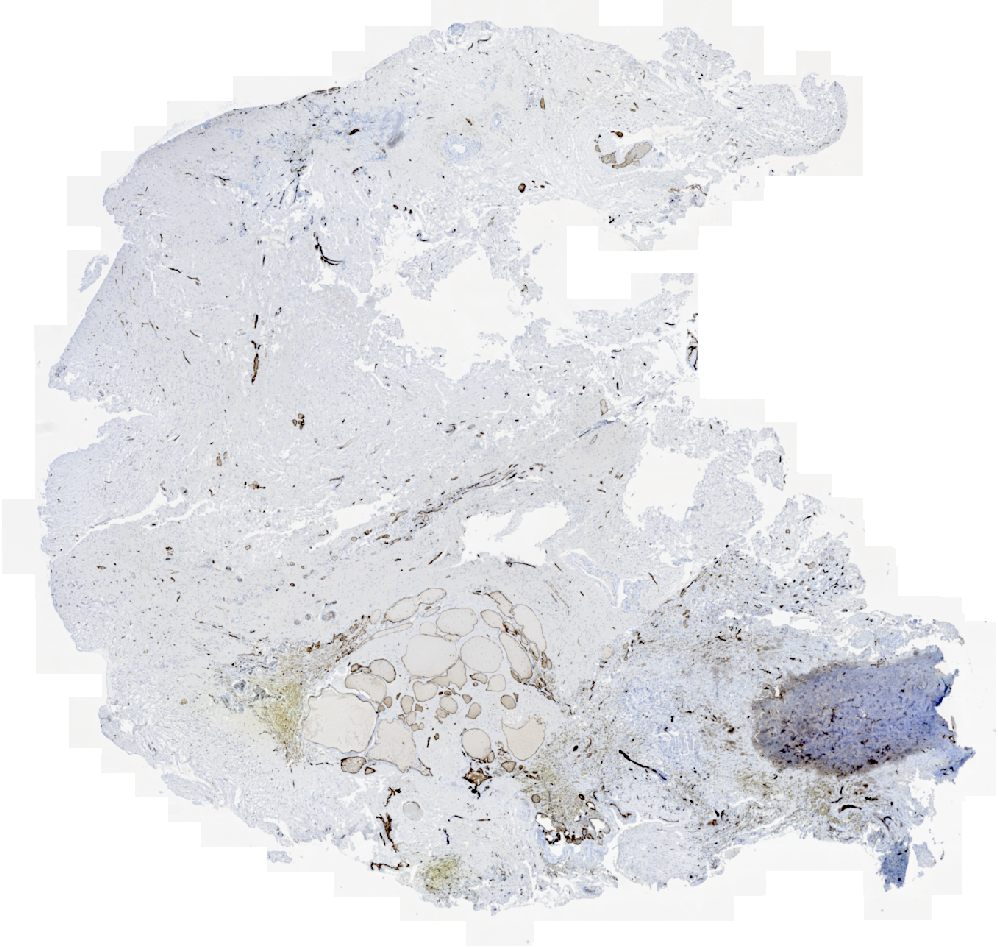

Supplement: Supplementary file 6 — Source data Fig. 1 [file 44321_2024_152_MOESM6_ESM.zip › Figure 1/1H/AA1134_CD34_overview.tif]

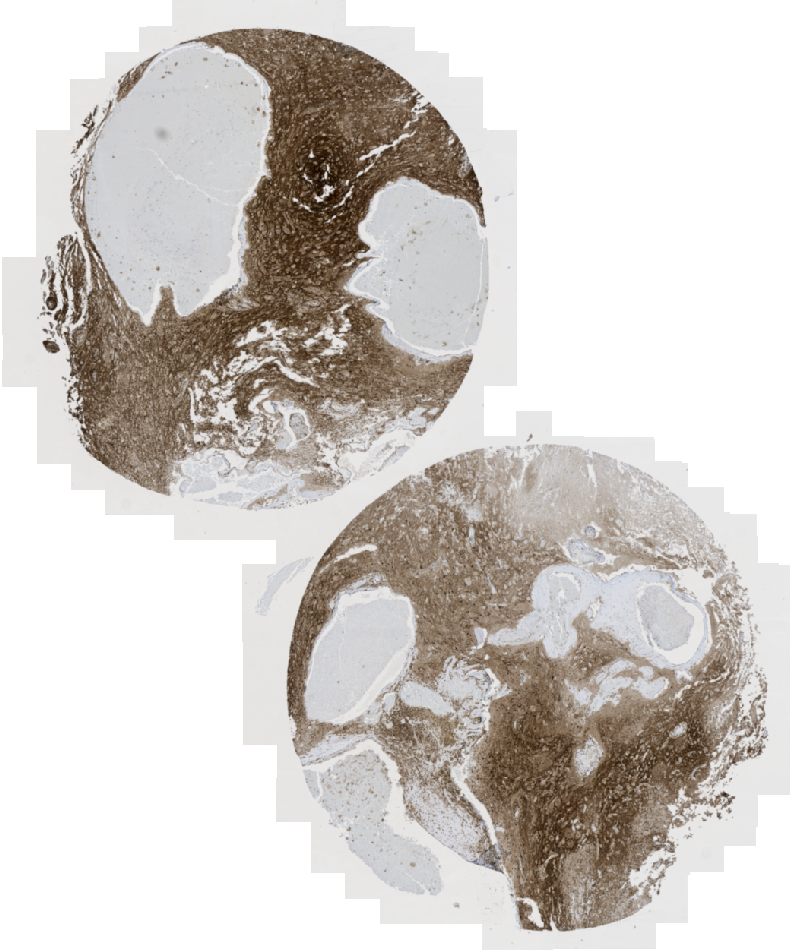

Supplement: Supplementary file 6 — Source data Fig. 1 [file 44321_2024_152_MOESM6_ESM.zip › Figure 1/1I/AA1150_CBX7overview.tif]

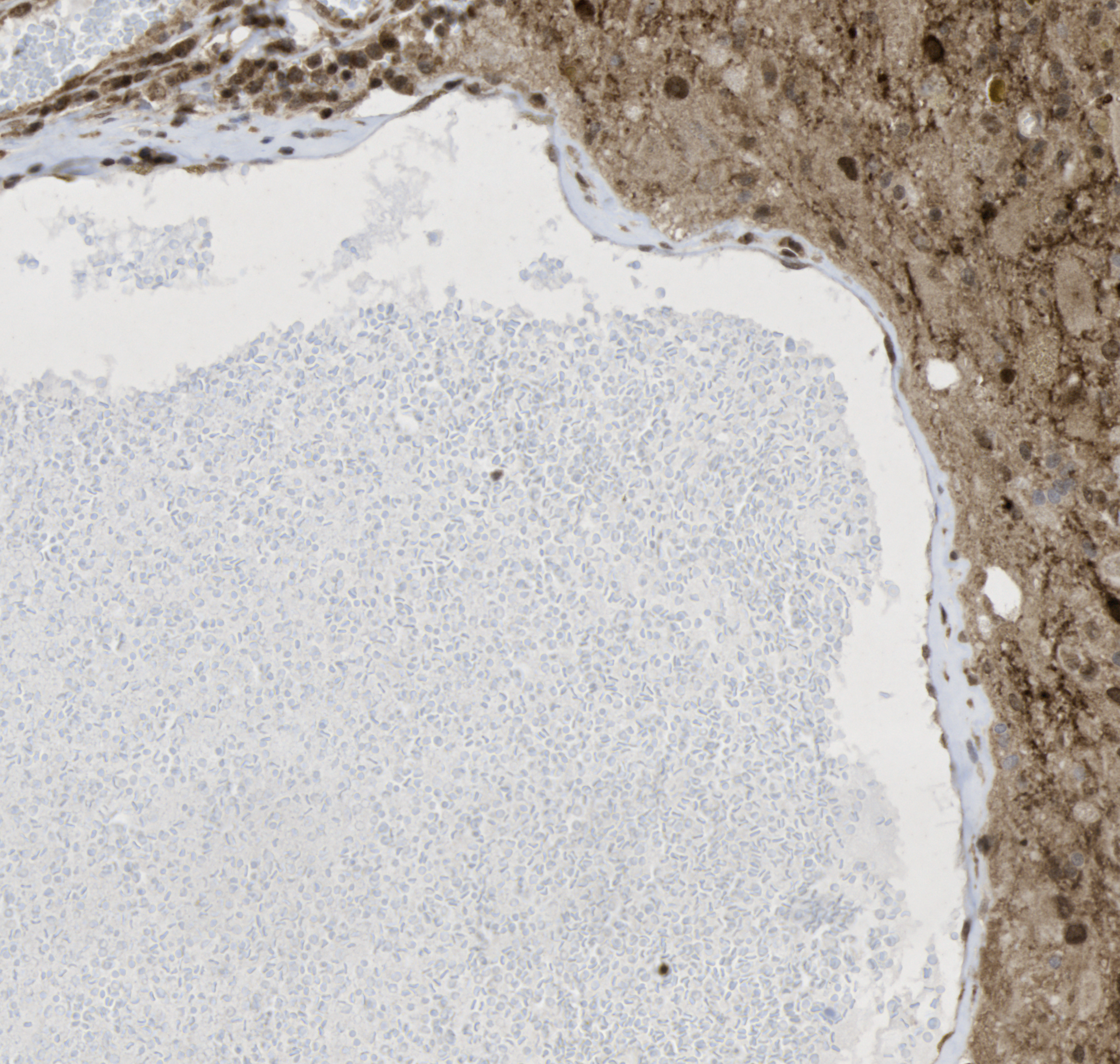

Supplement: Supplementary file 6 — Source data Fig. 1 [file 44321_2024_152_MOESM6_ESM.zip › Figure 1/1I/AA1150_CBX7z35.tif]

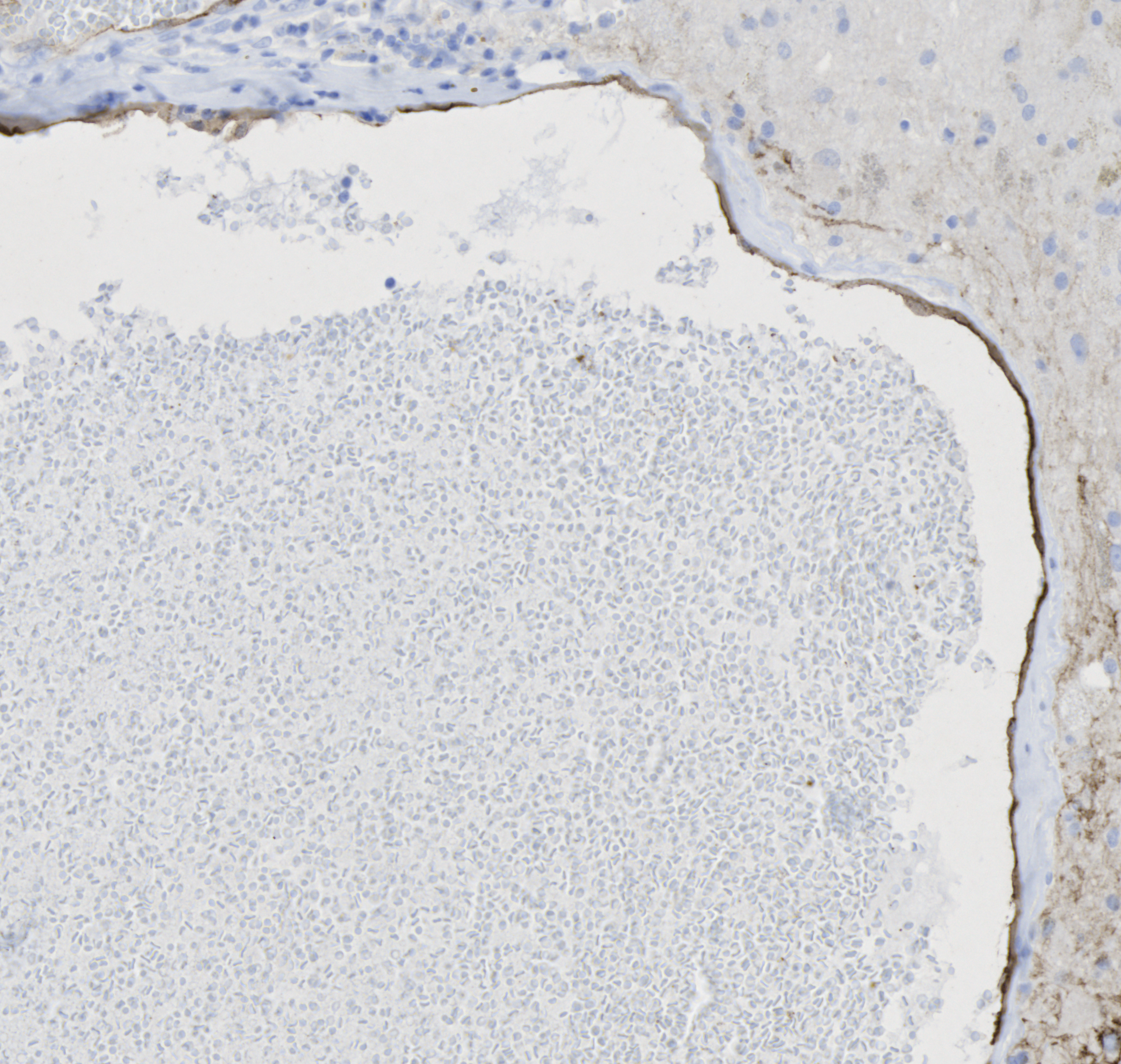

Supplement: Supplementary file 6 — Source data Fig. 1 [file 44321_2024_152_MOESM6_ESM.zip › Figure 1/1I/AA1150_CD34z35.tif]

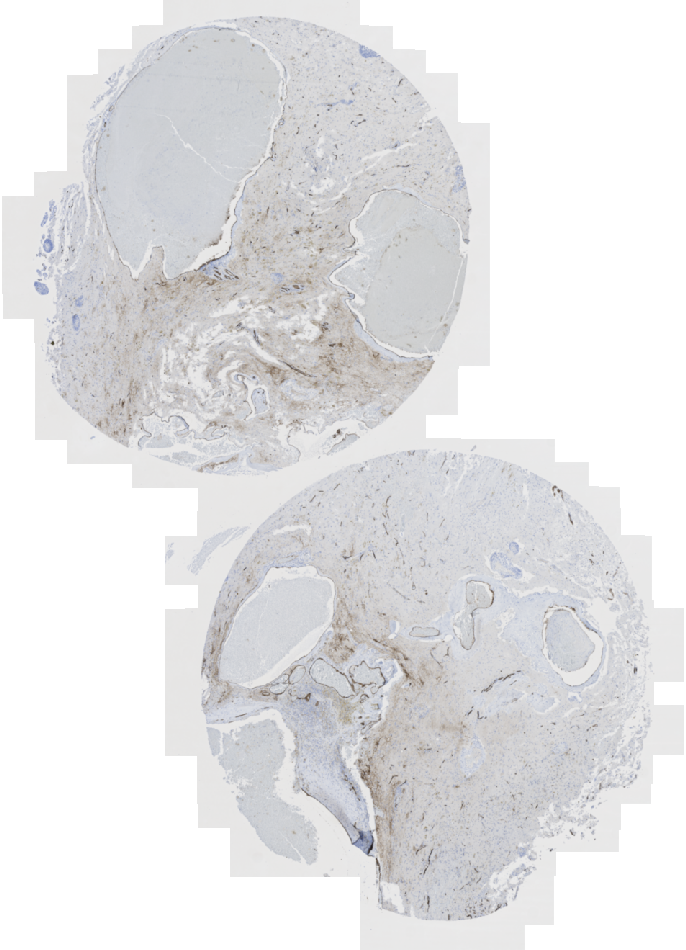

Supplement: Supplementary file 6 — Source data Fig. 1 [file 44321_2024_152_MOESM6_ESM.zip › Figure 1/1I/AA1150_CD34_overview.tif]

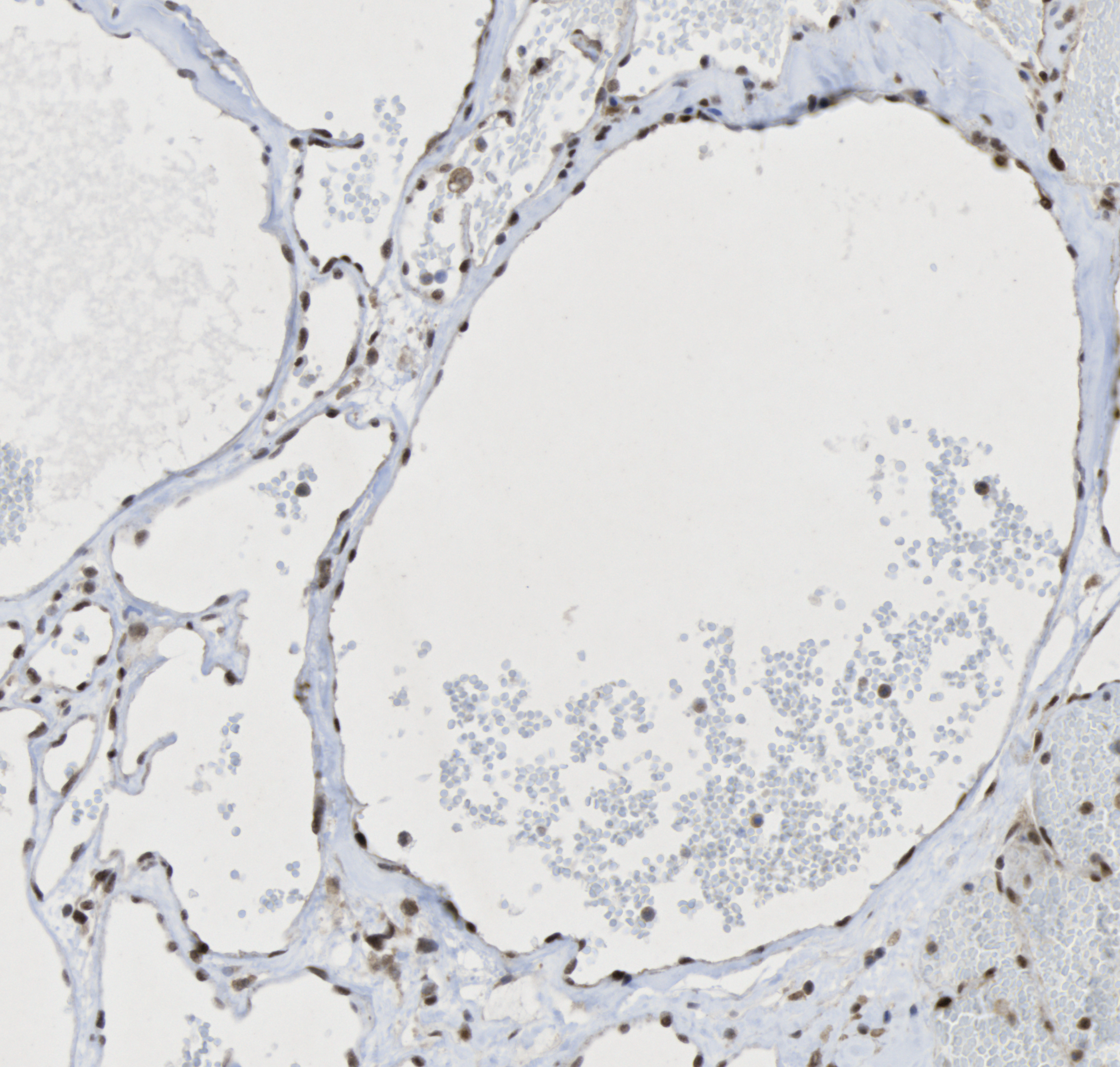

Supplement: Supplementary file 6 — Source data Fig. 1 [file 44321_2024_152_MOESM6_ESM.zip › Figure 1/1J/AA1152_CBX7z35.tif]

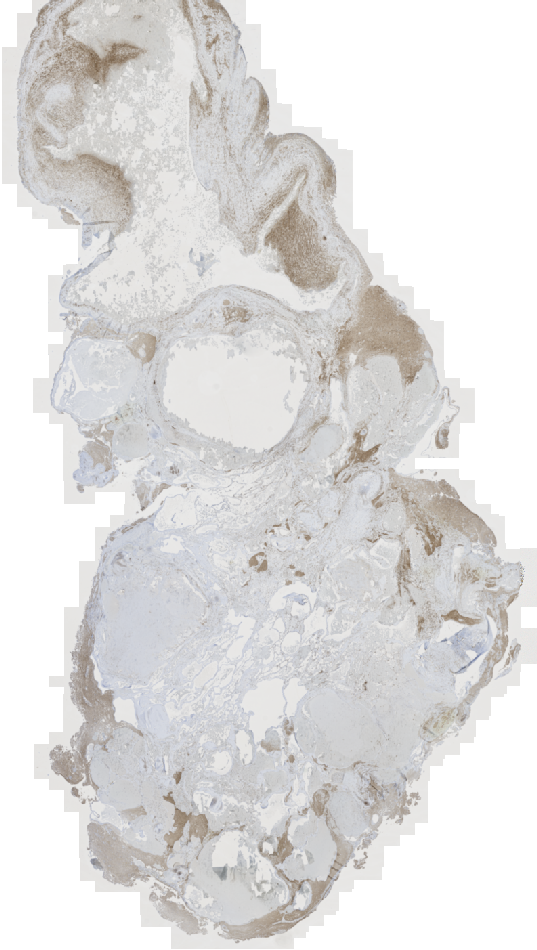

Supplement: Supplementary file 6 — Source data Fig. 1 [file 44321_2024_152_MOESM6_ESM.zip › Figure 1/1J/AA1152_CBX7_overview.tif]

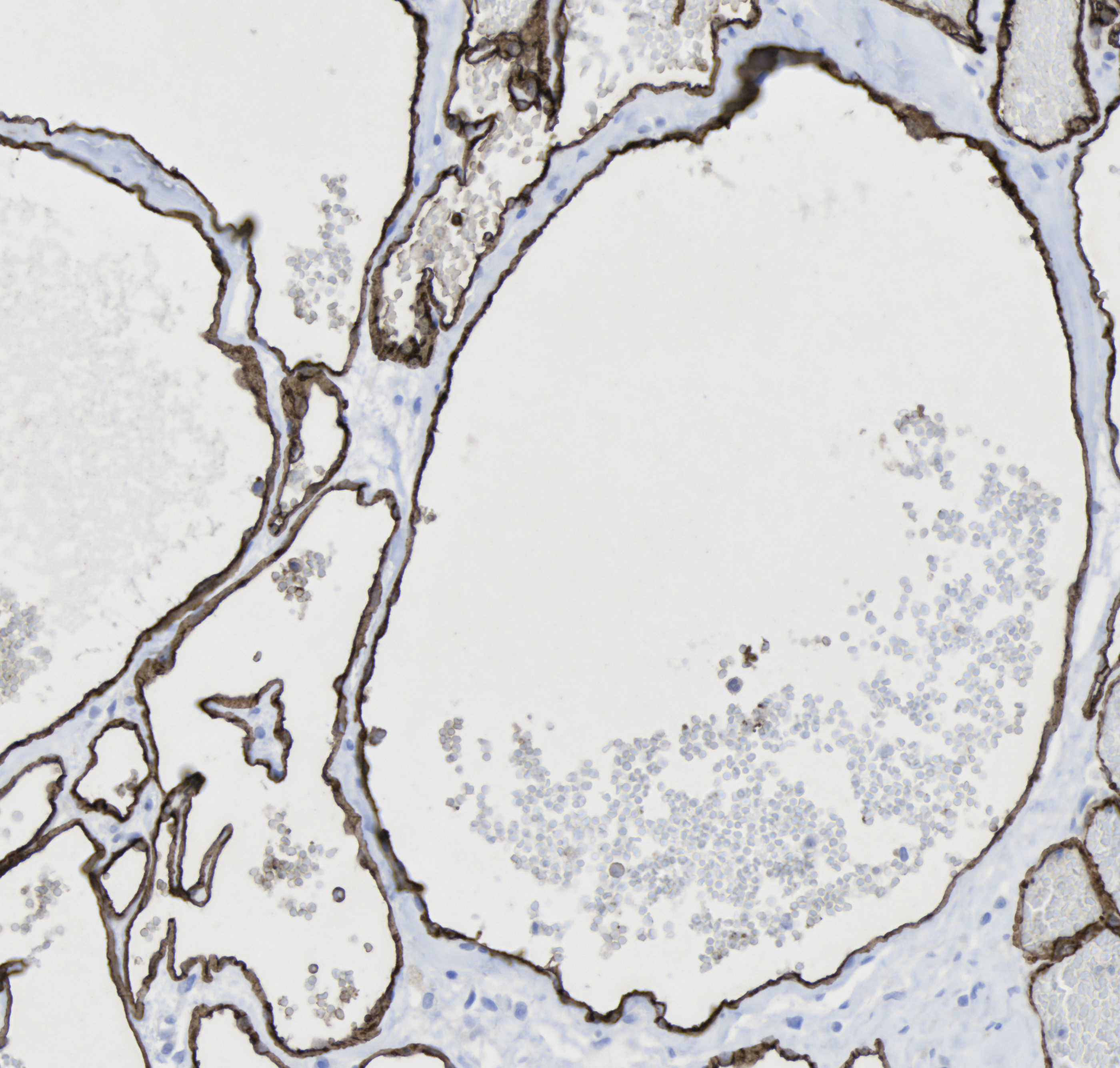

Supplement: Supplementary file 6 — Source data Fig. 1 [file 44321_2024_152_MOESM6_ESM.zip › Figure 1/1J/AA1152_CD34z35.tif]

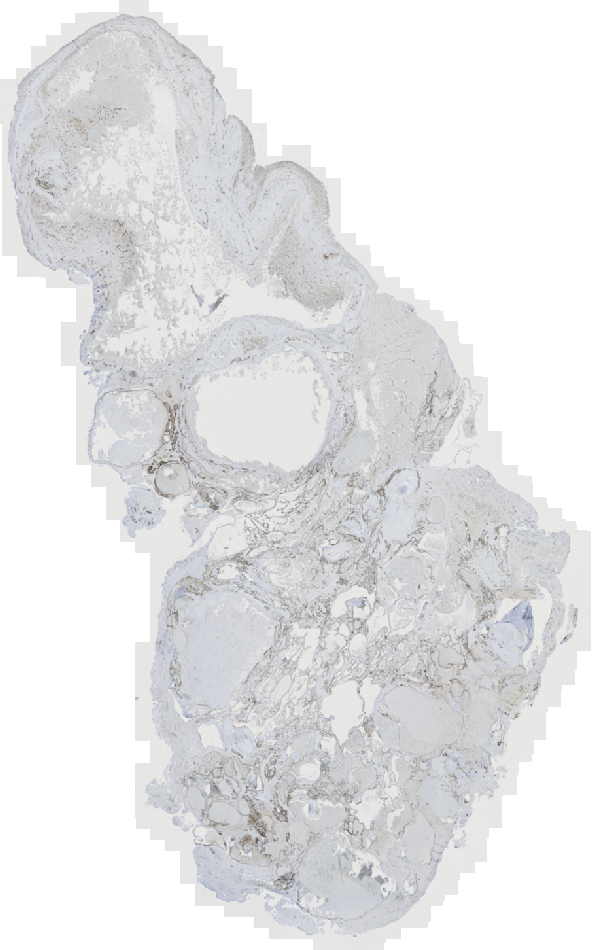

Supplement: Supplementary file 6 — Source data Fig. 1 [file 44321_2024_152_MOESM6_ESM.zip › Figure 1/1J/AA1152_CD34_overview.tif]

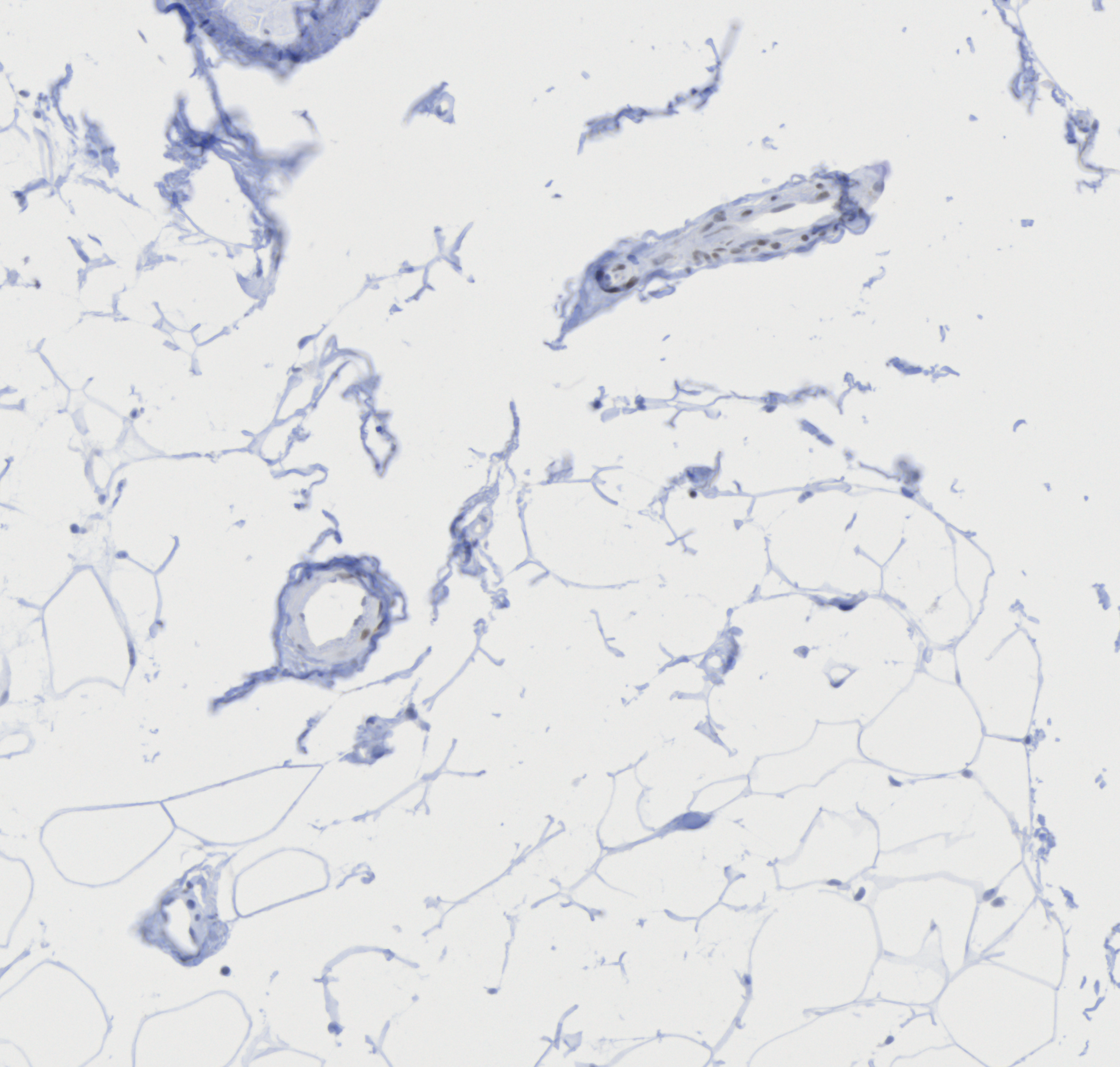

Supplement: Supplementary file 6 — Source data Fig. 1 [file 44321_2024_152_MOESM6_ESM.zip › Figure 1/1K/healtybrainz35.tif]

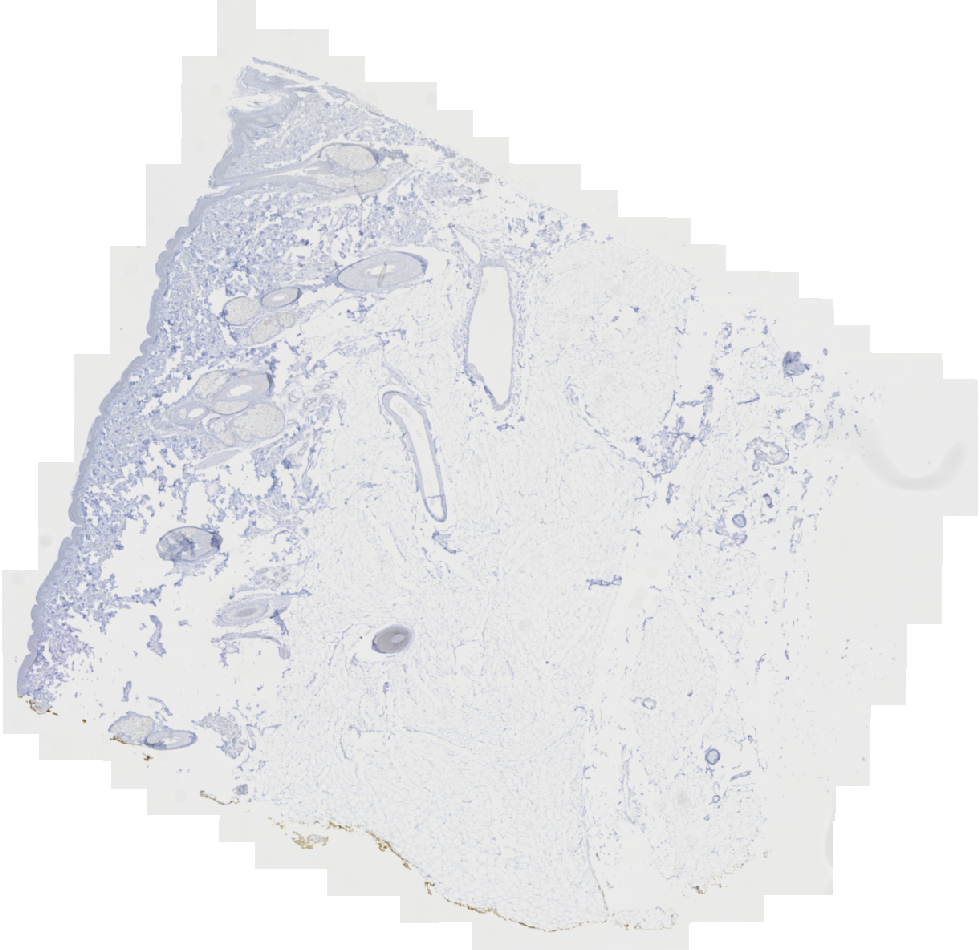

Supplement: Supplementary file 6 — Source data Fig. 1 [file 44321_2024_152_MOESM6_ESM.zip › Figure 1/1K/healtybrain_overview.tif]

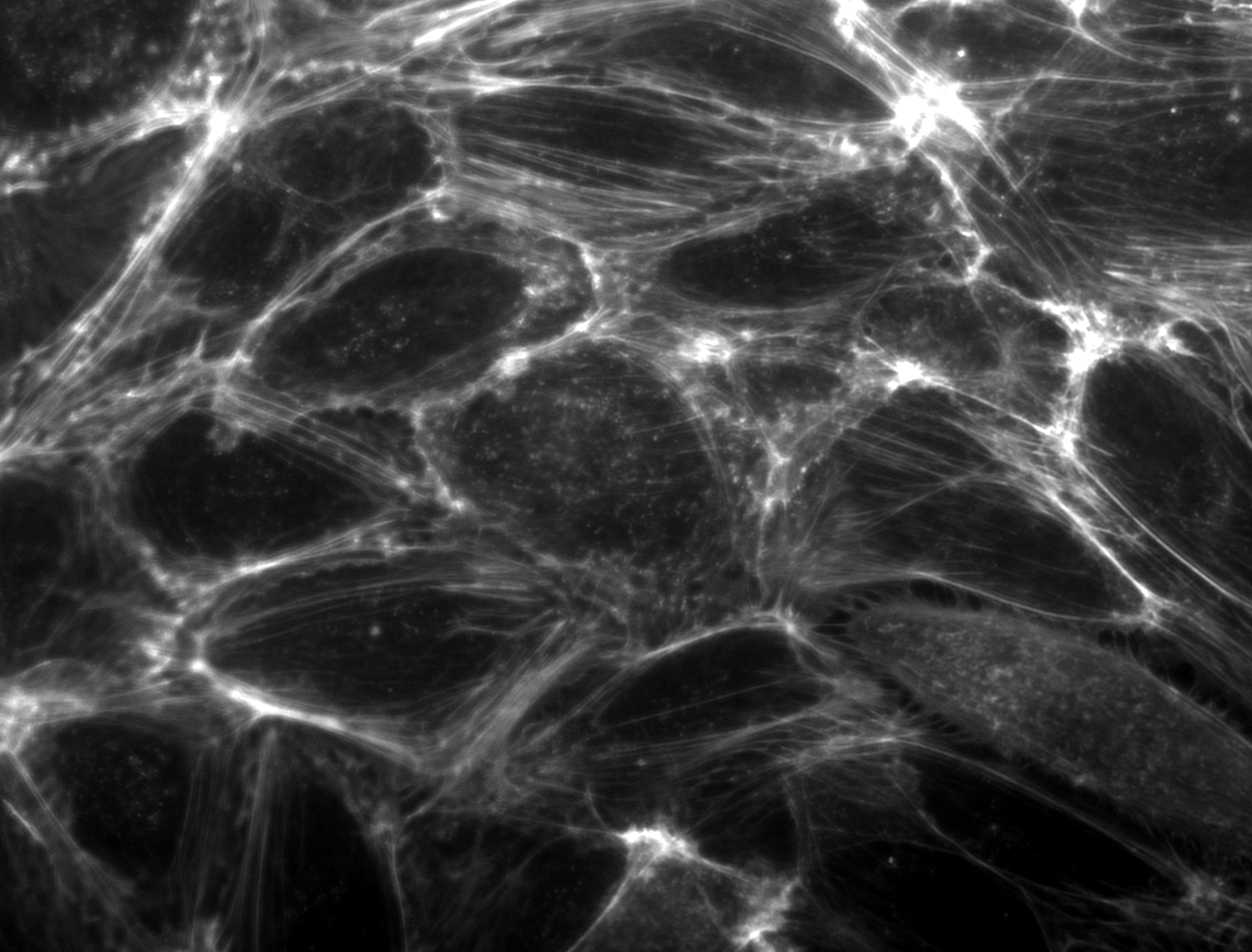

Supplement: Supplementary file 7 — Source data Fig. 2 [file 44321_2024_152_MOESM7_ESM.zip › Figure 2/2H/CT actin.tif]

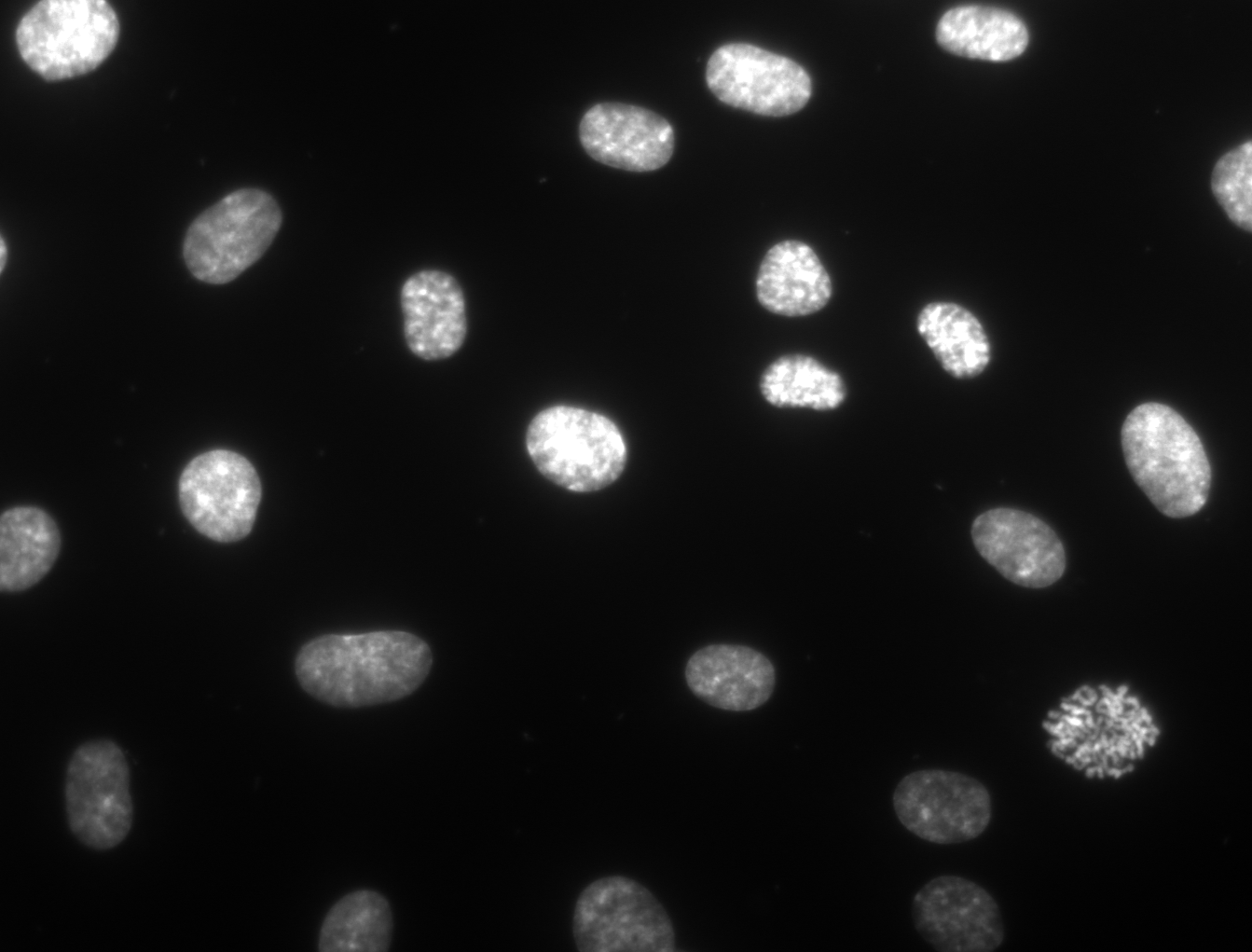

Supplement: Supplementary file 7 — Source data Fig. 2 [file 44321_2024_152_MOESM7_ESM.zip › Figure 2/2H/CT DAPI.tif]

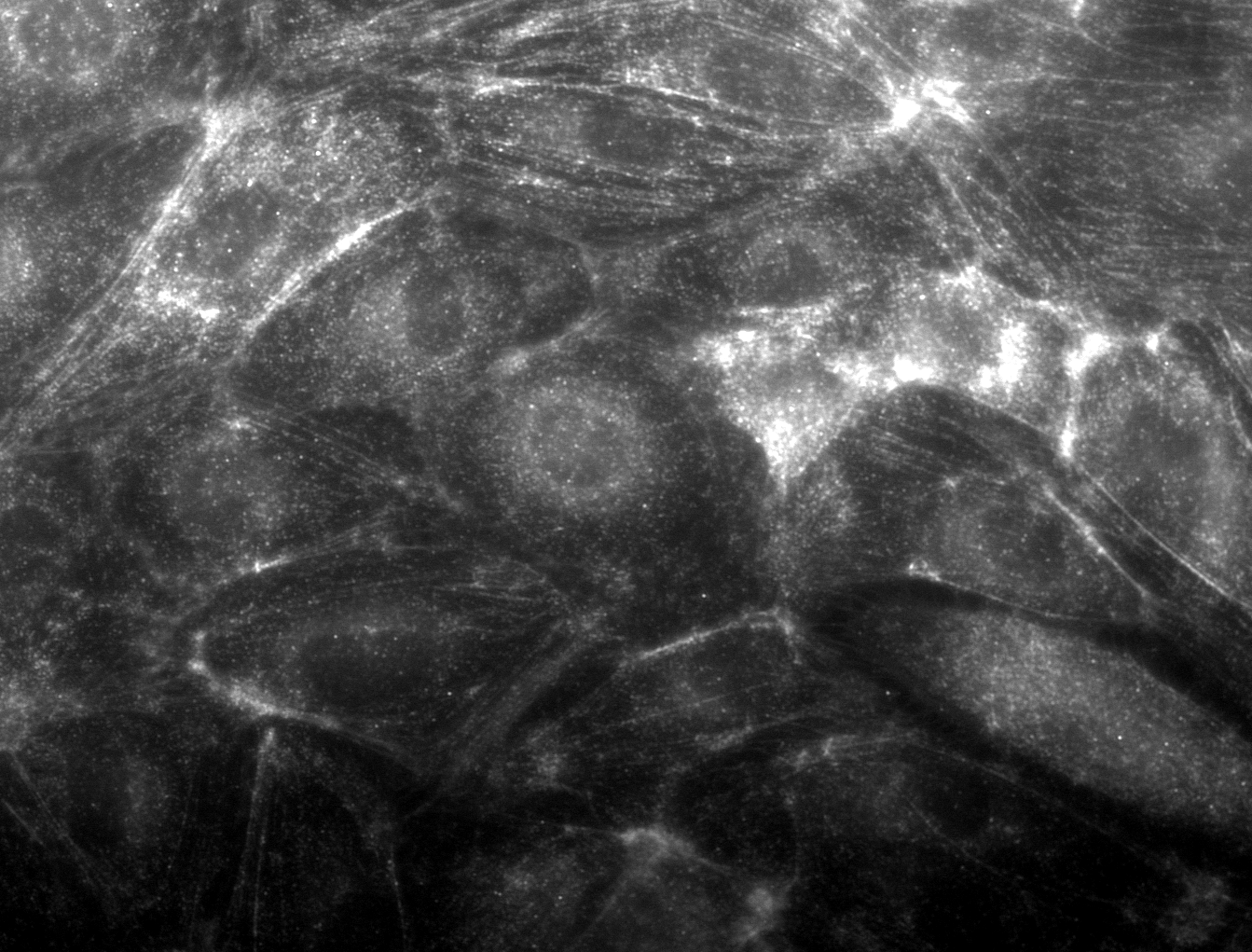

Supplement: Supplementary file 7 — Source data Fig. 2 [file 44321_2024_152_MOESM7_ESM.zip › Figure 2/2H/CT pMLC.tif]

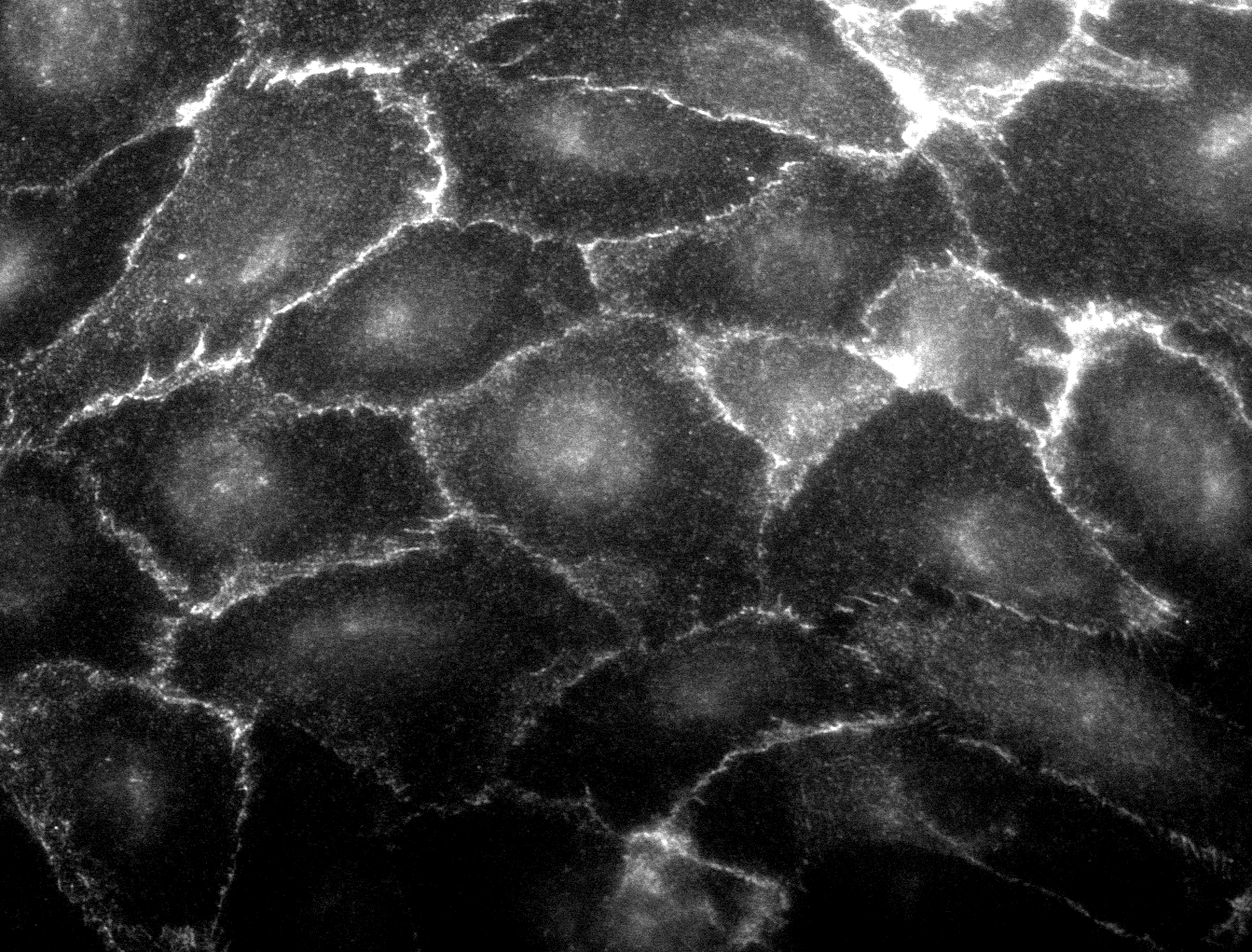

Supplement: Supplementary file 7 — Source data Fig. 2 [file 44321_2024_152_MOESM7_ESM.zip › Figure 2/2H/CT VE-cadherin.tif]

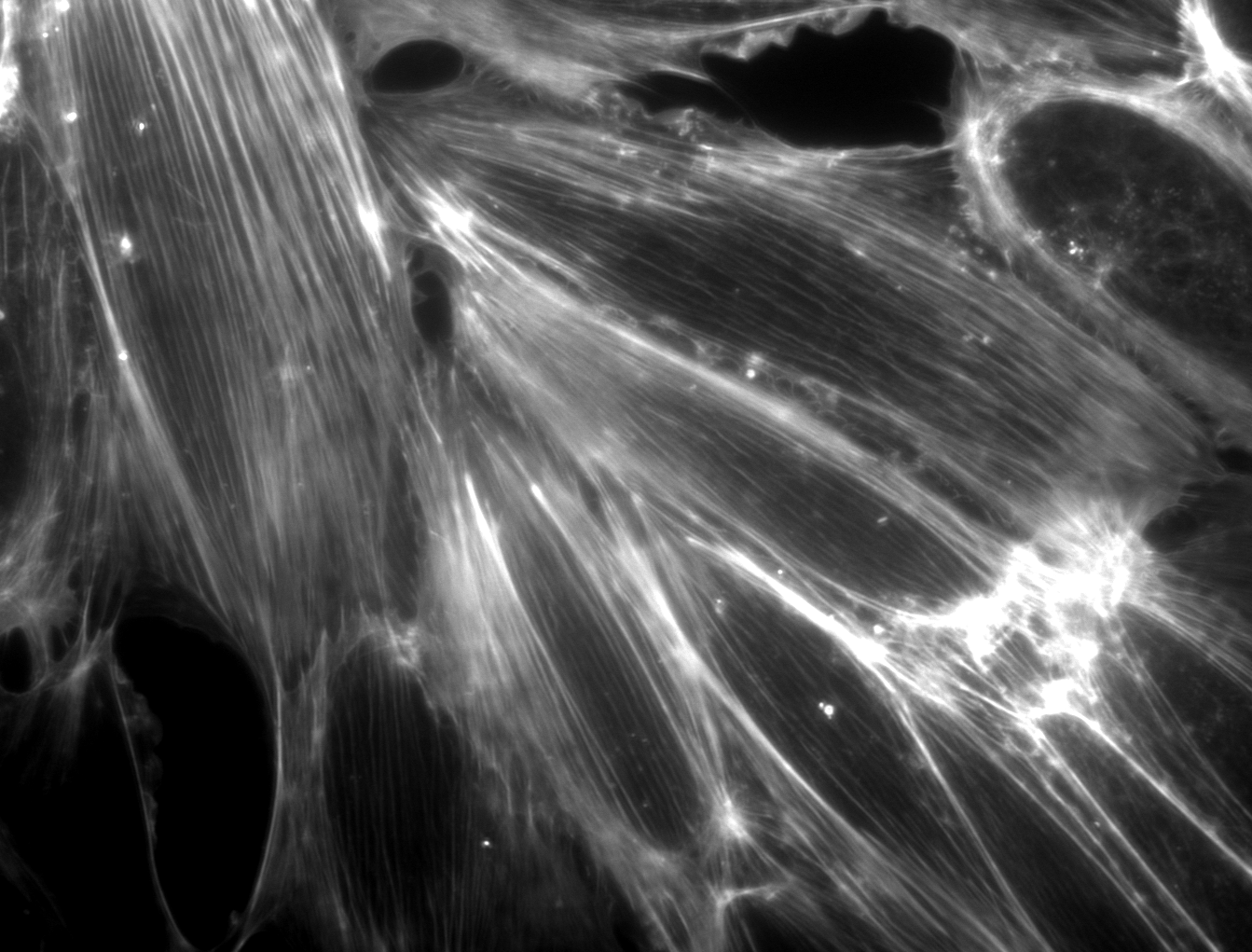

Supplement: Supplementary file 7 — Source data Fig. 2 [file 44321_2024_152_MOESM7_ESM.zip › Figure 2/2I/CCM actin2.tif]

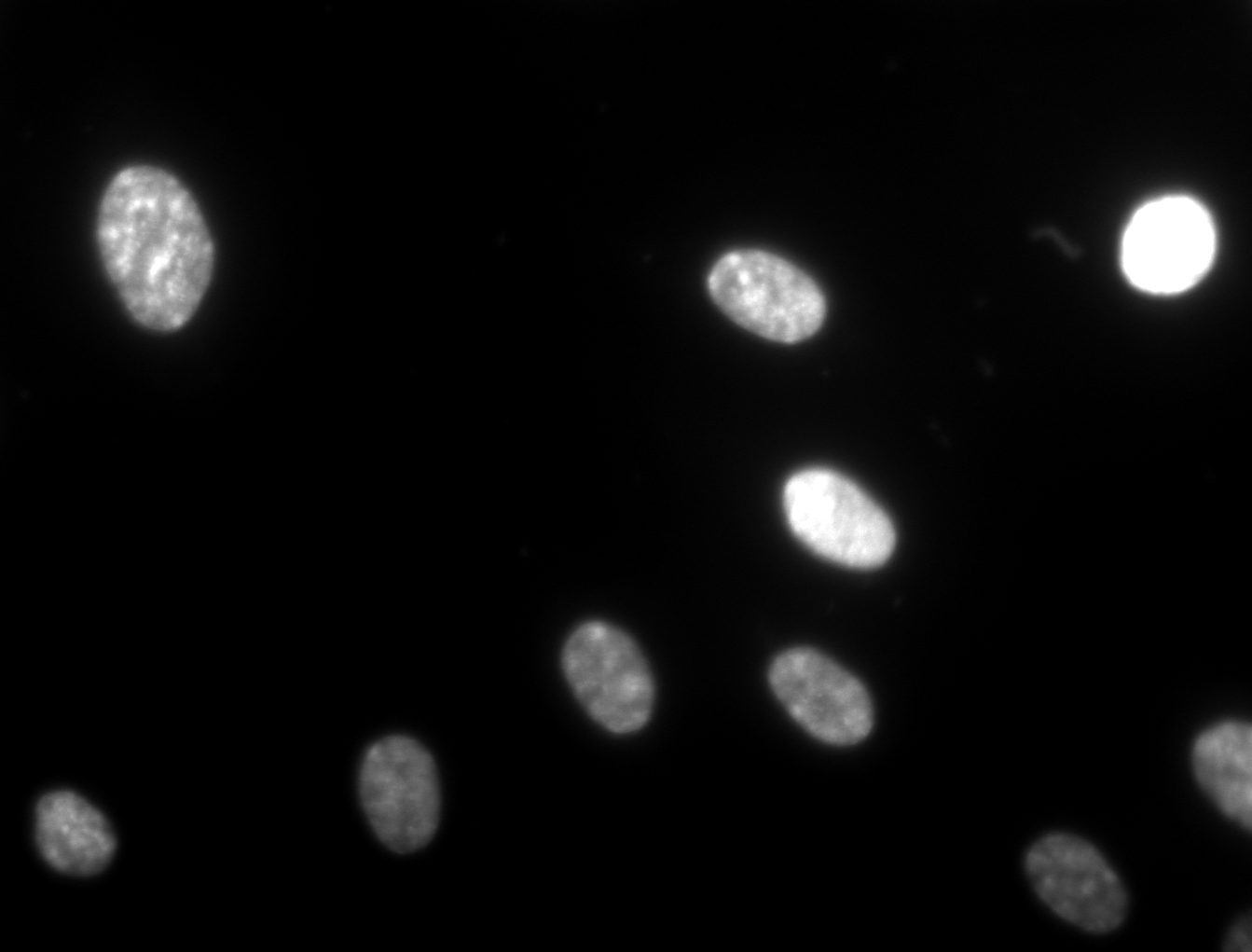

Supplement: Supplementary file 7 — Source data Fig. 2 [file 44321_2024_152_MOESM7_ESM.zip › Figure 2/2I/CCM2 DAPI2.tif]

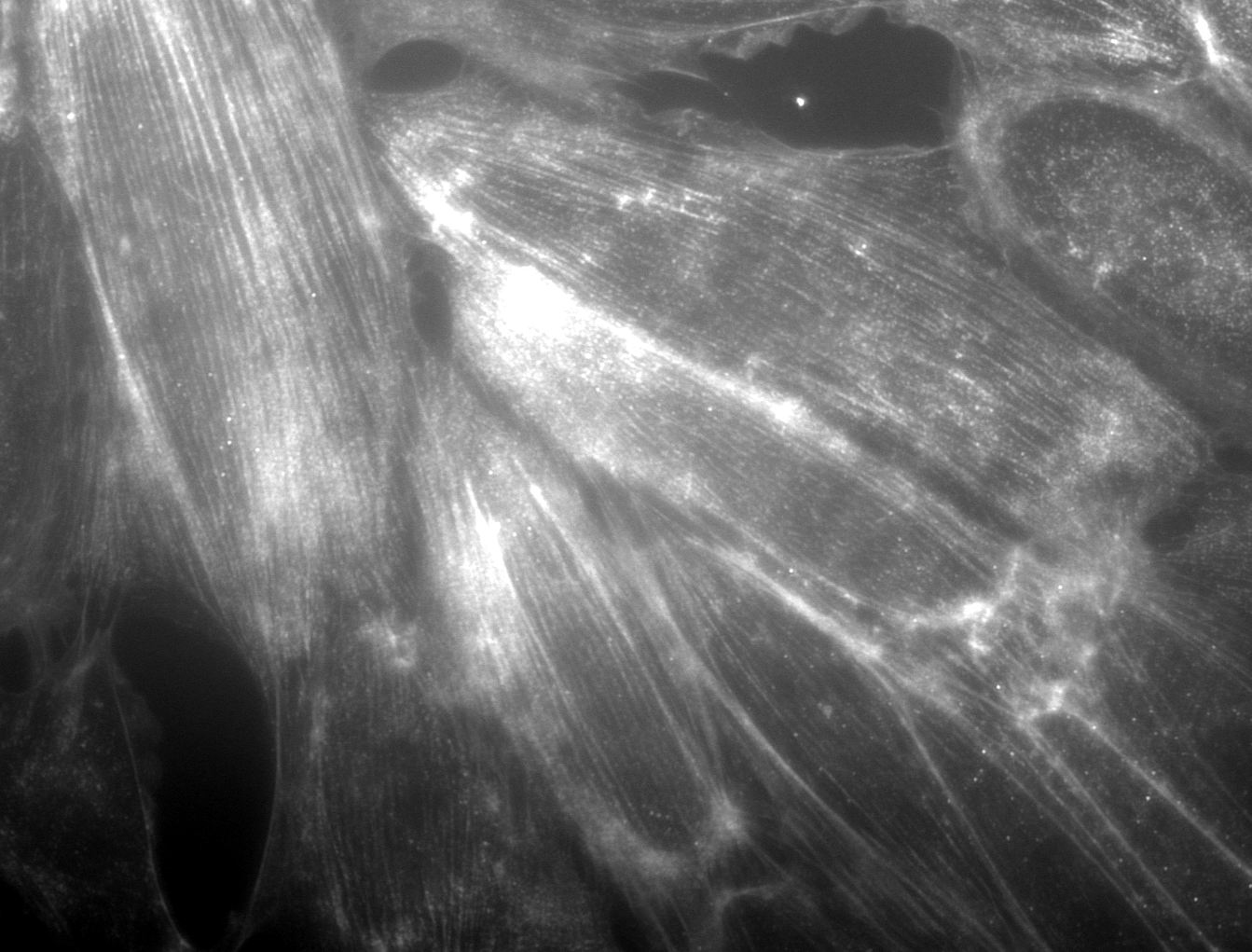

Supplement: Supplementary file 7 — Source data Fig. 2 [file 44321_2024_152_MOESM7_ESM.zip › Figure 2/2I/CCM2 pMLC2.tif]

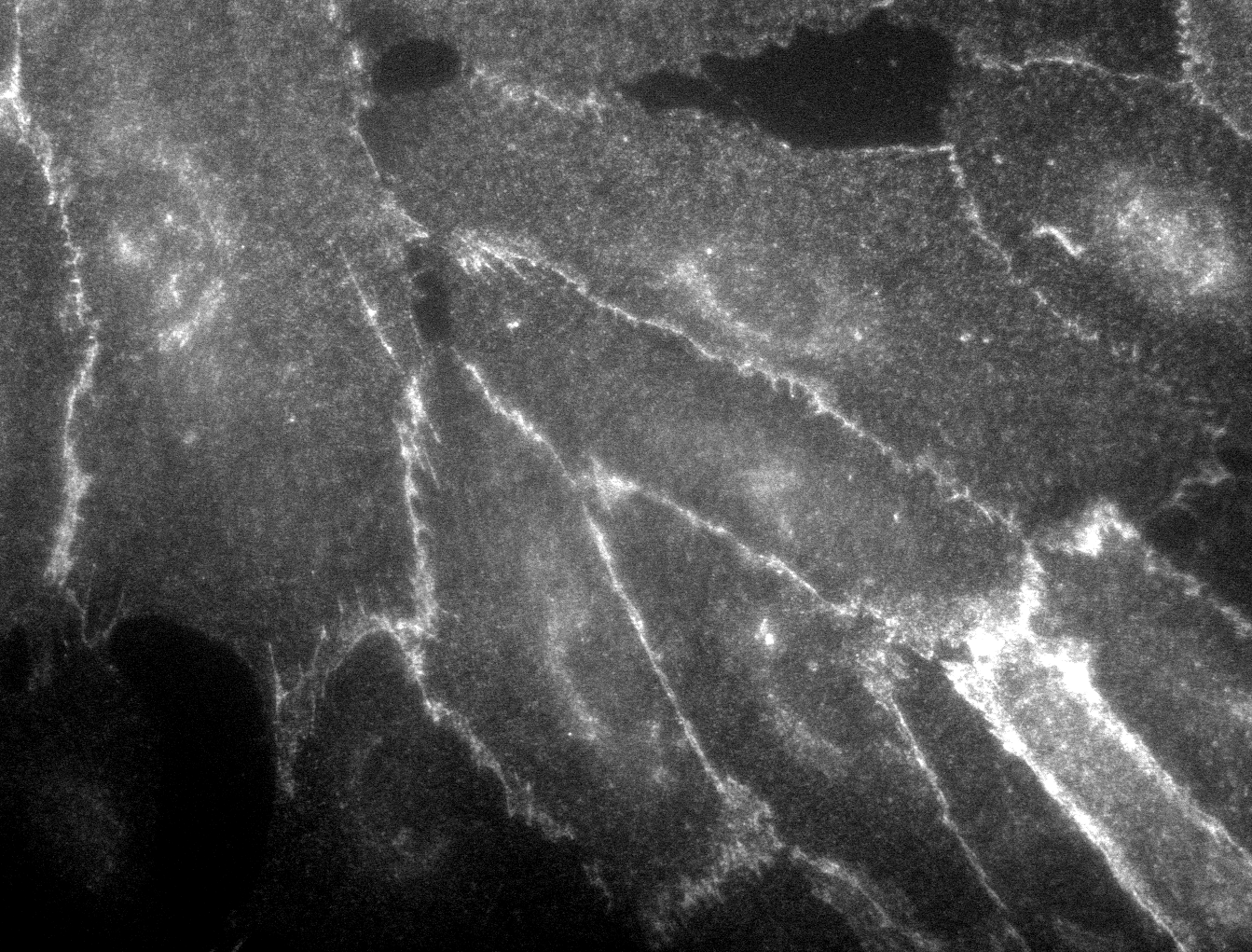

Supplement: Supplementary file 7 — Source data Fig. 2 [file 44321_2024_152_MOESM7_ESM.zip › Figure 2/2I/CCM2 VE-cadherin2.tif]

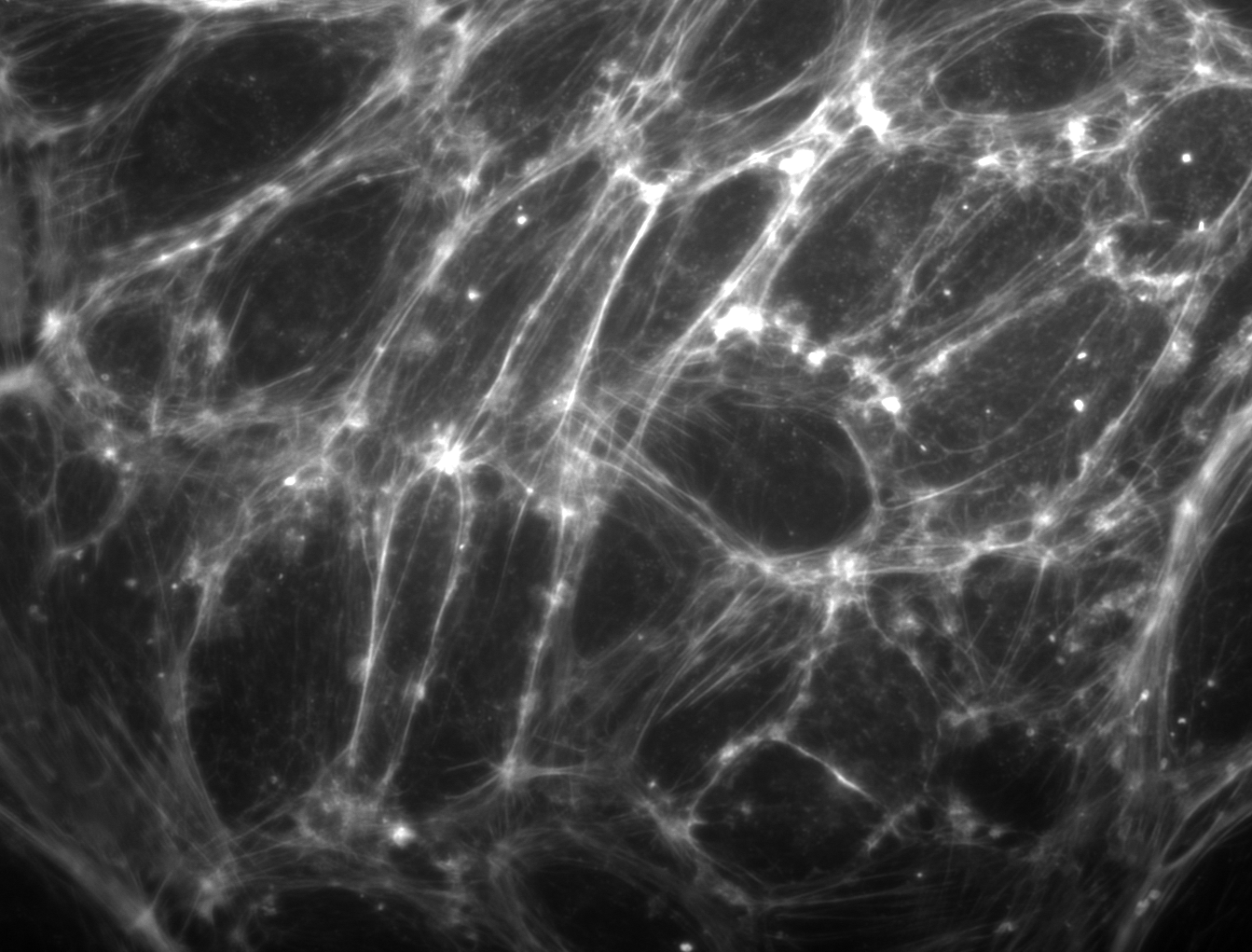

Supplement: Supplementary file 7 — Source data Fig. 2 [file 44321_2024_152_MOESM7_ESM.zip › Figure 2/2J/CCM2 CBX7 actin.tif]

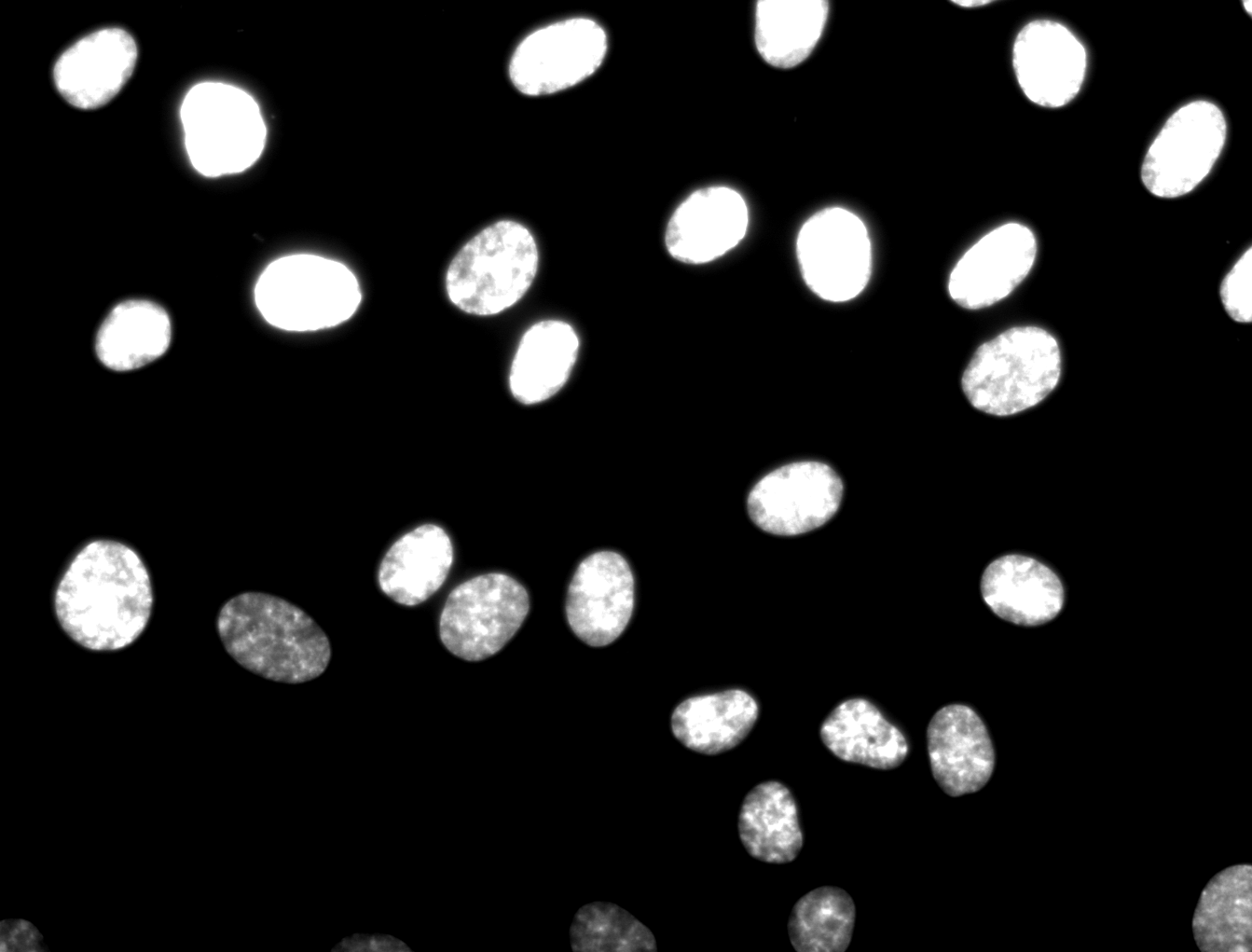

Supplement: Supplementary file 7 — Source data Fig. 2 [file 44321_2024_152_MOESM7_ESM.zip › Figure 2/2J/CCM2 CBX7 DAPI.tif]

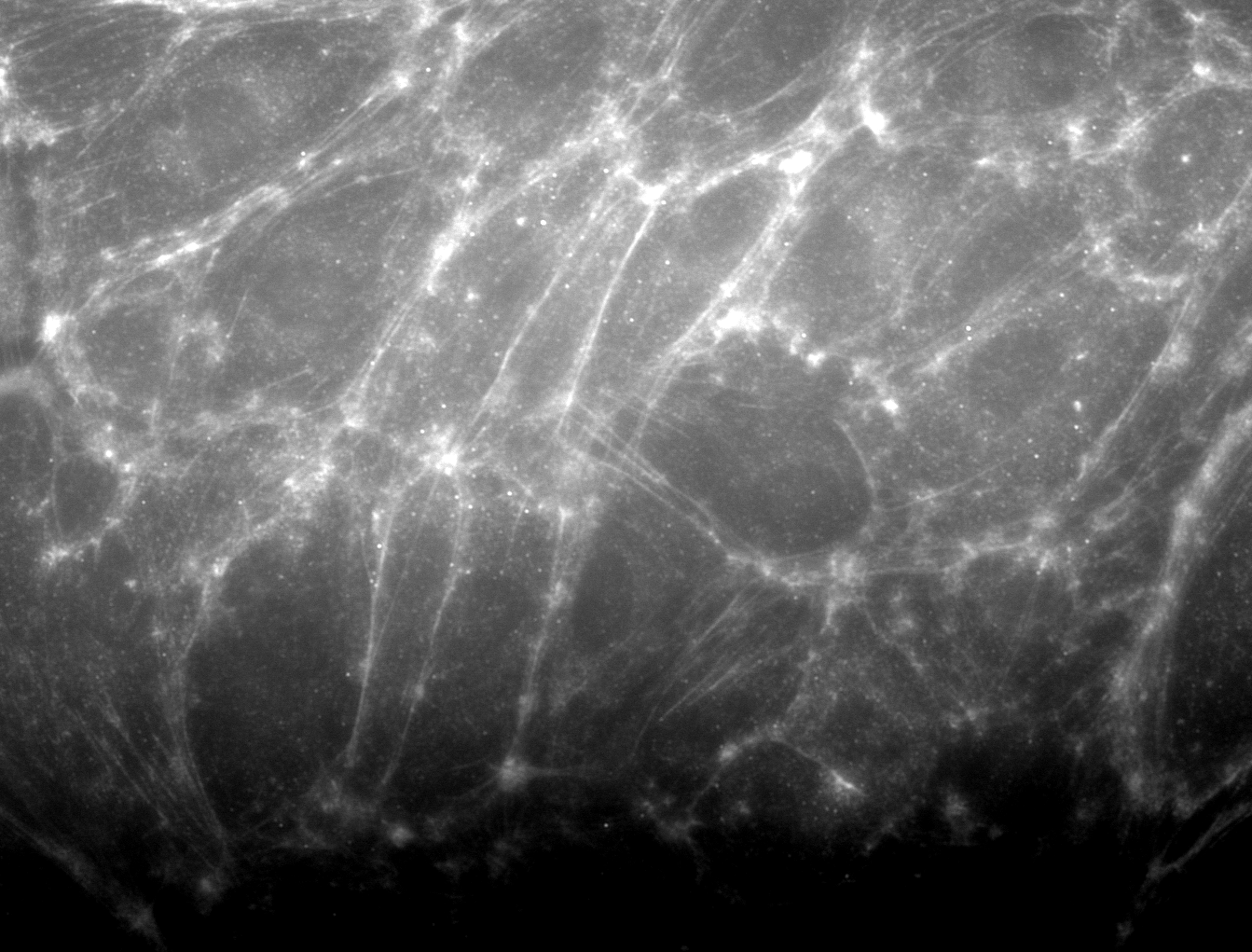

Supplement: Supplementary file 7 — Source data Fig. 2 [file 44321_2024_152_MOESM7_ESM.zip › Figure 2/2J/CCM2 CBX7 pMLC.tif]

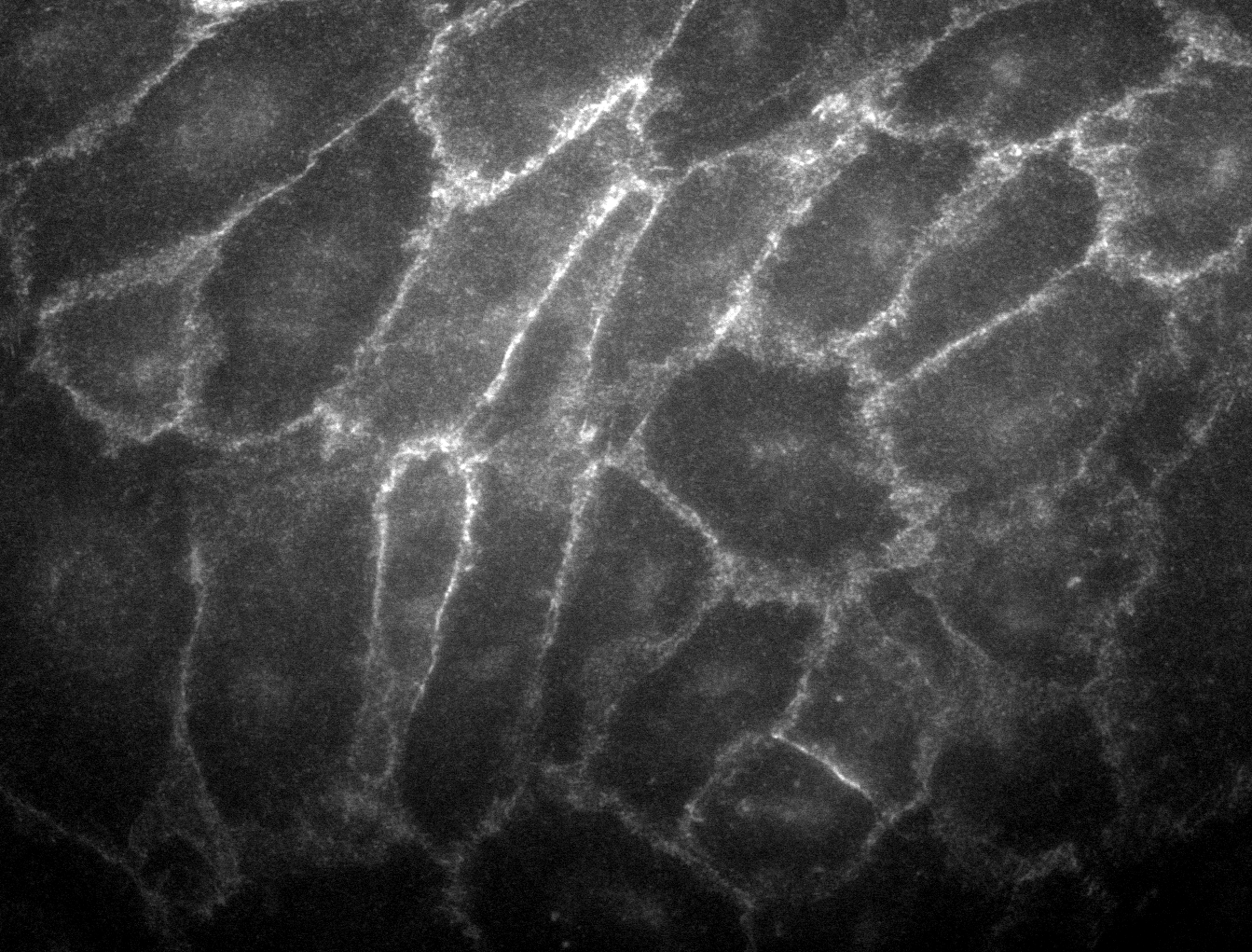

Supplement: Supplementary file 7 — Source data Fig. 2 [file 44321_2024_152_MOESM7_ESM.zip › Figure 2/2J/CCM2 CBX7 VEcadherin.tif]

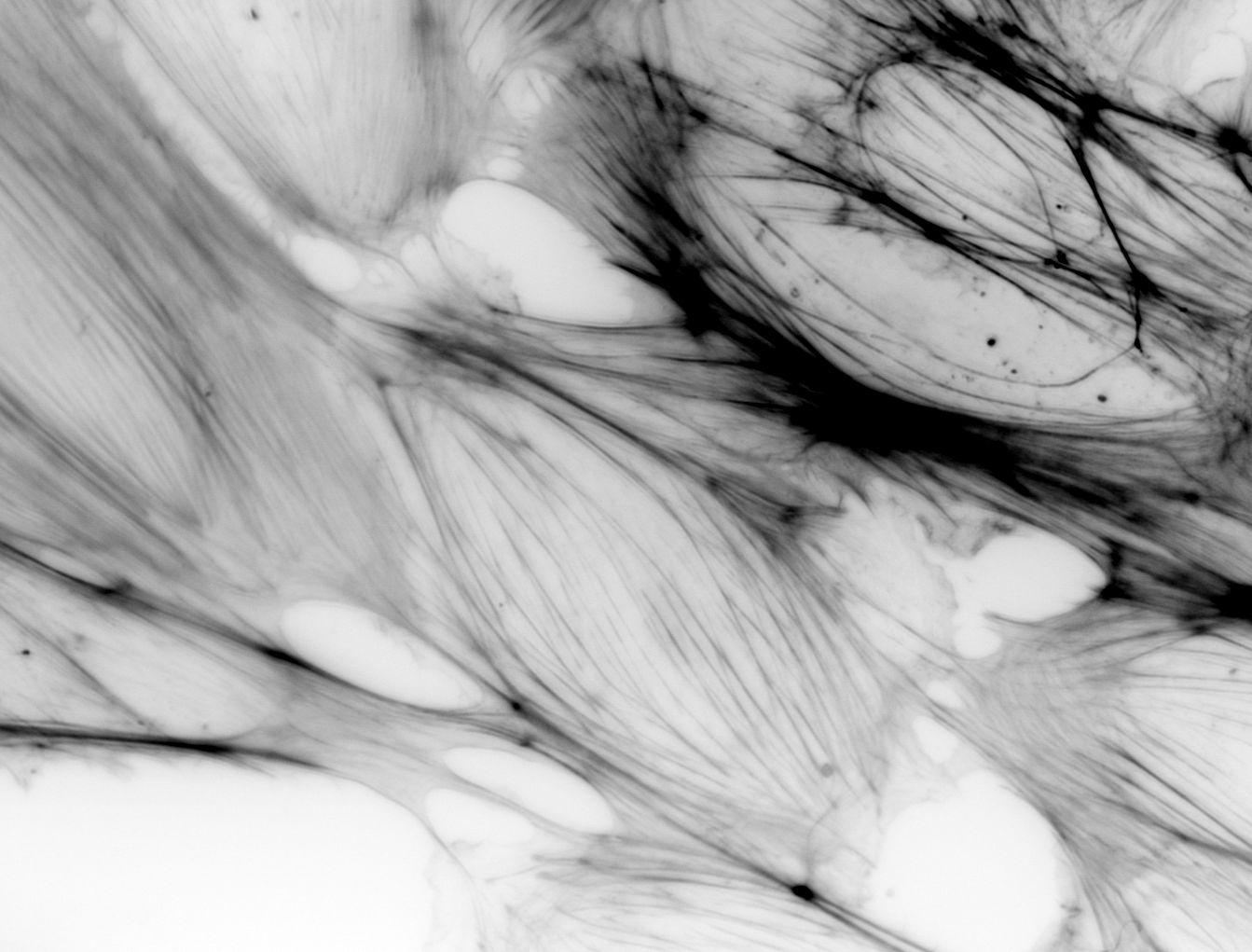

Supplement: Supplementary file 7 — Source data Fig. 2 [file 44321_2024_152_MOESM7_ESM.zip › Figure 2/2K/CCM2 CBX2 actin.tif]

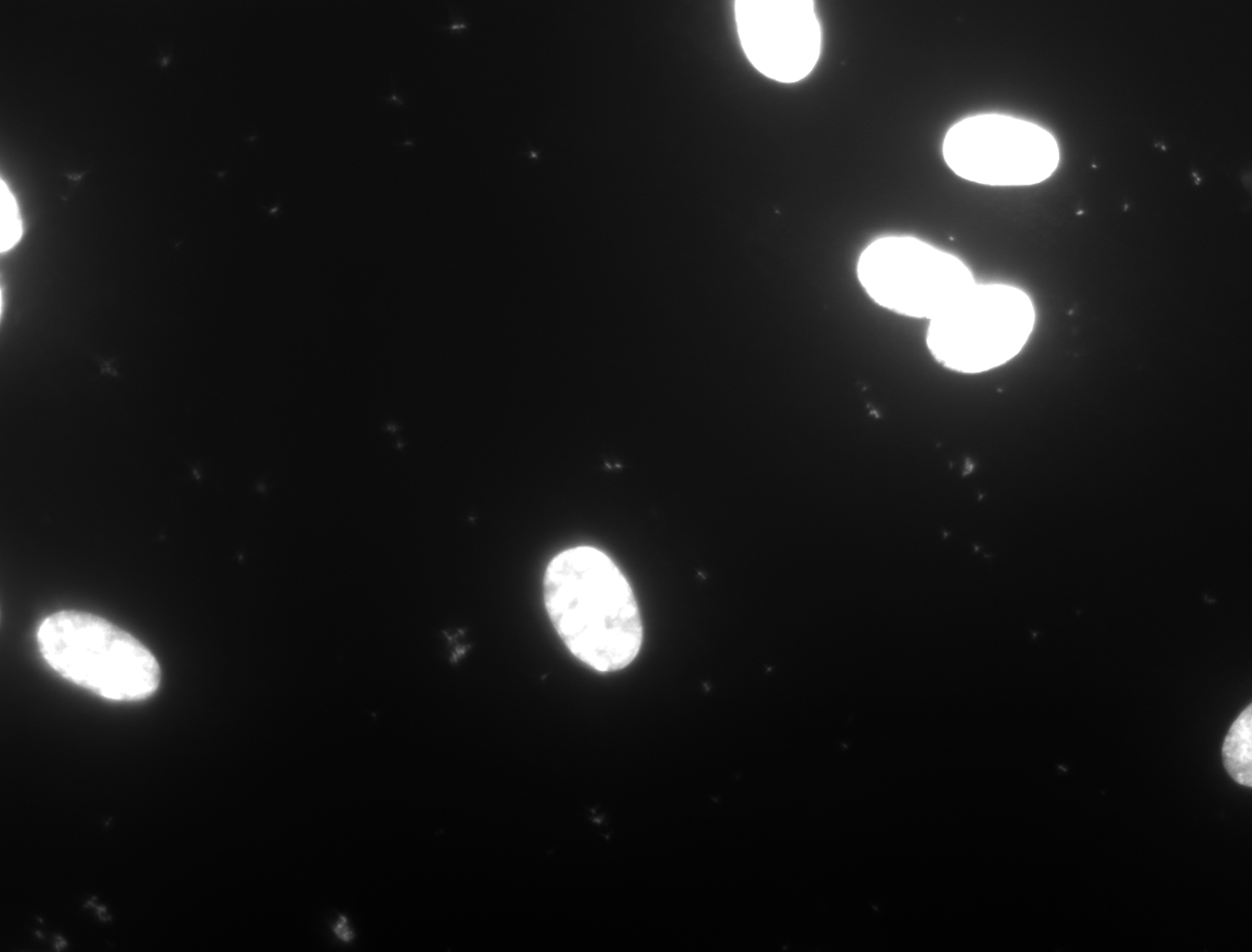

Supplement: Supplementary file 7 — Source data Fig. 2 [file 44321_2024_152_MOESM7_ESM.zip › Figure 2/2K/CCM2 CBX2 DAPI.tif]

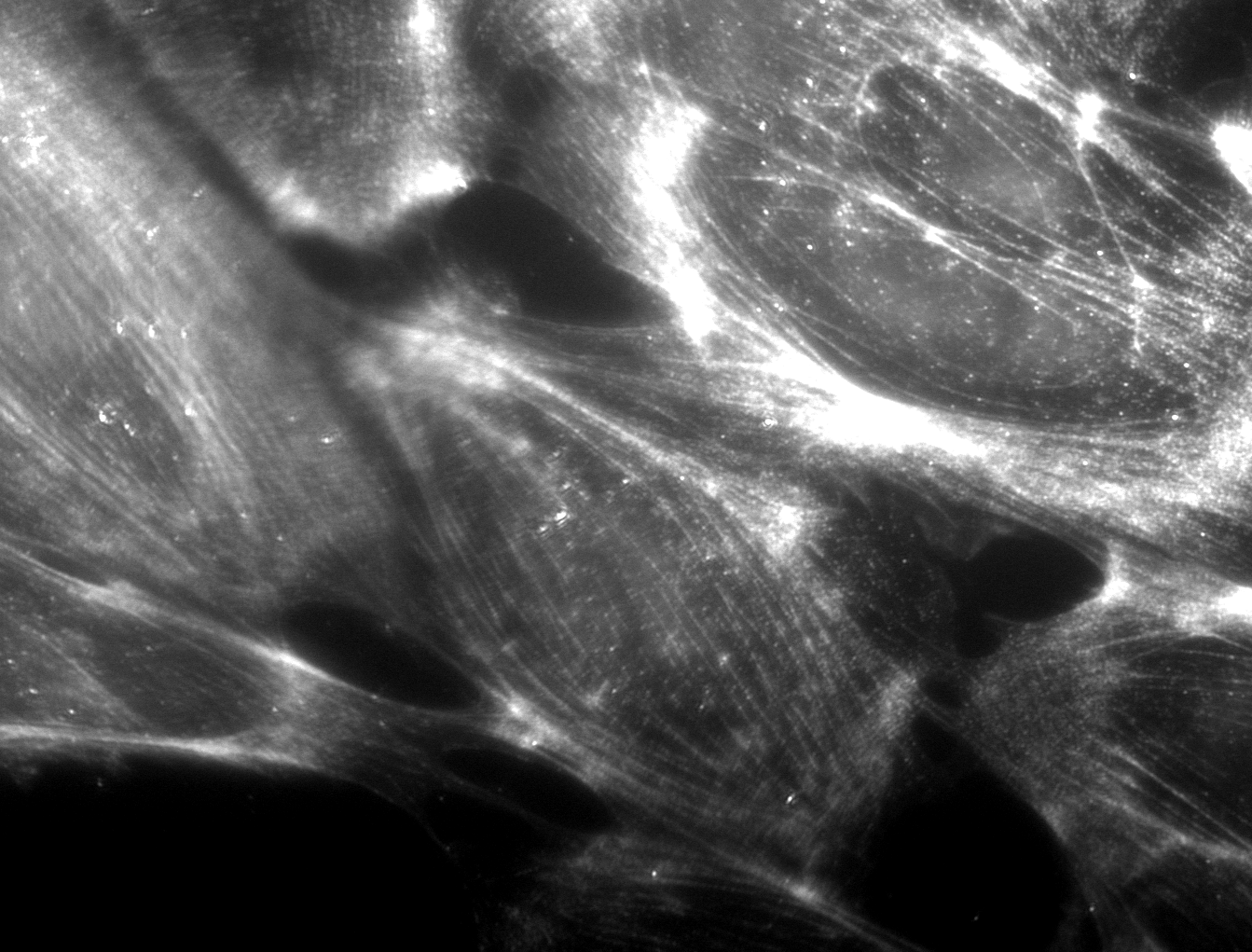

Supplement: Supplementary file 7 — Source data Fig. 2 [file 44321_2024_152_MOESM7_ESM.zip › Figure 2/2K/CCM2 CBX2 pMLC.tif]

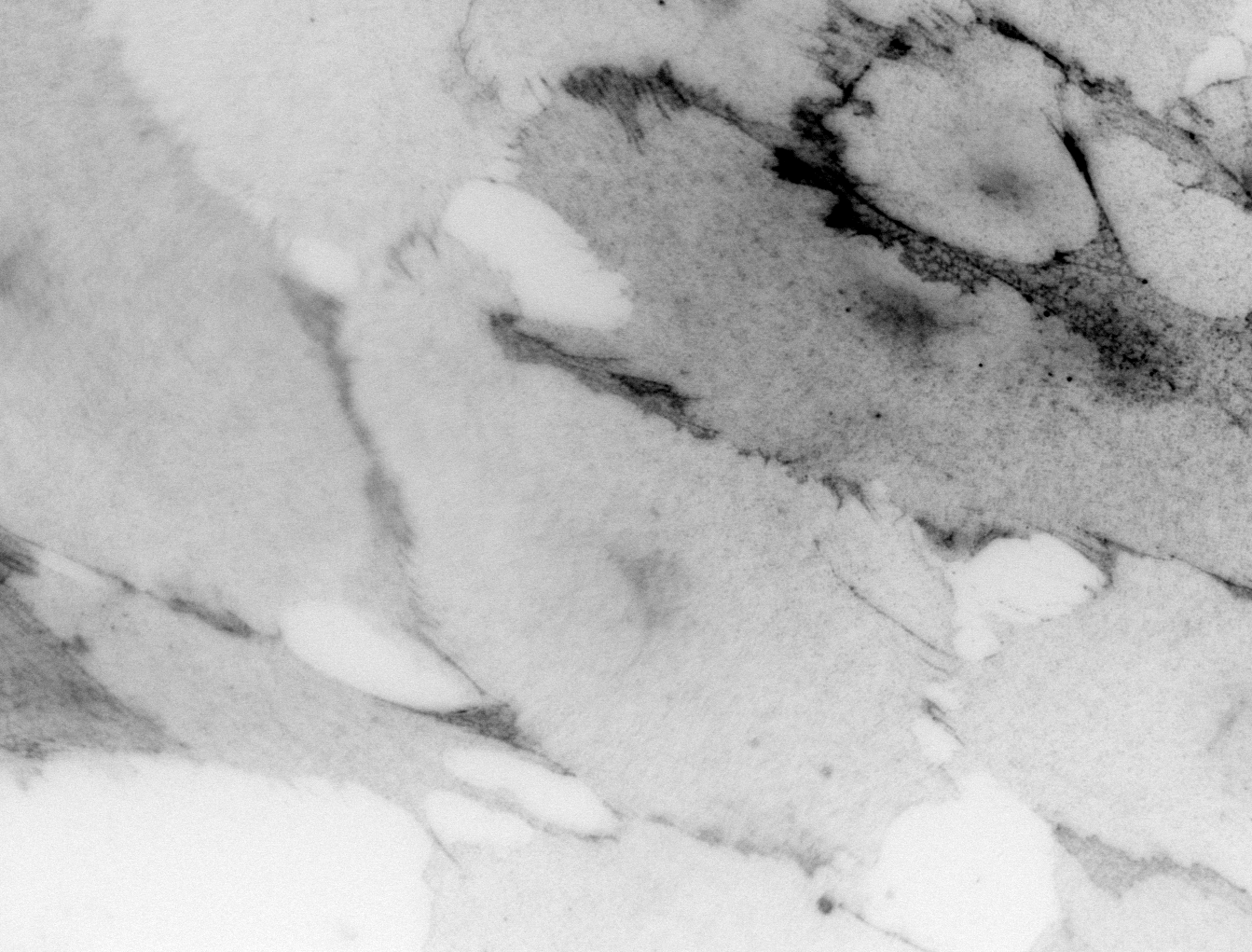

Supplement: Supplementary file 7 — Source data Fig. 2 [file 44321_2024_152_MOESM7_ESM.zip › Figure 2/2K/CCM2 CBX2 VEcadherin.tif]

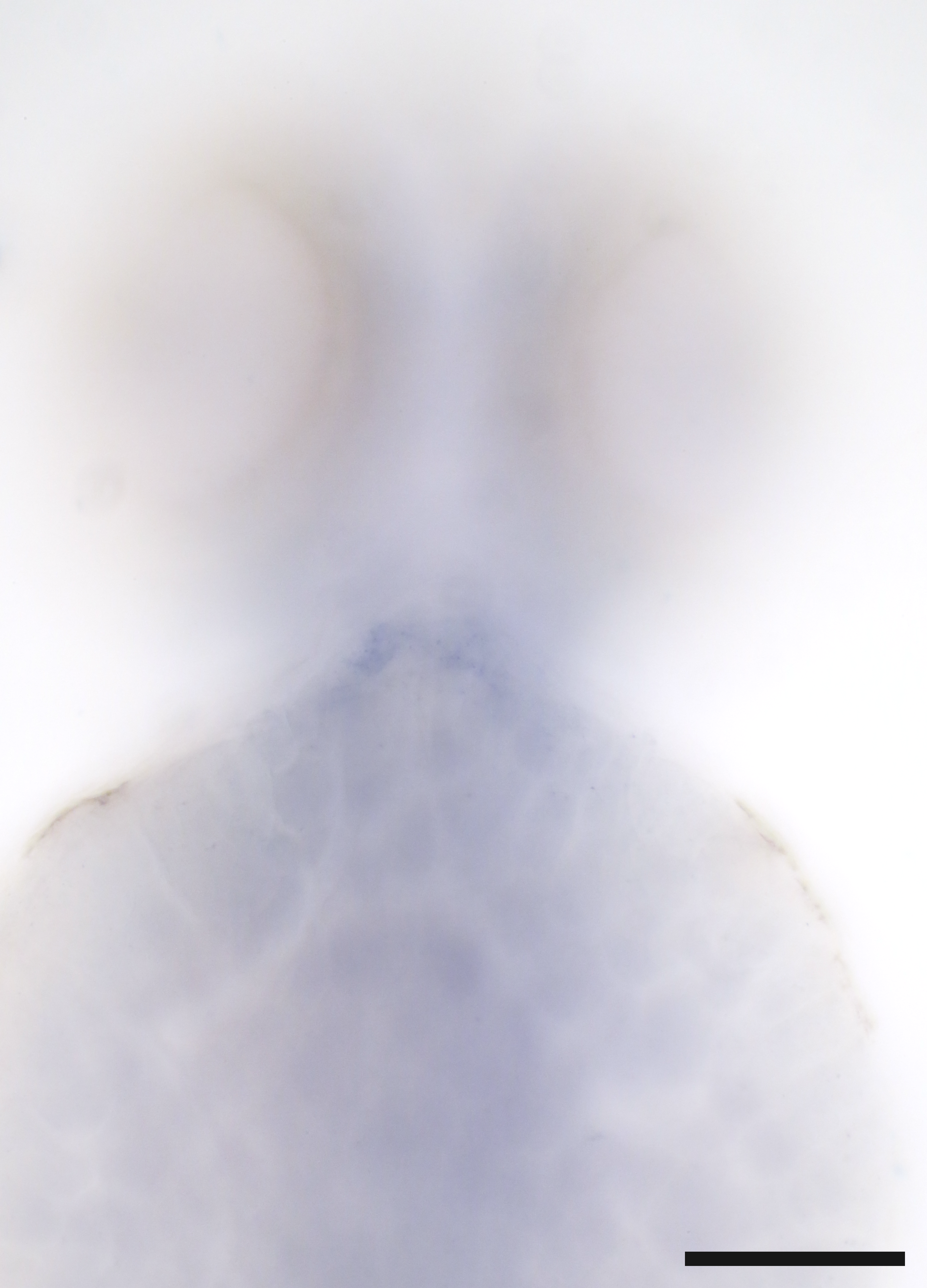

Supplement: Supplementary file 7 — Source data Fig. 2 [file 44321_2024_152_MOESM7_ESM.zip › Figure 2/2M/cbx7a-wish-wt-56hpf-2-1-scale.png]

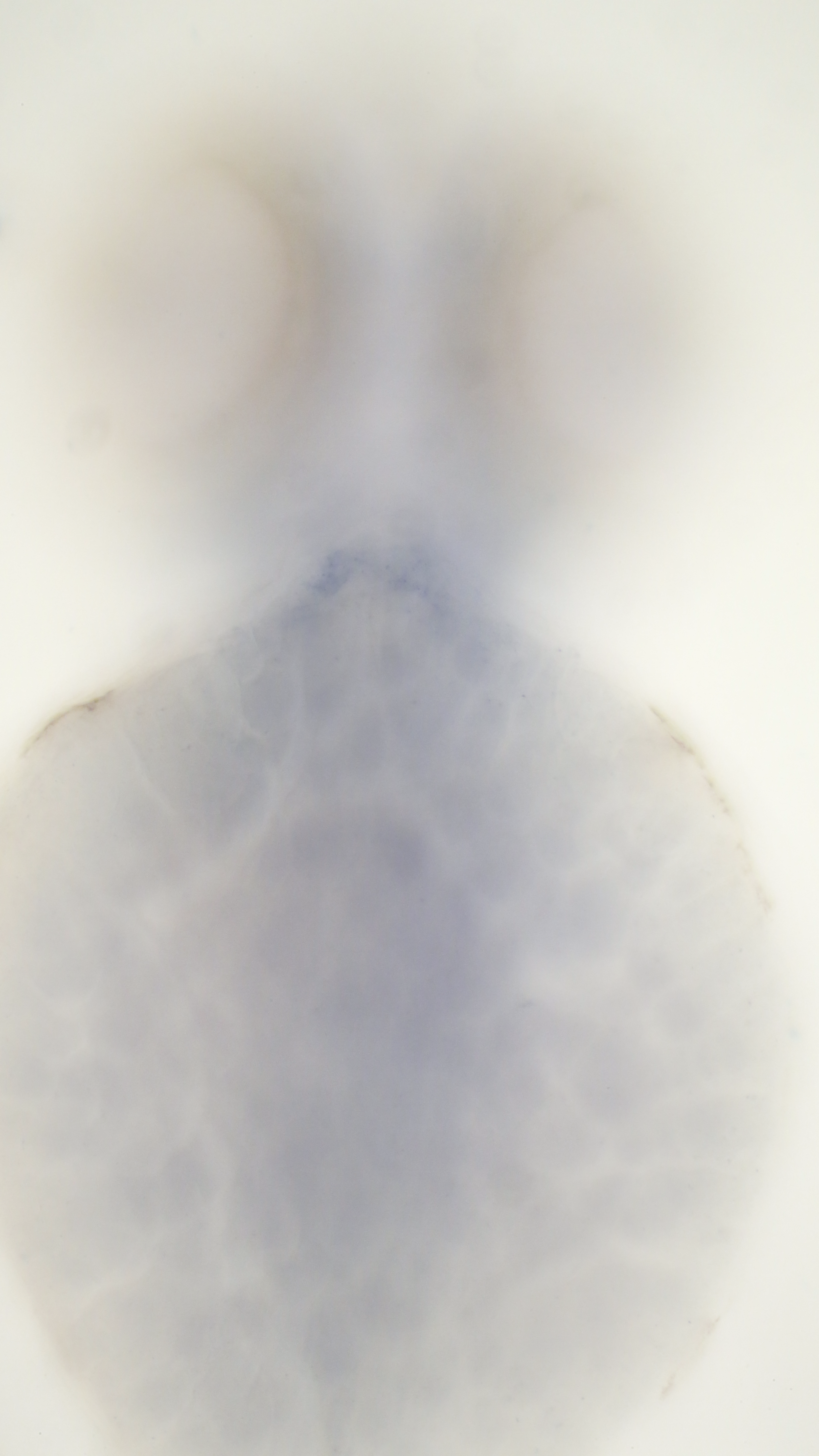

Supplement: Supplementary file 7 — Source data Fig. 2 [file 44321_2024_152_MOESM7_ESM.zip › Figure 2/2M/cbx7a-wish-wt-56hpf-2.tif]

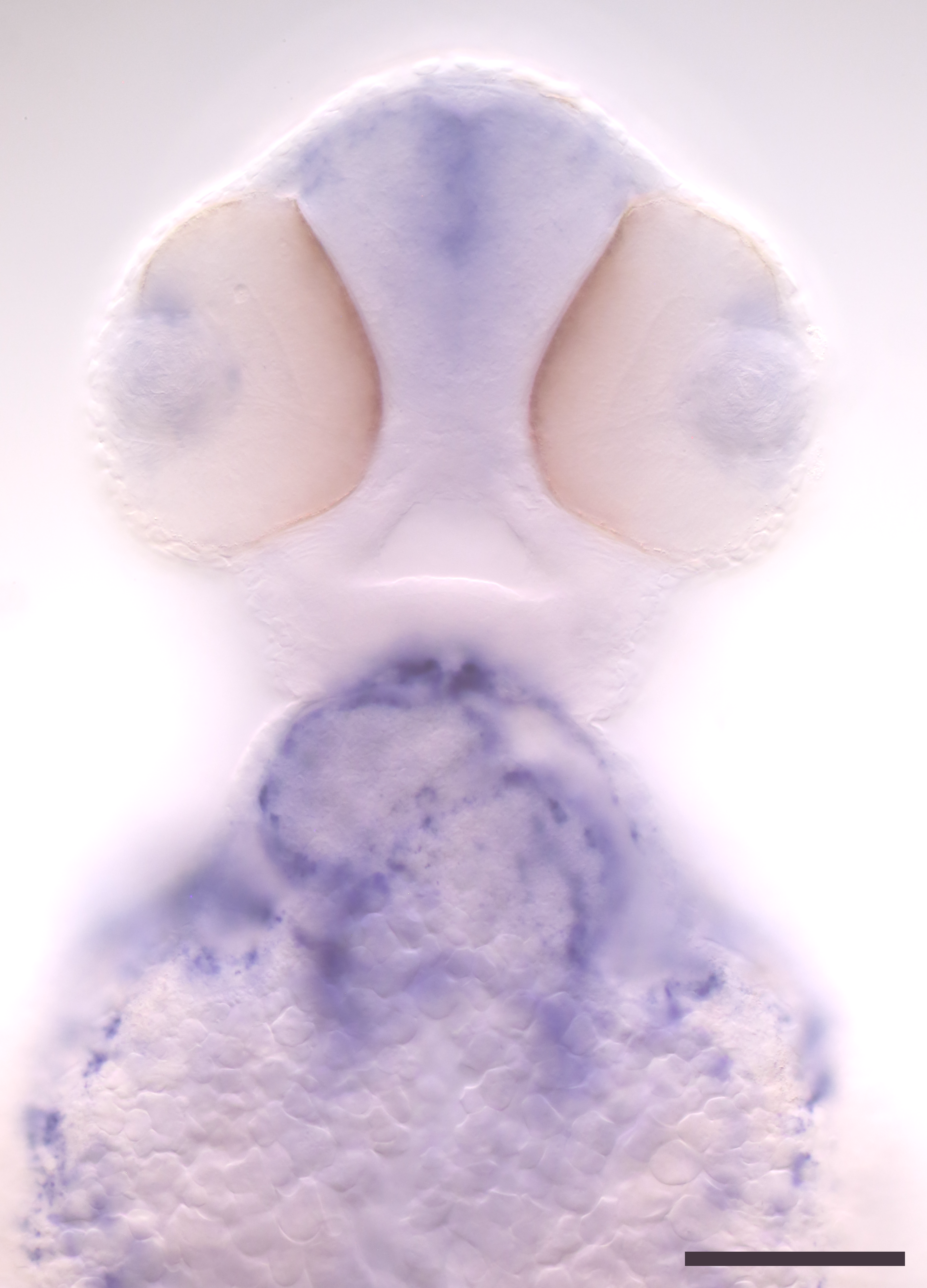

Supplement: Supplementary file 7 — Source data Fig. 2 [file 44321_2024_152_MOESM7_ESM.zip › Figure 2/2N/cbx7awish-ccm2-56hpf (4)-1-scalebar.png]

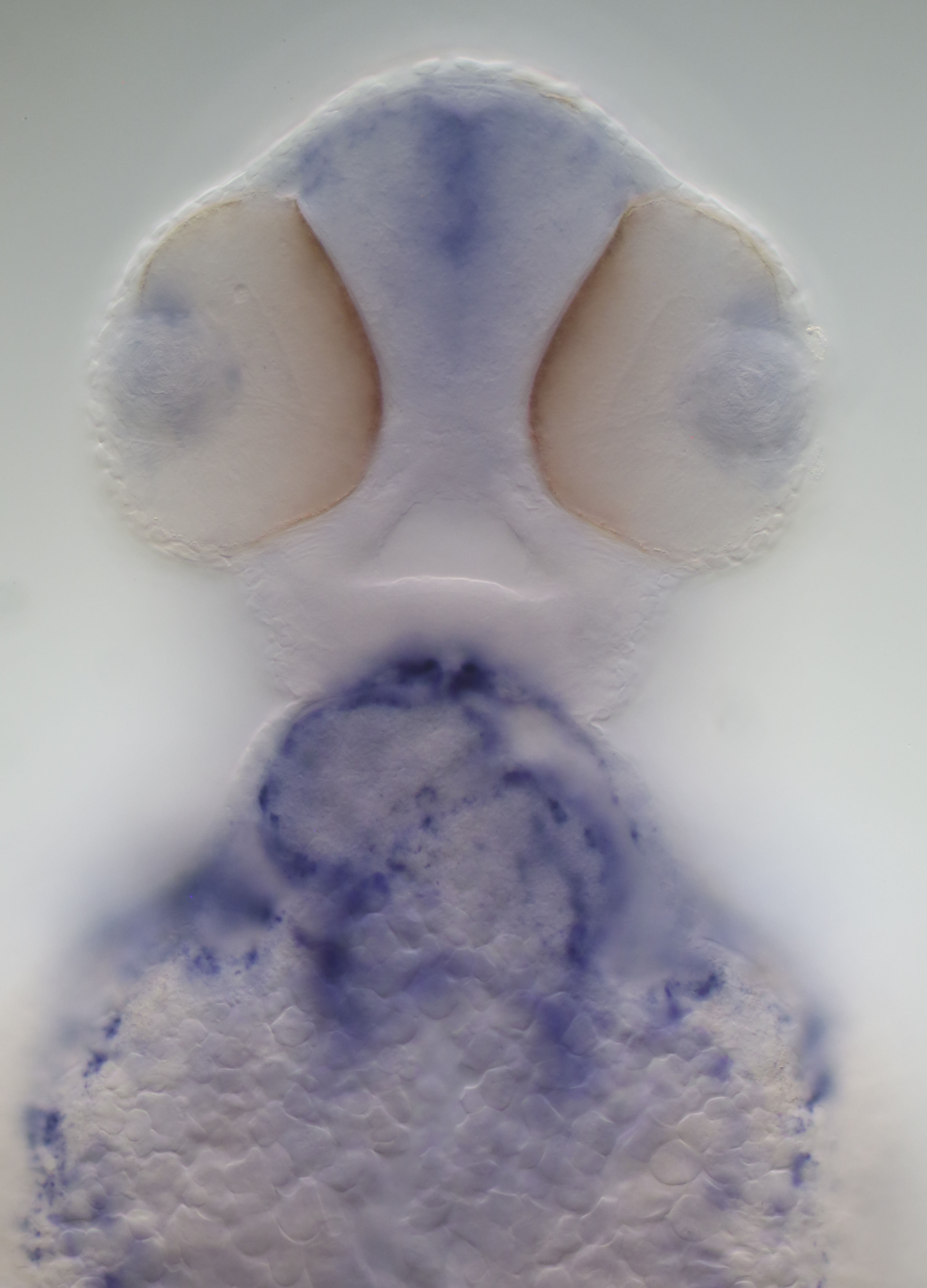

Supplement: Supplementary file 7 — Source data Fig. 2 [file 44321_2024_152_MOESM7_ESM.zip › Figure 2/2N/cbx7awish-ccm2-56hpf (4)-1.tif]

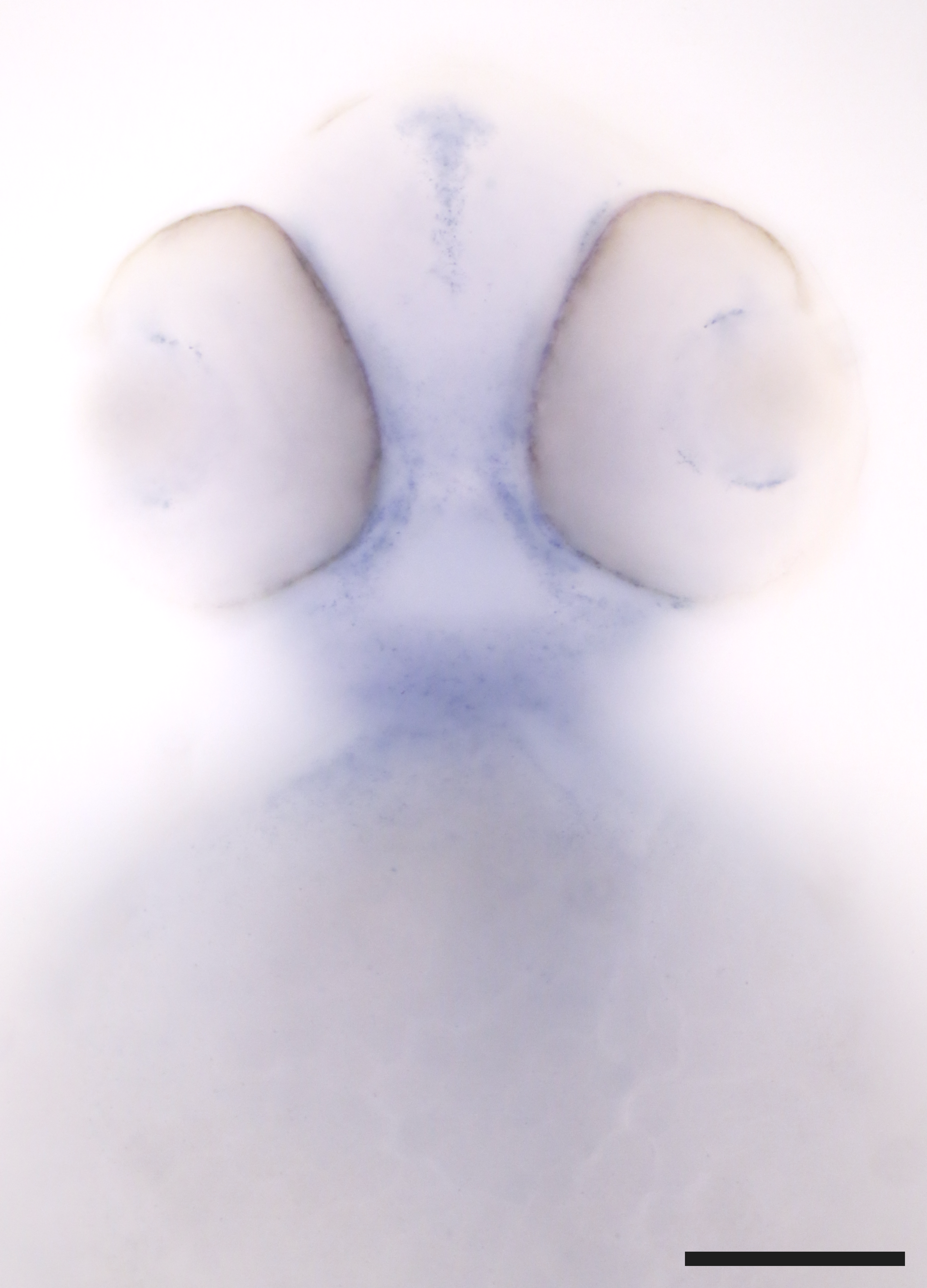

Supplement: Supplementary file 7 — Source data Fig. 2 [file 44321_2024_152_MOESM7_ESM.zip › Figure 2/2O/cbx7a-wish-ccm2-klf2a-b-56hpf-2 (2)-1.png]

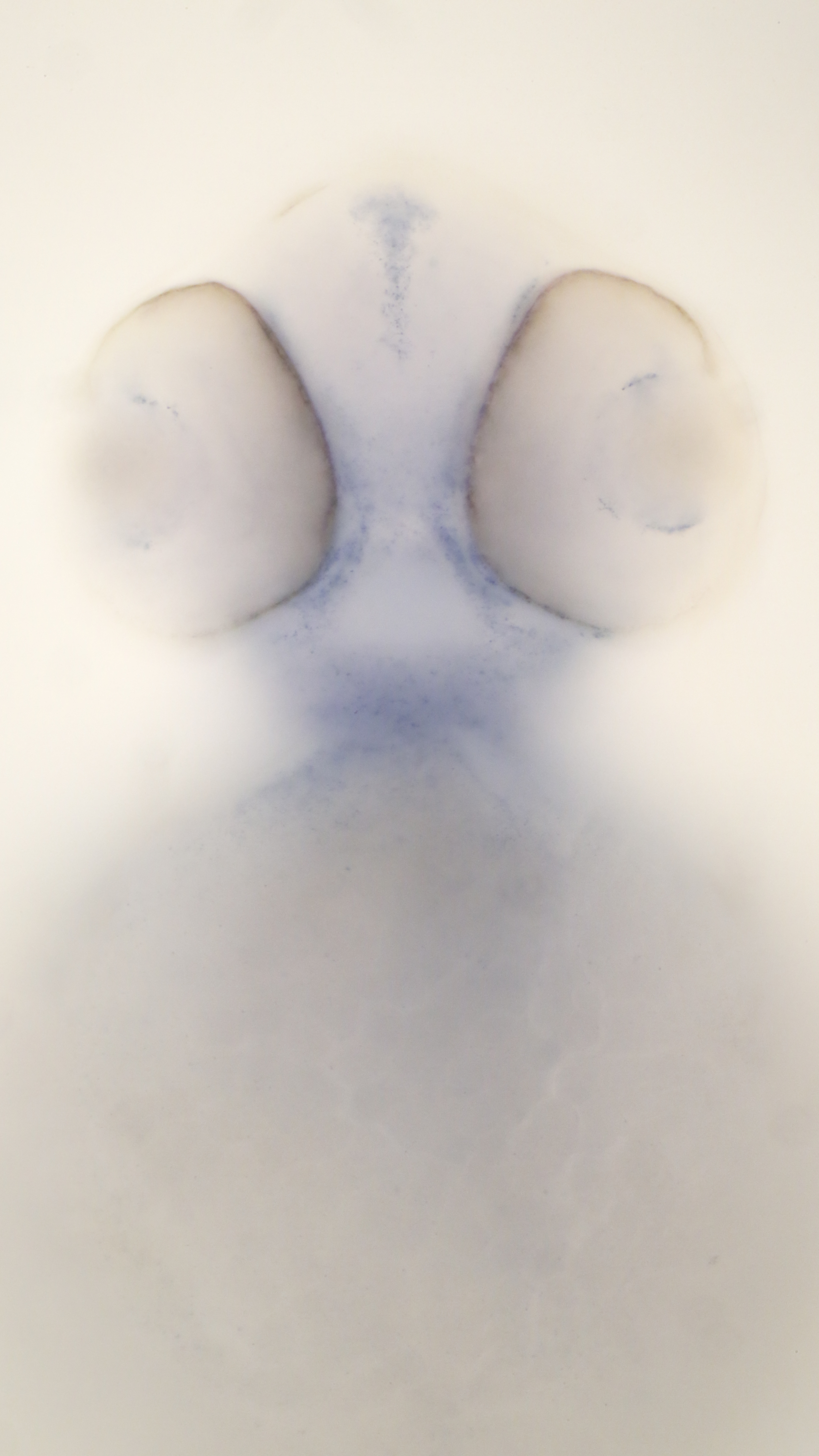

Supplement: Supplementary file 7 — Source data Fig. 2 [file 44321_2024_152_MOESM7_ESM.zip › Figure 2/2O/cbx7a-wish-ccm2-klf2a-b-56hpf-2 (2).tif]

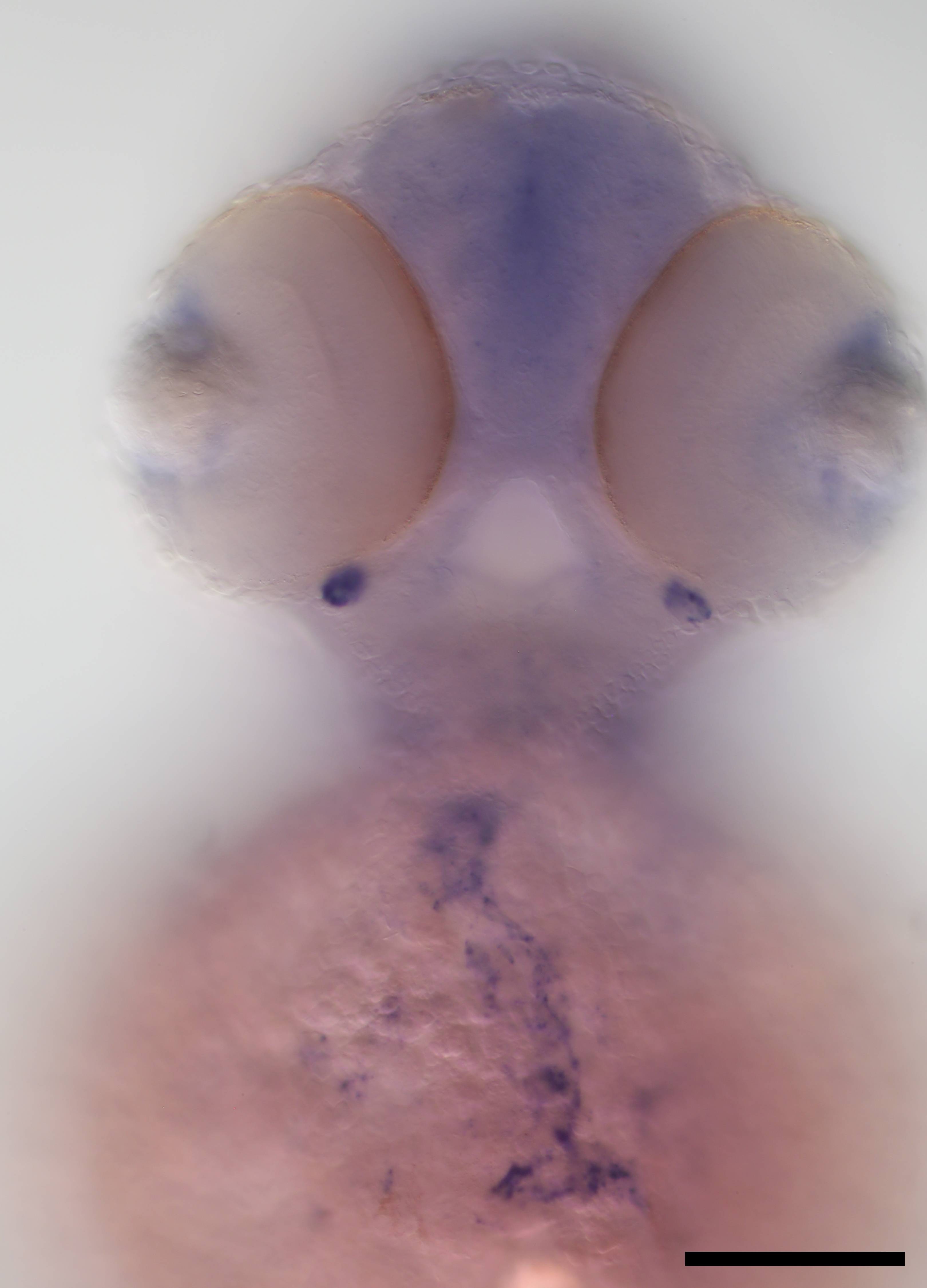

Supplement: Supplementary file 7 — Source data Fig. 2 [file 44321_2024_152_MOESM7_ESM.zip › Figure 2/2P/cbx7awish-ccm2-klf2a-56hpf (2)-1-scalebar.png]

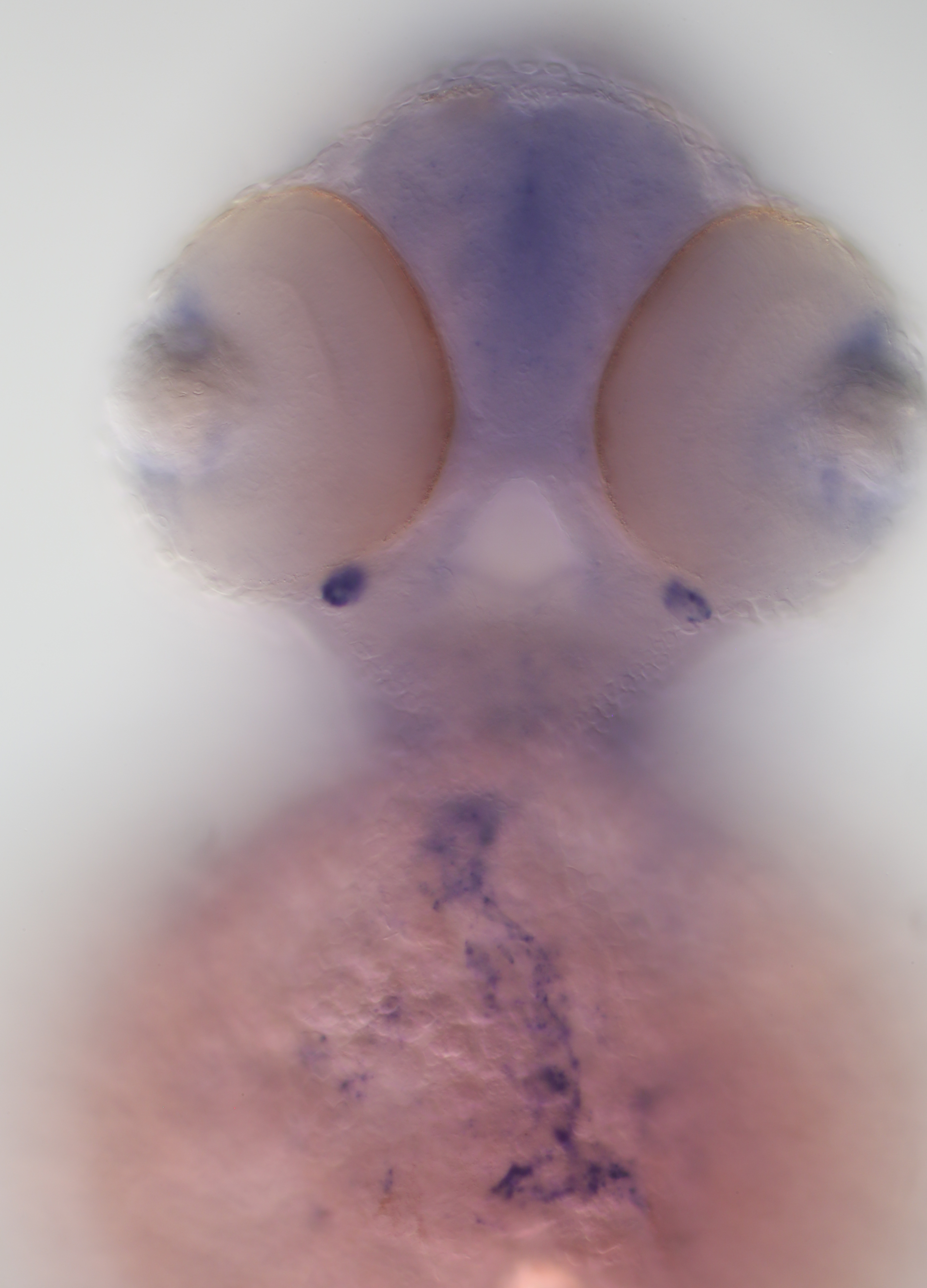

Supplement: Supplementary file 7 — Source data Fig. 2 [file 44321_2024_152_MOESM7_ESM.zip › Figure 2/2P/cbx7awish-ccm2-klf2a-56hpf (2)-1.tif]

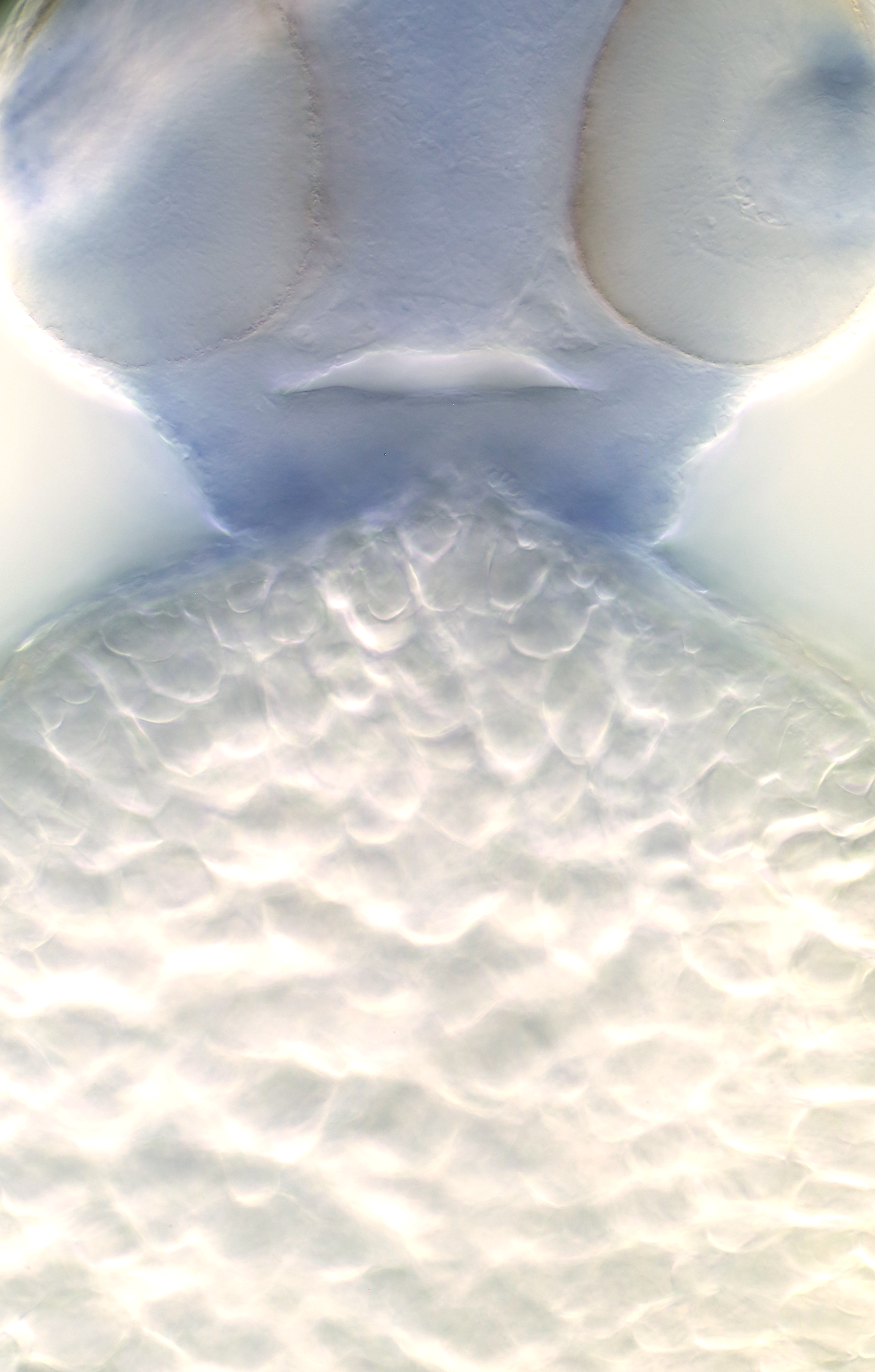

Supplement: Supplementary file 7 — Source data Fig. 2 [file 44321_2024_152_MOESM7_ESM.zip › Figure 2/2Q/cbx7a-WISH-fli1a-klf2aOE-56hpf-2 (2)-2.tif]

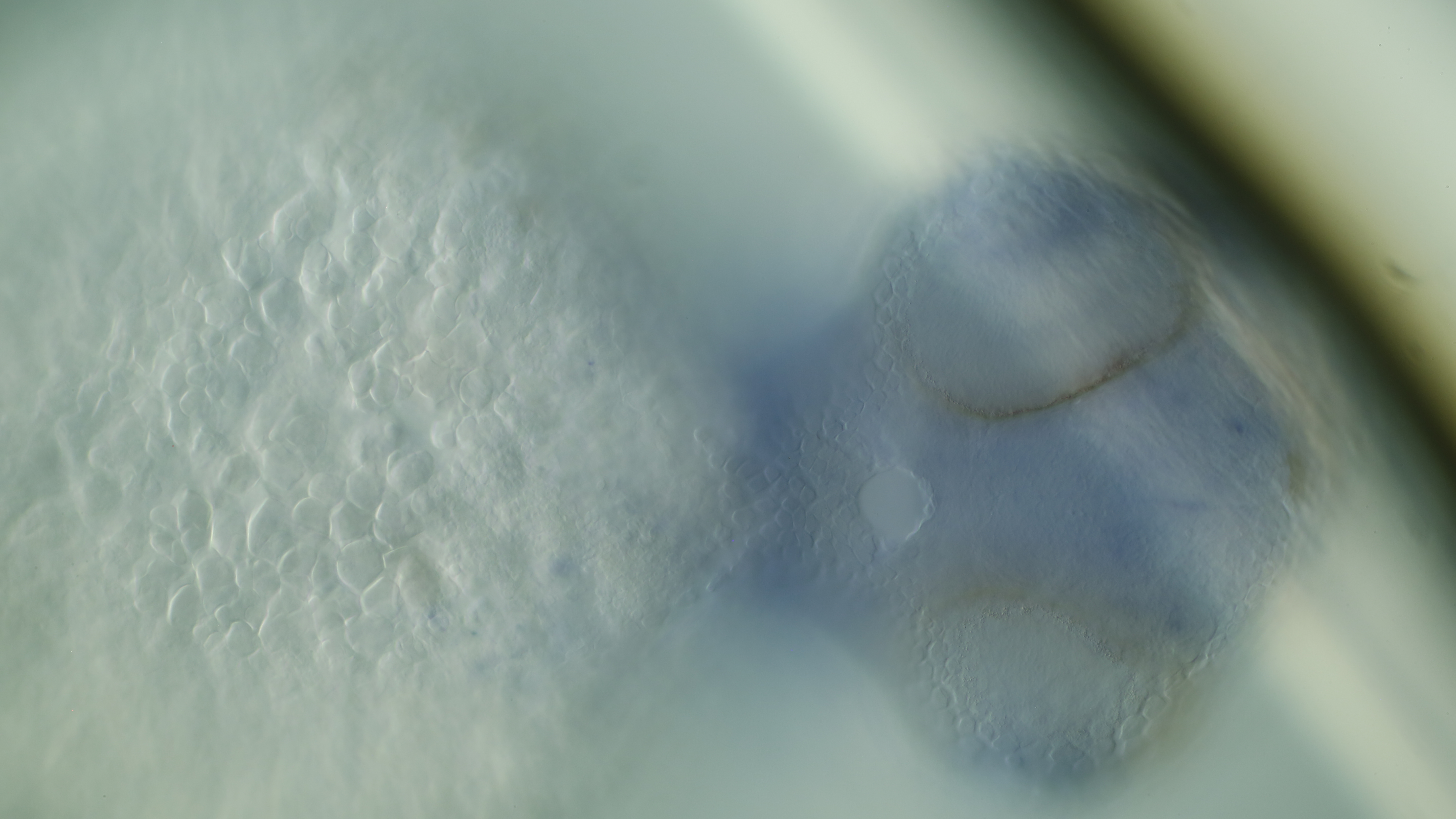

Supplement: Supplementary file 7 — Source data Fig. 2 [file 44321_2024_152_MOESM7_ESM.zip › Figure 2/2Q/cbx7a-WISH-fli1a-klf2aOE-56hpf-2.JPG]

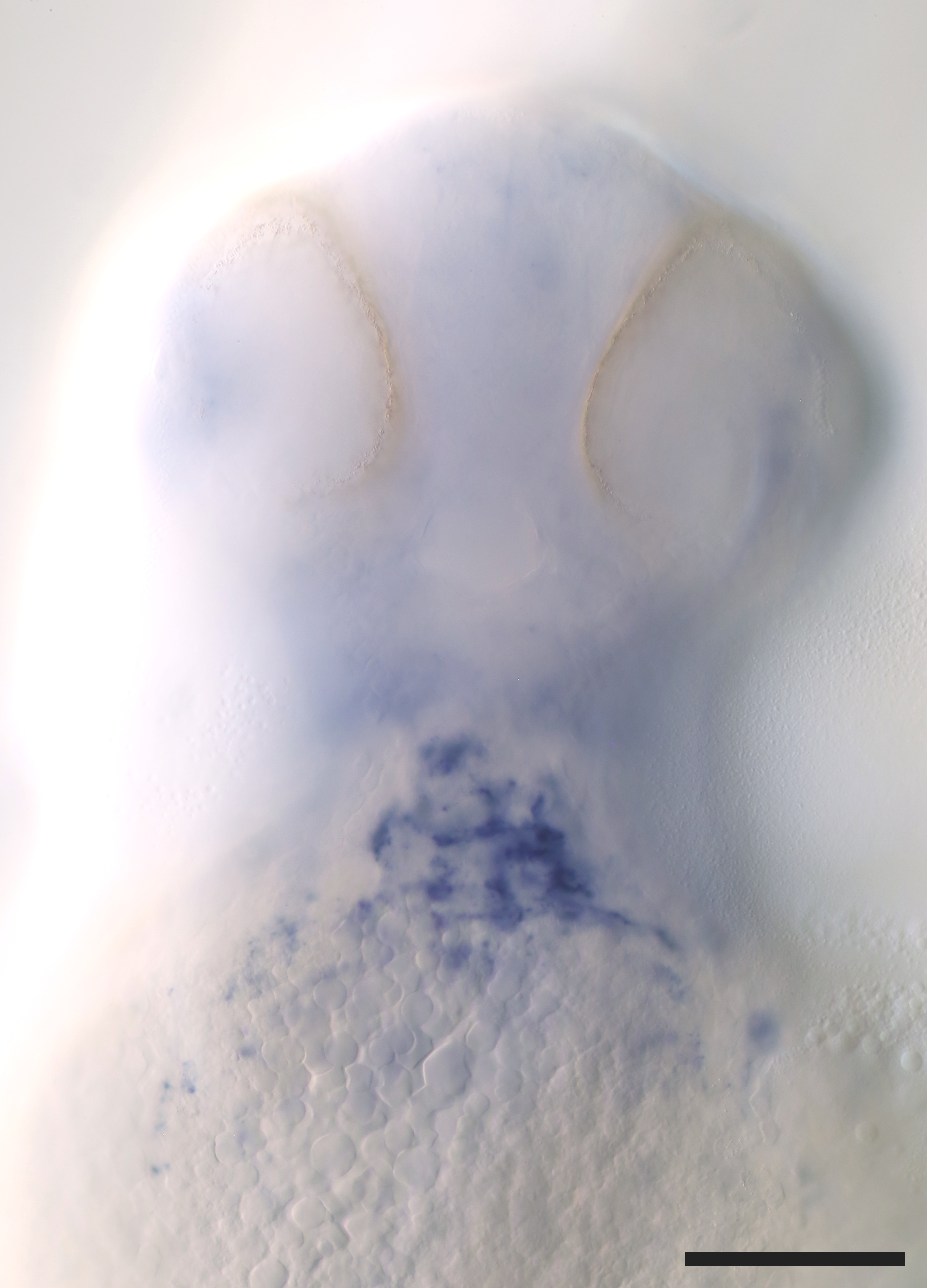

Supplement: Supplementary file 7 — Source data Fig. 2 [file 44321_2024_152_MOESM7_ESM.zip › Figure 2/2R/cbx7a-WISH-fli1a-klf2aOE-56hpf (2)-1-scalebar.png]

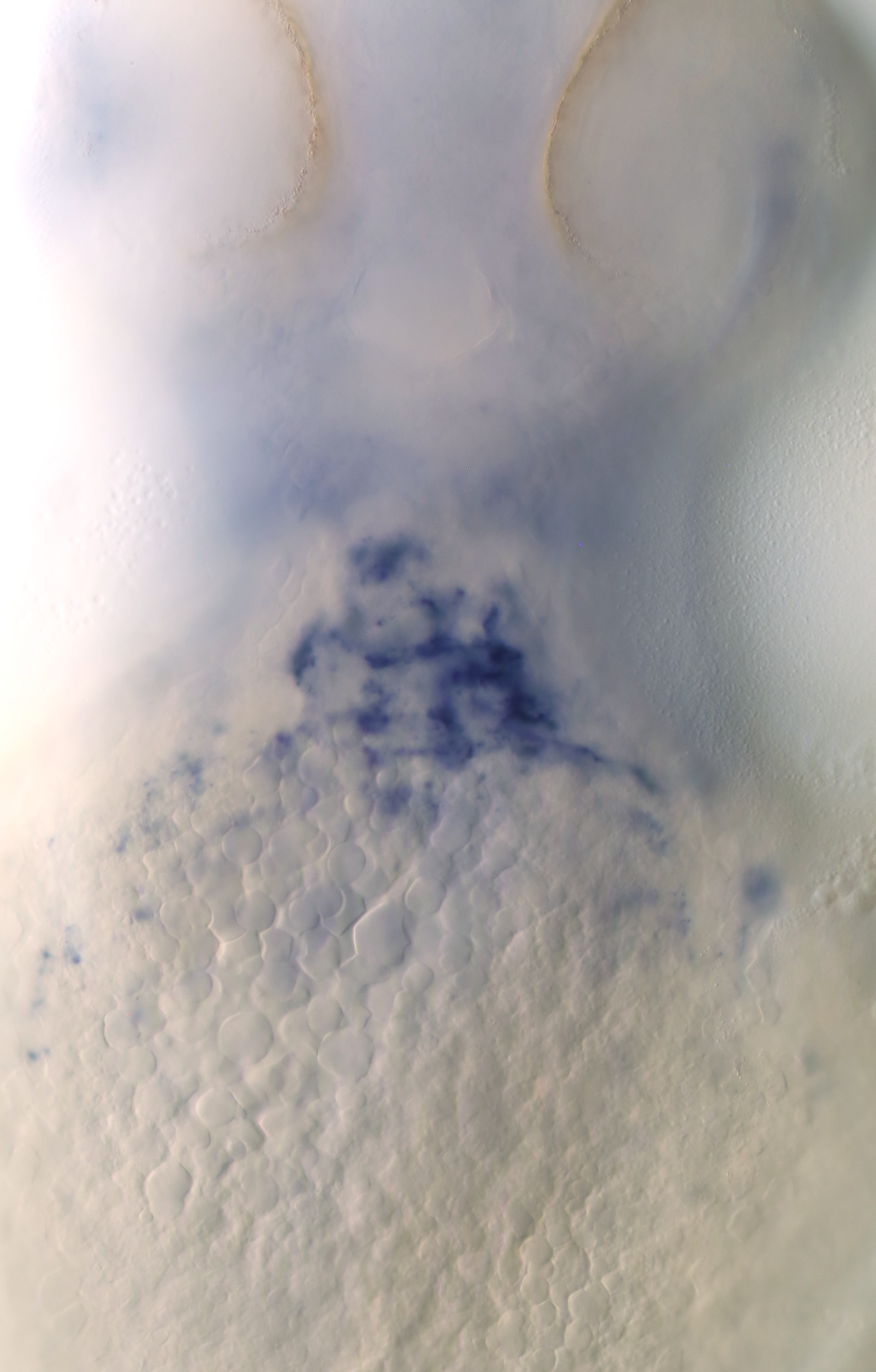

Supplement: Supplementary file 7 — Source data Fig. 2 [file 44321_2024_152_MOESM7_ESM.zip › Figure 2/2R/cbx7a-WISH-fli1a-klf2aOE-56hpf (2)-2.tif]

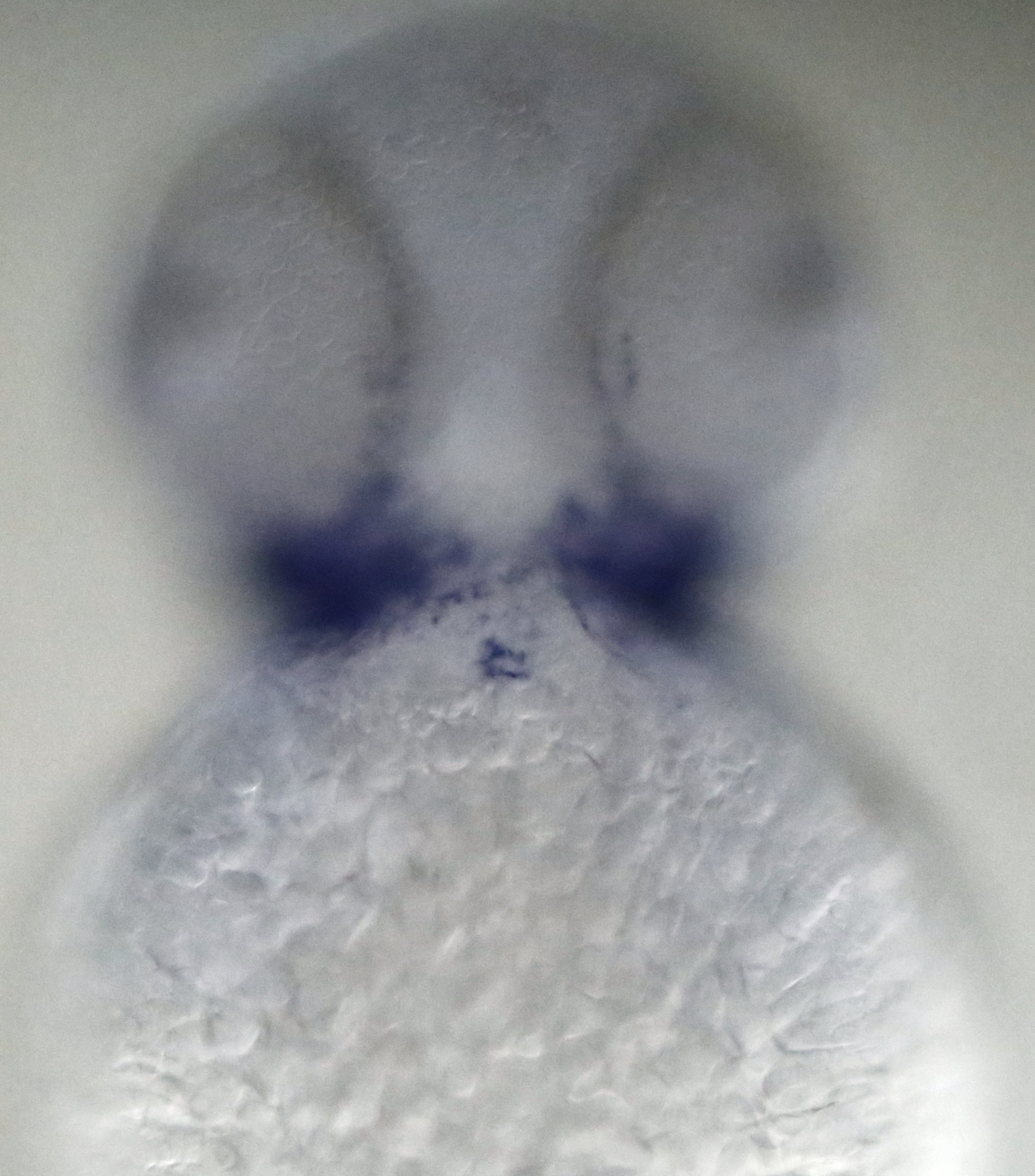

Supplement: Supplementary file 8 — Source data Fig. 3 [file 44321_2024_152_MOESM8_ESM.zip › Figure 3/3D/WtWnt9b.CR2 (RGB)-2200x2500.tif]

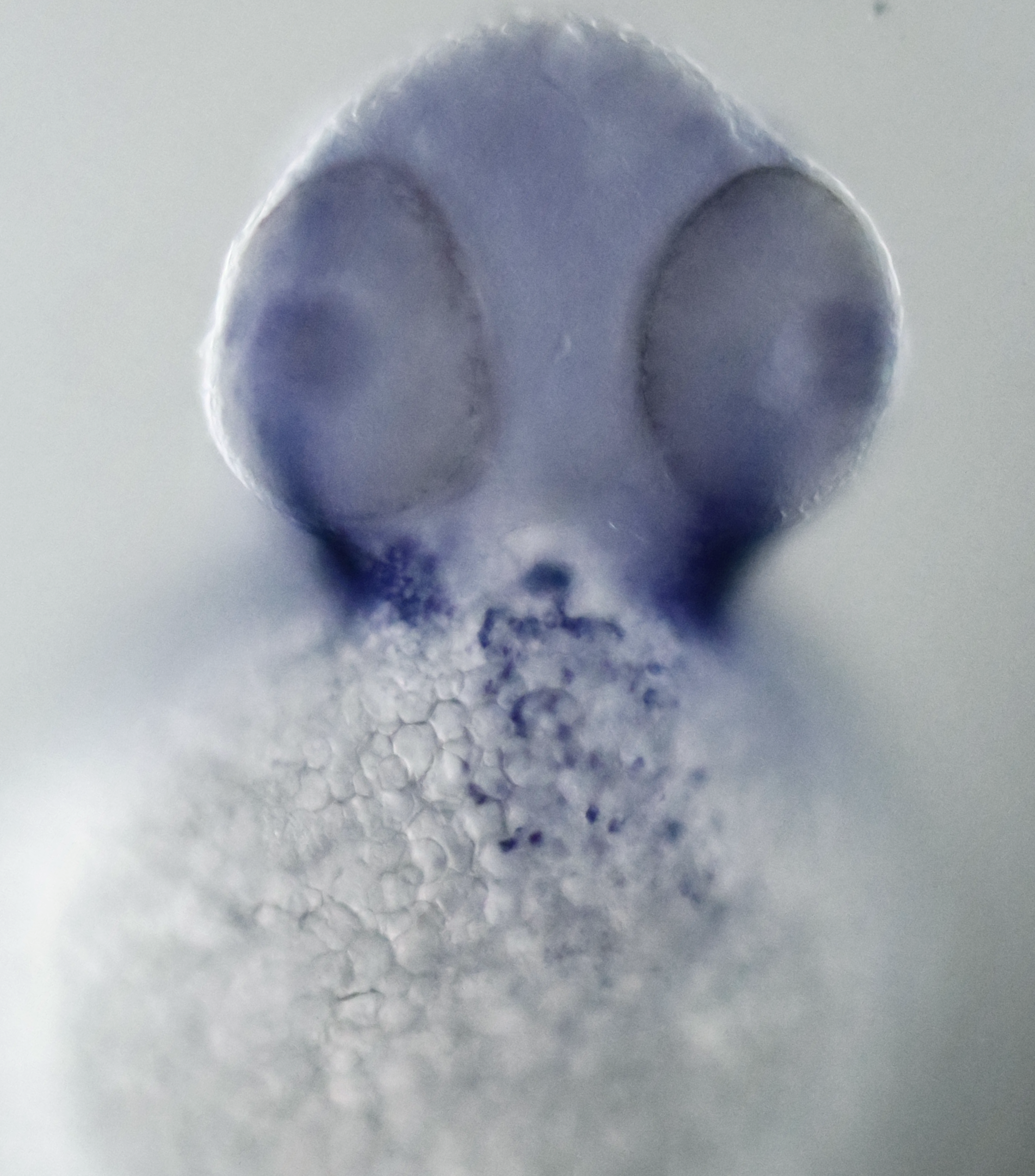

Supplement: Supplementary file 8 — Source data Fig. 3 [file 44321_2024_152_MOESM8_ESM.zip › Figure 3/3E/FliKlf2a_Wnt9b.CR2 (RGB)-2200x2500.tif]

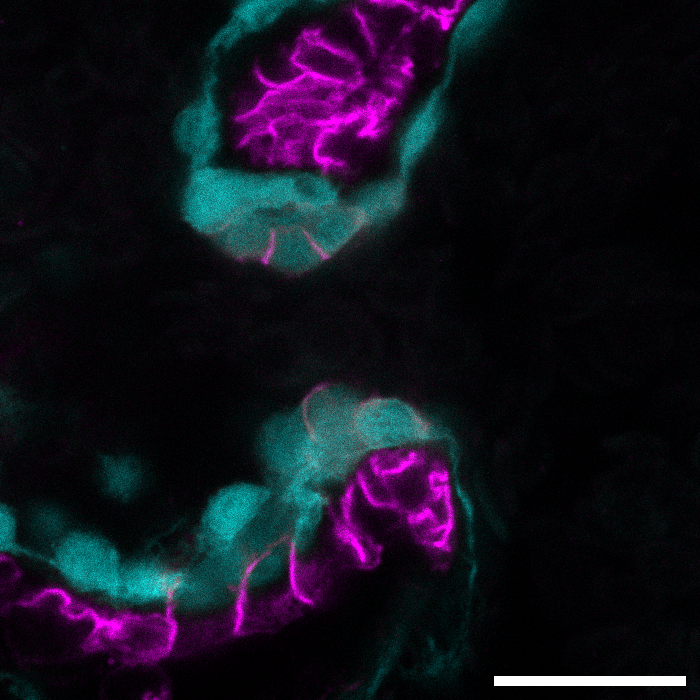

Supplement: Supplementary file 8 — Source data Fig. 3 [file 44321_2024_152_MOESM8_ESM.zip › Figure 3/3F/wt-stdMO-1-AVC-1.png]

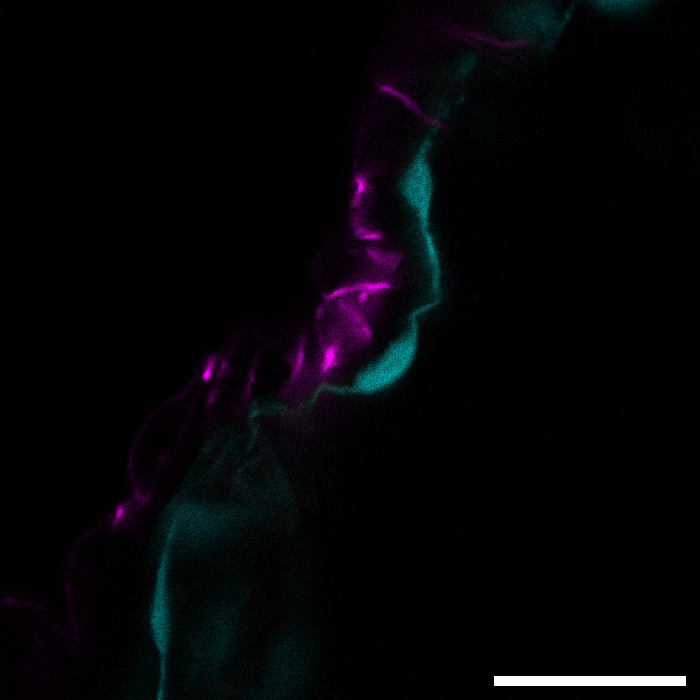

Supplement: Supplementary file 8 — Source data Fig. 3 [file 44321_2024_152_MOESM8_ESM.zip › Figure 3/3G/ccm2-stdMO-5-AVC-1.png]

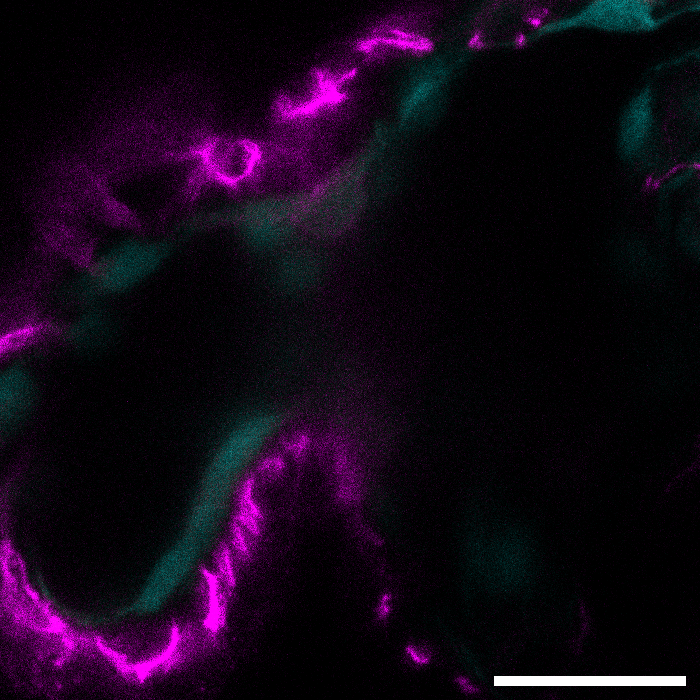

Supplement: Supplementary file 8 — Source data Fig. 3 [file 44321_2024_152_MOESM8_ESM.zip › Figure 3/3H/ccm2-wnt9bMO-9-1-AVC-scalebar.png]

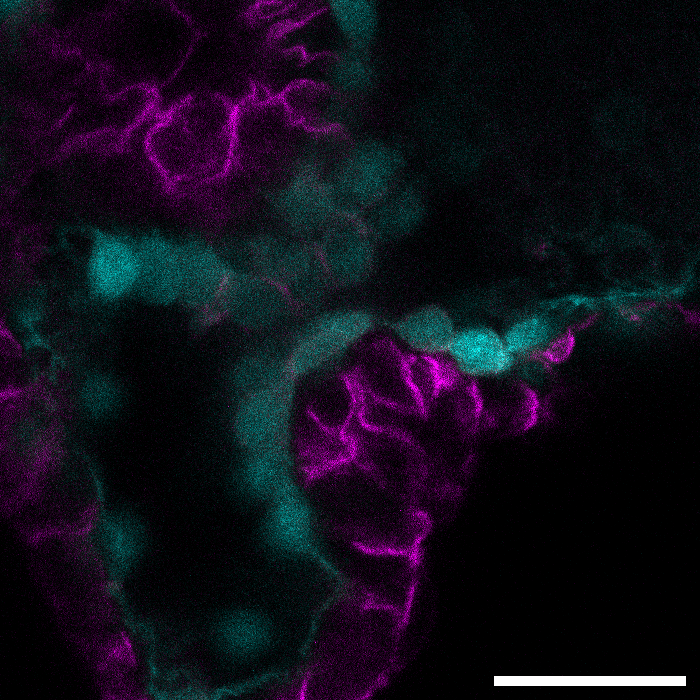

Supplement: Supplementary file 8 — Source data Fig. 3 [file 44321_2024_152_MOESM8_ESM.zip › Figure 3/3I/wt-wnt9bMO-4-AVC-scalebar.png]

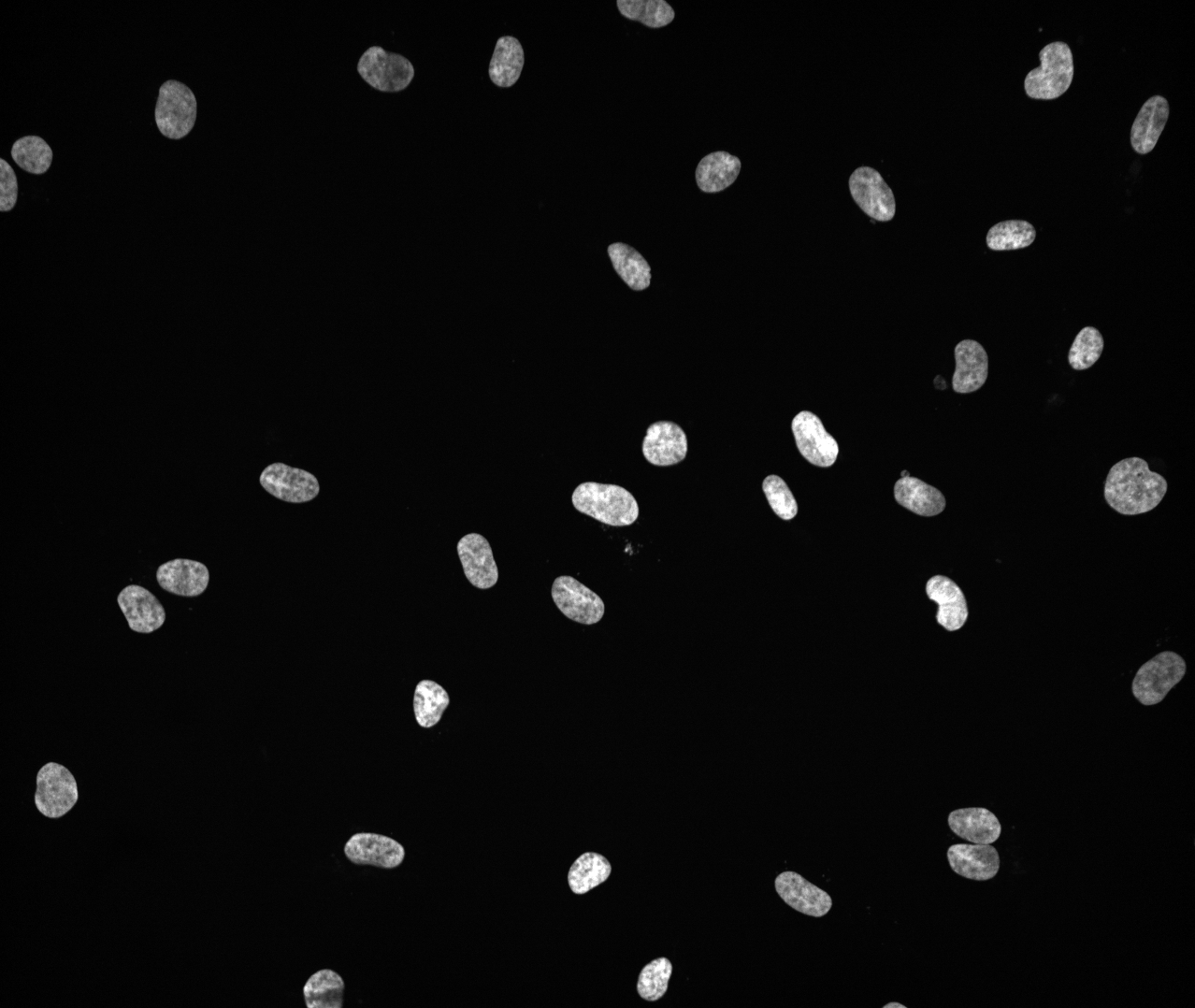

Supplement: Supplementary file 8 — Source data Fig. 3 [file 44321_2024_152_MOESM8_ESM.zip › Figure 3/3J/wt actin plt12 C3 F5.tif]

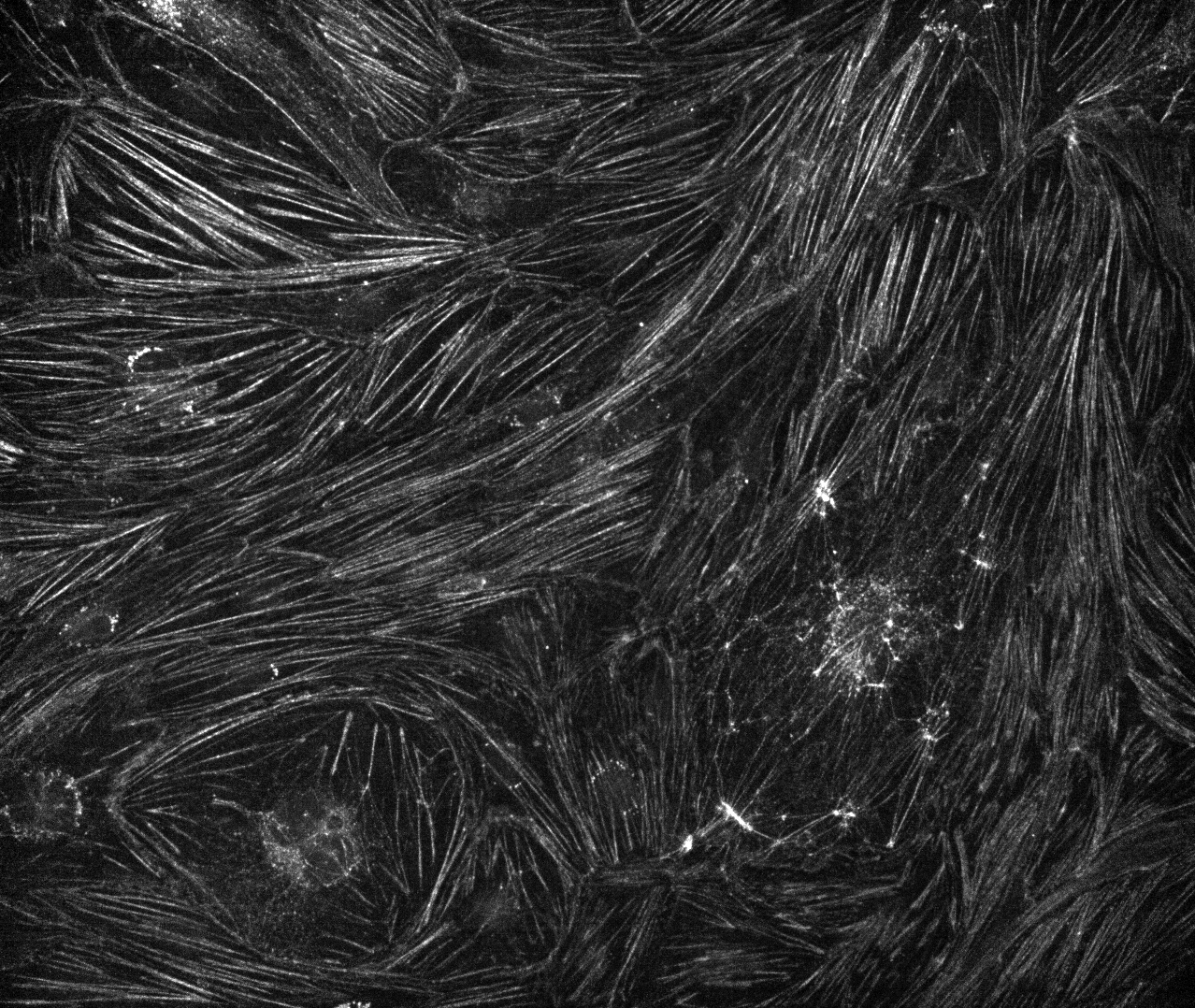

Supplement: Supplementary file 8 — Source data Fig. 3 [file 44321_2024_152_MOESM8_ESM.zip › Figure 3/3K/CCM2 ACTIN B3 F16 gray.tif]

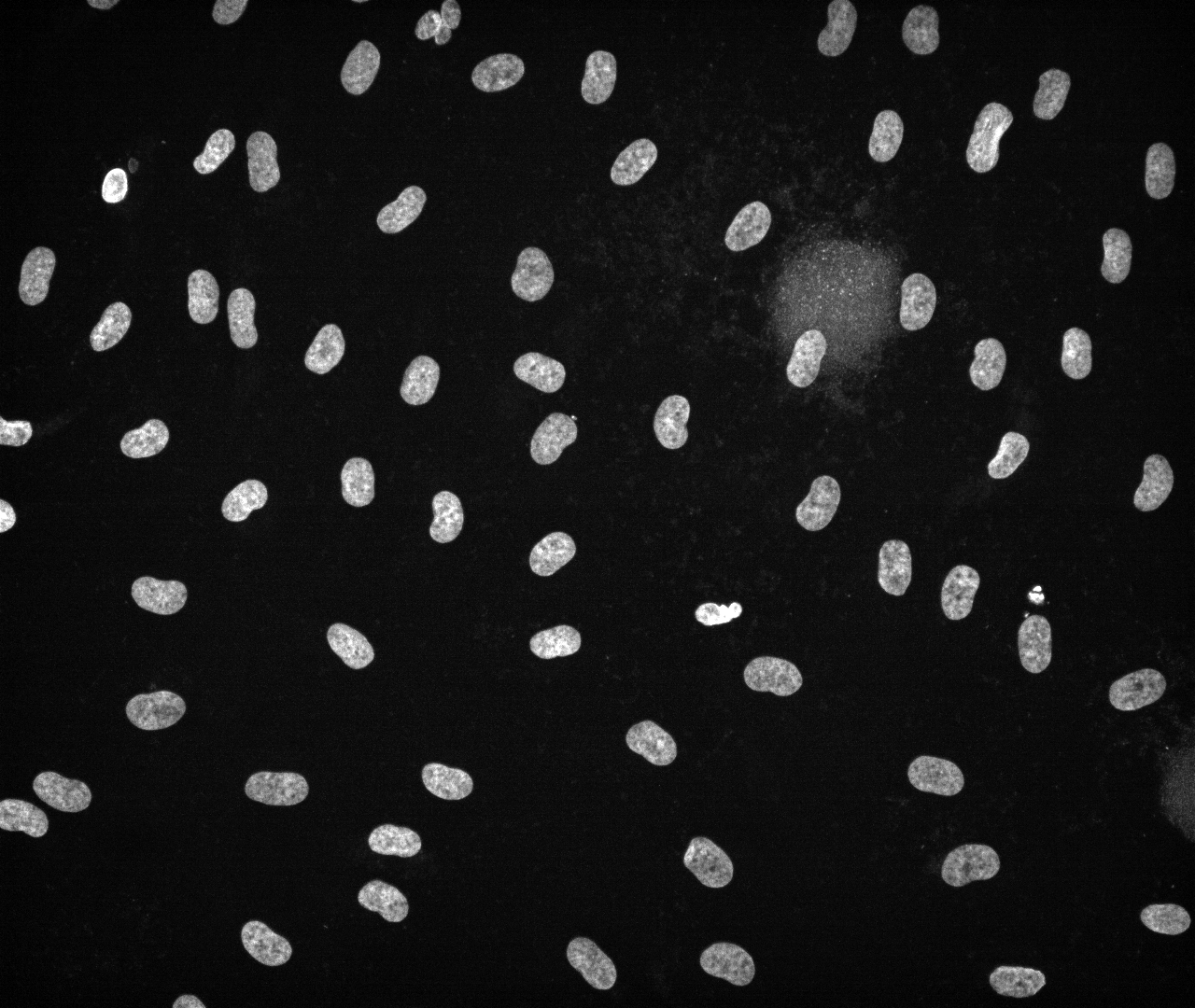

Supplement: Supplementary file 8 — Source data Fig. 3 [file 44321_2024_152_MOESM8_ESM.zip › Figure 3/3L/VeCad wt plt39 B4 F1.tif]

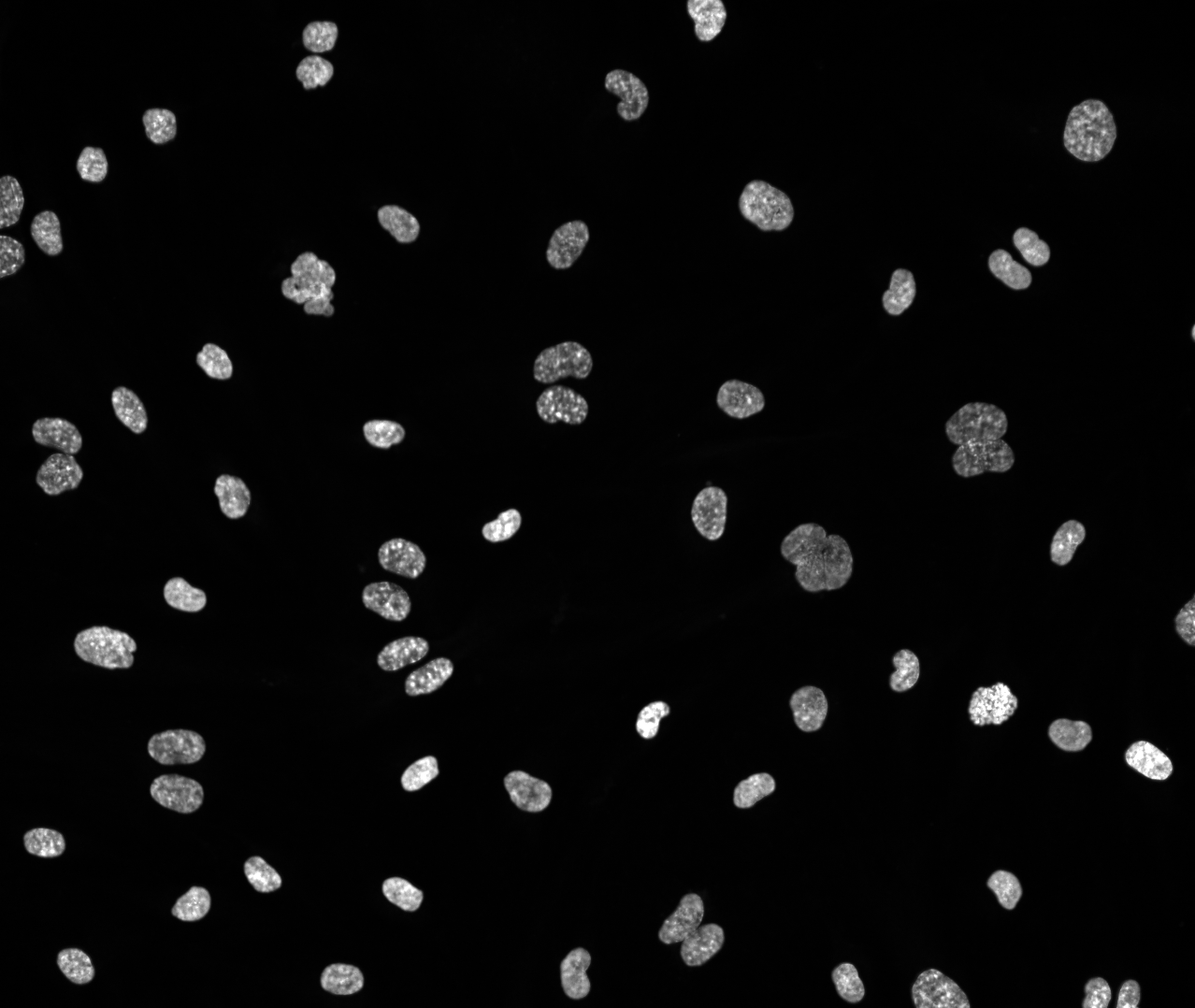

Supplement: Supplementary file 8 — Source data Fig. 3 [file 44321_2024_152_MOESM8_ESM.zip › Figure 3/3M/VeCad CCM2 plt17 E4 F6.tif]

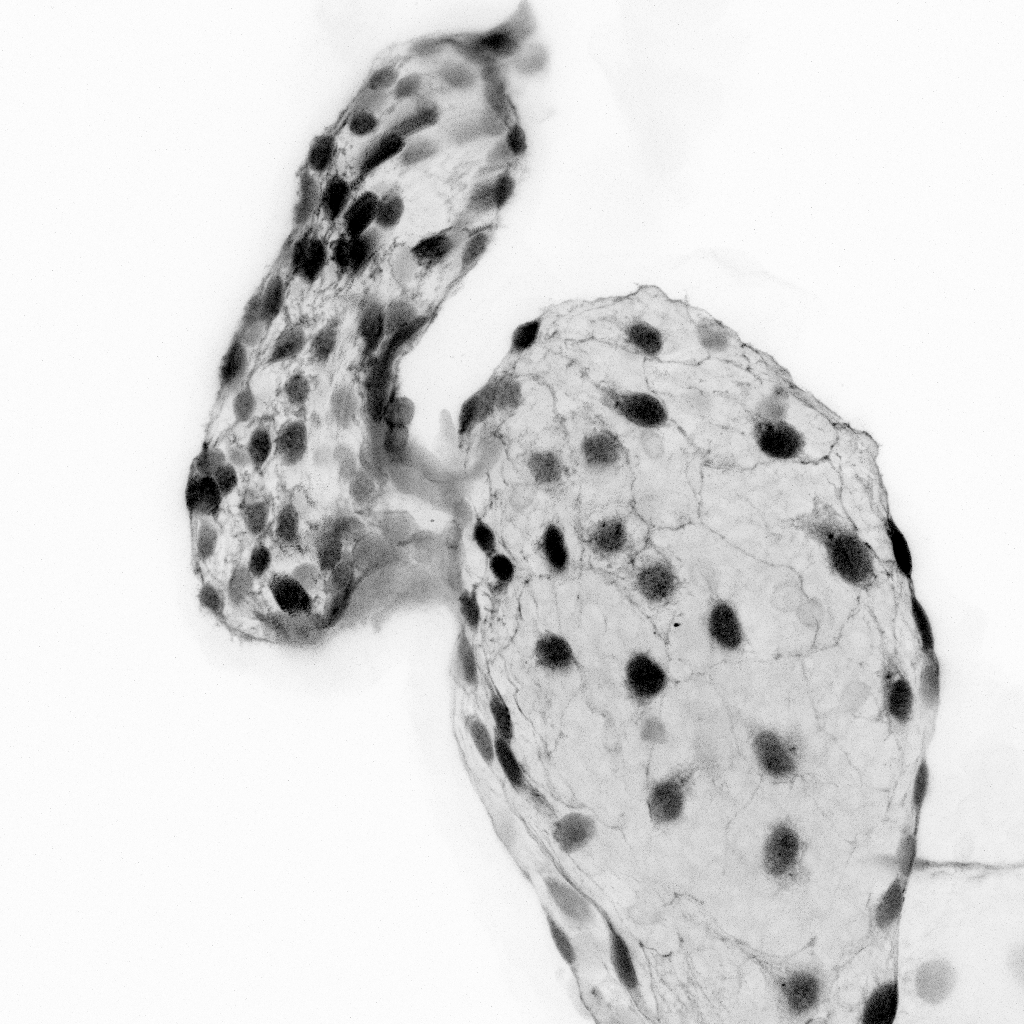

Supplement: Supplementary file 8 — Source data Fig. 3 [file 44321_2024_152_MOESM8_ESM.zip › Figure 3/3V/C1-extracted-wt_Maximum intensity projection.png]

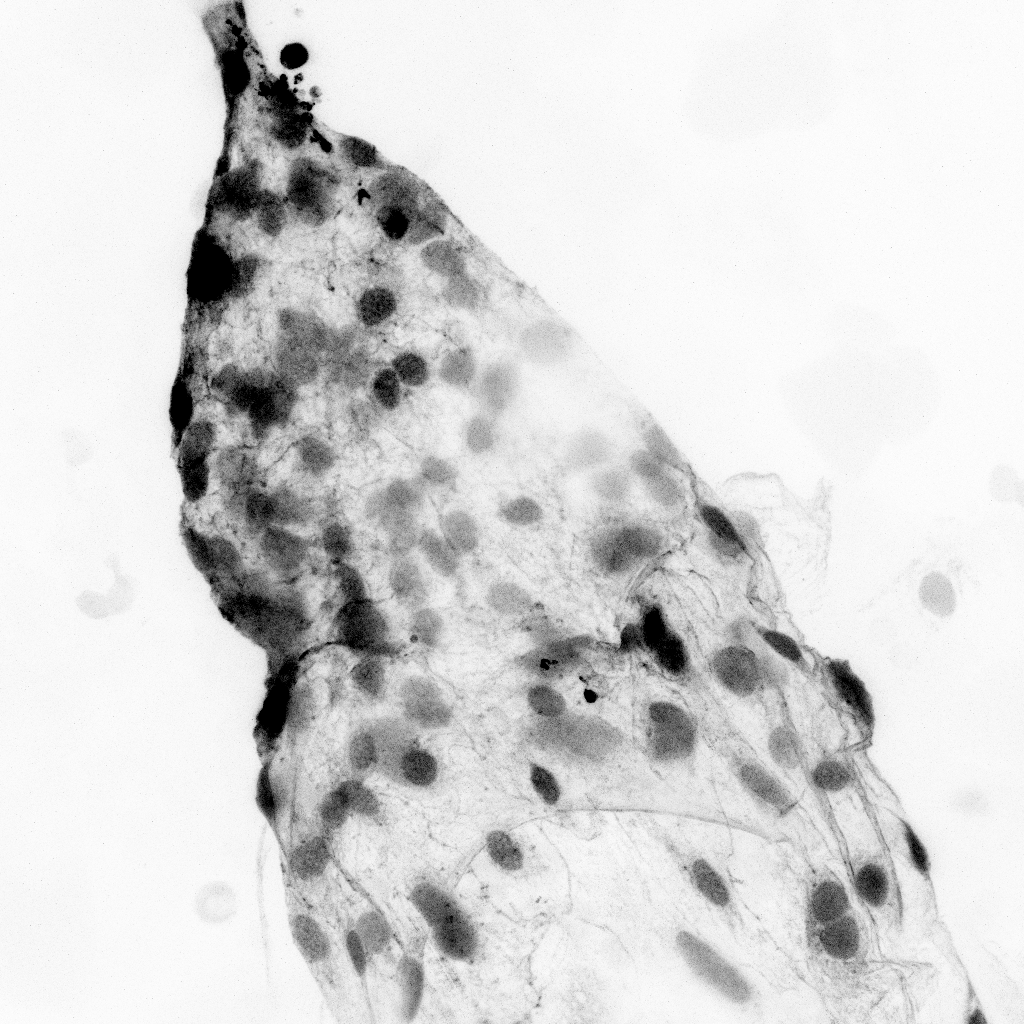

Supplement: Supplementary file 8 — Source data Fig. 3 [file 44321_2024_152_MOESM8_ESM.zip › Figure 3/3W/C1-MAX_extracted-krit1MO.png]

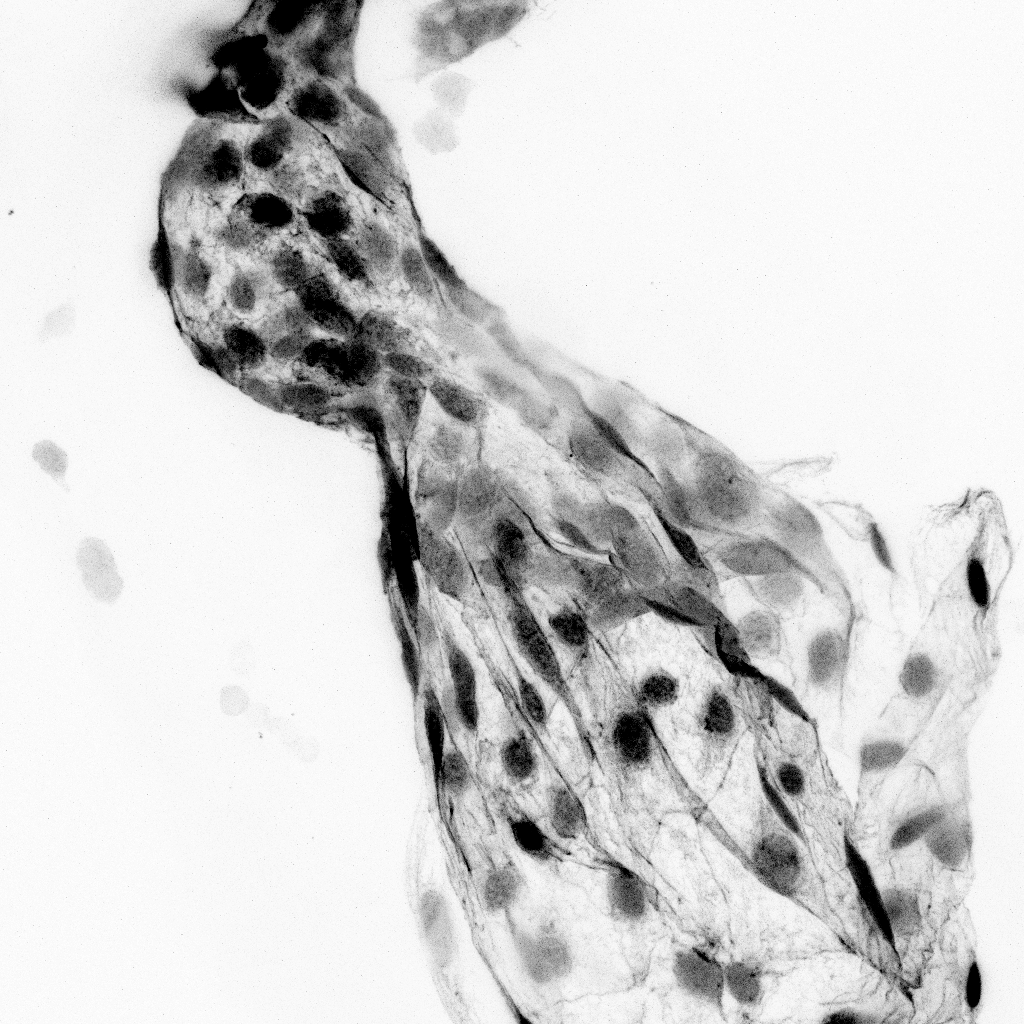

Supplement: Supplementary file 8 — Source data Fig. 3 [file 44321_2024_152_MOESM8_ESM.zip › Figure 3/3X/C1-MAX_extracted-rescued-alcam.png]

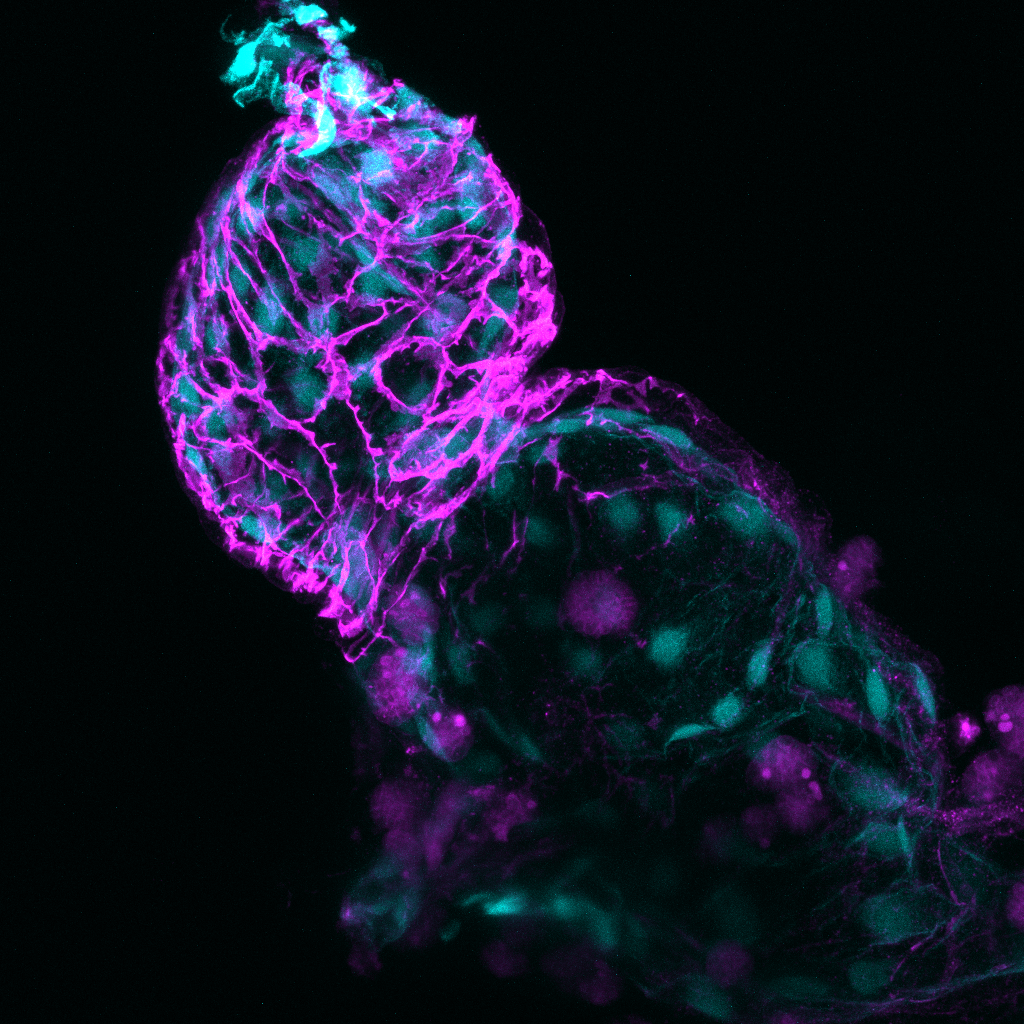

Supplement: Supplementary file 9 — Source data Fig. 4 [file 44321_2024_152_MOESM9_ESM.zip › Figure 4/4D/MAX_MS351-25uM-ccm2BF-1.tif (RGB).tif]

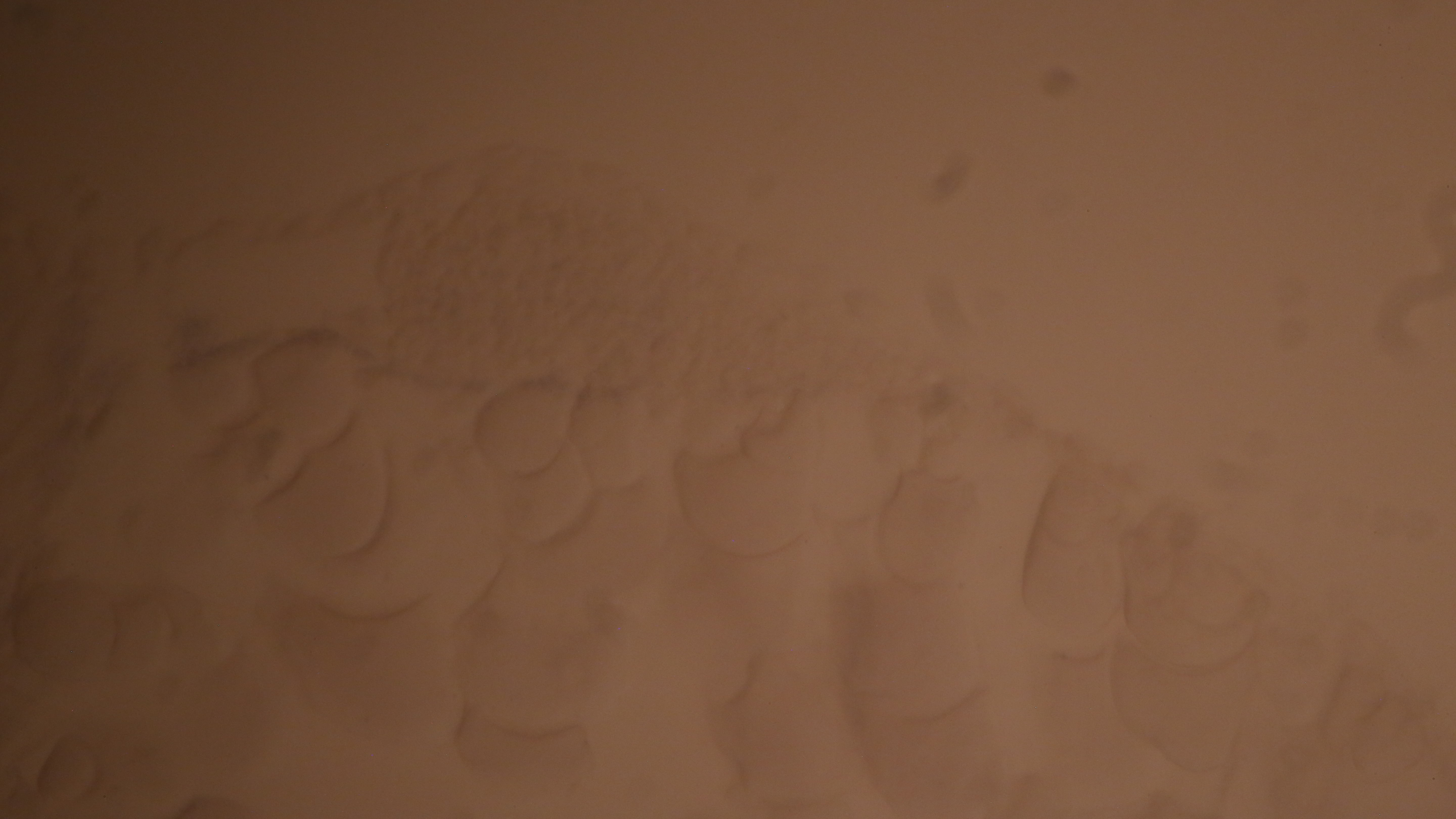

Supplement: Supplementary file 10 — Figure EV1 Source Data [file 44321_2024_152_MOESM10_ESM.zip › Figure EV1/A/wt_cbx7_s1__39.JPG]

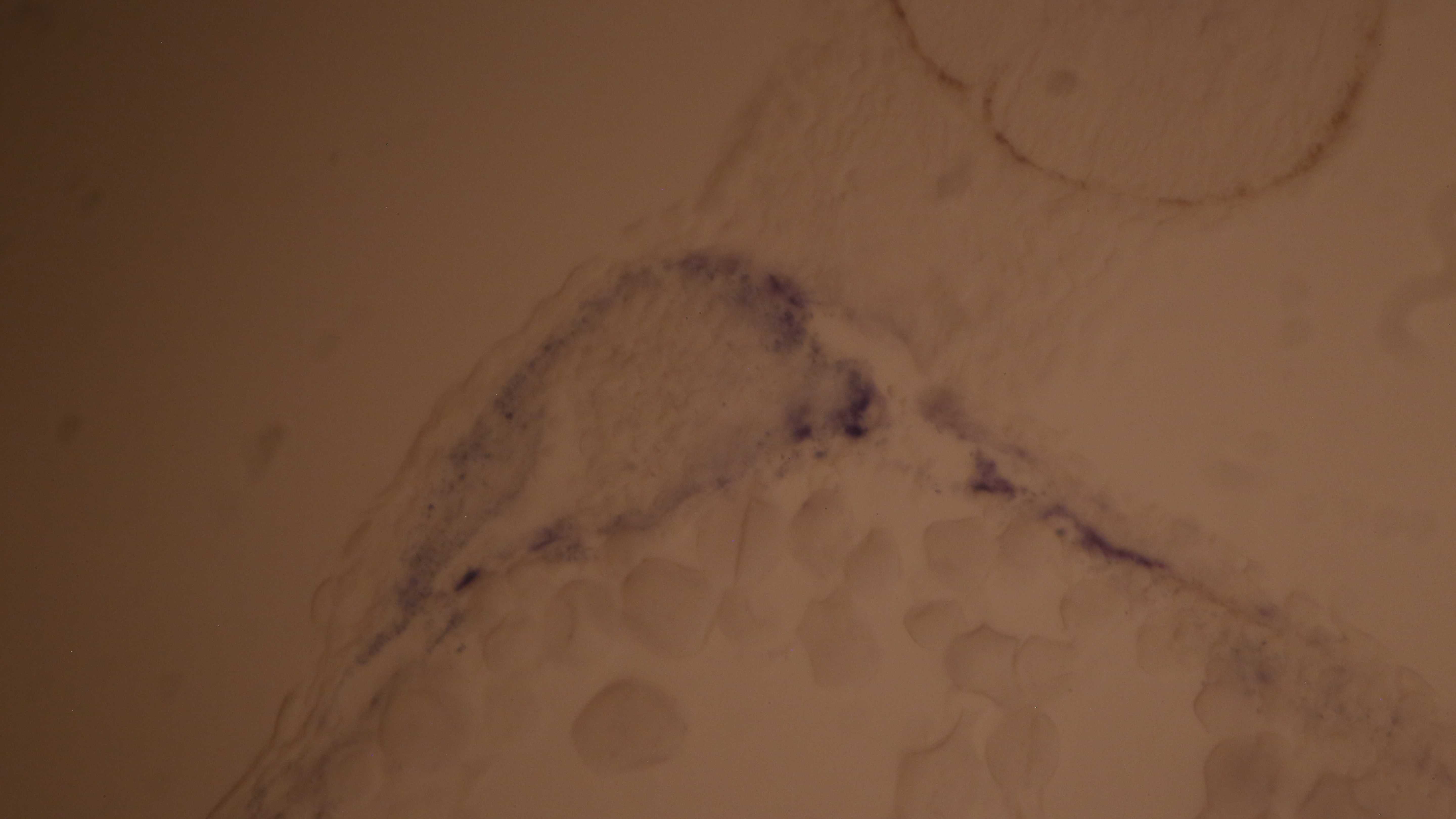

Supplement: Supplementary file 10 — Figure EV1 Source Data [file 44321_2024_152_MOESM10_ESM.zip › Figure EV1/B/ccm2_cbx7_s1__54.JPG]

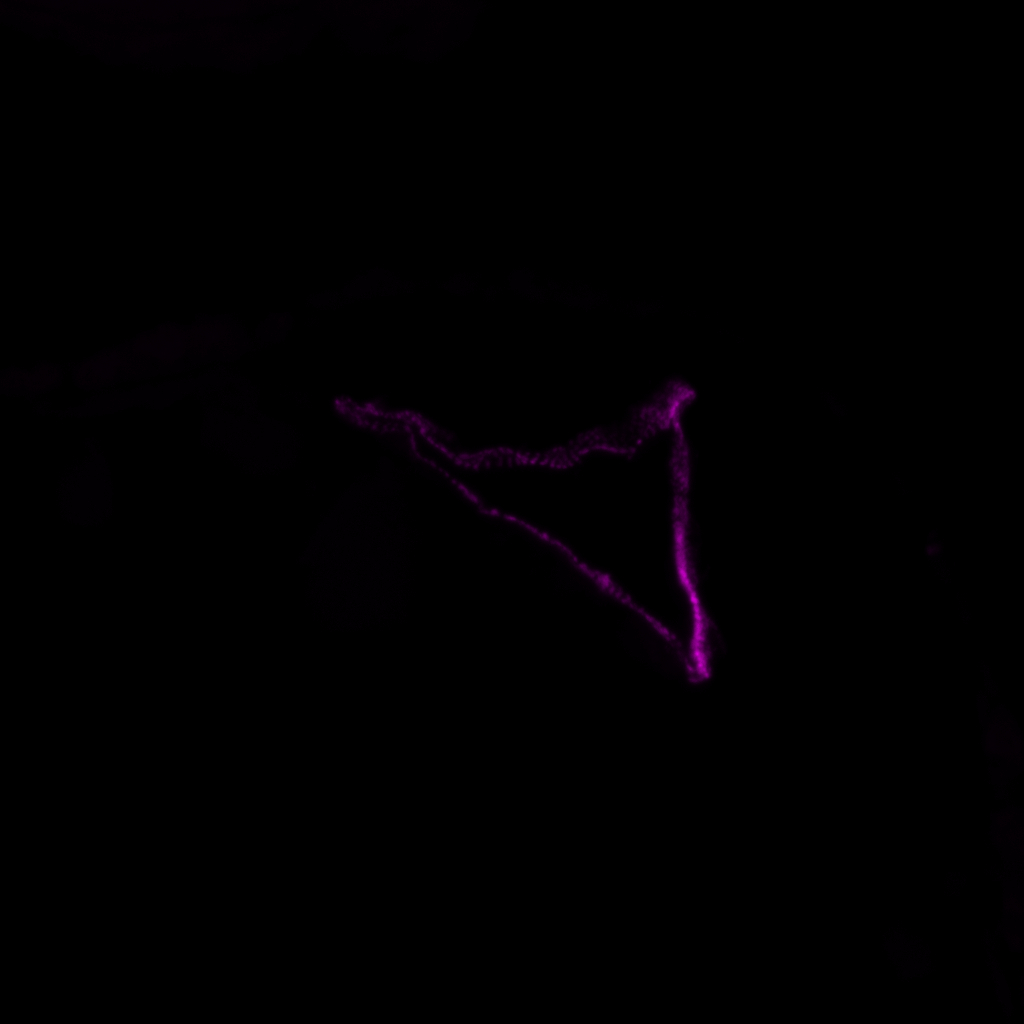

Supplement: Supplementary file 10 — Figure EV1 Source Data [file 44321_2024_152_MOESM10_ESM.zip › Figure EV1/C/AVG_wt_kdrl-GFP_cbx7-564_mf20-633_DAPI_s1_40x-_2024_06_19__09_49_33__p1.jpg]

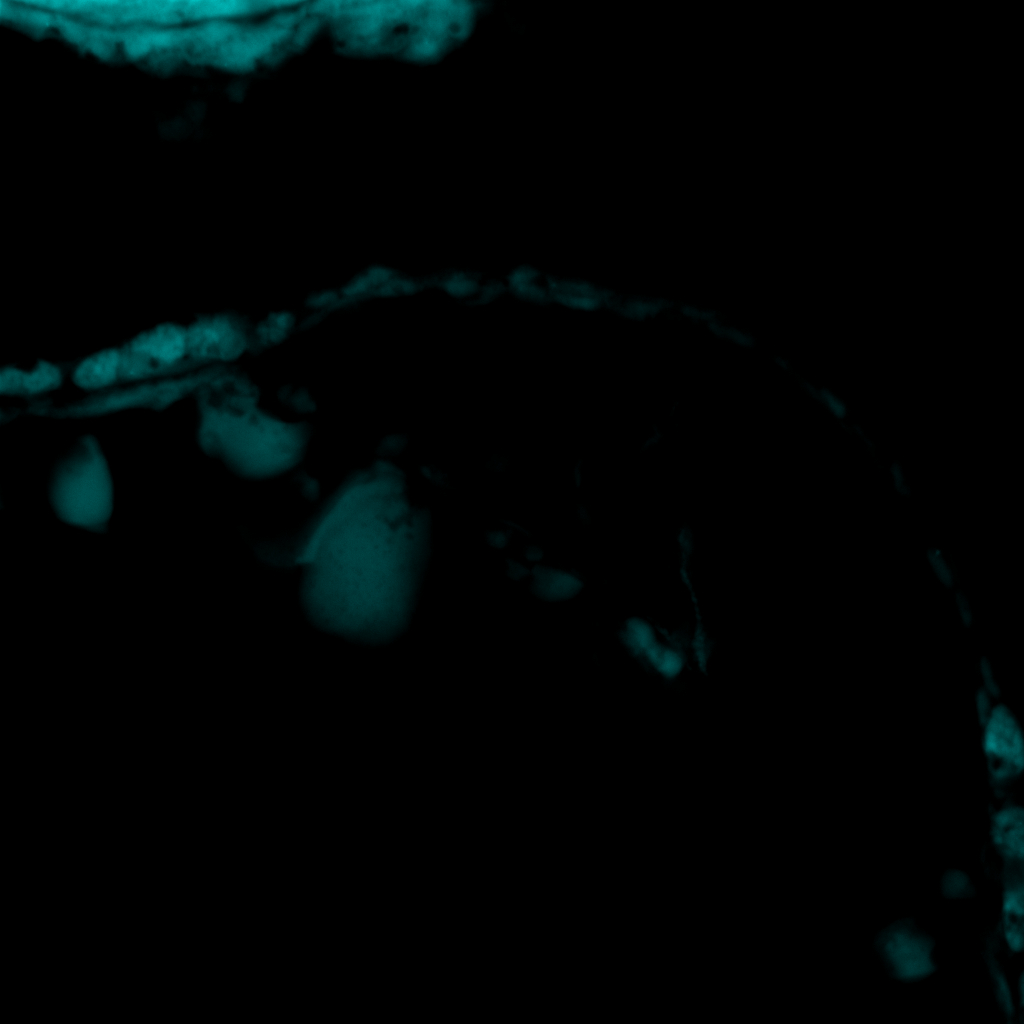

Supplement: Supplementary file 10 — Figure EV1 Source Data [file 44321_2024_152_MOESM10_ESM.zip › Figure EV1/C/AVG_wt_kdrl-GFP_cbx7-564_mf20-633_DAPI_s1_40x-_2024_06_19__09_49_33__p1c.jpg]

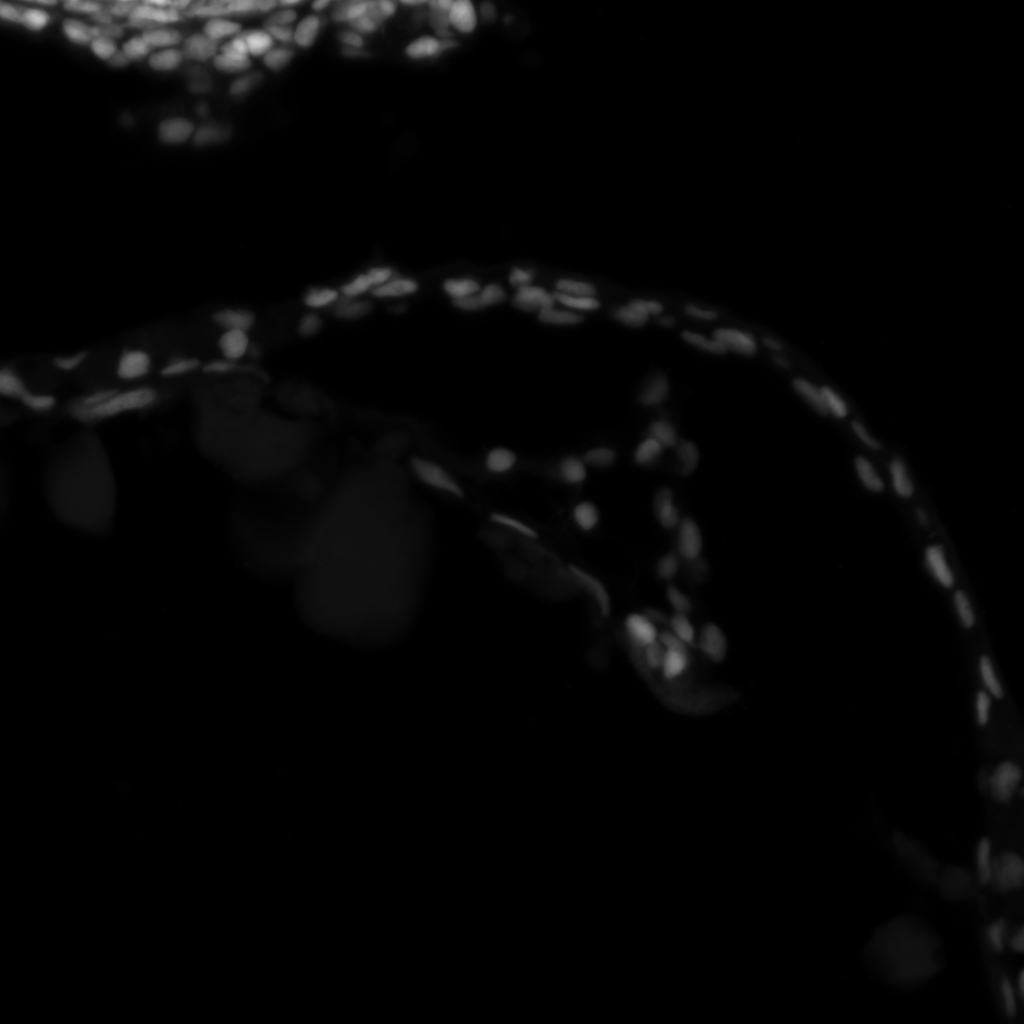

Supplement: Supplementary file 10 — Figure EV1 Source Data [file 44321_2024_152_MOESM10_ESM.zip › Figure EV1/C/AVG_wt_kdrl-GFP_cbx7-564_mf20-633_DAPI_s1_40x-_2024_06_19__09_49_33__p1d.jpg]

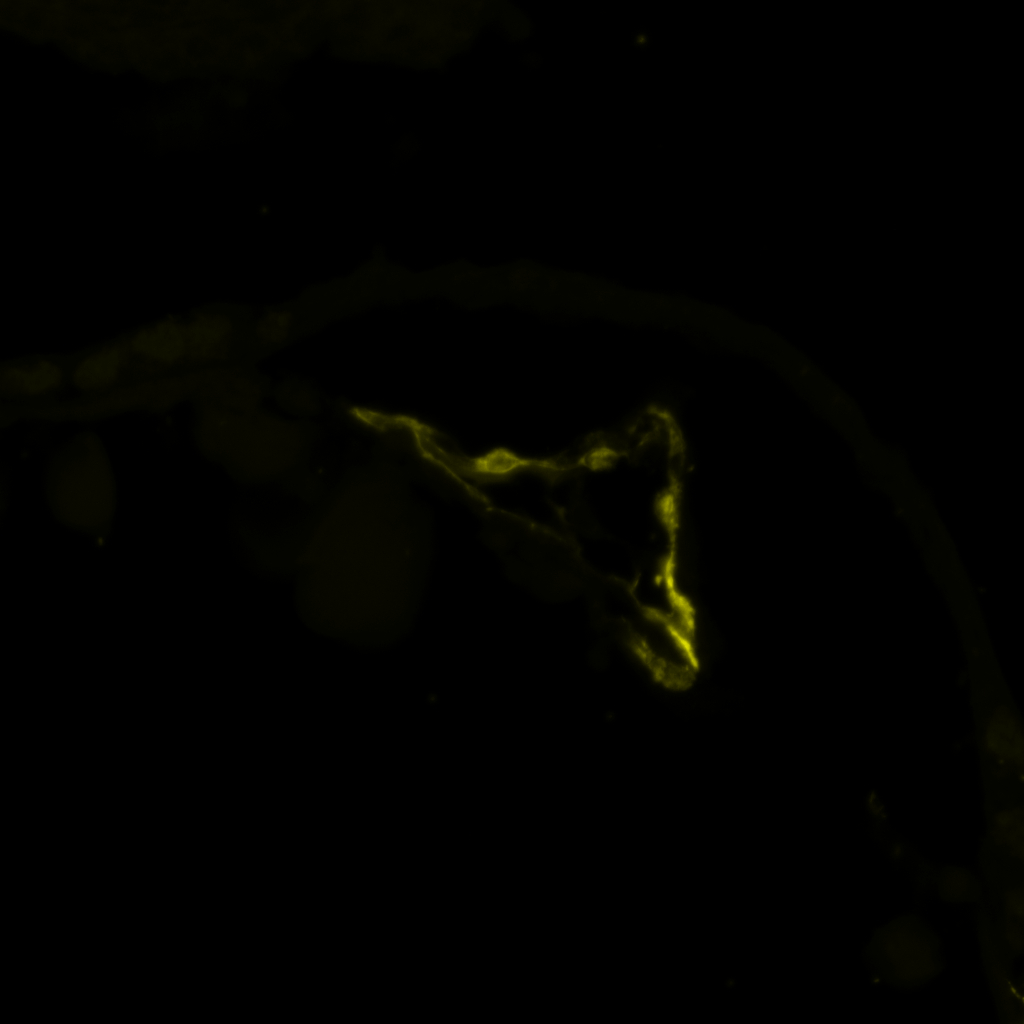

Supplement: Supplementary file 10 — Figure EV1 Source Data [file 44321_2024_152_MOESM10_ESM.zip › Figure EV1/C/AVG_wt_kdrl-GFP_cbx7-564_mf20-633_DAPI_s1_40x-_2024_06_19__09_49_33__p1g.jpg]

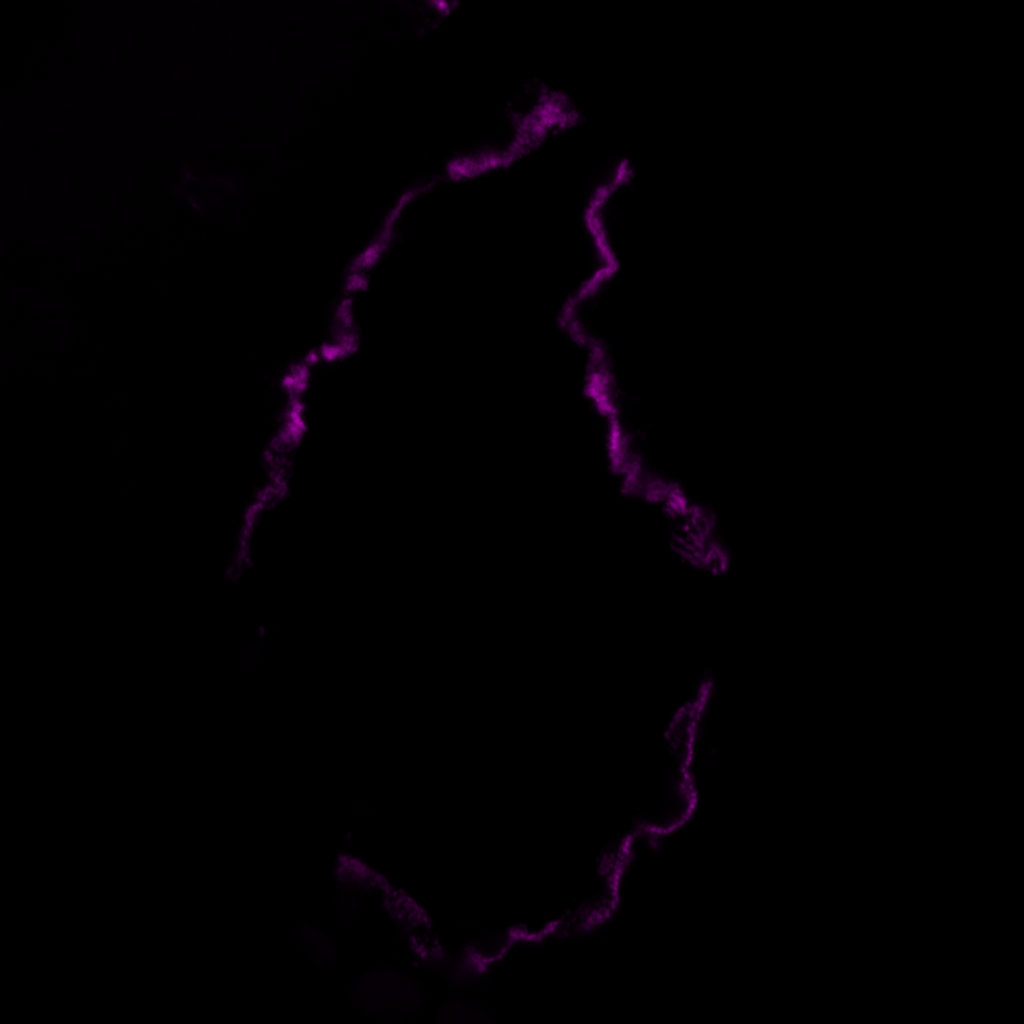

Supplement: Supplementary file 10 — Figure EV1 Source Data [file 44321_2024_152_MOESM10_ESM.zip › Figure EV1/D/AVG_ccm2_kdrl-GFP_cbx7-564_mf20-633_DAPI_s2_40x_8.jpg]

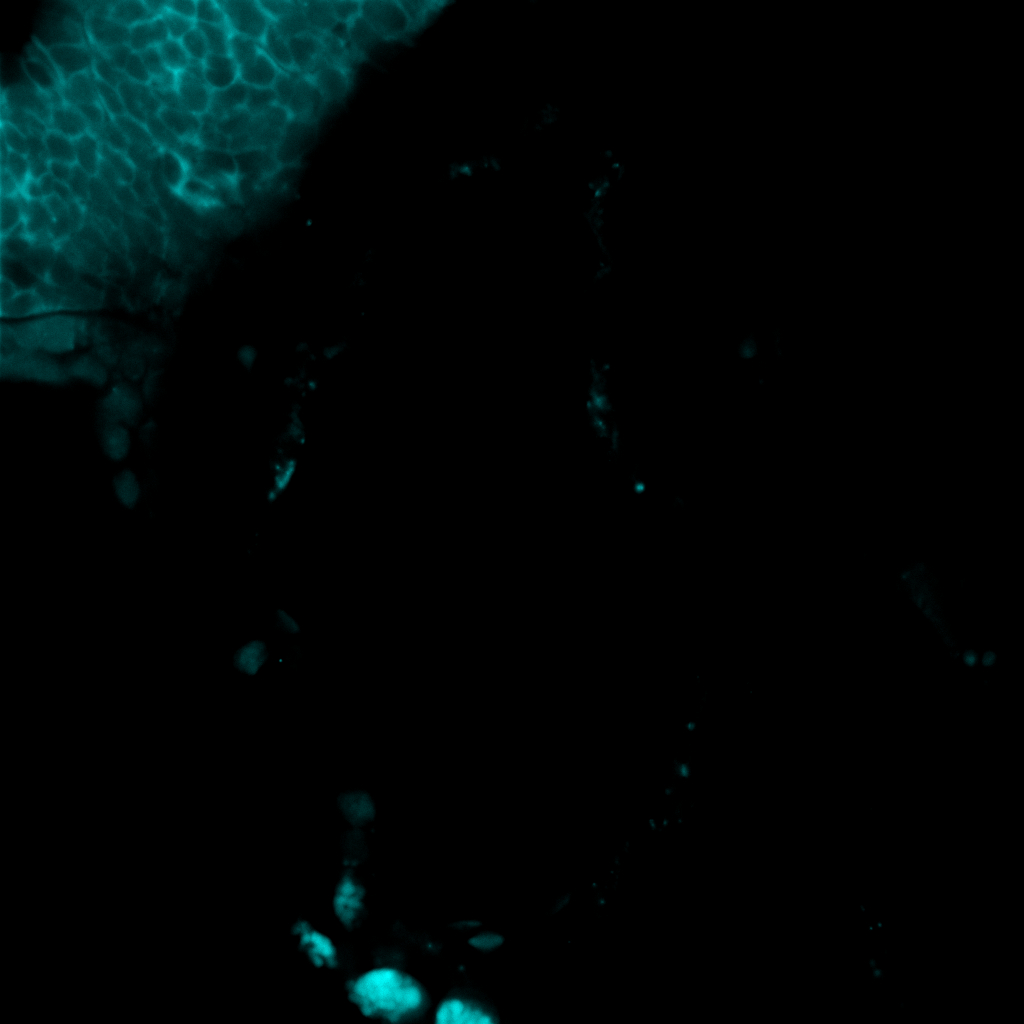

Supplement: Supplementary file 10 — Figure EV1 Source Data [file 44321_2024_152_MOESM10_ESM.zip › Figure EV1/D/AVG_ccm2_kdrl-GFP_cbx7-564_mf20-633_DAPI_s2_40x_8c.jpg]

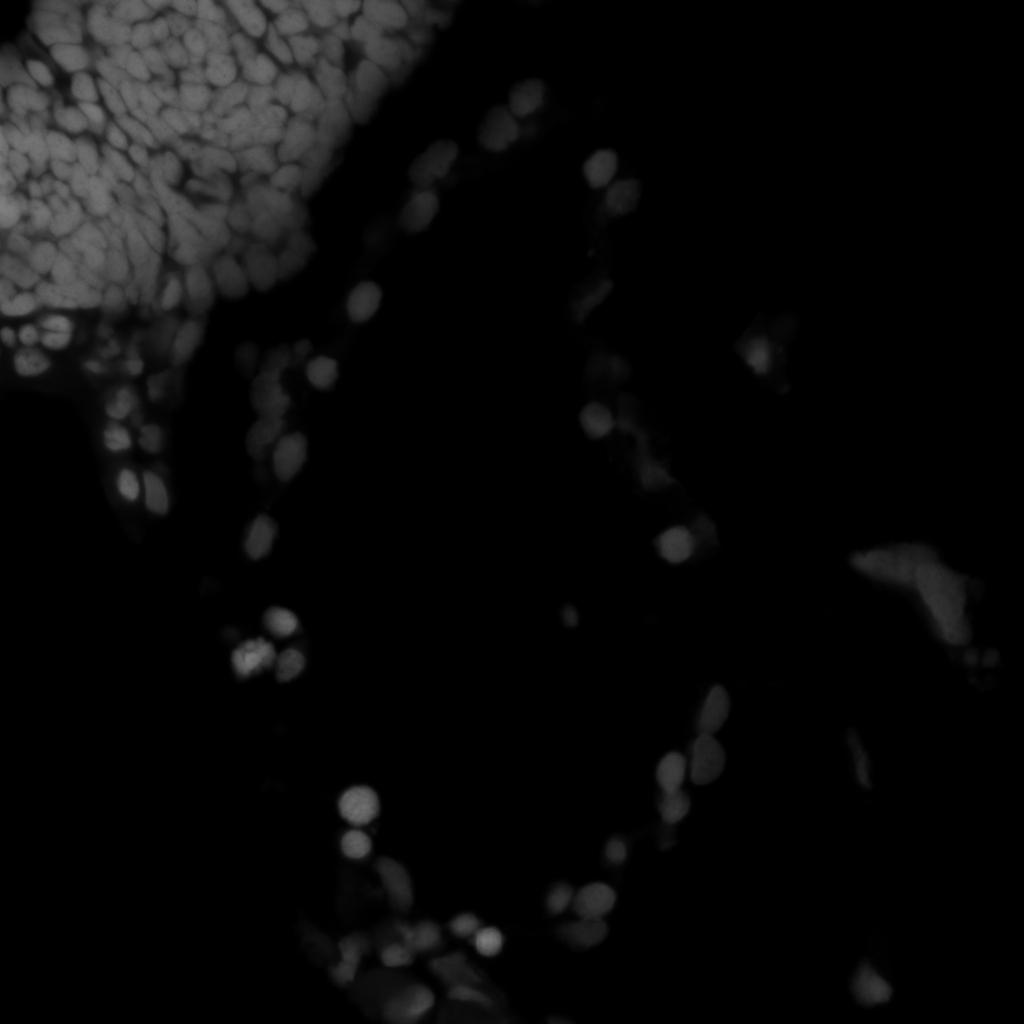

Supplement: Supplementary file 10 — Figure EV1 Source Data [file 44321_2024_152_MOESM10_ESM.zip › Figure EV1/D/AVG_ccm2_kdrl-GFP_cbx7-564_mf20-633_DAPI_s2_40x_8d.jpg]

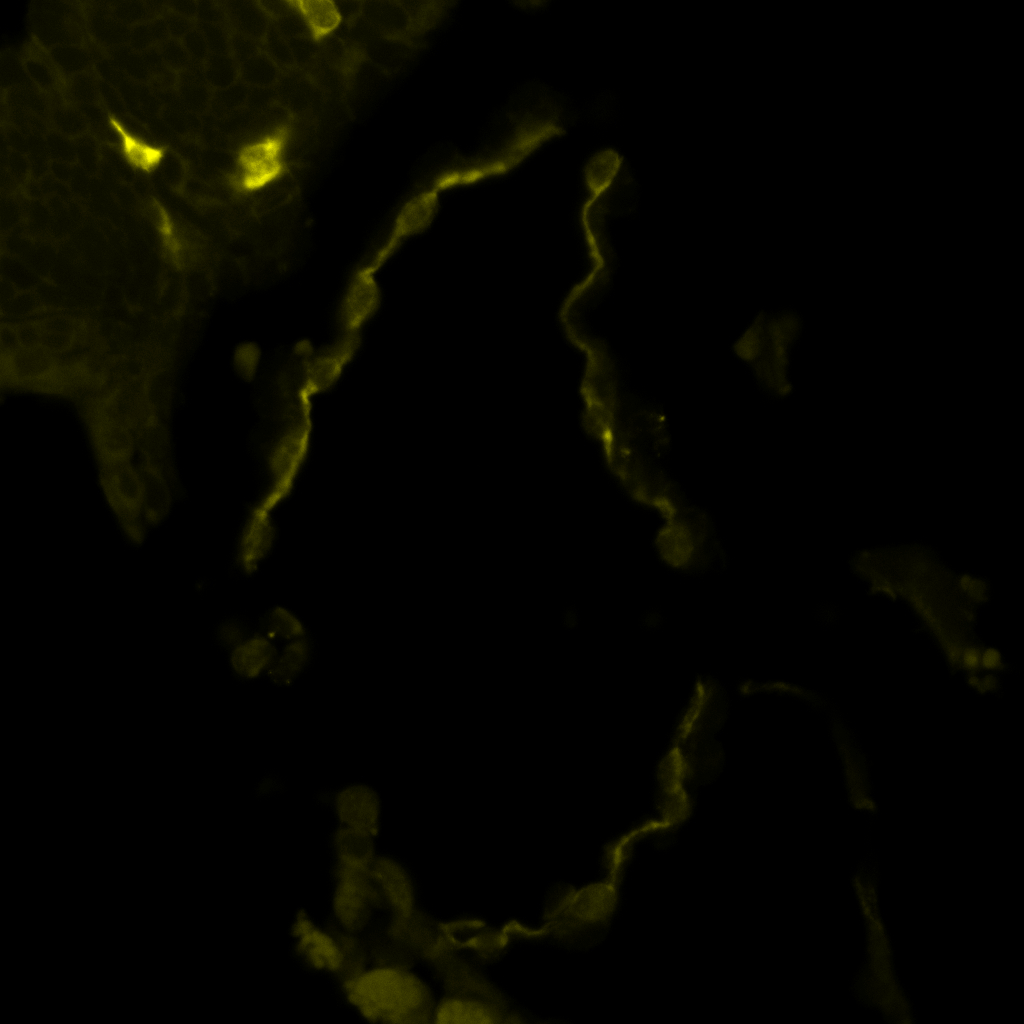

Supplement: Supplementary file 10 — Figure EV1 Source Data [file 44321_2024_152_MOESM10_ESM.zip › Figure EV1/D/AVG_ccm2_kdrl-GFP_cbx7-564_mf20-633_DAPI_s2_40x_8g.jpg]

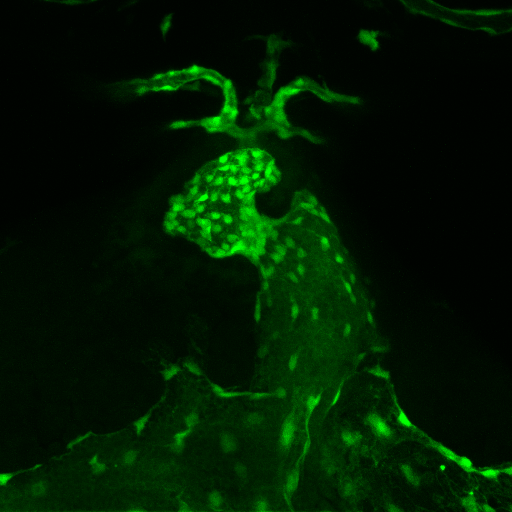

Supplement: Supplementary file 11 — Figure EV2 Source Data [file 44321_2024_152_MOESM11_ESM.zip › Figure EV2/B/MAX_wt-heat-2.lsm (RGB).tif]

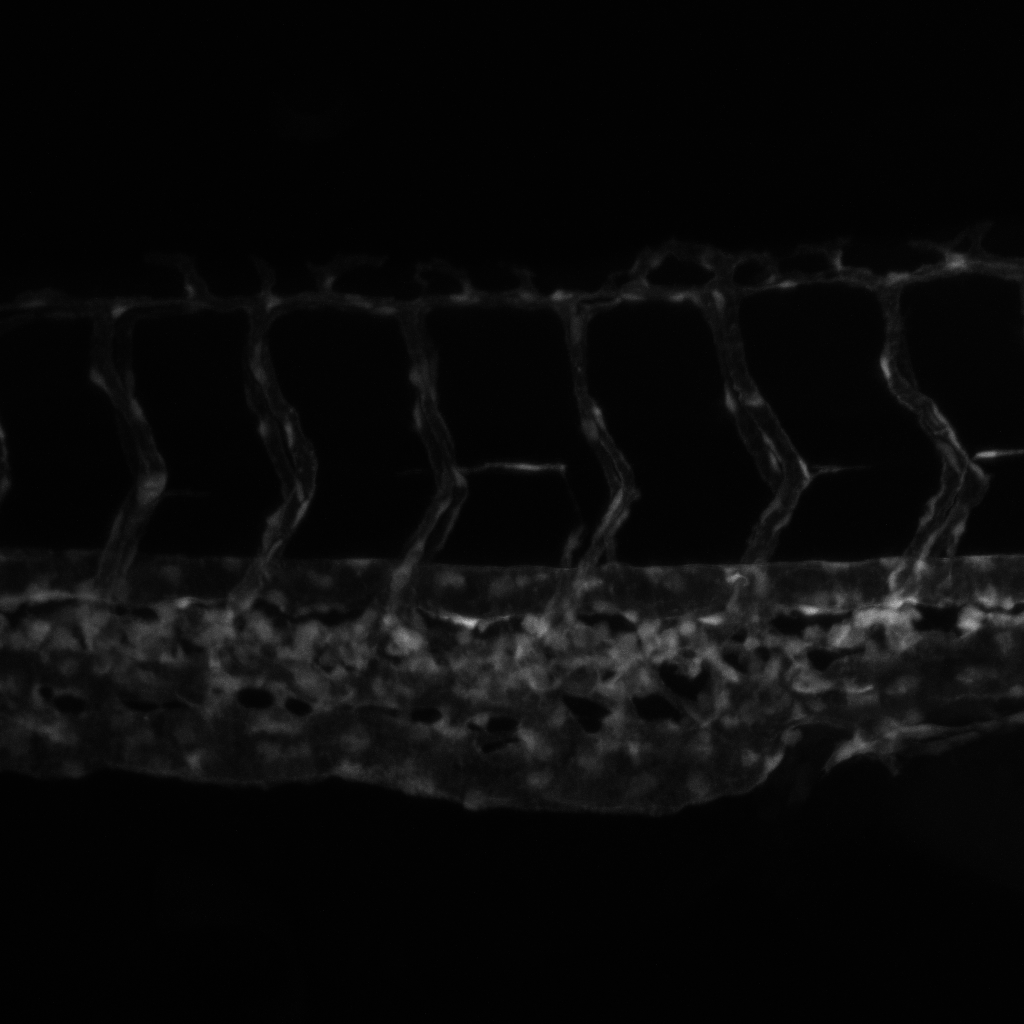

Supplement: Supplementary file 11 — Figure EV2 Source Data [file 44321_2024_152_MOESM11_ESM.zip › Figure EV2/D/MAX_wt-tail.tif]

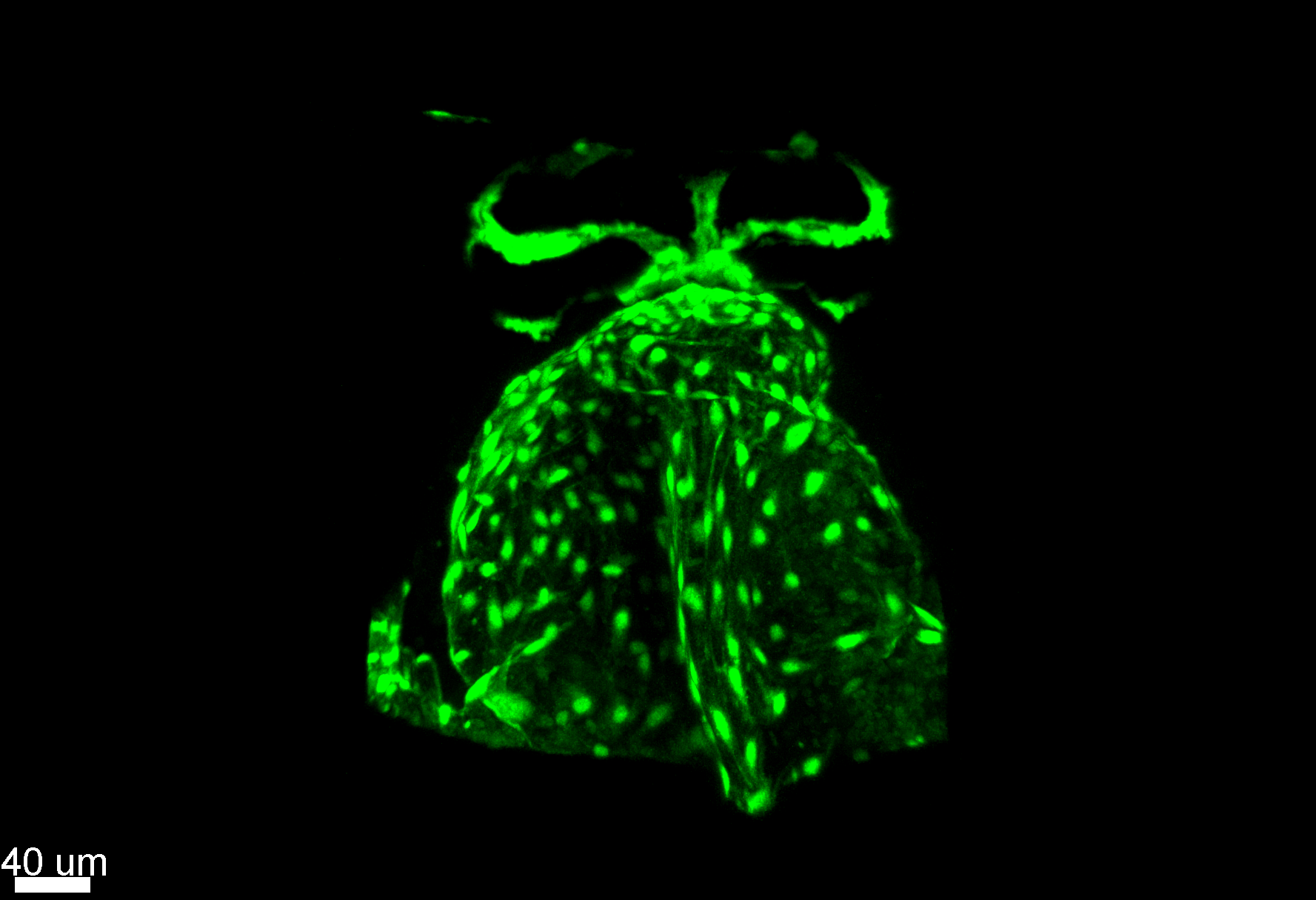

Supplement: Supplementary file 11 — Figure EV2 Source Data [file 44321_2024_152_MOESM11_ESM.zip › Figure EV2/E/ccm2-heart-5_2024-08-13T14-32-43.838.tif]

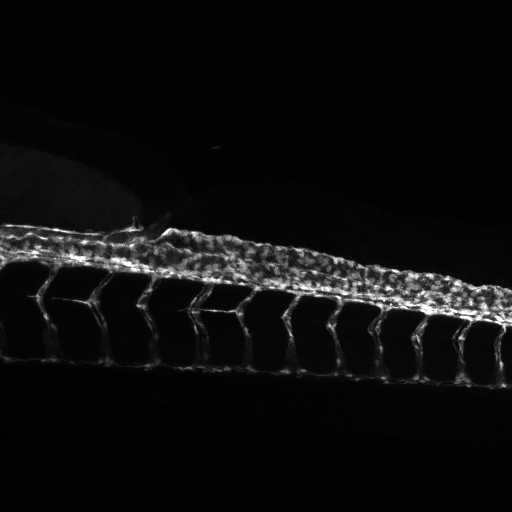

Supplement: Supplementary file 11 — Figure EV2 Source Data [file 44321_2024_152_MOESM11_ESM.zip › Figure EV2/G/MAX_B5591inx-ccm2hom-tail-14-8.11.tif]

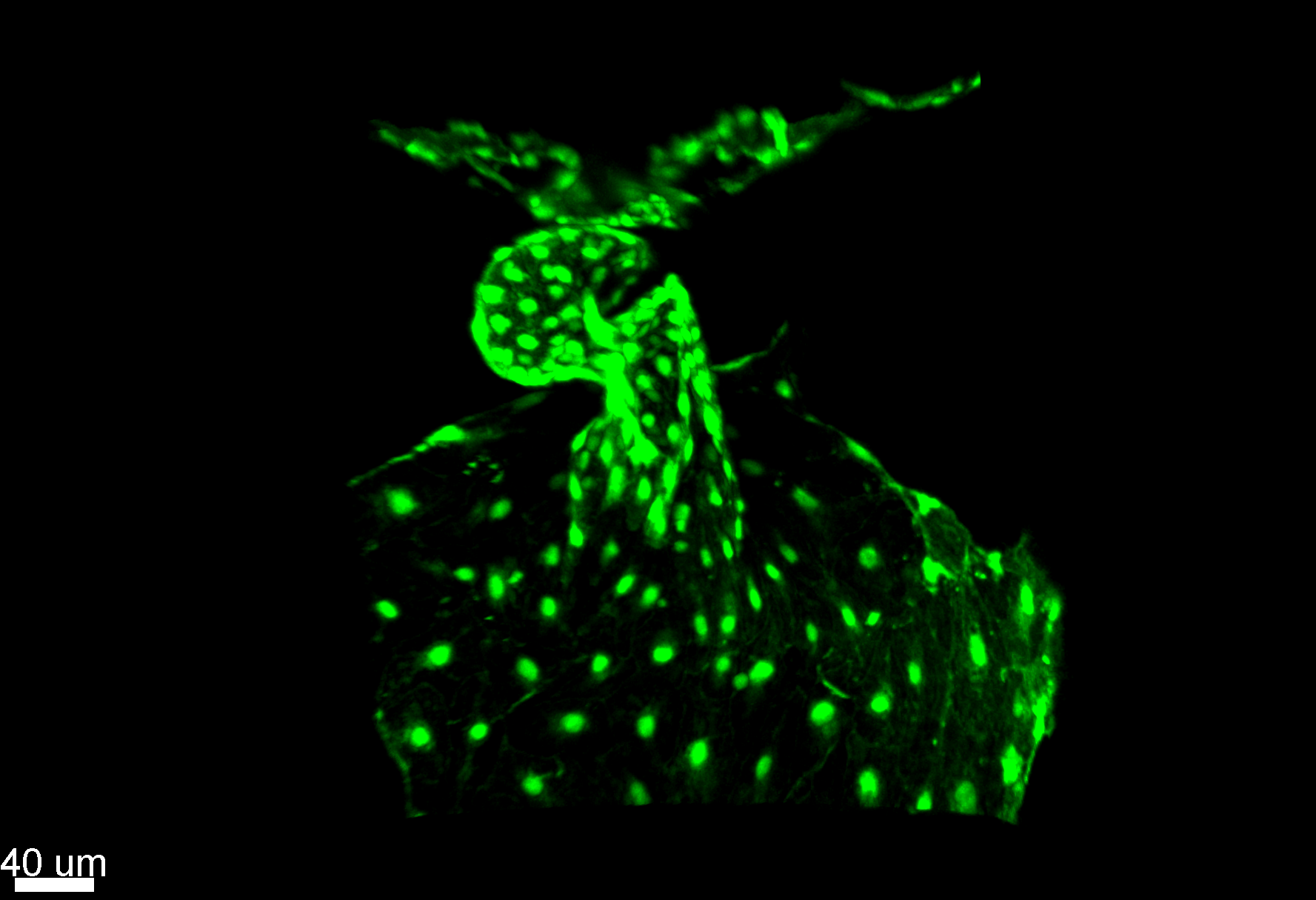

Supplement: Supplementary file 11 — Figure EV2 Source Data [file 44321_2024_152_MOESM11_ESM.zip › Figure EV2/H/ccm2hom-pbb62----BF2-heart_2024-08-13T14-33-41.981.tif]

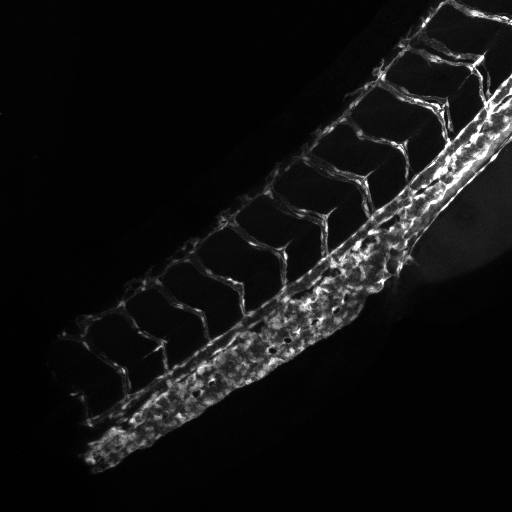

Supplement: Supplementary file 11 — Figure EV2 Source Data [file 44321_2024_152_MOESM11_ESM.zip › Figure EV2/J/MAX_pbb62hom-ccm2hom-tail-wBF-3-8.11.tif]

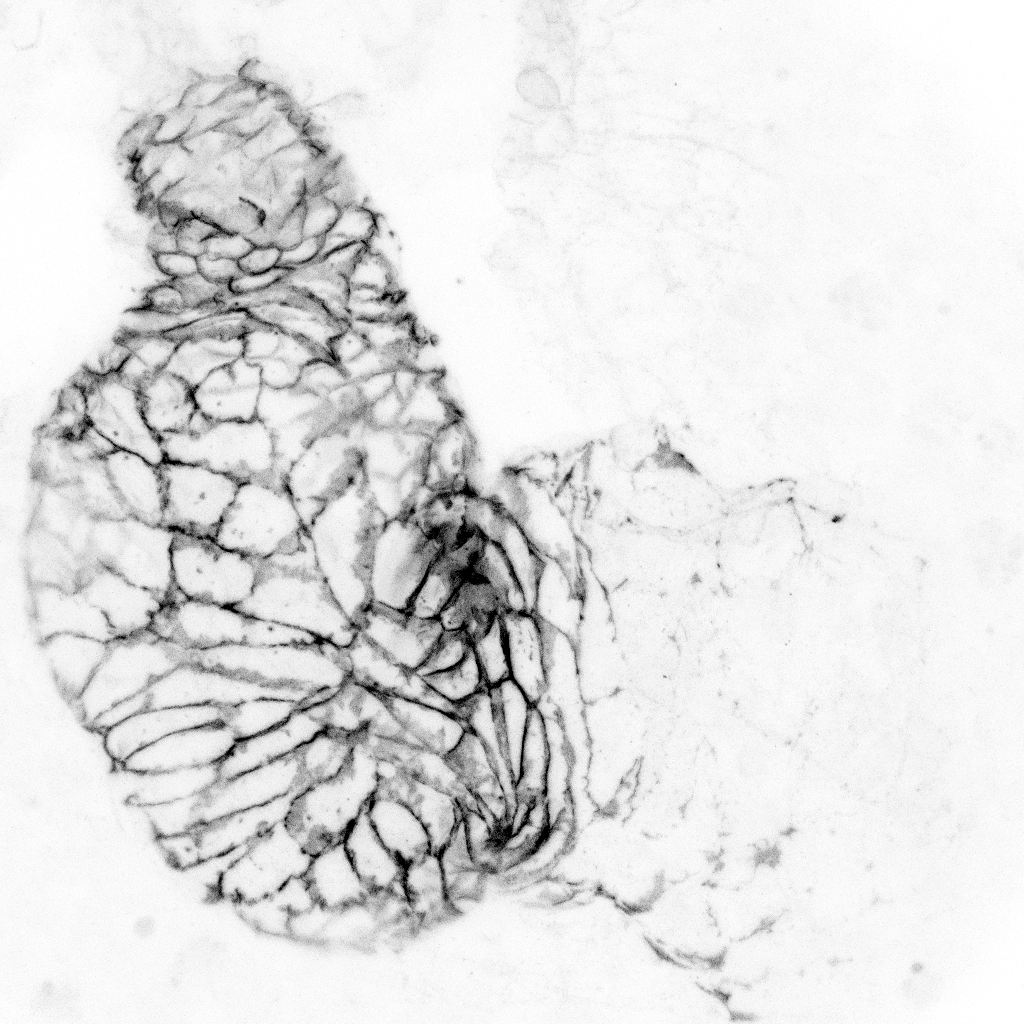

Supplement: Supplementary file 11 — Figure EV2 Source Data [file 44321_2024_152_MOESM11_ESM.zip › Figure EV2/N/MAX_52_stdMO_krit1sib_cbx7aMO_Alcam_1024x-Alcamtif.tif]

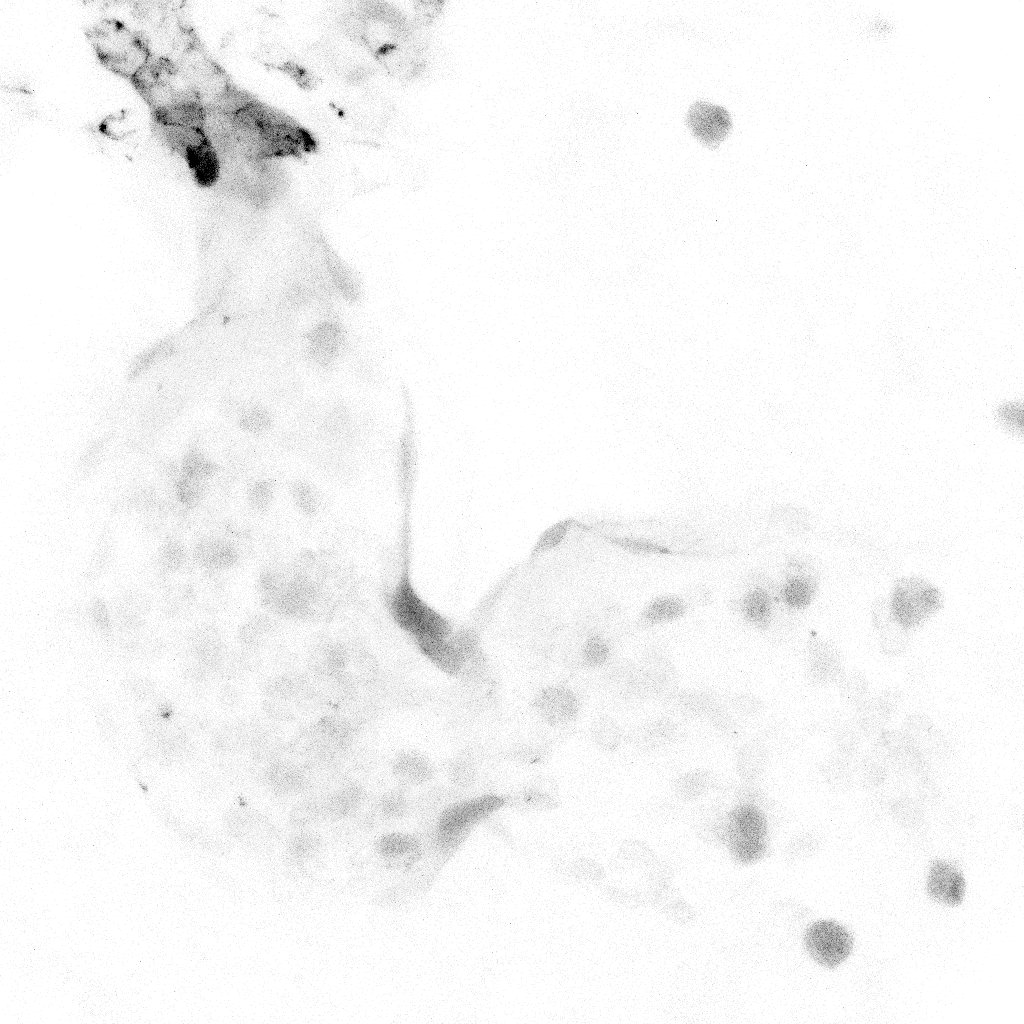

Supplement: Supplementary file 11 — Figure EV2 Source Data [file 44321_2024_152_MOESM11_ESM.zip › Figure EV2/N/MAX_52_stdMO_krit1sib_cbx7aMO_Alcam_1024x-Citrine.tif]

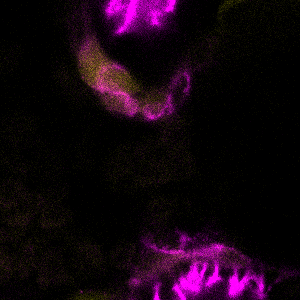

Supplement: Supplementary file 11 — Figure EV2 Source Data [file 44321_2024_152_MOESM11_ESM.zip › Figure EV2/N/MAX_52_stdMO_krit1sib_cbx7aMO_Alcam_1024x-z22-1.tif]

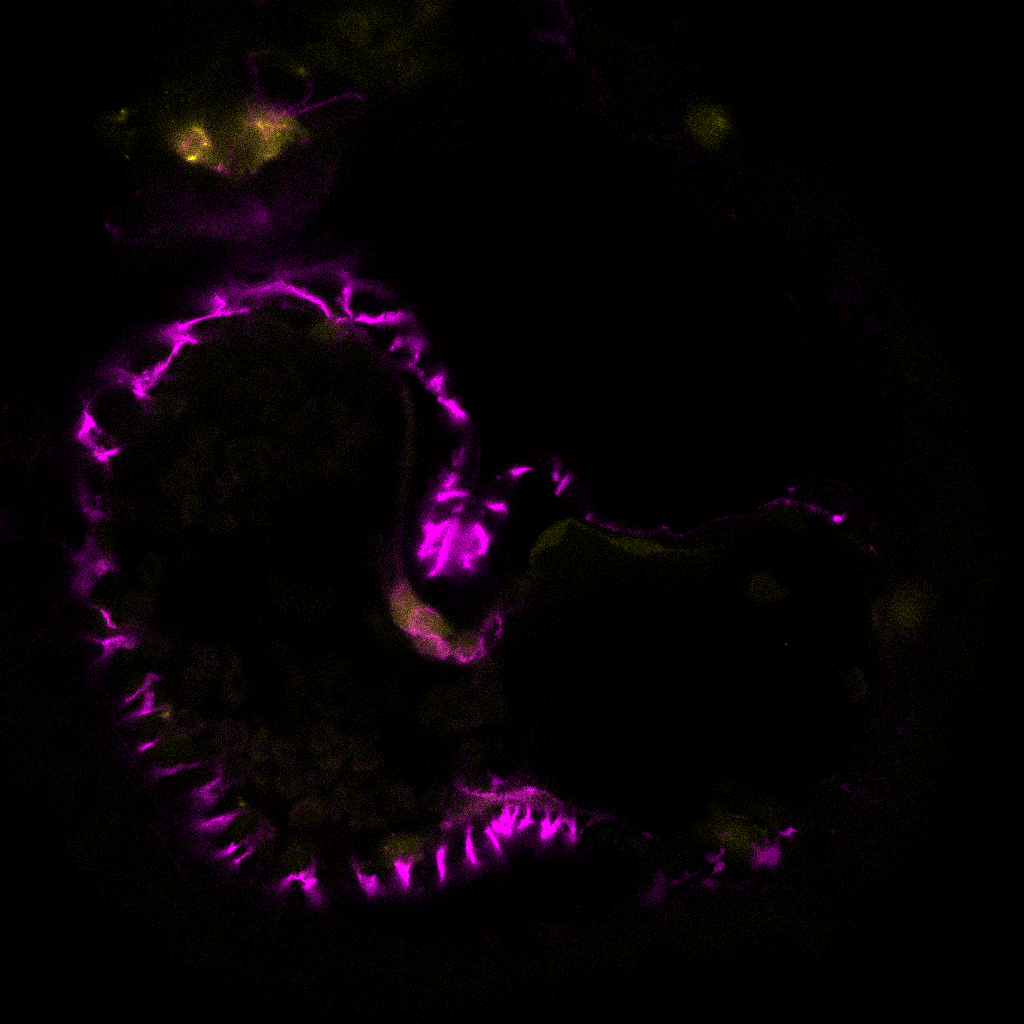

Supplement: Supplementary file 11 — Figure EV2 Source Data [file 44321_2024_152_MOESM11_ESM.zip › Figure EV2/N/MAX_52_stdMO_krit1sib_cbx7aMO_Alcam_1024x-z22.tif]

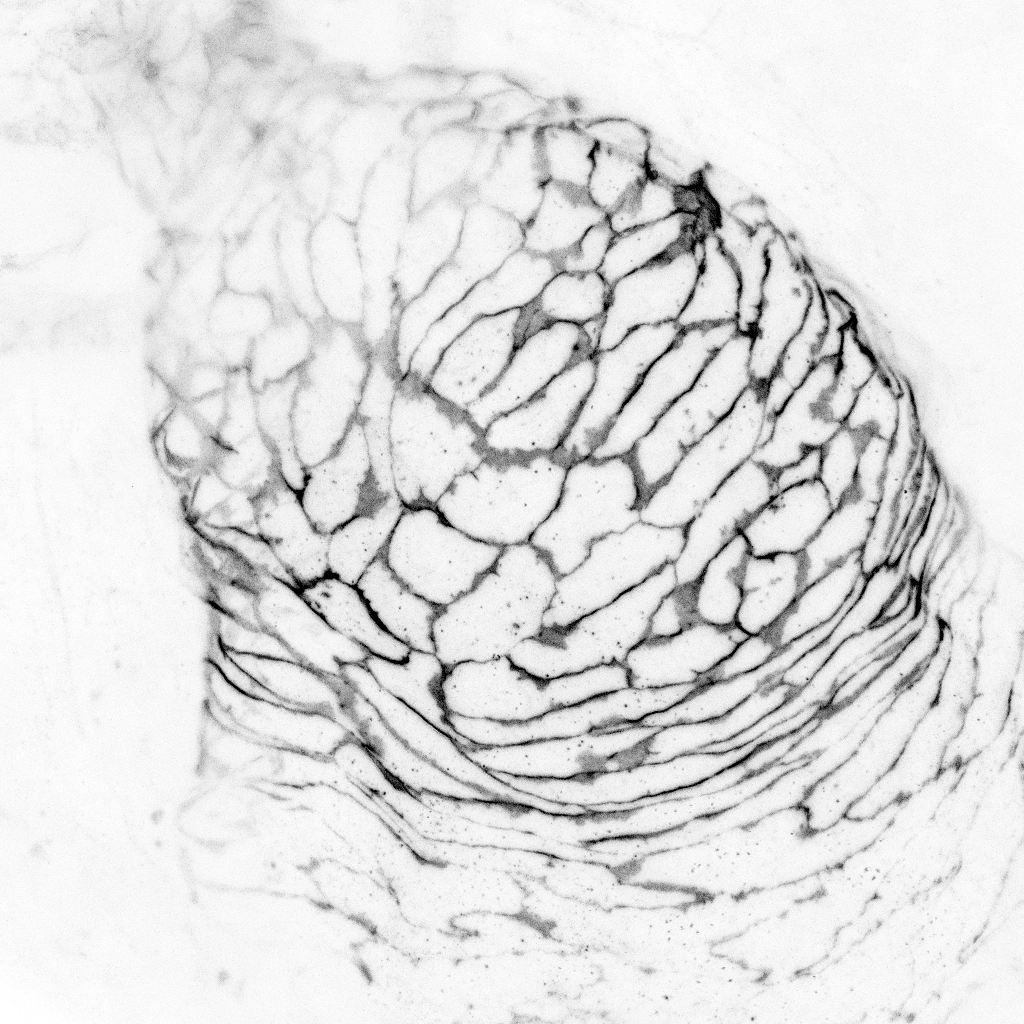

Supplement: Supplementary file 11 — Figure EV2 Source Data [file 44321_2024_152_MOESM11_ESM.zip › Figure EV2/O/MAX_60_stdMO_krit1mut_cbx7aMO_Alcam_1024x_Alcam.tif]

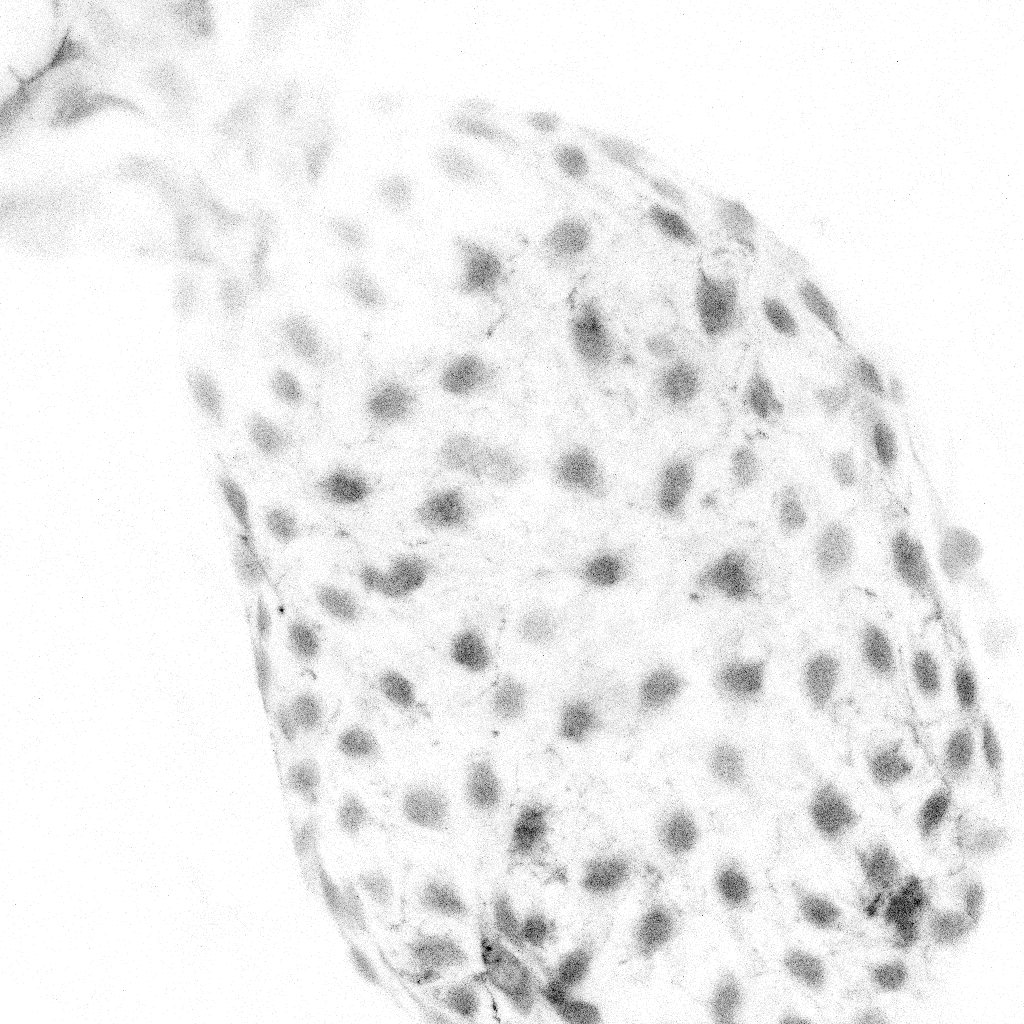

Supplement: Supplementary file 11 — Figure EV2 Source Data [file 44321_2024_152_MOESM11_ESM.zip › Figure EV2/O/MAX_60_stdMO_krit1mut_cbx7aMO_Alcam_1024x_Citrine.tif]

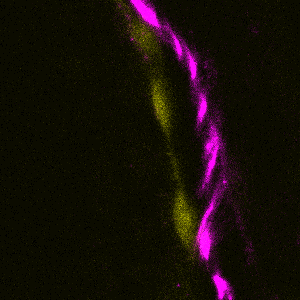

Supplement: Supplementary file 11 — Figure EV2 Source Data [file 44321_2024_152_MOESM11_ESM.zip › Figure EV2/O/MAX_60_stdMO_krit1mut_cbx7aMO_Alcam_1024x_z41-1.tif]

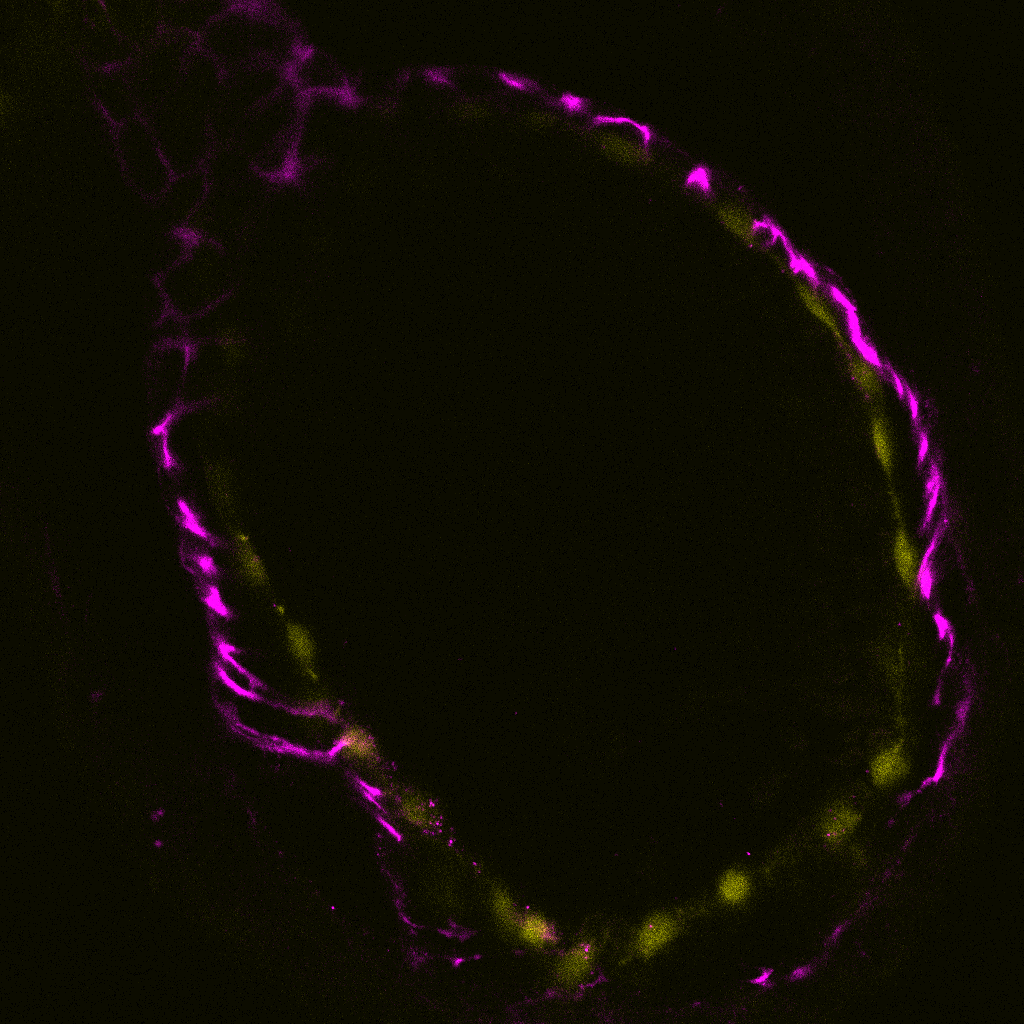

Supplement: Supplementary file 11 — Figure EV2 Source Data [file 44321_2024_152_MOESM11_ESM.zip › Figure EV2/O/MAX_60_stdMO_krit1mut_cbx7aMO_Alcam_1024x_z41.tif]

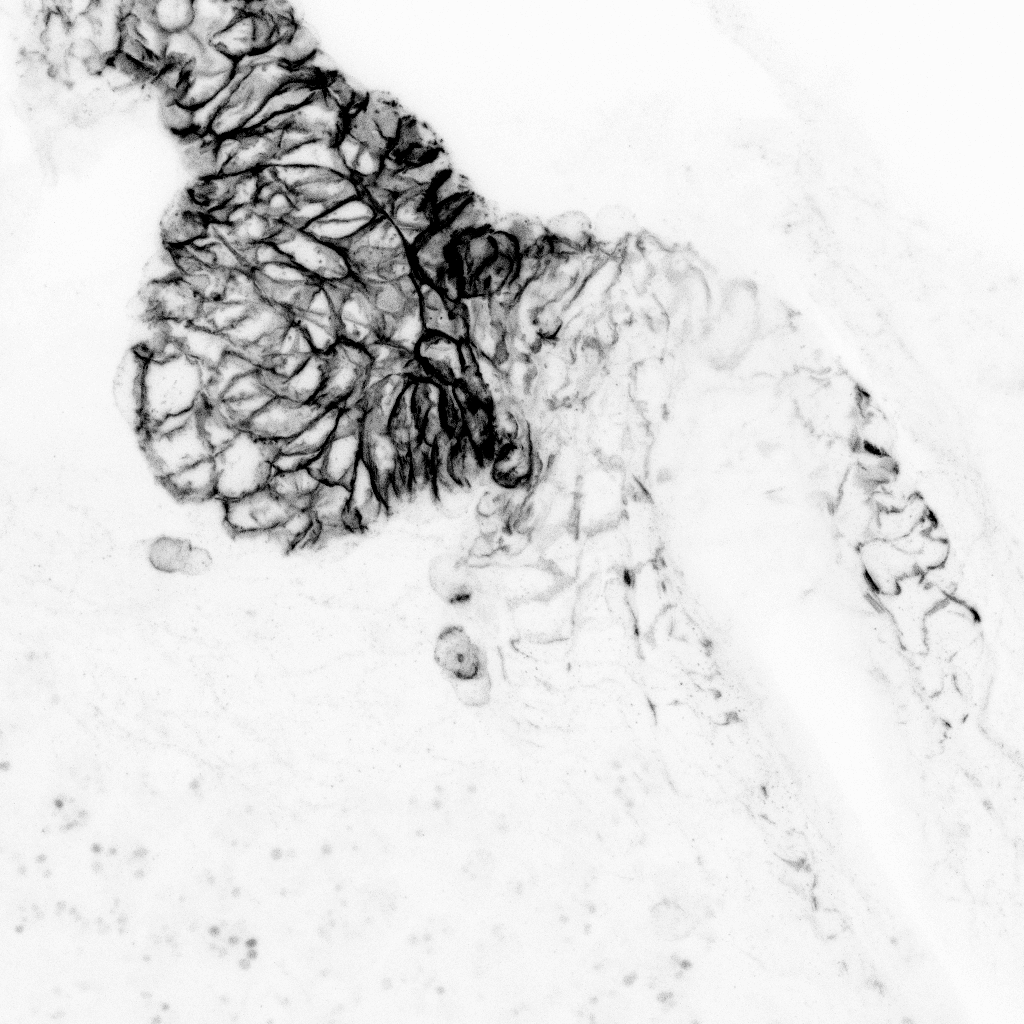

Supplement: Supplementary file 11 — Figure EV2 Source Data [file 44321_2024_152_MOESM11_ESM.zip › Figure EV2/P/MAX_33_krit1mut_cbx7aMO_Alcam_1024x-Alcam.tif]

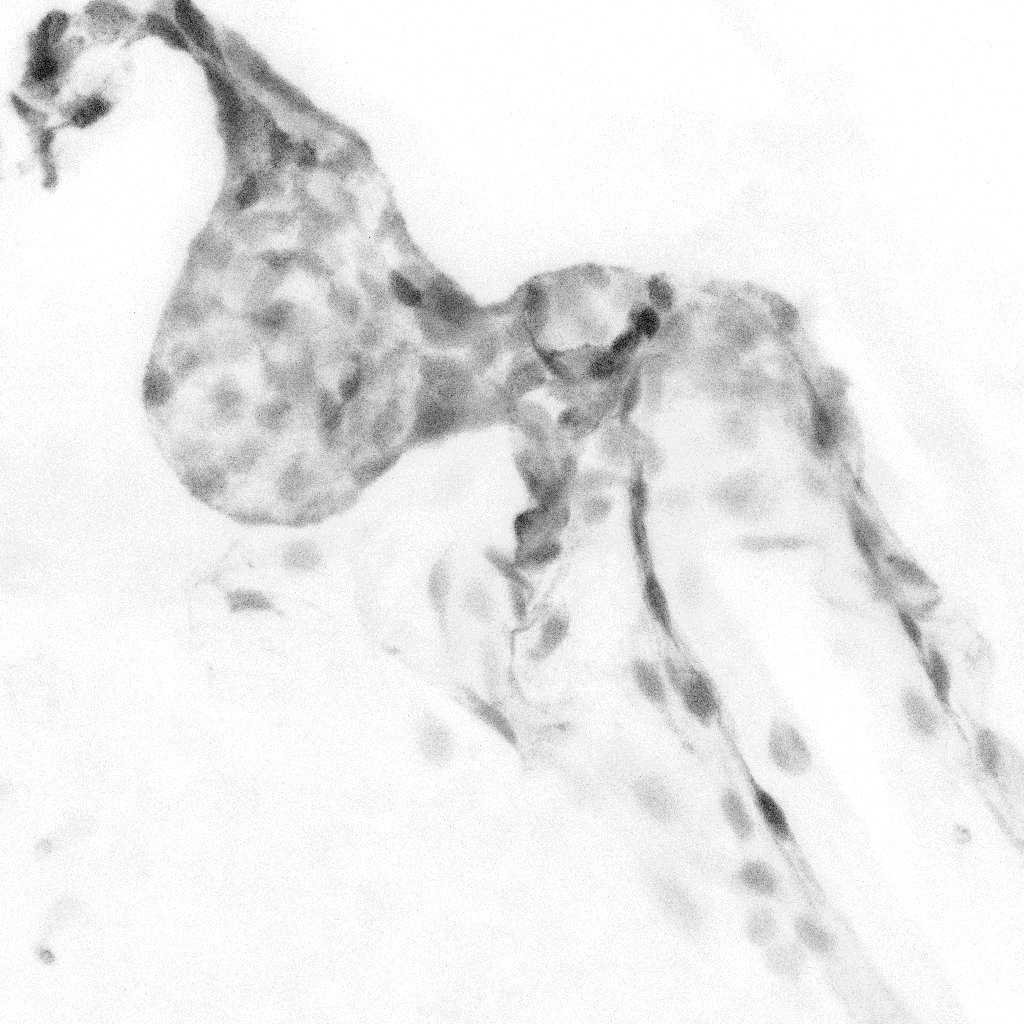

Supplement: Supplementary file 11 — Figure EV2 Source Data [file 44321_2024_152_MOESM11_ESM.zip › Figure EV2/P/MAX_33_krit1mut_cbx7aMO_Alcam_1024x-Citrine.tif]

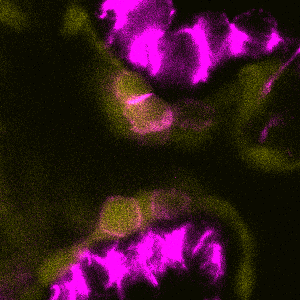

Supplement: Supplementary file 11 — Figure EV2 Source Data [file 44321_2024_152_MOESM11_ESM.zip › Figure EV2/P/MAX_33_krit1mut_cbx7aMO_Alcam_1024x-z28-1.tif]

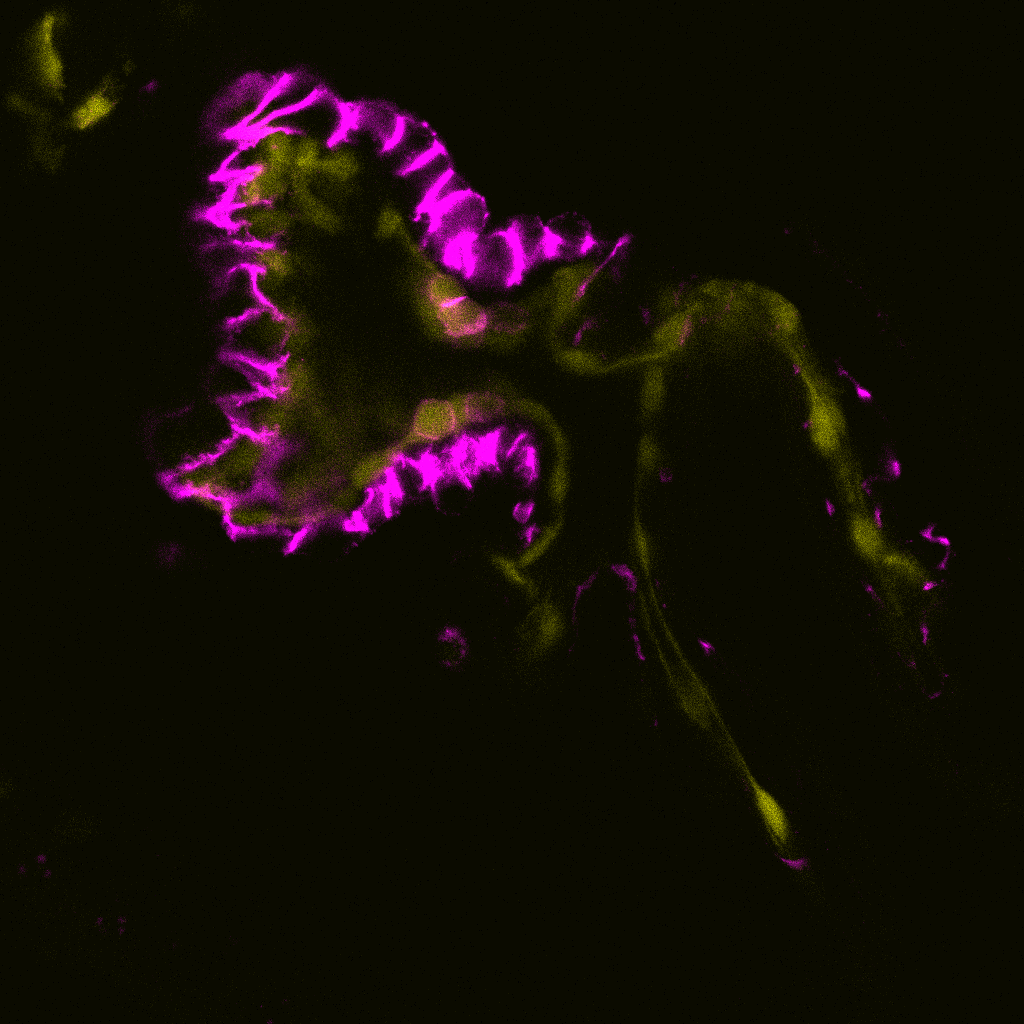

Supplement: Supplementary file 11 — Figure EV2 Source Data [file 44321_2024_152_MOESM11_ESM.zip › Figure EV2/P/MAX_33_krit1mut_cbx7aMO_Alcam_1024x-z28.tif]

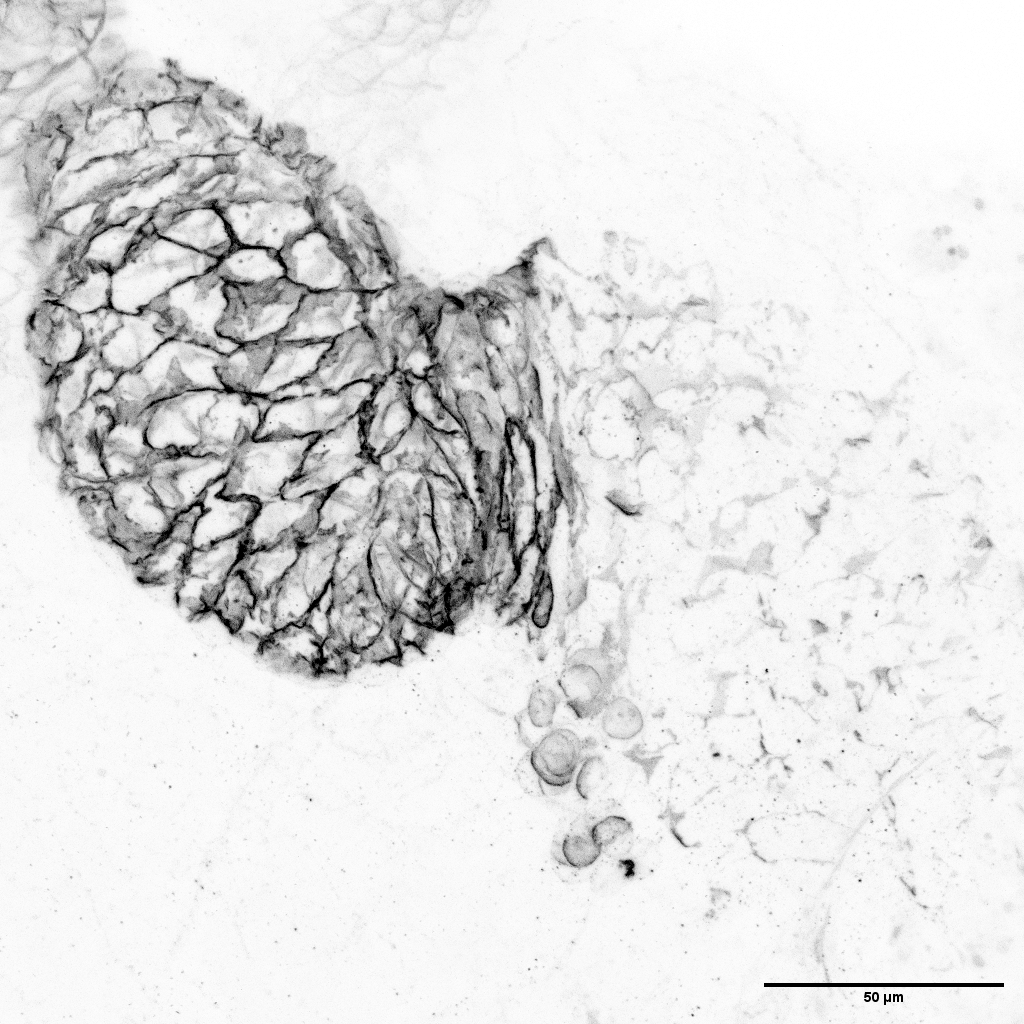

Supplement: Supplementary file 11 — Figure EV2 Source Data [file 44321_2024_152_MOESM11_ESM.zip › Figure EV2/Q/MAX_08_krit1sib_cbx7aMO_Alcam_1024x-Alcam-sb.tif]

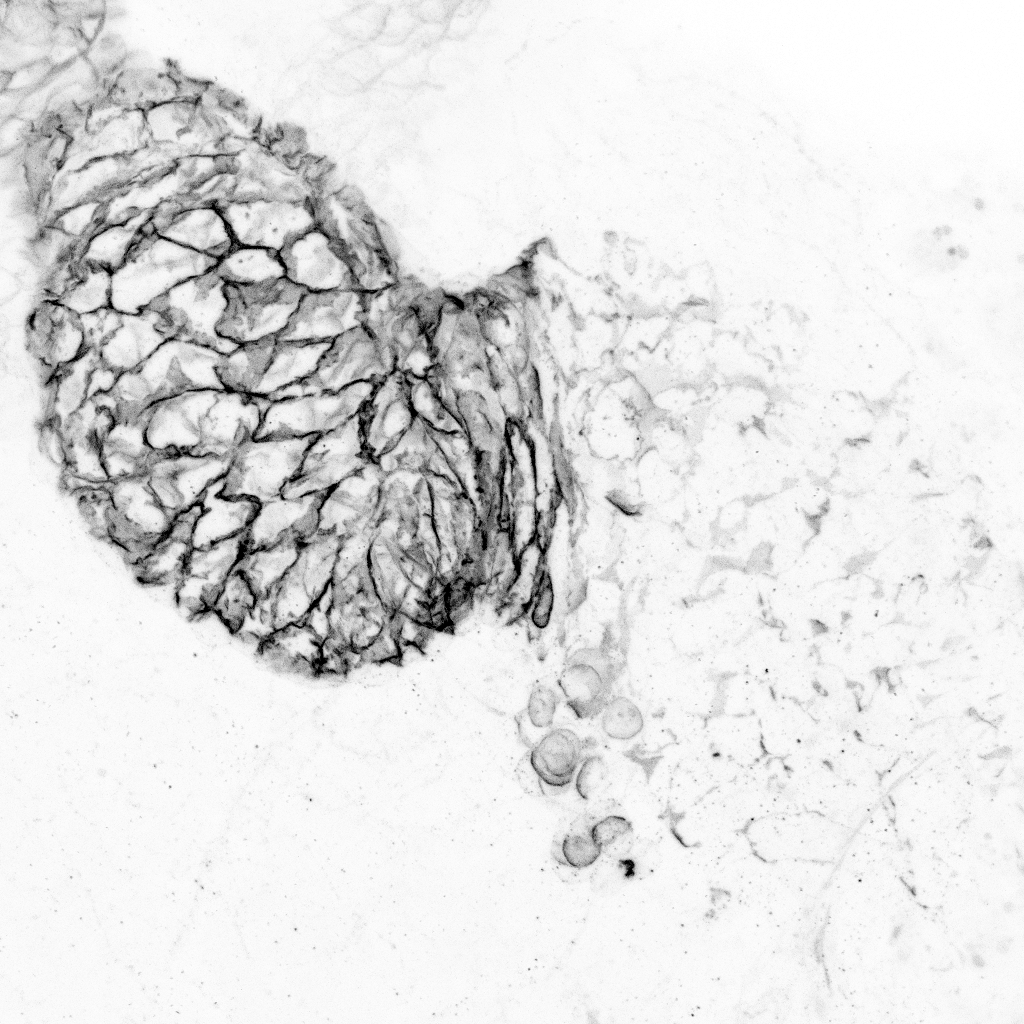

Supplement: Supplementary file 11 — Figure EV2 Source Data [file 44321_2024_152_MOESM11_ESM.zip › Figure EV2/Q/MAX_08_krit1sib_cbx7aMO_Alcam_1024x-Alcam.tif]

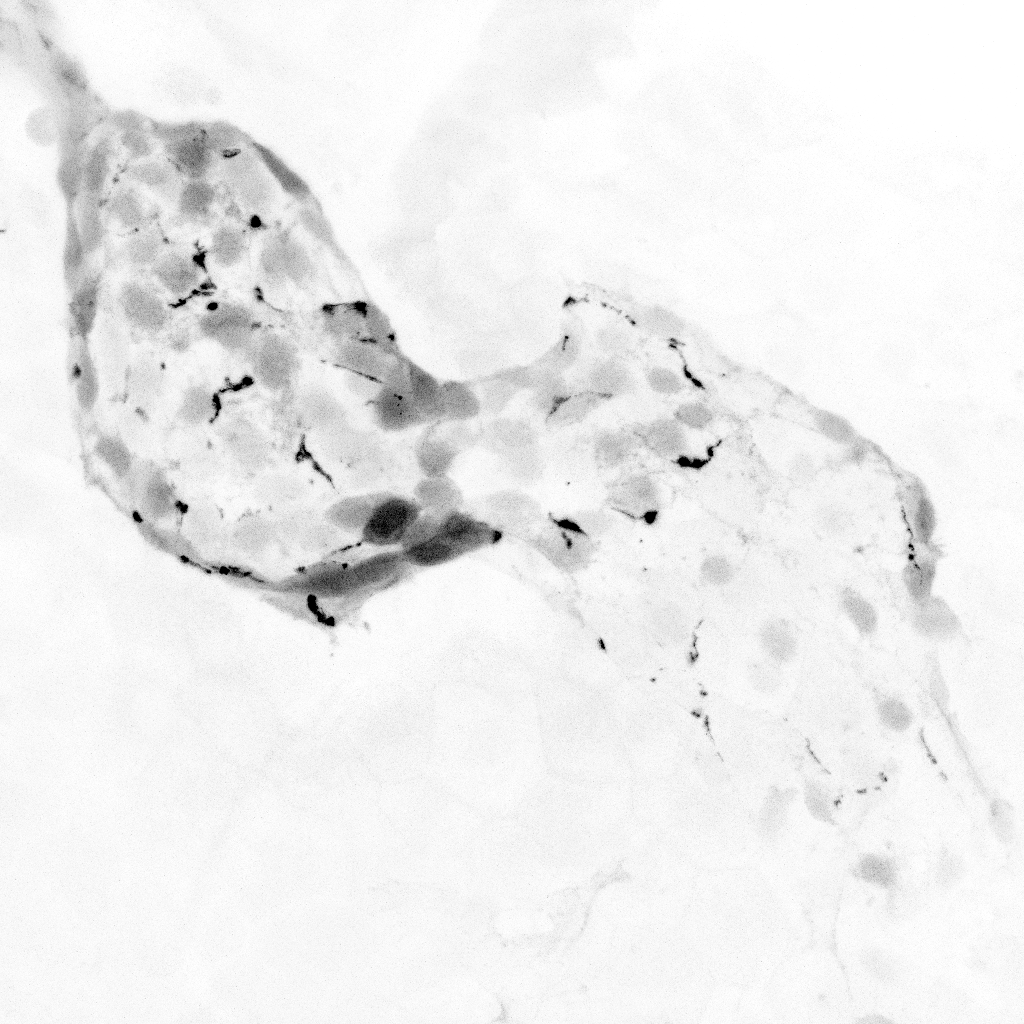

Supplement: Supplementary file 11 — Figure EV2 Source Data [file 44321_2024_152_MOESM11_ESM.zip › Figure EV2/Q/MAX_08_krit1sib_cbx7aMO_Alcam_1024x-Citrine.tif]

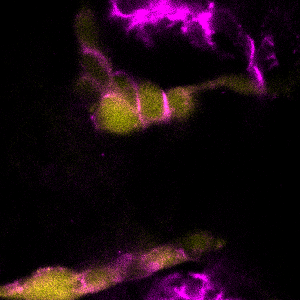

Supplement: Supplementary file 11 — Figure EV2 Source Data [file 44321_2024_152_MOESM11_ESM.zip › Figure EV2/Q/MAX_08_krit1sib_cbx7aMO_Alcam_1024x-z28-1.tif]

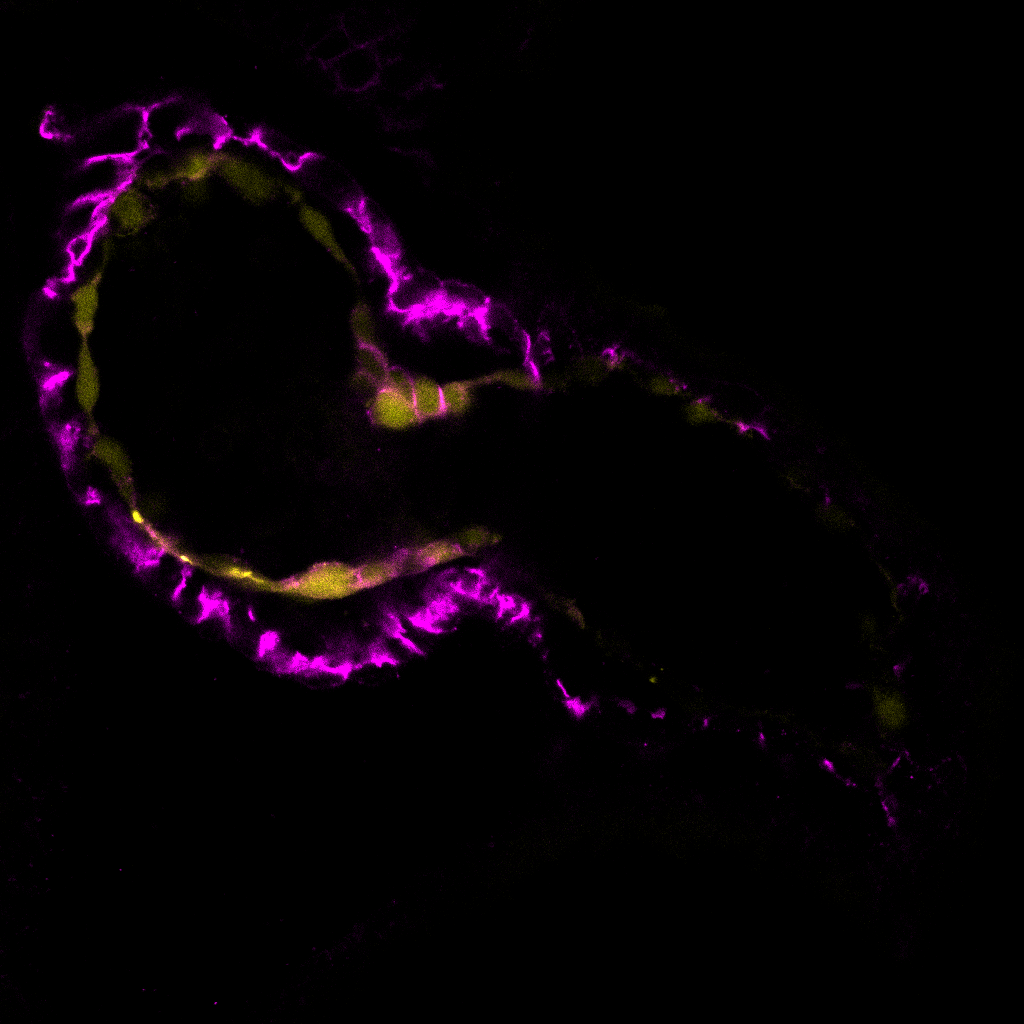

Supplement: Supplementary file 11 — Figure EV2 Source Data [file 44321_2024_152_MOESM11_ESM.zip › Figure EV2/Q/MAX_08_krit1sib_cbx7aMO_Alcam_1024x-z28.tif]

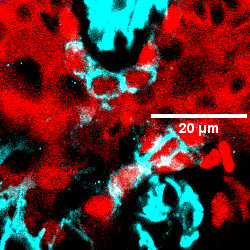

Supplement: Supplementary file 12 — Figure EV3 Source Data [file 44321_2024_152_MOESM12_ESM.zip › Figure EV3/A/MAX_2_ctr_4-250px-1scalebar.tif]
