# Supplementary figures and images for: Epigenetic regulation by polycomb repressive complex 1 promotes cerebral cavernous malformations (part 2 of 2)
Source: EMBO Mol Med. 2024 Oct 14;16(11):10. doi: 10.1038/s44321-024-00152-9 (PMC11555420; doi:10.1038/s44321-024-00152-9)

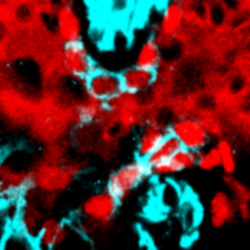

Supplement: Supplementary file 12 — Figure EV3 Source Data [file 44321_2024_152_MOESM12_ESM.zip › Figure EV3/A/MAX_2_ctr_4-250px-2.tif]

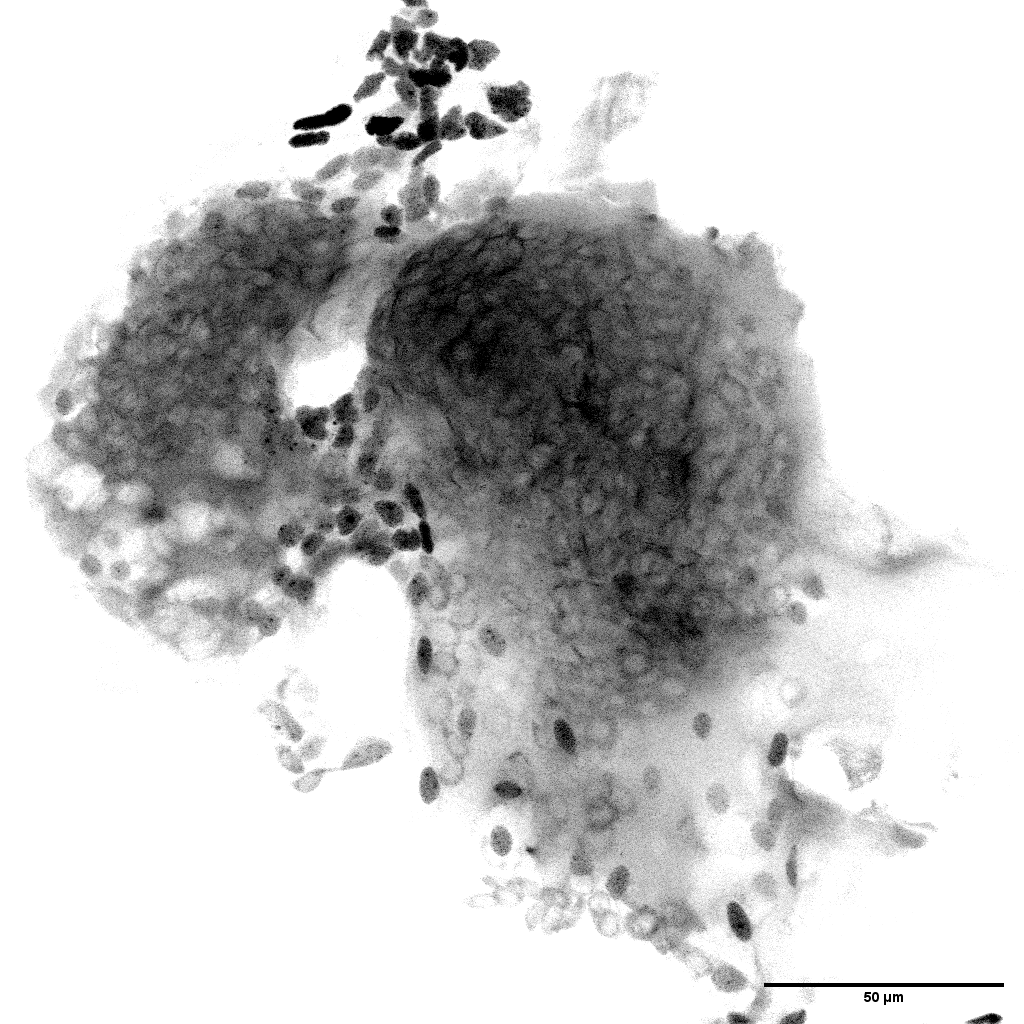

Supplement: Supplementary file 12 — Figure EV3 Source Data [file 44321_2024_152_MOESM12_ESM.zip › Figure EV3/A/MAX_2_ctr_4-BW-scalebar.tif]

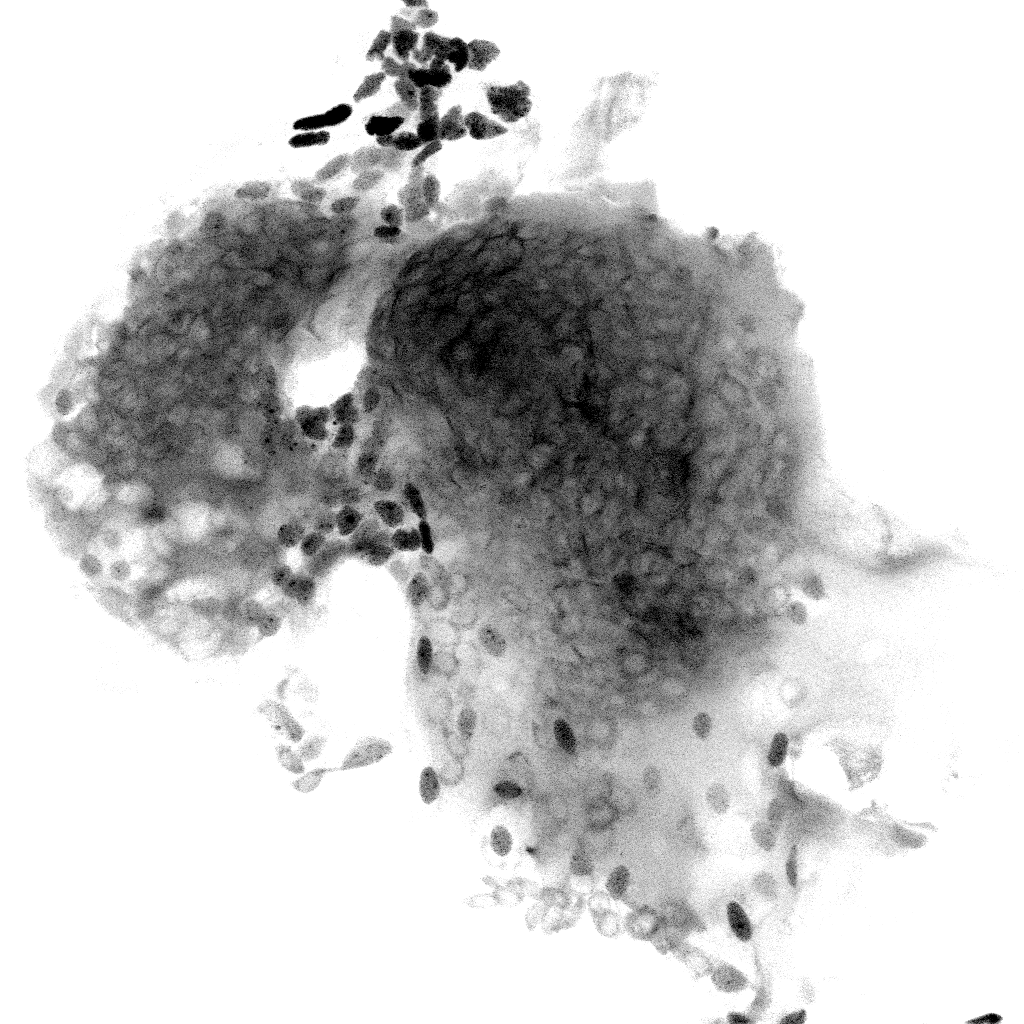

Supplement: Supplementary file 12 — Figure EV3 Source Data [file 44321_2024_152_MOESM12_ESM.zip › Figure EV3/A/MAX_2_ctr_4-BW.tif]

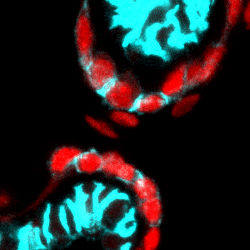

Supplement: Supplementary file 12 — Figure EV3 Source Data [file 44321_2024_152_MOESM12_ESM.zip › Figure EV3/B/MAX_1_klf2aOE_ctr_wAVC_2-1-250px.tif]

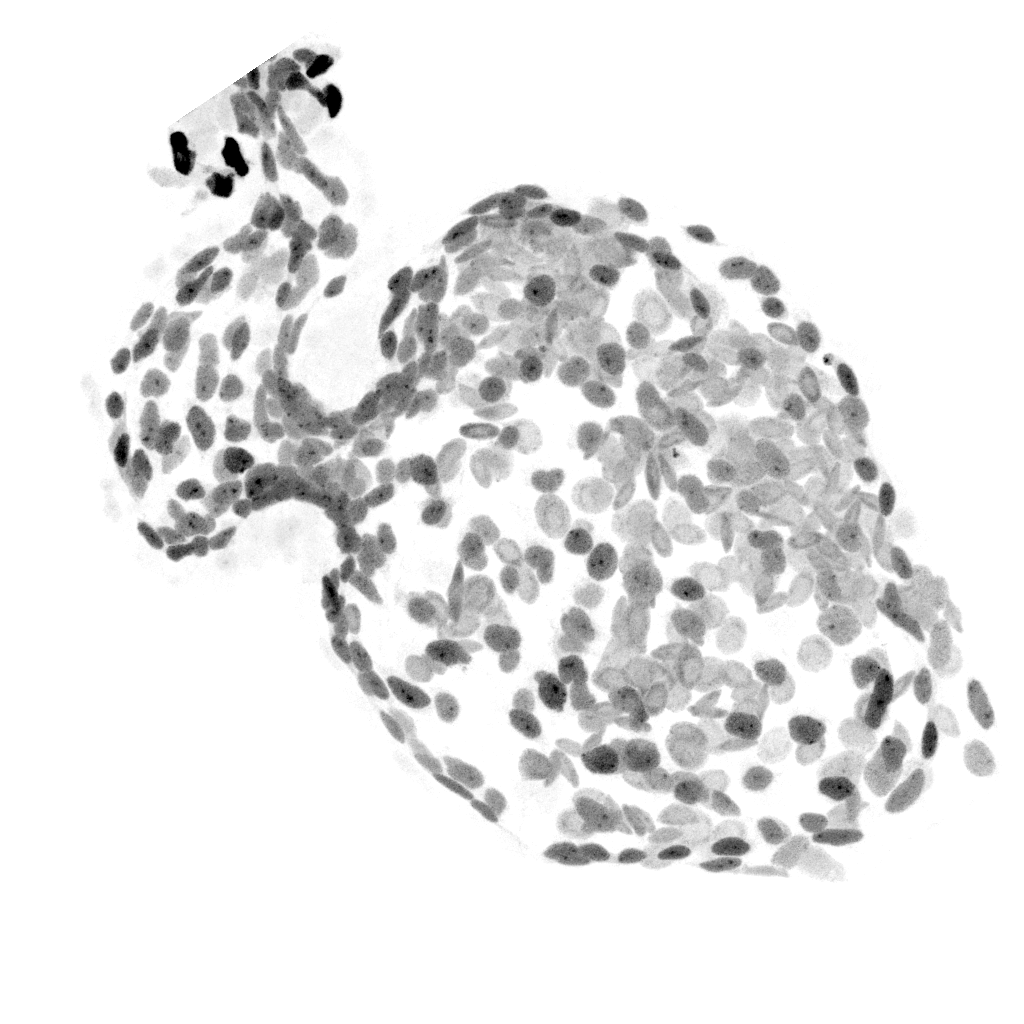

Supplement: Supplementary file 12 — Figure EV3 Source Data [file 44321_2024_152_MOESM12_ESM.zip › Figure EV3/B/MAX_1_klf2aOE_ctr_wAVC_2-BW.tif]

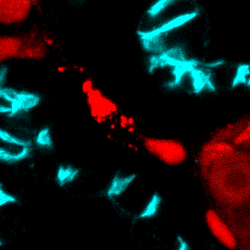

Supplement: Supplementary file 12 — Figure EV3 Source Data [file 44321_2024_152_MOESM12_ESM.zip › Figure EV3/C/MAX_4_sihMO_4-2-250px.tif]

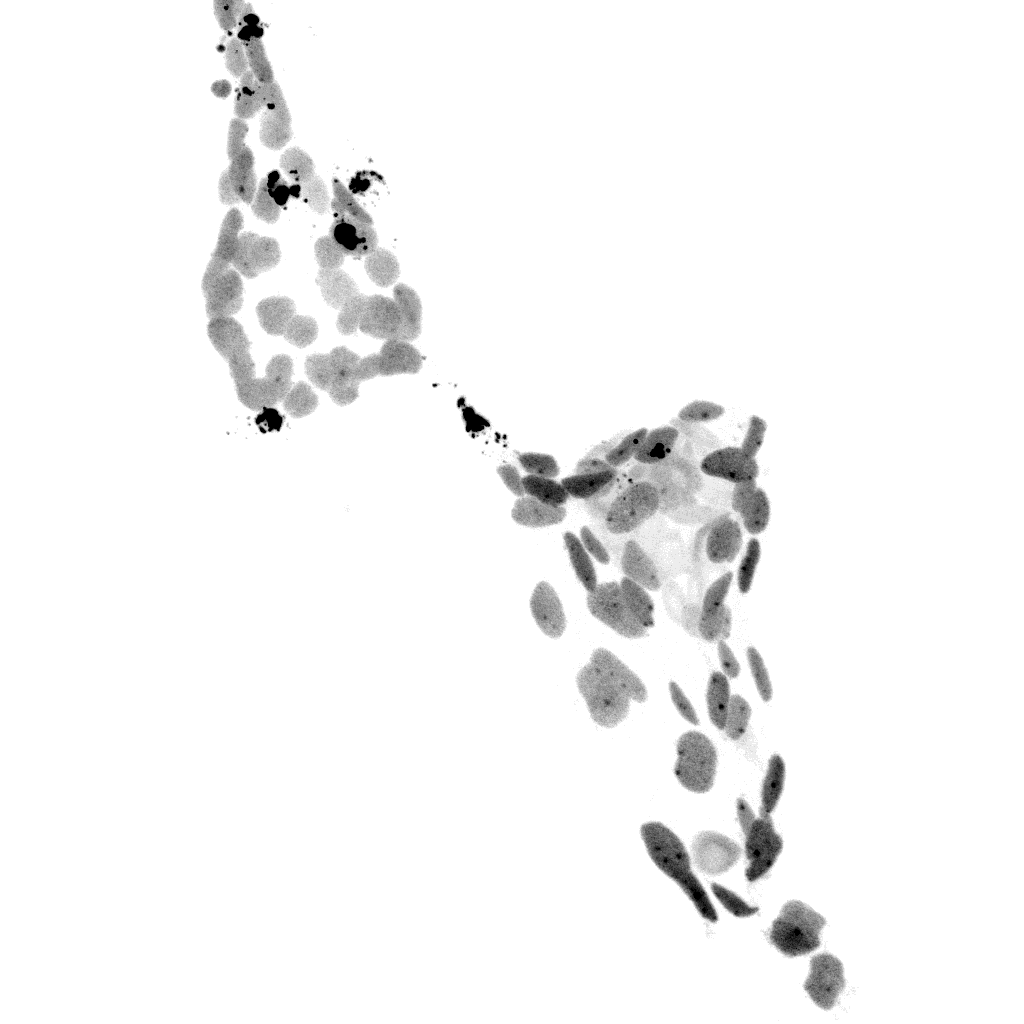

Supplement: Supplementary file 12 — Figure EV3 Source Data [file 44321_2024_152_MOESM12_ESM.zip › Figure EV3/C/MAX_4_sihMO_4-BW.tif]

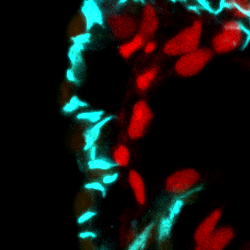

Supplement: Supplementary file 12 — Figure EV3 Source Data [file 44321_2024_152_MOESM12_ESM.zip › Figure EV3/D/MAX_3_klf2aOE_sihMO_dilated_2-250px.tif]

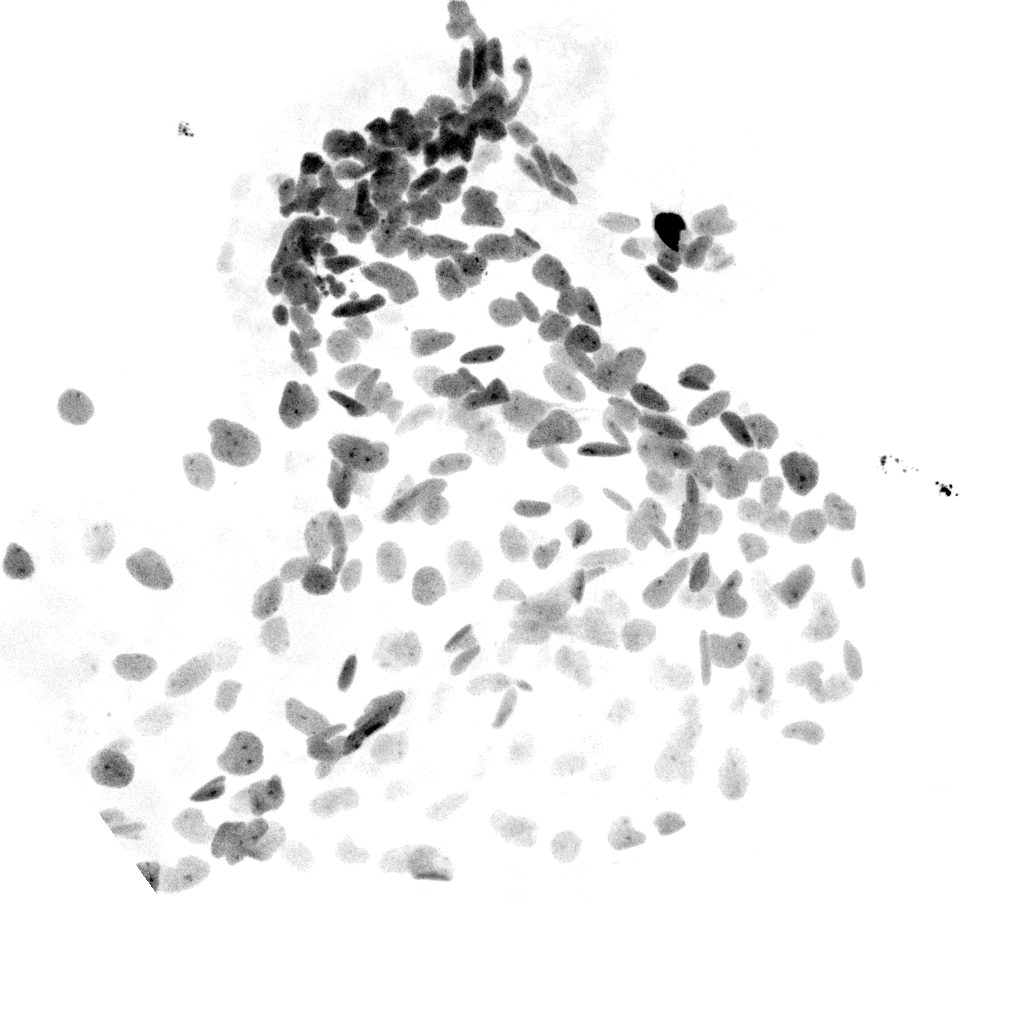

Supplement: Supplementary file 12 — Figure EV3 Source Data [file 44321_2024_152_MOESM12_ESM.zip › Figure EV3/D/MAX_3_klf2aOE_sihMO_dilated_2-BW.tif]

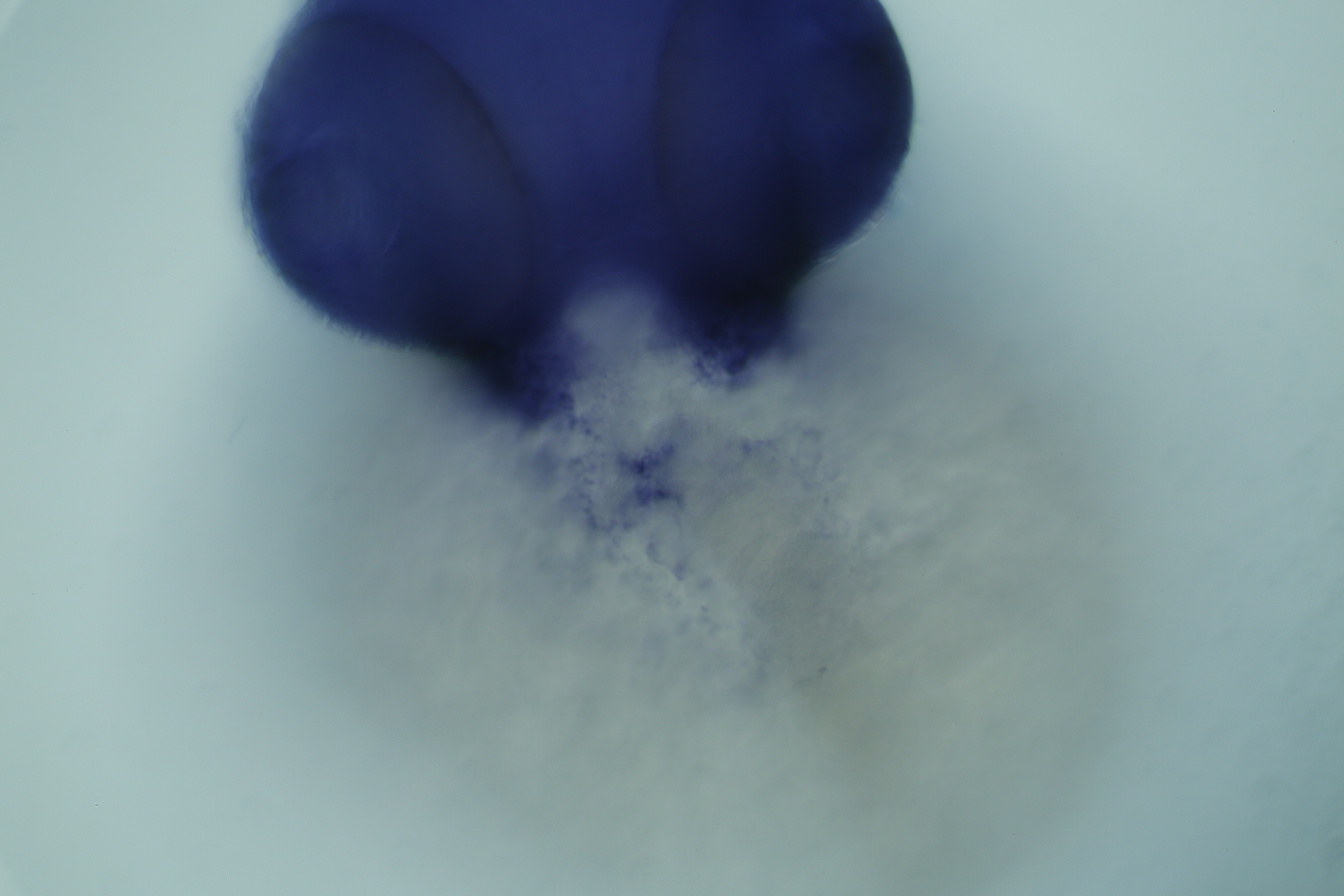

Supplement: Supplementary file 13 — Figure EV4 Source Data [file 44321_2024_152_MOESM13_ESM.zip › Figure EV4/A/Control.JPG]

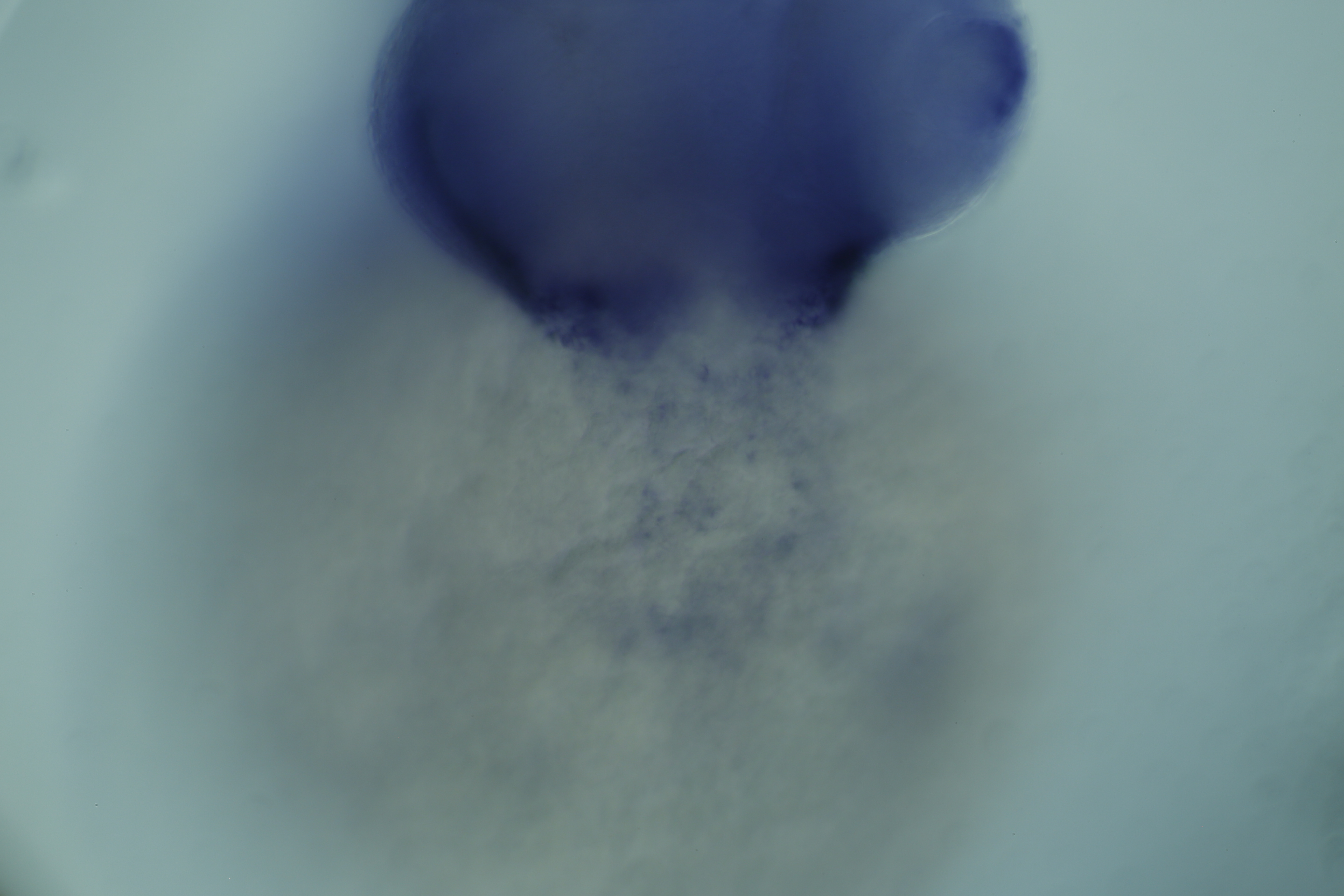

Supplement: Supplementary file 13 — Figure EV4 Source Data [file 44321_2024_152_MOESM13_ESM.zip › Figure EV4/B/Mutant.JPG]

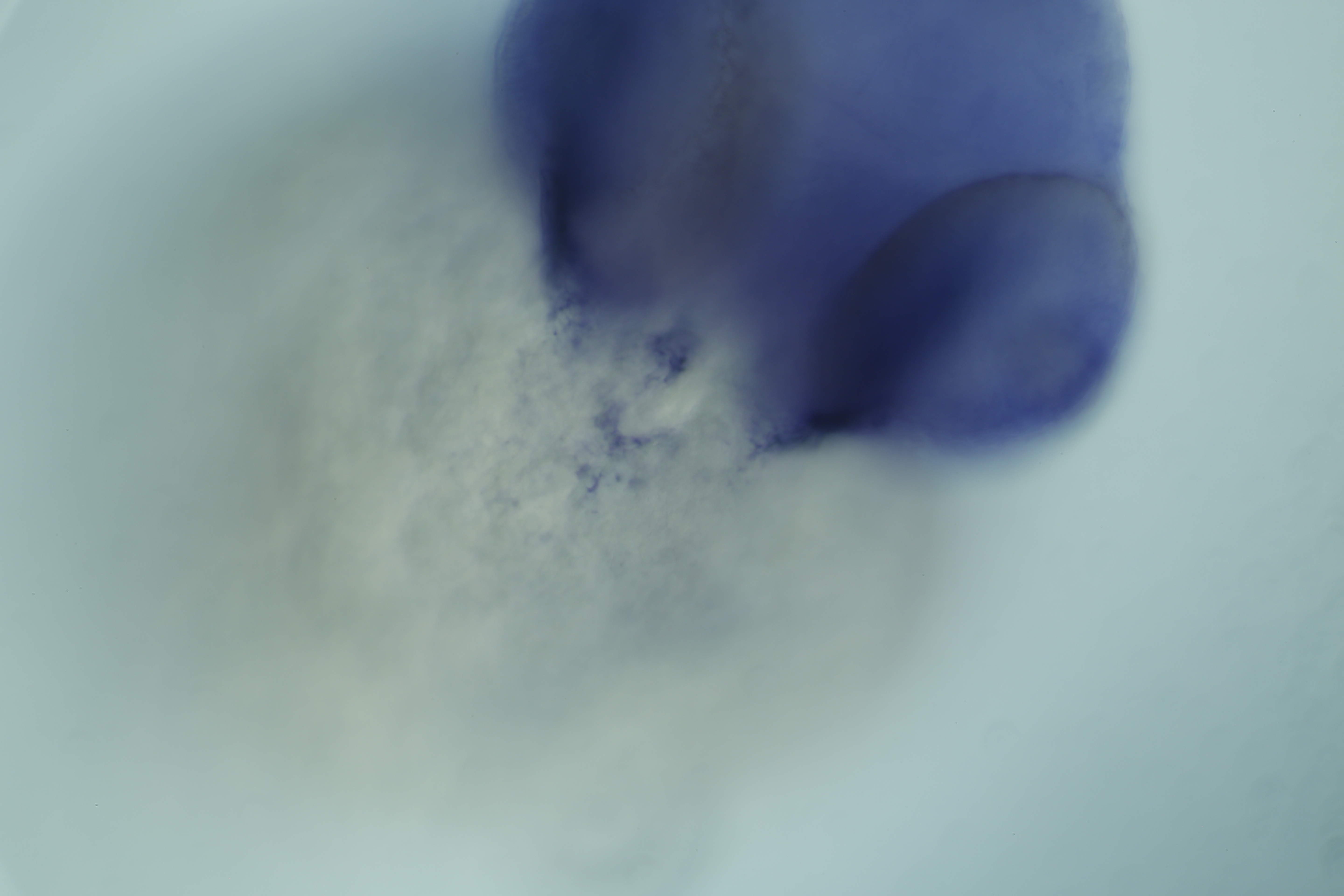

Supplement: Supplementary file 13 — Figure EV4 Source Data [file 44321_2024_152_MOESM13_ESM.zip › Figure EV4/C/Rescued.JPG]

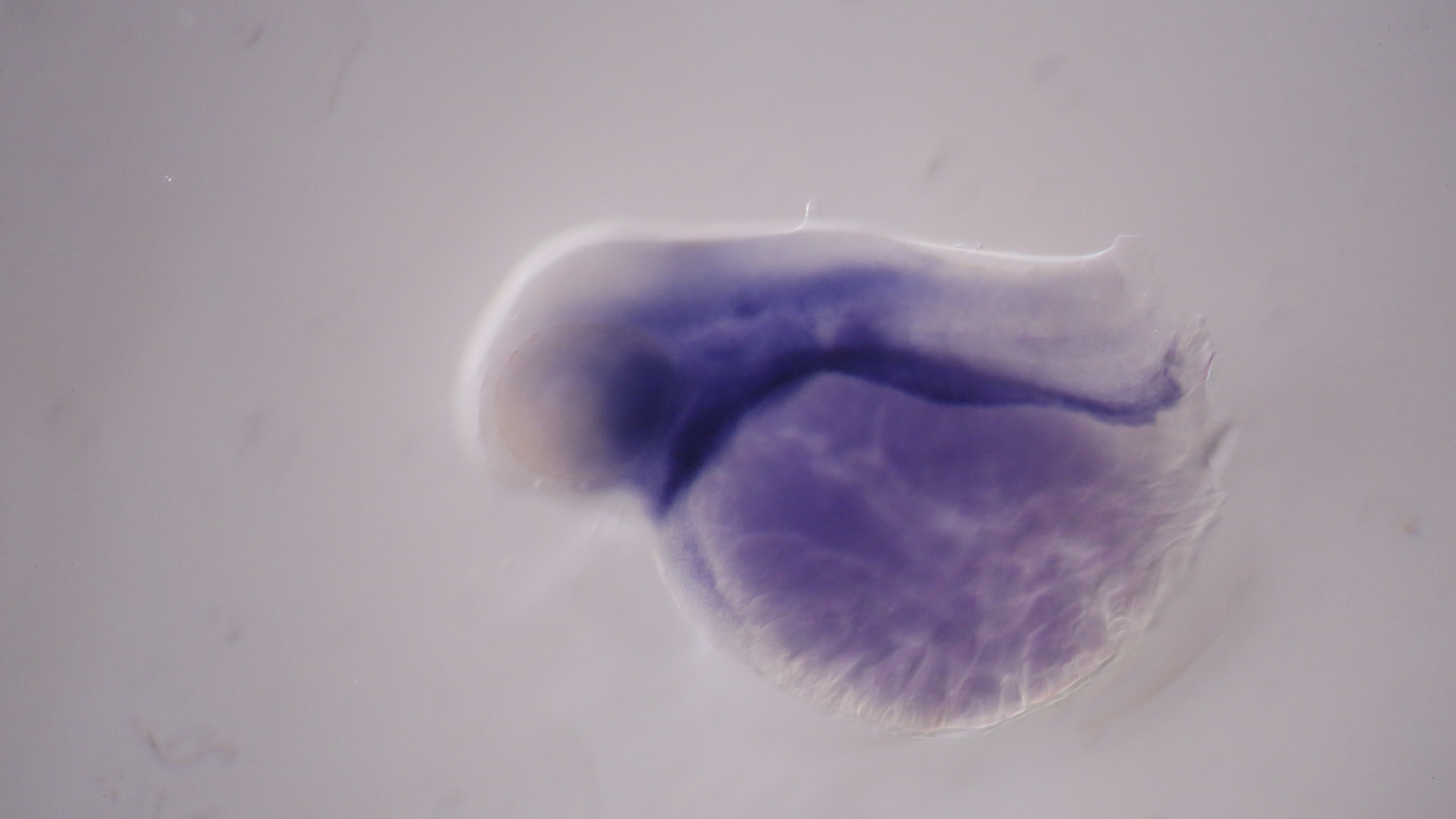

Supplement: Supplementary file 13 — Figure EV4 Source Data [file 44321_2024_152_MOESM13_ESM.zip › Figure EV4/D/WISH_wnt9b-_44.JPG]

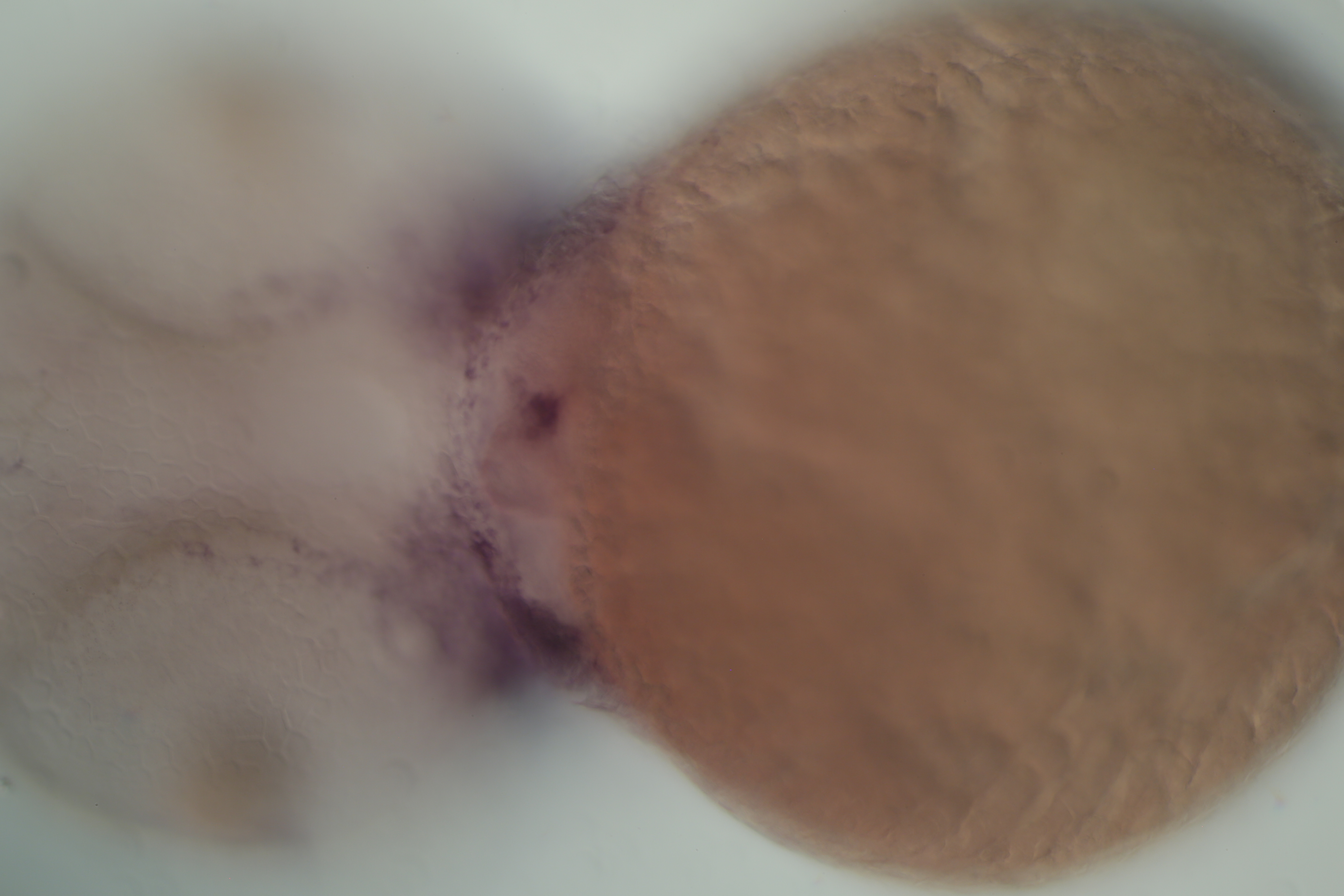

Supplement: Supplementary file 13 — Figure EV4 Source Data [file 44321_2024_152_MOESM13_ESM.zip › Figure EV4/D/WtFrontal.JPG]

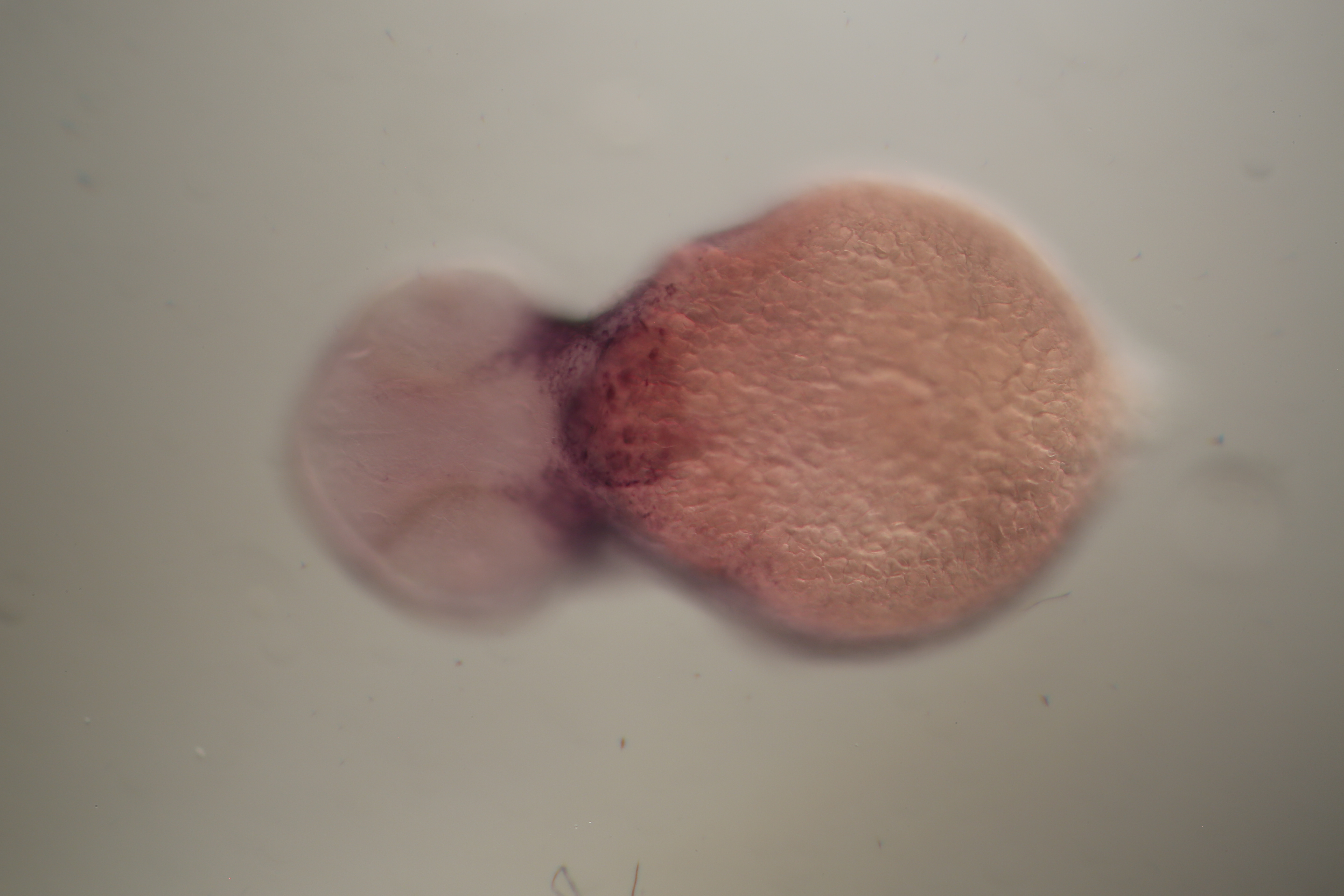

Supplement: Supplementary file 13 — Figure EV4 Source Data [file 44321_2024_152_MOESM13_ESM.zip › Figure EV4/E/Ccm2Frontal.JPG]

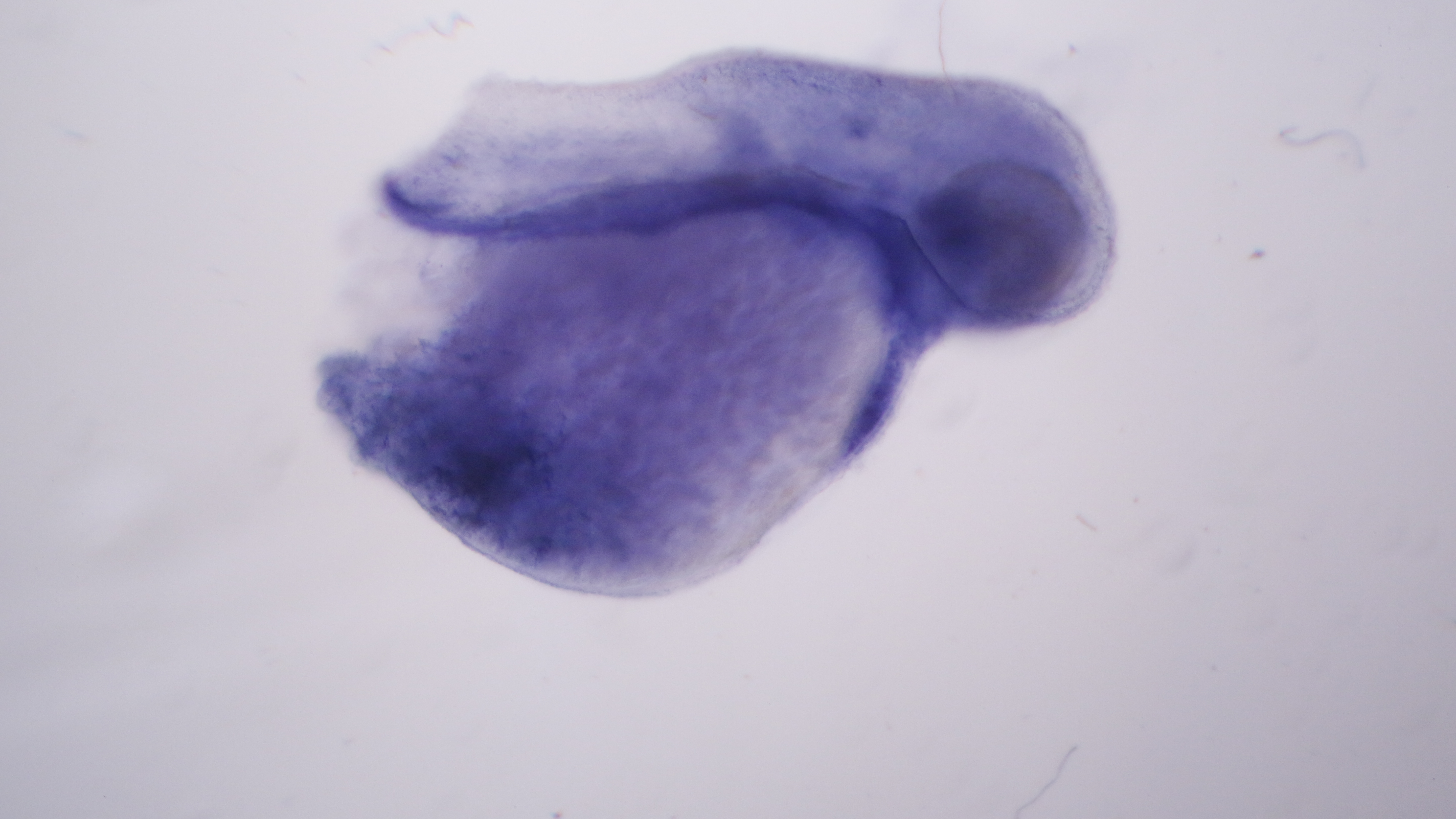

Supplement: Supplementary file 13 — Figure EV4 Source Data [file 44321_2024_152_MOESM13_ESM.zip › Figure EV4/E/WISH_wnt9b-_54.JPG]

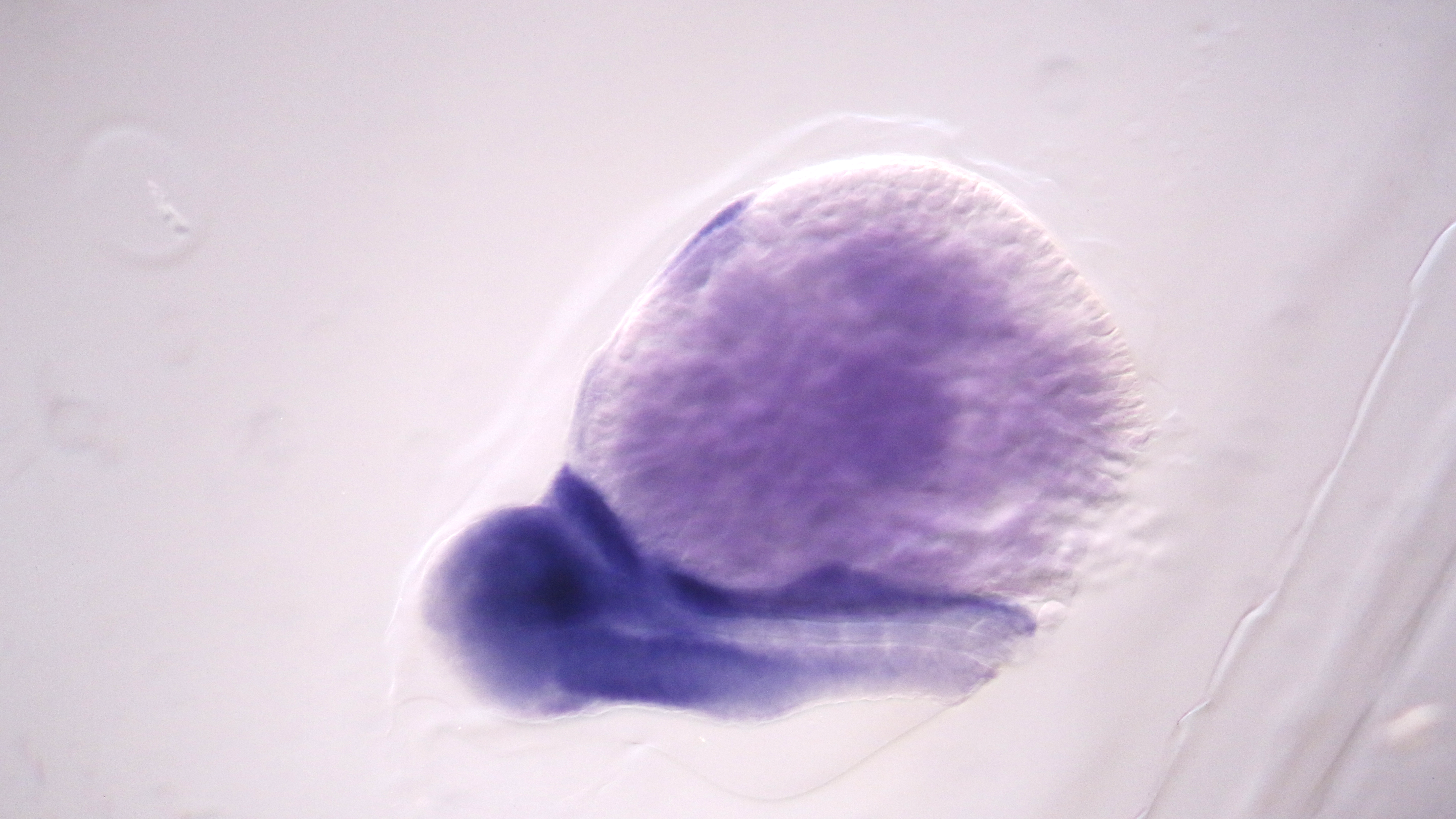

Supplement: Supplementary file 13 — Figure EV4 Source Data [file 44321_2024_152_MOESM13_ESM.zip › Figure EV4/F/WISH_wnt9b-_41.tif]

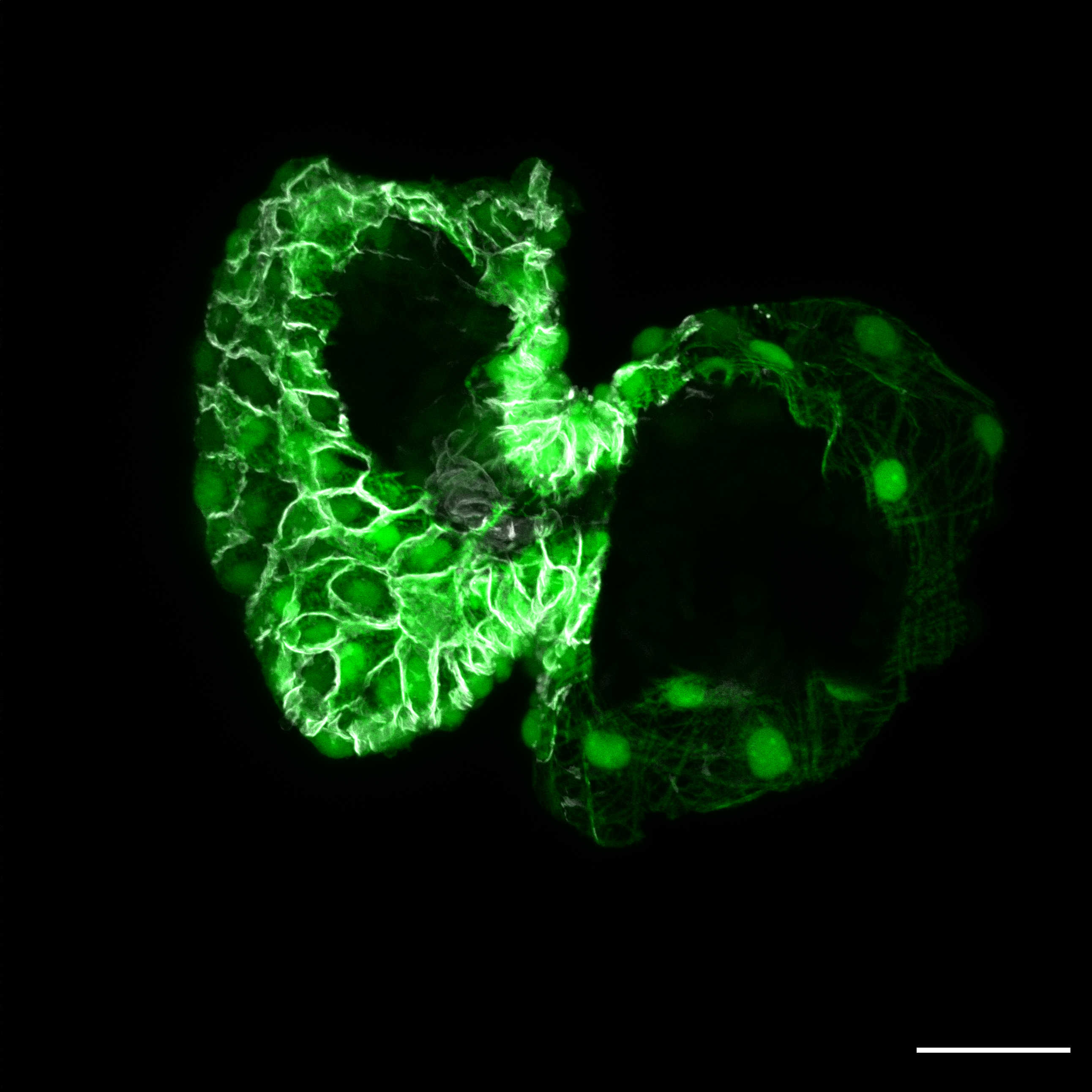

Supplement: Supplementary file 13 — Figure EV4 Source Data [file 44321_2024_152_MOESM13_ESM.zip › Figure EV4/G/MAX_WtCtr7.png]

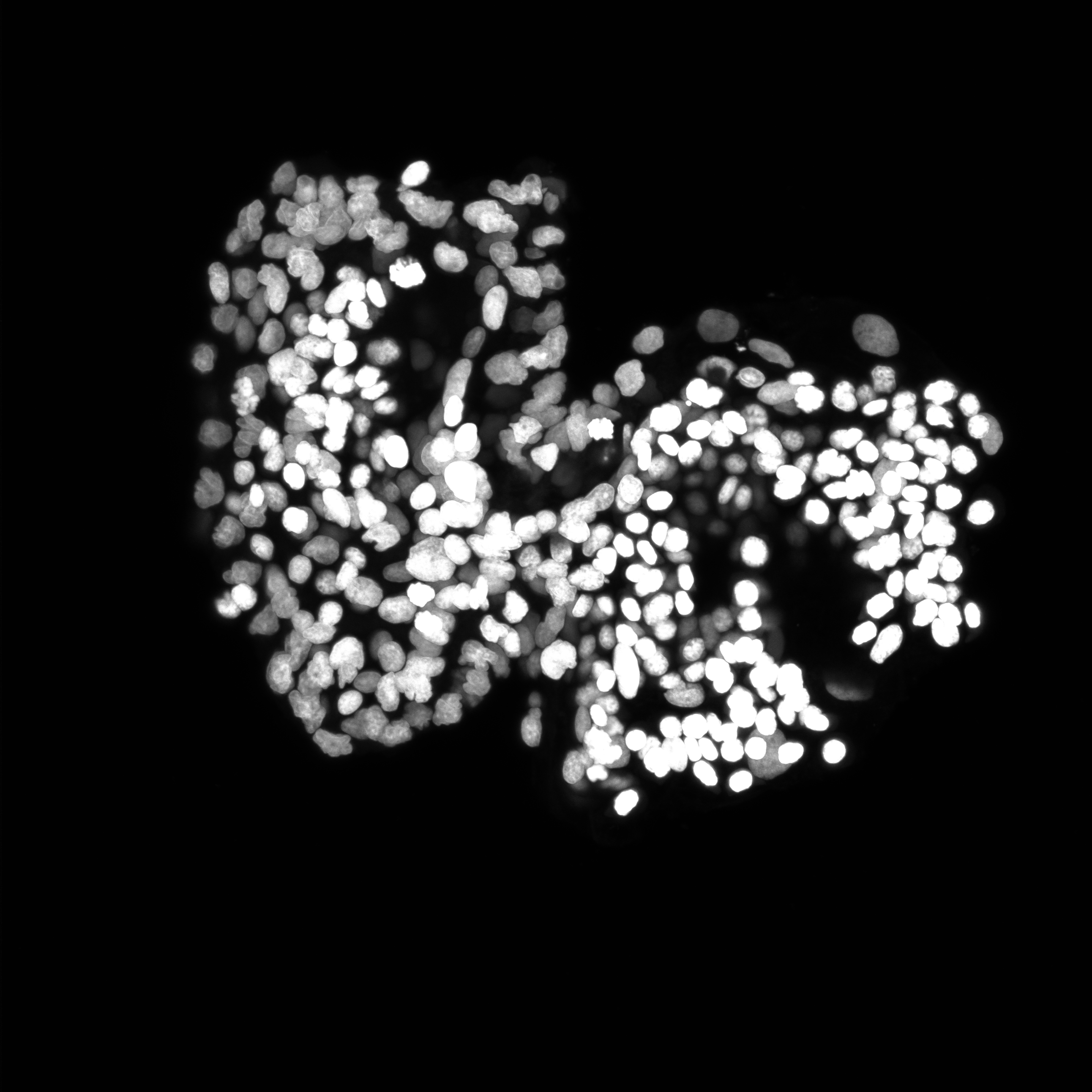

Supplement: Supplementary file 13 — Figure EV4 Source Data [file 44321_2024_152_MOESM13_ESM.zip › Figure EV4/G/MAX_WtCtr7.tif]

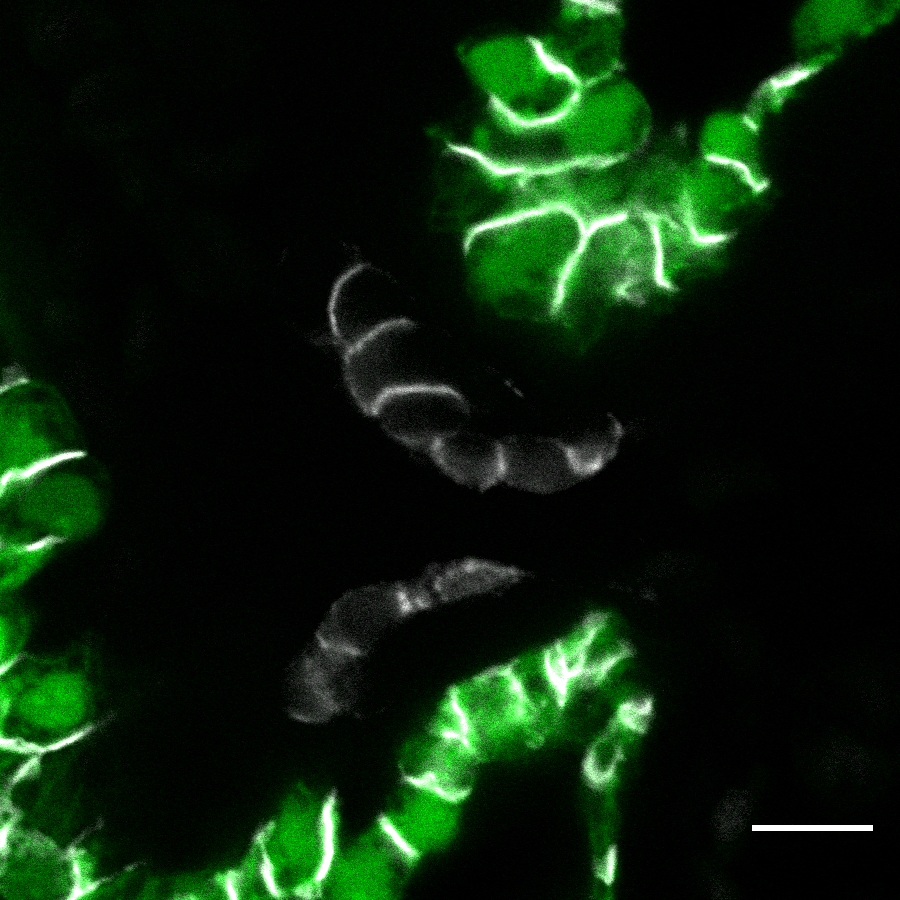

Supplement: Supplementary file 13 — Figure EV4 Source Data [file 44321_2024_152_MOESM13_ESM.zip › Figure EV4/G/WtCtr7zoomgreengrey.png]

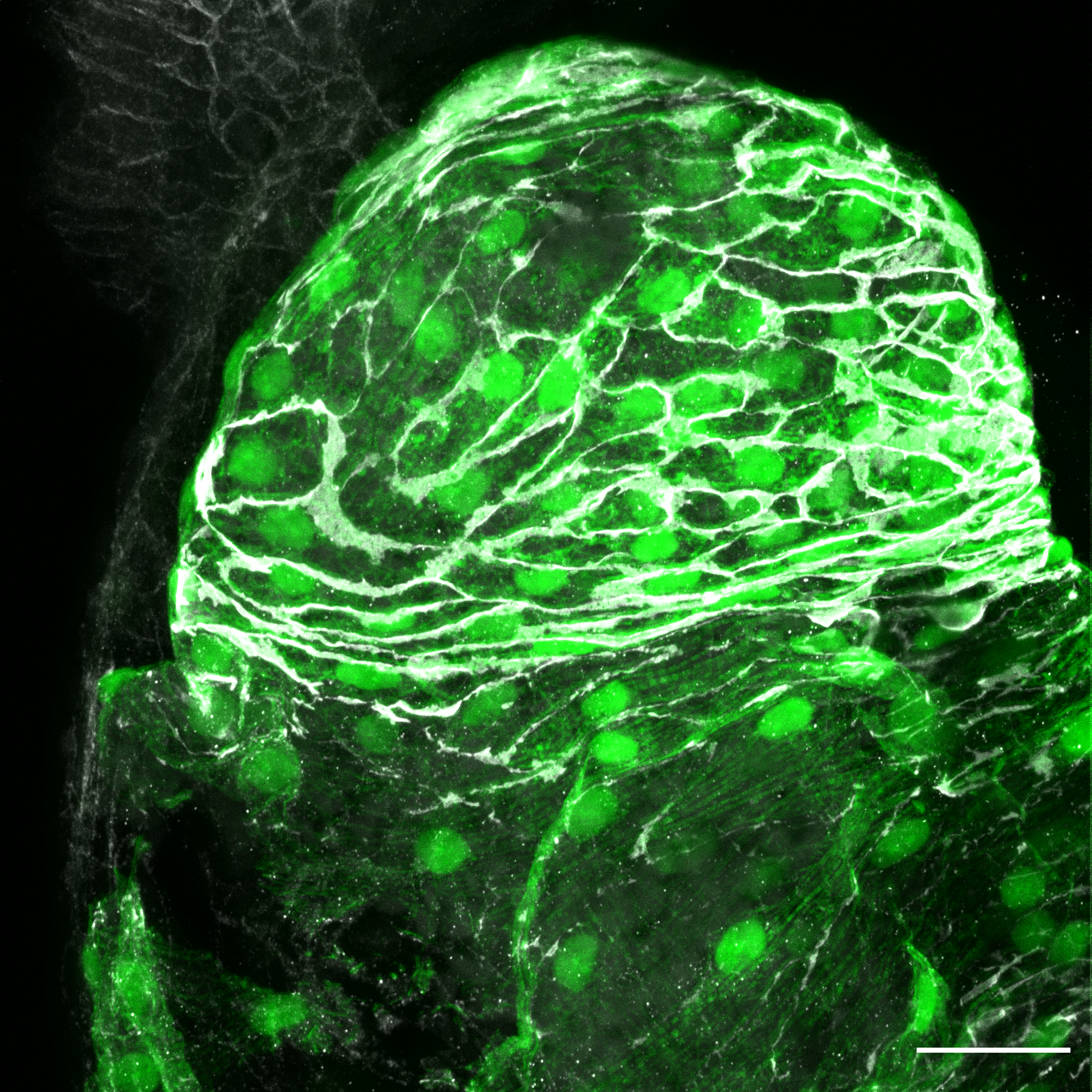

Supplement: Supplementary file 13 — Figure EV4 Source Data [file 44321_2024_152_MOESM13_ESM.zip › Figure EV4/H/MAX_MutCtr3.png]

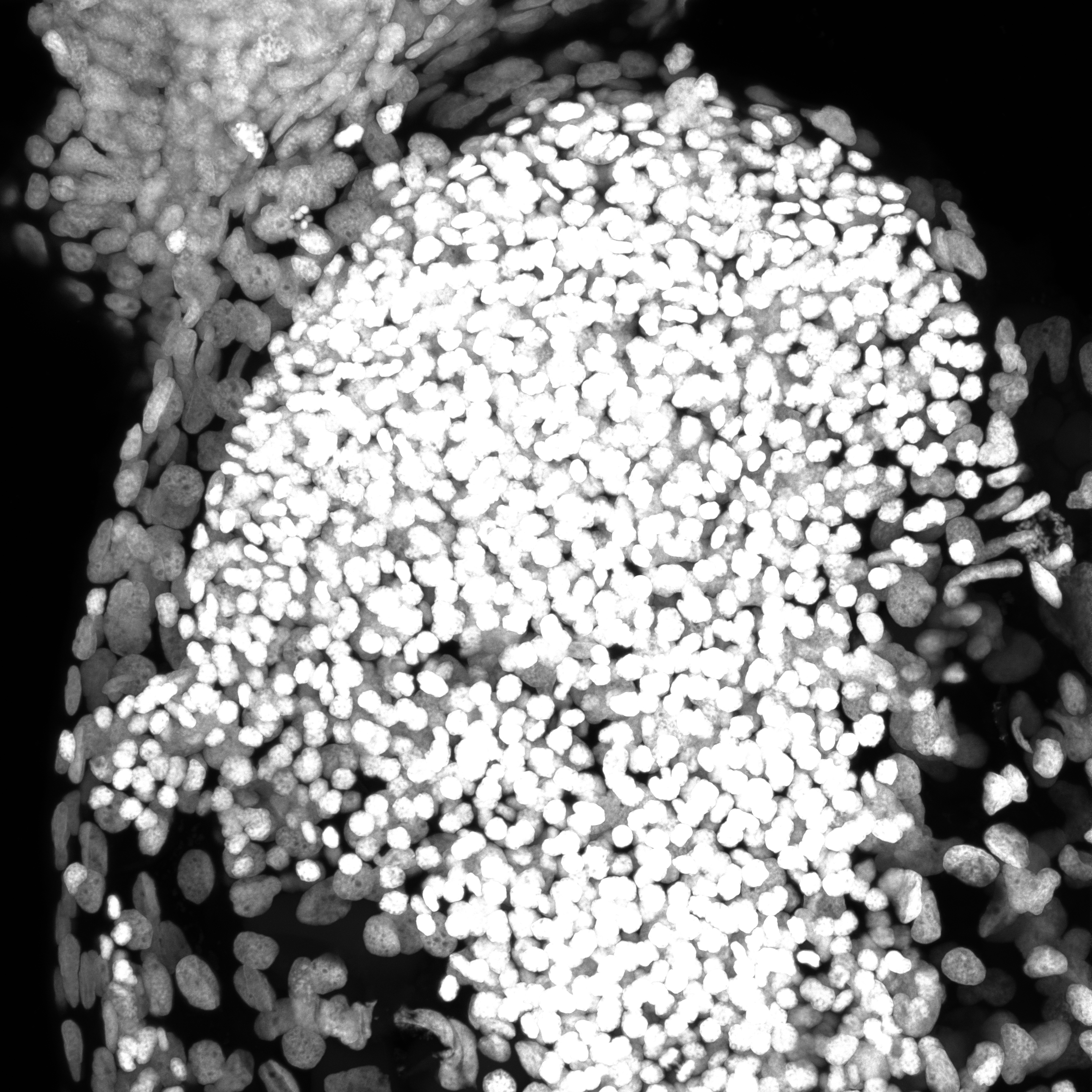

Supplement: Supplementary file 13 — Figure EV4 Source Data [file 44321_2024_152_MOESM13_ESM.zip › Figure EV4/H/MAX_MutCtr3.tif]

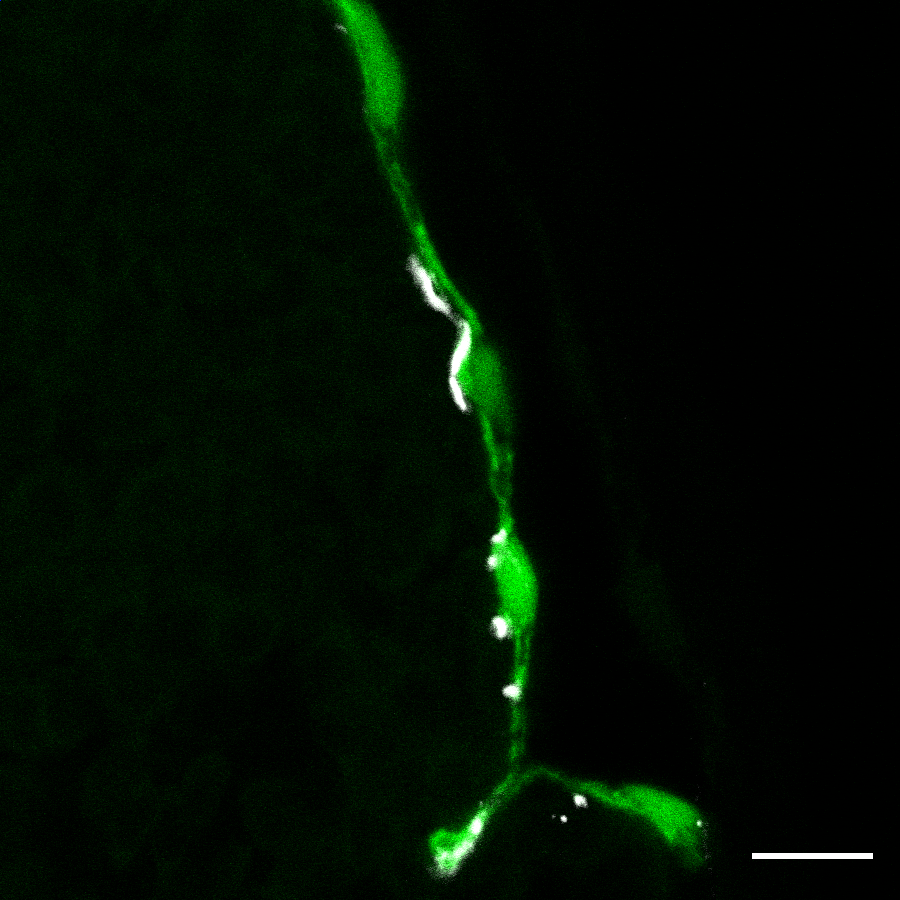

Supplement: Supplementary file 13 — Figure EV4 Source Data [file 44321_2024_152_MOESM13_ESM.zip › Figure EV4/H/MutCtr3zoomgreengrey.png]

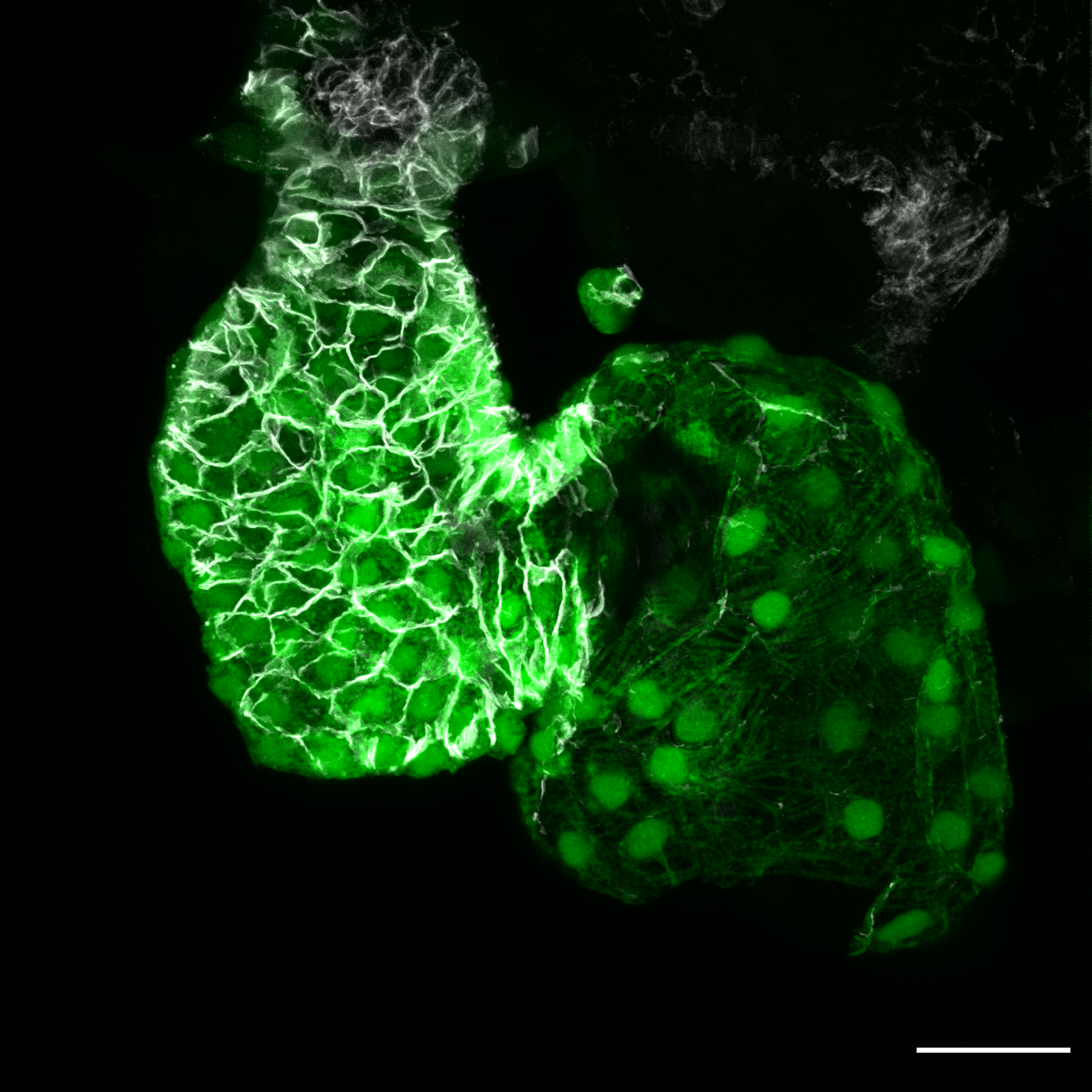

Supplement: Supplementary file 13 — Figure EV4 Source Data [file 44321_2024_152_MOESM13_ESM.zip › Figure EV4/I/MAX_MutInj3.png]

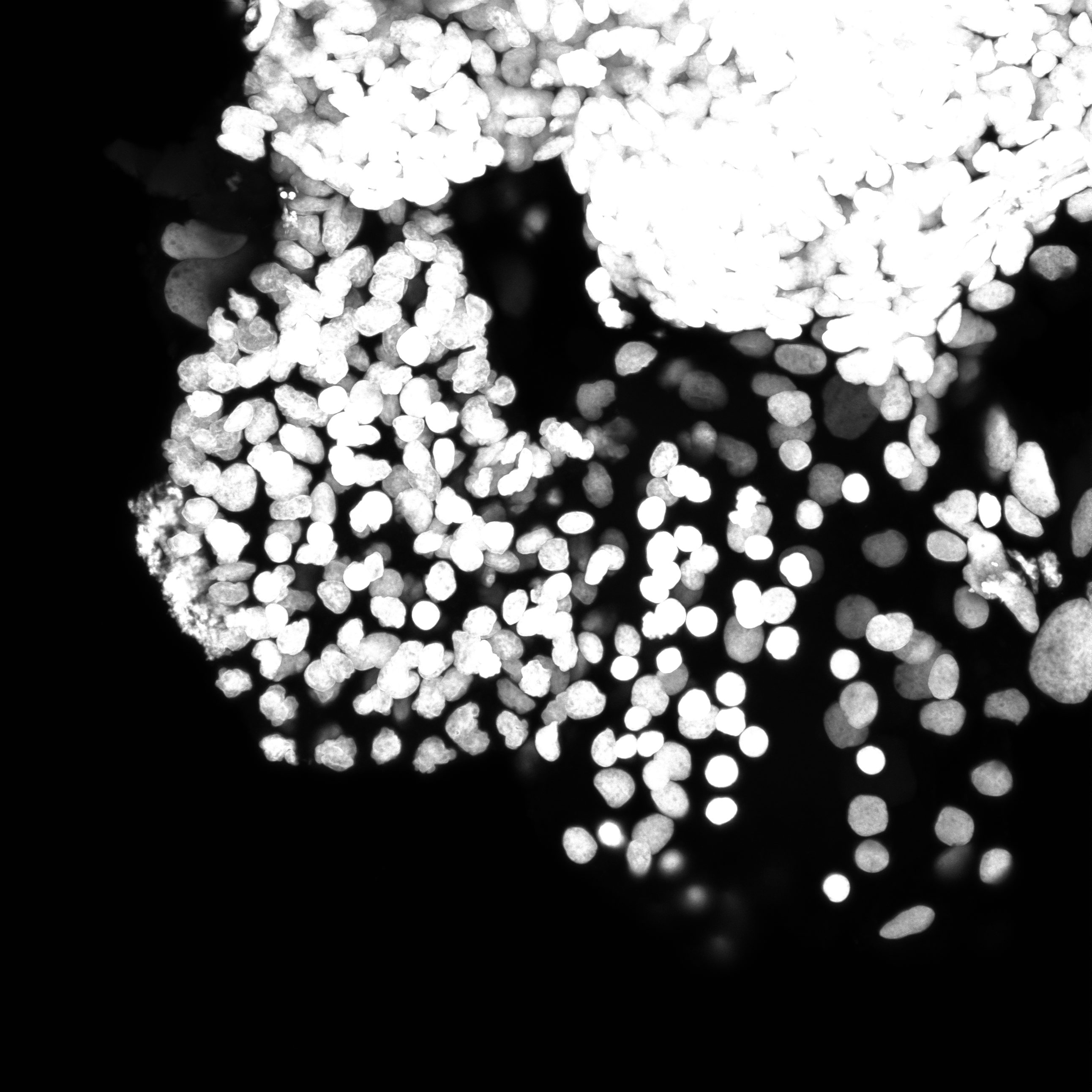

Supplement: Supplementary file 13 — Figure EV4 Source Data [file 44321_2024_152_MOESM13_ESM.zip › Figure EV4/I/MAX_MutInj3.tif]

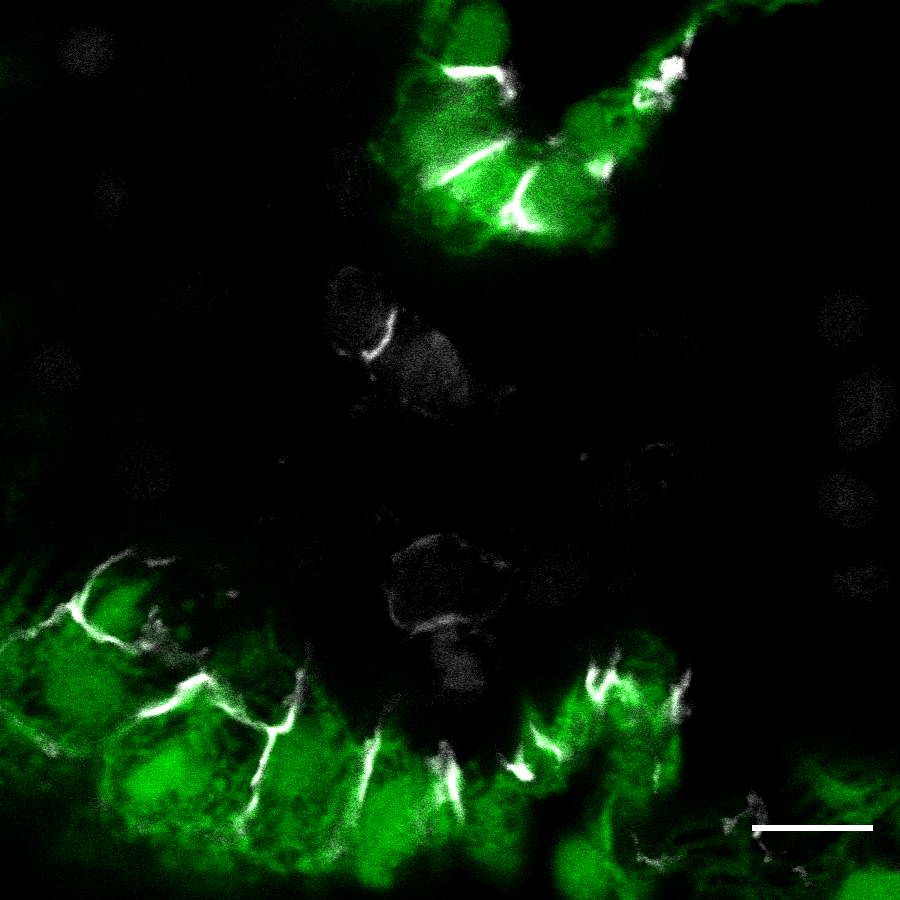

Supplement: Supplementary file 13 — Figure EV4 Source Data [file 44321_2024_152_MOESM13_ESM.zip › Figure EV4/I/MutInj3zoomgreengrey.png]

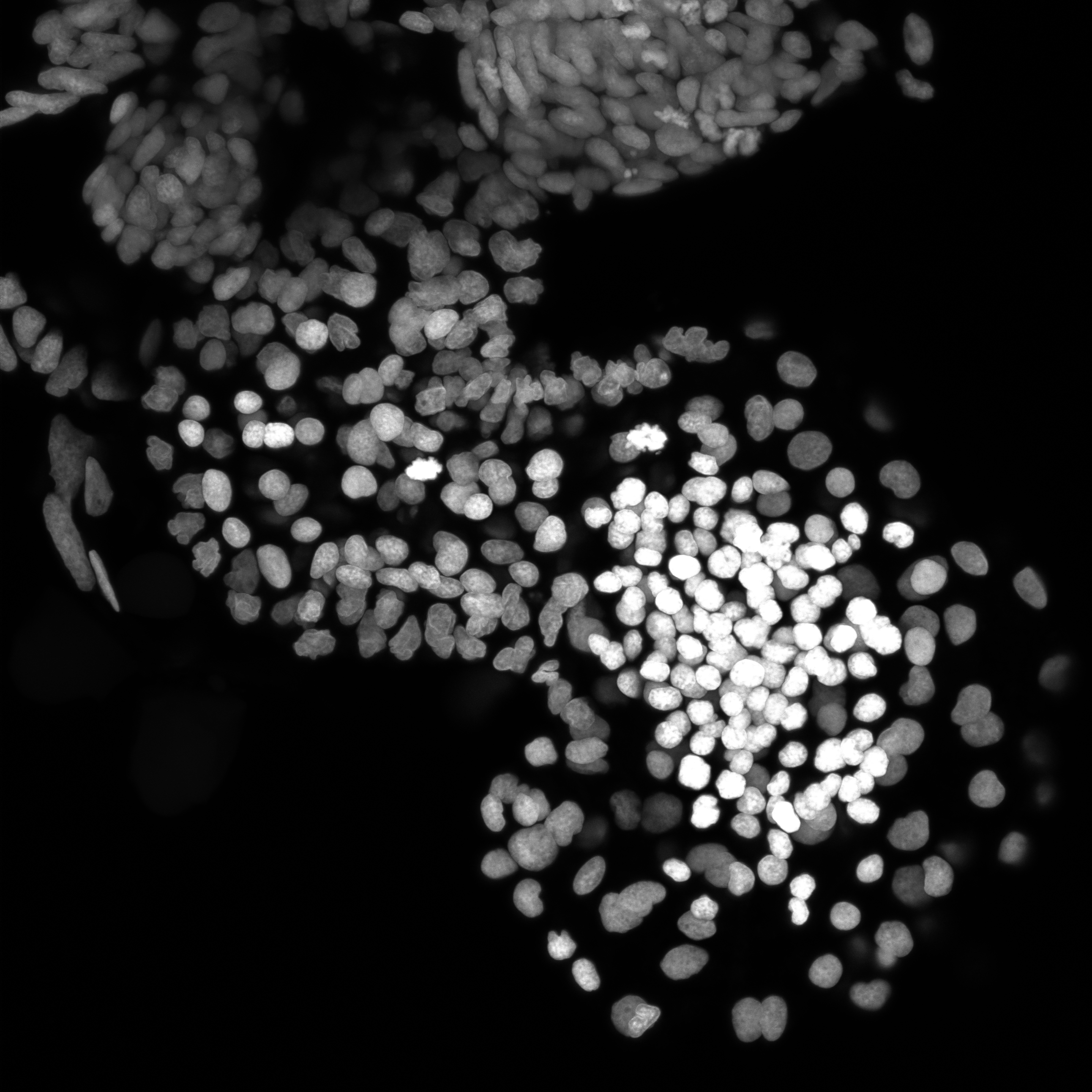

Supplement: Supplementary file 13 — Figure EV4 Source Data [file 44321_2024_152_MOESM13_ESM.zip › Figure EV4/J/MAX_WtInj2.tif]

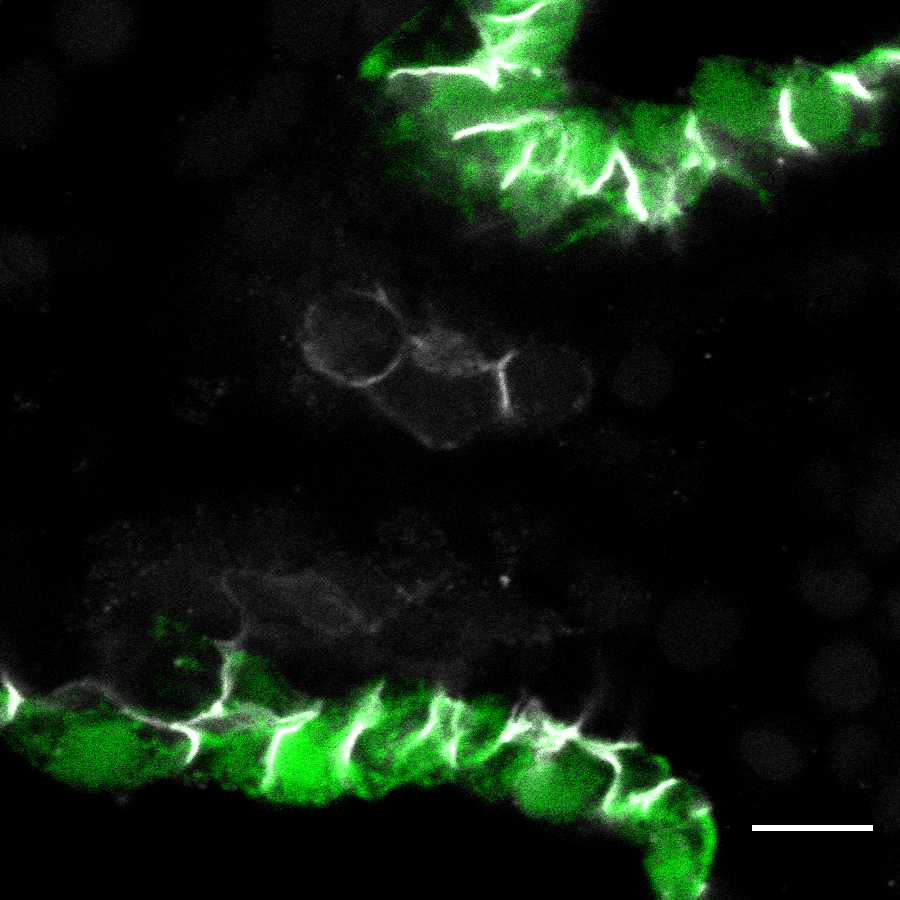

Supplement: Supplementary file 13 — Figure EV4 Source Data [file 44321_2024_152_MOESM13_ESM.zip › Figure EV4/J/WtInj2zoomgreengrey.png]

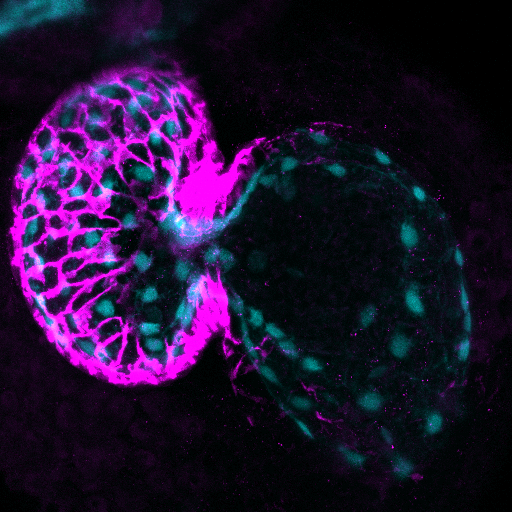

Supplement: Supplementary file 13 — Figure EV4 Source Data [file 44321_2024_152_MOESM13_ESM.zip › Figure EV4/K/MAX_01_ccm2sib_kdrlEGFP_Alcam.tif]

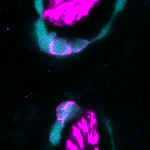

Supplement: Supplementary file 13 — Figure EV4 Source Data [file 44321_2024_152_MOESM13_ESM.zip › Figure EV4/K/MAX_01_ccm2sib_kdrlEGFP_Alcamz10-zoom.tif]

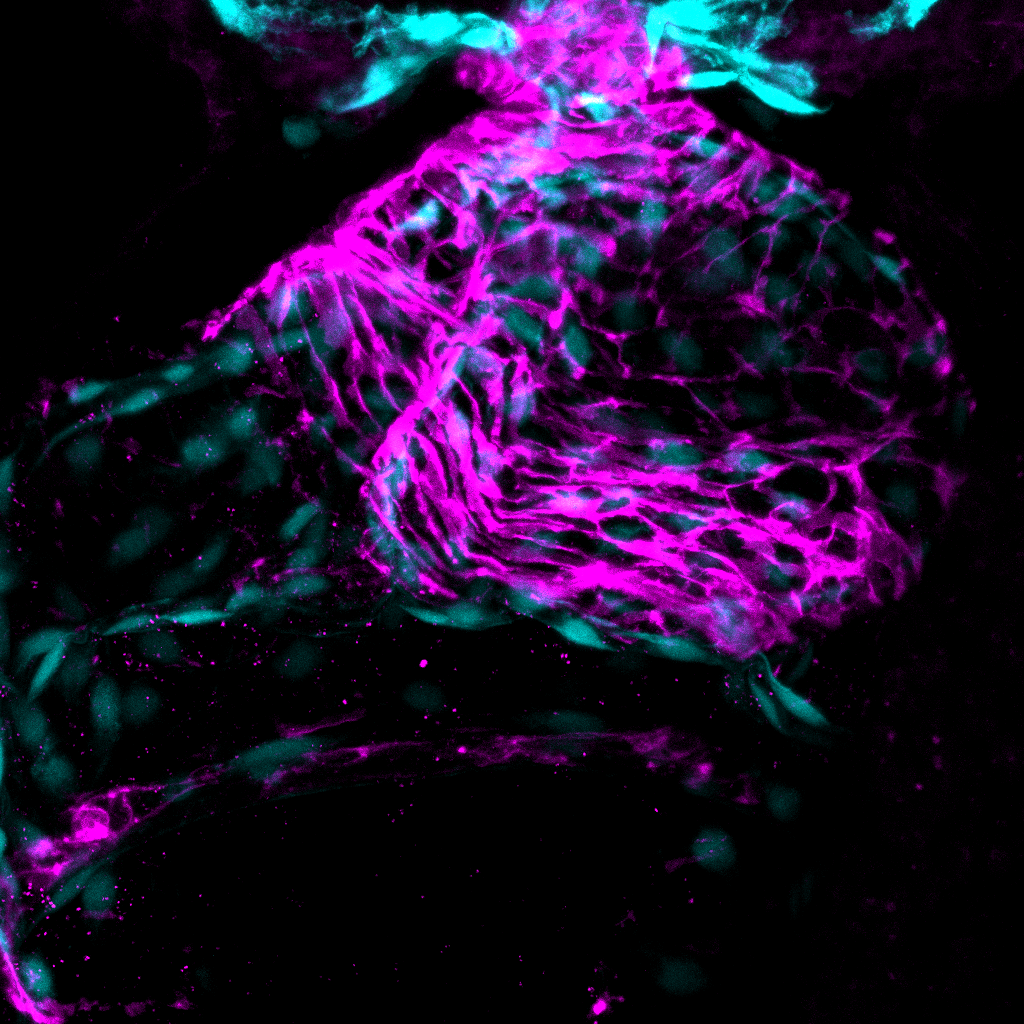

Supplement: Supplementary file 13 — Figure EV4 Source Data [file 44321_2024_152_MOESM13_ESM.zip › Figure EV4/L/MAX_13_ccm2mut_kdrlEGFP_Alcam-1.tif]

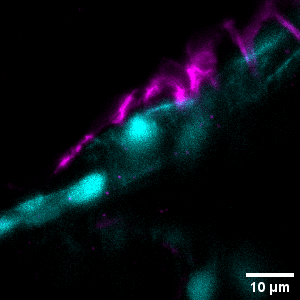

Supplement: Supplementary file 13 — Figure EV4 Source Data [file 44321_2024_152_MOESM13_ESM.zip › Figure EV4/L/MAX_13_ccm2mut_kdrlEGFP_Alcam_z73-75-zoom-sb10um.tif]

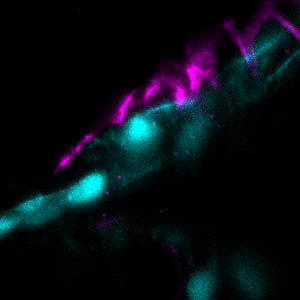

Supplement: Supplementary file 13 — Figure EV4 Source Data [file 44321_2024_152_MOESM13_ESM.zip › Figure EV4/L/MAX_13_ccm2mut_kdrlEGFP_Alcam_z73-75-zoom.tif]

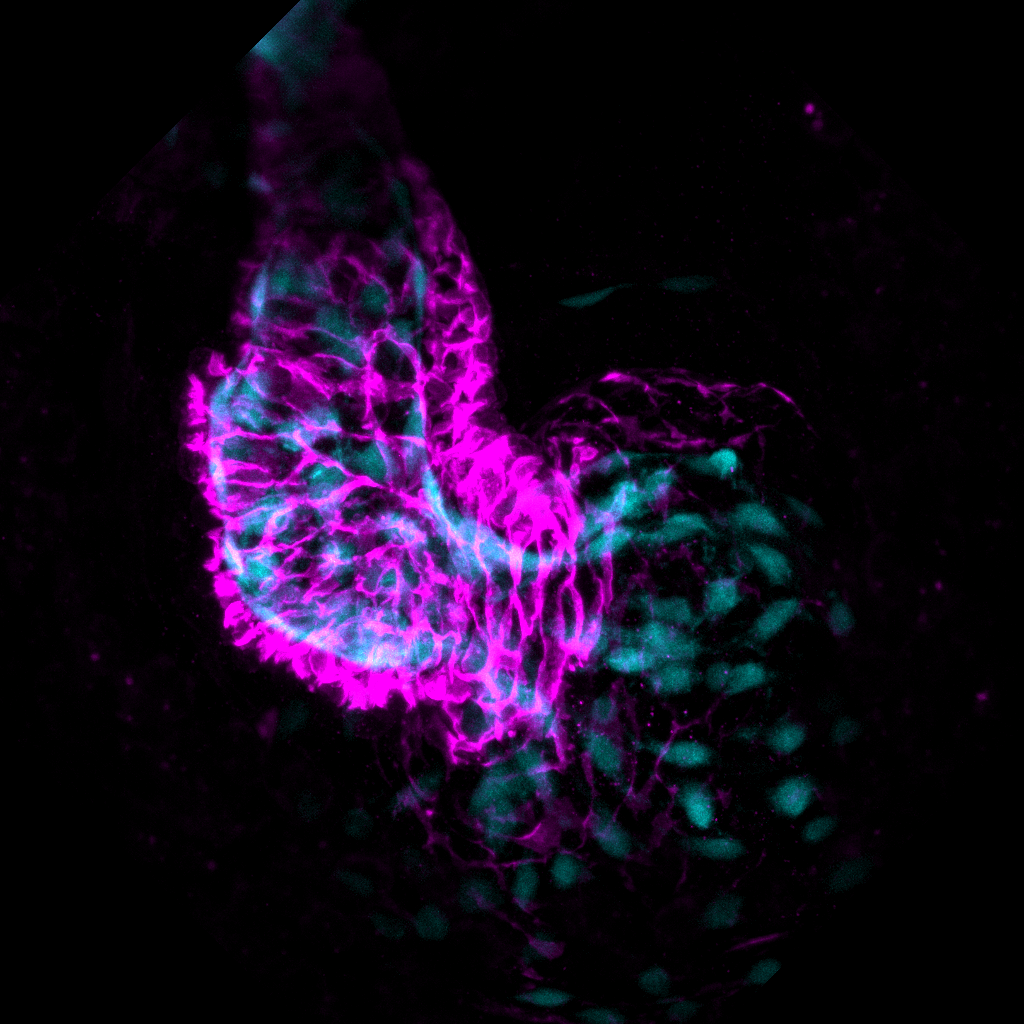

Supplement: Supplementary file 13 — Figure EV4 Source Data [file 44321_2024_152_MOESM13_ESM.zip › Figure EV4/M/MAX_18_ccm2mut_resc_kdrlEGFP_Alcam-1.tif]

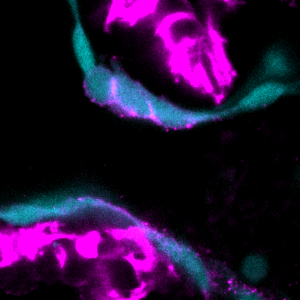

Supplement: Supplementary file 13 — Figure EV4 Source Data [file 44321_2024_152_MOESM13_ESM.zip › Figure EV4/M/MAX_18_ccm2mut_resc_kdrlEGFP_Alcam-z19-20-zoom.tif]

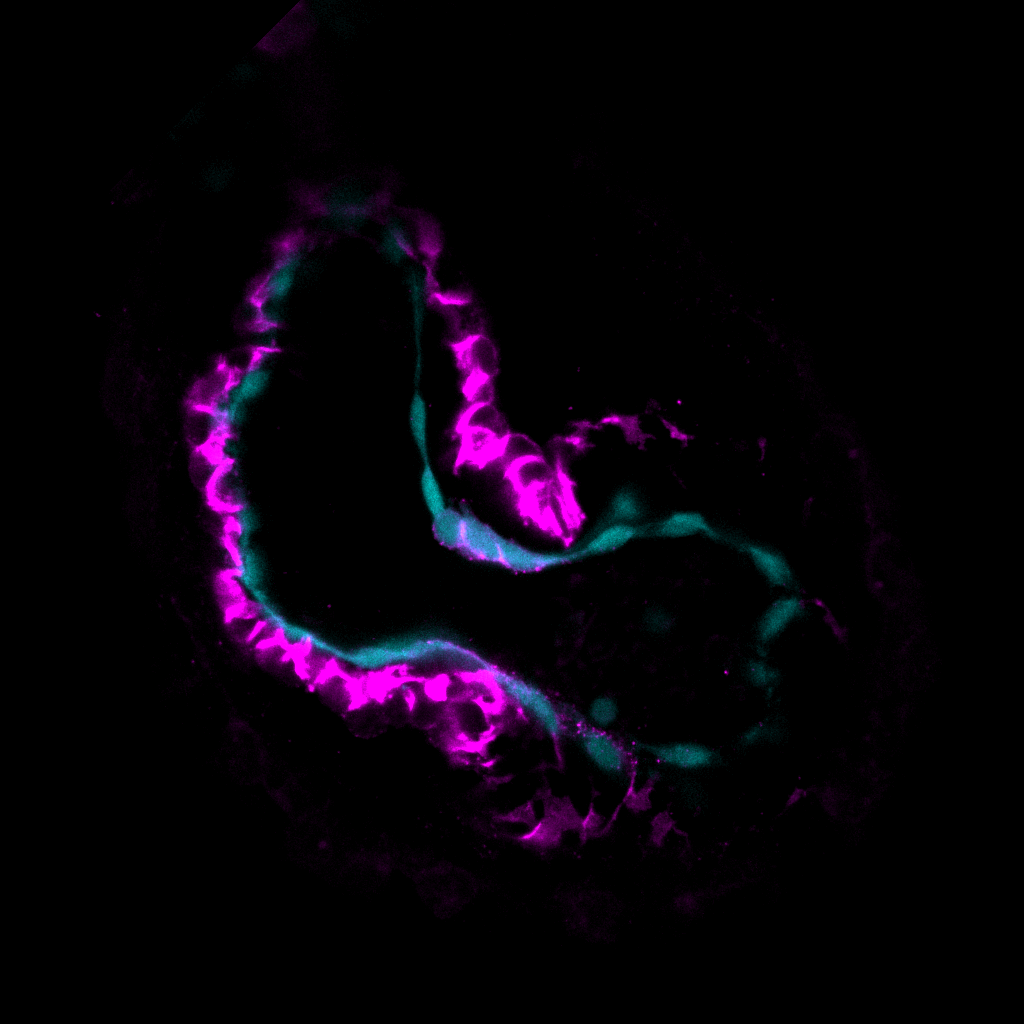

Supplement: Supplementary file 13 — Figure EV4 Source Data [file 44321_2024_152_MOESM13_ESM.zip › Figure EV4/M/MAX_18_ccm2mut_resc_kdrlEGFP_Alcam-z19-20.tif]

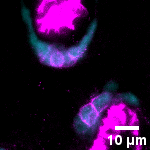

Supplement: Supplementary file 13 — Figure EV4 Source Data [file 44321_2024_152_MOESM13_ESM.zip › Figure EV4/N/MAX_05_ccm2sib_wnt9bmut_kdrlEGFP_Alcam-1-zoom-sb10.tif]

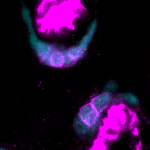

Supplement: Supplementary file 13 — Figure EV4 Source Data [file 44321_2024_152_MOESM13_ESM.zip › Figure EV4/N/MAX_05_ccm2sib_wnt9bmut_kdrlEGFP_Alcam-1-zoom.tif]

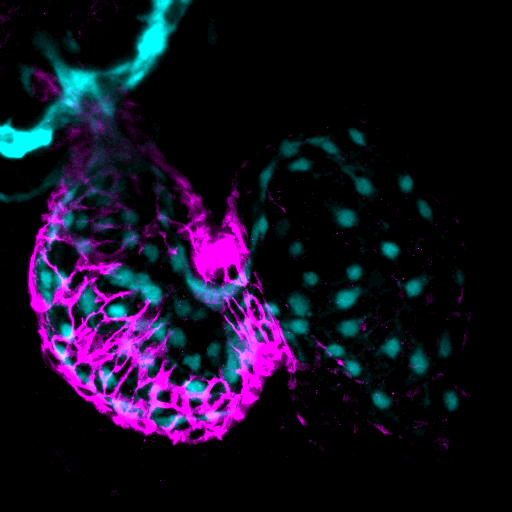

Supplement: Supplementary file 13 — Figure EV4 Source Data [file 44321_2024_152_MOESM13_ESM.zip › Figure EV4/N/MAX_05_ccm2sib_wnt9bmut_kdrlEGFP_Alcam.tif]
