# Supplementary material for: Epigenetic regulation by polycomb repressive complex 1 promotes cerebral cavernous malformations
Source: EMBO Mol Med. 2024 Oct 14;16(11):10. doi: 10.1038/s44321-024-00152-9 (PMC11555420; doi:10.1038/s44321-024-00152-9)
Supplement: Supplementary file 15 — Expanded View Figures [file 44321_2024_152_MOESM15_ESM.pdf]

Expanded View Figures

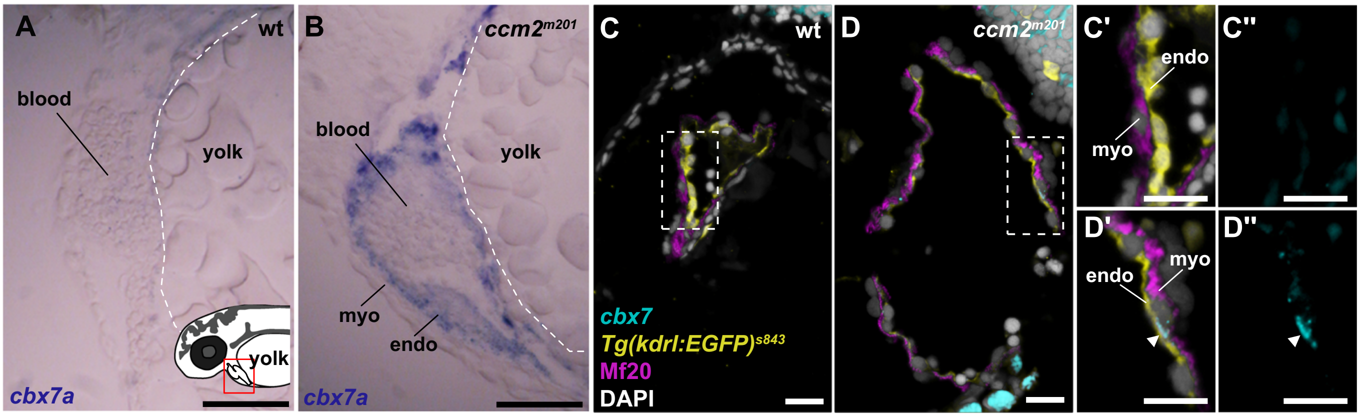

Figure EV1. ***cbx7a*** **is upregulated in the zebrafish endocardium.**

(**A, B**) Shown are sagittal sections of whole-mount in situ hybridizations of zebrafish embryos at 56 hpf in wild-type (wt) (**A**) and *ccm2*^{m201} mutants (**B**), revealing elevated expression levels of *cbx7a* mRNA in *ccm2*^{m201} mutants throughout the entire endocardium. (**C**, **D**) Shown are confocal optical sections of whole-mount fluorescent in situ hybridization for *cbx7a* transcripts and immunohistological co-staining for myocardial marker Mf20. The endocardium is marked by *Tg(kdrl:EGFP)*^{s843} and nuclei are marked by DAPI. Transcripts of *cbx7a* are detected only in the endocardium of *ccm2*^{m201} mutants (**D**, **D′**,**D″**, arrowheads indicate the location of *cbx7a* transcripts), whereas wild-type embryos lack signals for *cbx7a* (**C**, **C′**, **C″**). Scale bars are (**A**, **B**) 100 µm; (**C**, **D**) 20 µm; (**C′**, **D″**) 10 µm. Source data are available online for this figure.

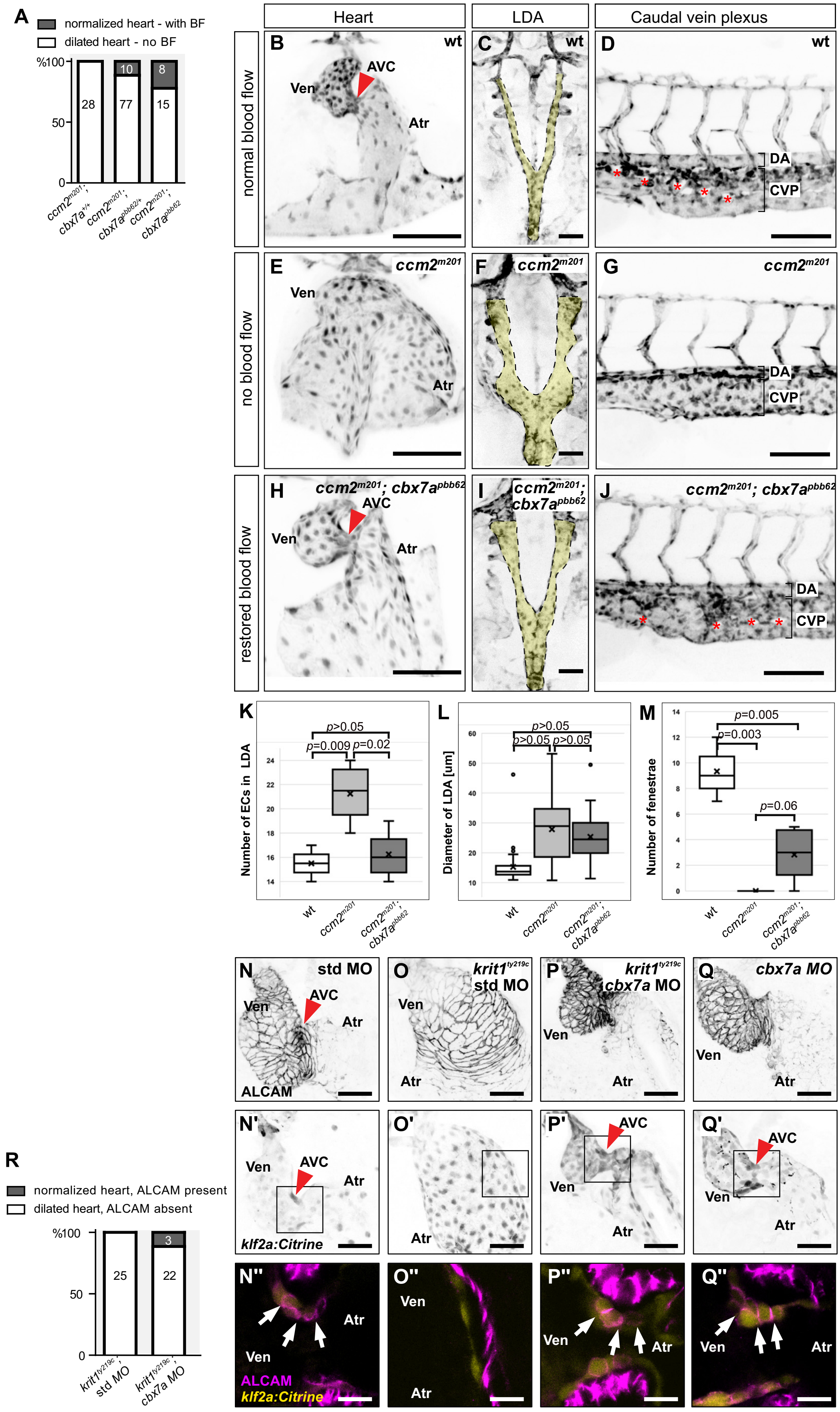
A
normalized heart - with BF
dilated heart - no BF
Heart
LDA
Caudal vein plexus
normal blood flow
no blood flow
restored blood flow
AVC
Ven
Atr
DA
CVP
wt
ccm2m201
ccm2m201; cbx7apbb62
R
normalized heart, ALCAM present
dilated heart, ALCAM absent
std MO
krit1ty219c std MO
krit1ty219c cbx7a MO
cbx7a MO
ALCAM
klf2a:Citrine
Number of ECs in LDA
Diameter of LDA [um]
Number of fenestrae

◀ **Figure EV2. CCM mutant cardiovascular defects are suppressed in zebrafisch embryos upon depletion of Cbx7a.**

(**A**) Shown are quantifications of the share of *ccm2*^{m201} mutant embryos in which cardiovascular phenotypes are normalized, which is never seen in *ccm2*^{m201} mutants. Normalization was assessed by the presence of a constricted atrioventricular canal (AVC) and blood flow (BF). (**B–J**) Shown are maximum projections of confocal microscopic z-stacks with comparisons of cardiovascular phenotypes in wild-type (wt), *ccm2*^{m201} mutants, and *ccm2*^{m201};*cbx7a*^{pbb62} double mutants. The morphology of the heart is normalized upon the loss of *cbx7a* in *ccm2*^{m201} mutants (**B**, **E**, **H**). The lateral dorsal aorta is dilated in *ccm2*^{m201} mutants, and *ccm2*^{m201};*cbx7a*^{pbb62} double mutants (**C**, **F**, **I**; highlighted in yellow). The caudal vein plexus (CVP) is dilated and fused into a single tube in *ccm2*^{m201} mutants. Asterisks indicate spaces (fenestrae) within the caudal vein plexus, which do not form in *ccm2*^{m201} mutants but are present in wild-type and *ccm2*^{m201};*cbx7a*^{pbb62} double mutants (**D**, **G**, **J**). (**K–M**) Quantifications of LDA endothelial cell numbers ($n = 4$ for each condition) and diameter ($n = 4$ for each condition), and number of fenestrae in caudal vein plexus (wild-type, $n = 3$; *ccm2*^{m201}, $n = 3$; *ccm2*^{m201};*cbx7a*^{pbb62} double mutants, $n = 6$) Standard boxplots show median and quartiles with minima and maxima indicated by whiskers (statistical testing is based on pairwise student's *T*-test). The x represents the mean value. Endothelial cell numbers in the lateral dorsal aorta) are normalized (**K**), albeit vessel diameter remains mostly unchanged (**L**). The caudal vein plexus (CVP) is normalized upon loss of *cbx7a* in *ccm2*^{m201} mutants (**M**). (**N–Q**) Functional rescue of endocardial phenotypes through genetic depletion of Cbx7a in zebrafish *krit1*^{ty219c} mutant embryos. Shown are maximum projections of confocal image z-stacks of zebrafish hearts at 56 hpf with myocardium marked with ALCAM (**N–Q**) and the endocardium being marked by *Tg(klf2a:Citrine)*^{mu107} expression (**N′–Q′**). The expression of ALCAM in endocardial AVC cells (**N″**; arrows) is lost in *krit1*^{ty219c} mutants (**O″**). The depletion of *cbx7a* using an antisense oligo morpholino (*cbx7a* MO) rescues cardiac morphology in *krit1*^{ty219c} mutant hearts. In 3 of 25 embryos, the AVC region is restored (**P′**, red arrowhead), and ALCAM expression at the endocardial AVC is restored (**P″**; arrows; **R**). Injection of a control morpholino does not have any effects on overall morphology or ALCAM expression (**N**, **N′**, **N″**). Knockdown of *cbx7a* alone does not affect AVC formation and ALCAM expression (**Q**, **Q′**, **Q″**). AVC atrioventricular canal, Ven ventricle, Atr atrium. Statistical testing is based on unpaired student's *T*-test between (s.e.m. bars indicated). Red arrowheads indicate AVC while white arrows indicate the presence of ALCAM expression in AVC endocardial cells. AVC atrioventricular canal, CVP caudal vein plexus, Ven ventricle, Atr atrium. Scale bars are (**B**, **D**, **E**, **G**, **H**, **J**) 100 µm; (**N–Q**, **N′–Q′**) 50 µm; (**C**, **F**, **I**, **N″–Q″**) 10 µm. Source data are available online for this figure.

© The Author(s)

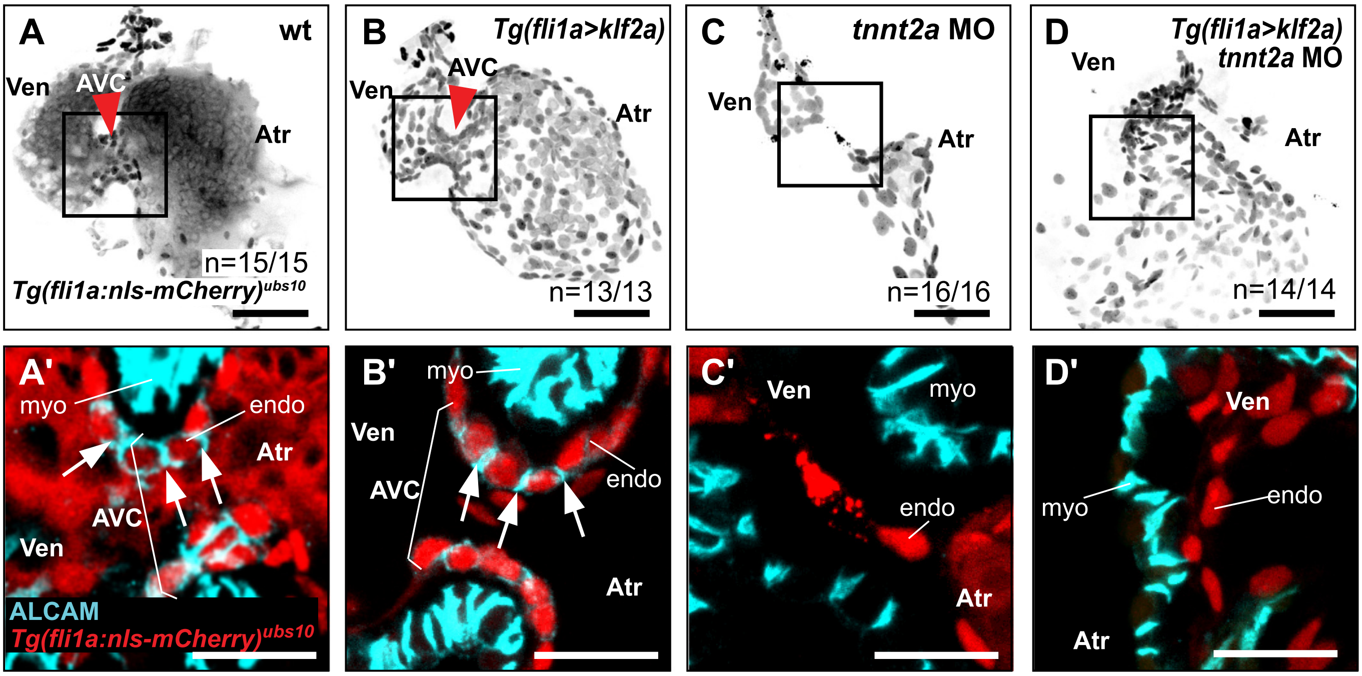

Figure EV3. Concomitant loss of blood flow and endothelial overexpression Klf2a causes CCM-like cardiac phenotypes in zebrafish embryos.

(**A–D**) Shown are confocal z-scan projections of zebrafish embryonic endocardium marked by expression of *Tg(fli1a:nls-mCherry)*^{ubs10} at 48 hpf of wild-type (**A**, **A**′), *Tg(fli1a:Gal4FF)*^{ubs3}; *Tg(UAS:klf2a)*^{ig1} double transgenic embryos [*Tg(fli1a>klf2a)*]. (**B**, **B**′) An antisense morpholino oligo against *tnnt2a* injected into wild-type (**C**, **C**′) and *Tg(fli1a>klf2a)* (**D**, **D**′). Inserts (**A**′–**D**′) provide magnifications based on projections of fewer z-scan section planes of the atrioventricular canal (AVC) region. In comparison to wild-type (**A**, **A**′), *Tg(fli1a>klf2a)* hearts show a ballooning of the atrium (**B**), while the overall morphology of the AVC appears normal, with ALCAM expression intact at the endocardial cells of the AVC (**B**, red arrow; **B**′, arrowheads). The MO-mediated depletion of *tnnt2a* in wild-type leads to a severe reduction in cardiac size (**C**), and endocardial cells of the AVC fail to express ALCAM (**C**′). Knock-down of *tnnt2a* in *Tg(fli1a>klf2a)* augments the cardiac ballooning, leading to loss of the AVC constriction (**D**) and loss of expression of ALCAM in endocardial AVC cells (**D**′). Numbers (*n*) indicate observed phenotypes in different conditions. Red arrowheads indicate AVC while white arrows indicate the presence of ALCAM expression in AVC endocardial cells. AVC atrioventricular canal, endo endocardium, myo myocardium, OFT outflow tract, Ven ventricle, Atr atrium. Scale bars are (**A–D**) 50 μm, (**A**′–**D**′) 20 μm. Source data are available online for this figure.

 © The Author(s)

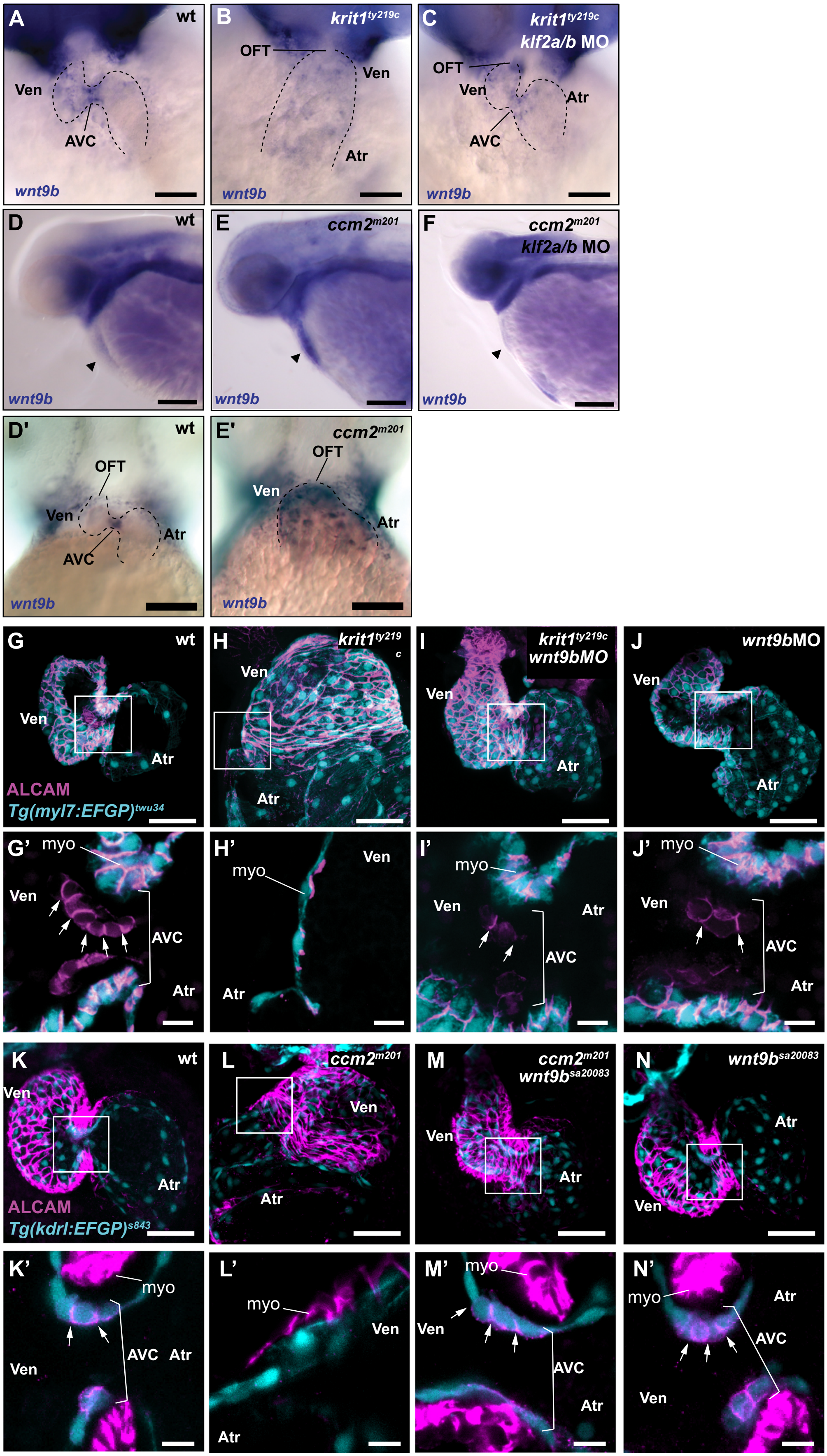

A wt
Ven
AVC
wnt9b
B krit1ty219c
OFT
Ven
Atr
wnt9b
C krit1ty219c klf2a/b MO
OFT
Ven
Atr
AVC
wnt9b
D wt
wnt9b
E ccm2m201
wnt9b
F ccm2m201 klf2a/b MO
wnt9b
D' wt
OFT
Ven
Atr
AVC
wnt9b
E' ccm2m201
OFT
Ven
Atr
wnt9b
G wt
Ven
Atr
ALCAM
Tg(myl7:EFGP)twu34
H krit1ty219c
Ven
Atr
I krit1ty219c wnt9bMO
Ven
Atr
J wnt9bMO
Ven
Atr
G' myo
Ven
AVC
Atr
H' myo
Ven
Atr
I' myo
Ven
Atr
AVC
J' myo
Ven
AVC
Atr
K wt
Ven
Atr
ALCAM
Tg(kdrl:EFGP)s843
L ccm2m201
Ven
Atr
M ccm2m201 wnt9bsa20083
Ven
Atr
N wnt9bsa20083
Atr
Ven
K' myo
Ven
AVC
Atr
L' myo
Ven
Atr
M' myo
Ven
AVC
Atr
N' myo
Atr
AVC
Ven

Figure EV4. The upregulation of *wnt9b* expression in *ccm* zebrafish mutants involves Klf2 and contributes to CCM phenotypes.

(**A–F**) Representative images of whole-mount in situ hybridizations against *wnt9b* mRNA in the zebrafish heart (outlined by dotted lines) at 48 hpf (**D–F**, lateral views). *wnt9b* mRNA expression is restricted to the AVC region in wild-type embryos (A,D,D′) and elevated throughout the entire heart in $ccm2^{m201}$ and $krit1^{ty219c}$ mutants (**B**, **E**, **E′**). (**C**, **F**) Upon knock-down of *klf2a* and *klf2b* in $ccm2^{m201}$ and $krit1^{ty219c}$ mutants, expression of *wnt9b* is restricted to the AVC region. (**G–N**) Shown are confocal z-scan projections of zebrafish embryonic hearts at 48 hpf with inserts (**G′–N′**) providing magnifications based on projections of fewer z-scan section planes of the atrioventricular canal (AVC) region. The myocardium is marked by $Tg(myl7:EGFP)^{twu34}$ expression and ALCAM (**G–J**, **G′–J′**) or only ALCAM (**K–N**; **K′–N′**). In comparison to the heart morphology in wild-type (**G**, **G′**, arrows indicate ALCAM expression in endocardial cells of the AVC $n = 10/10$ hearts), $krit1^{ty219c}$ mutant hearts exhibit a ballooning morphology (**H**, $n = 4/4$ hearts) and lack ALCAM expression in endocardial cells of the AVC (**H′**). The morpholino-mediated depletion of *wnt9b* in $krit1^{ty219c}$ mutants normalizes the heart morphology with a constriction at the AVC (**I**) and ALCAM expression in AVC endocardial cells (**I′**; arrows). The morpholino-mediated knock-down of *wnt9b* in wild-type does not affect AVC formation and endocardial ALCAM expression (**J**, **J′**, $n = 3/3$ hearts). In $ccm2^{m201}$ mutant hearts, the cardiac ballooning morphology (**L**) and lack of ALCAM expression in endocardial cells of the AVC (**L′**) is restored only in embryos that are also mutant for $wnt9b^{sa20083}$ (**M**, **M′**, $n = 5/14$ $ccm2^{m201}$ mutant hearts) with a normalized heart morphology and a constriction at the AVC. In comparison, $wnt9b^{sa20083}$ mutants do not exhibit defects in cardiac morphology or ALCAM expression (**N**, **N′**, $n = 4/4$ hearts). Black arrowheads indicate the location of the heart. White arrows indicate the presence of ALCAM expression in AVC endocardial cells. AVC atrioventricular canal, myo myocardium, Ven ventricle, Atr atrium. Scale bars are (**A–F**) 100 µm, (**G–J**, **K–N**) 50 µm, (**G′–J′**, **K′–N′**) 10 µm. Source data are available online for this figure.

 © The Author(s)

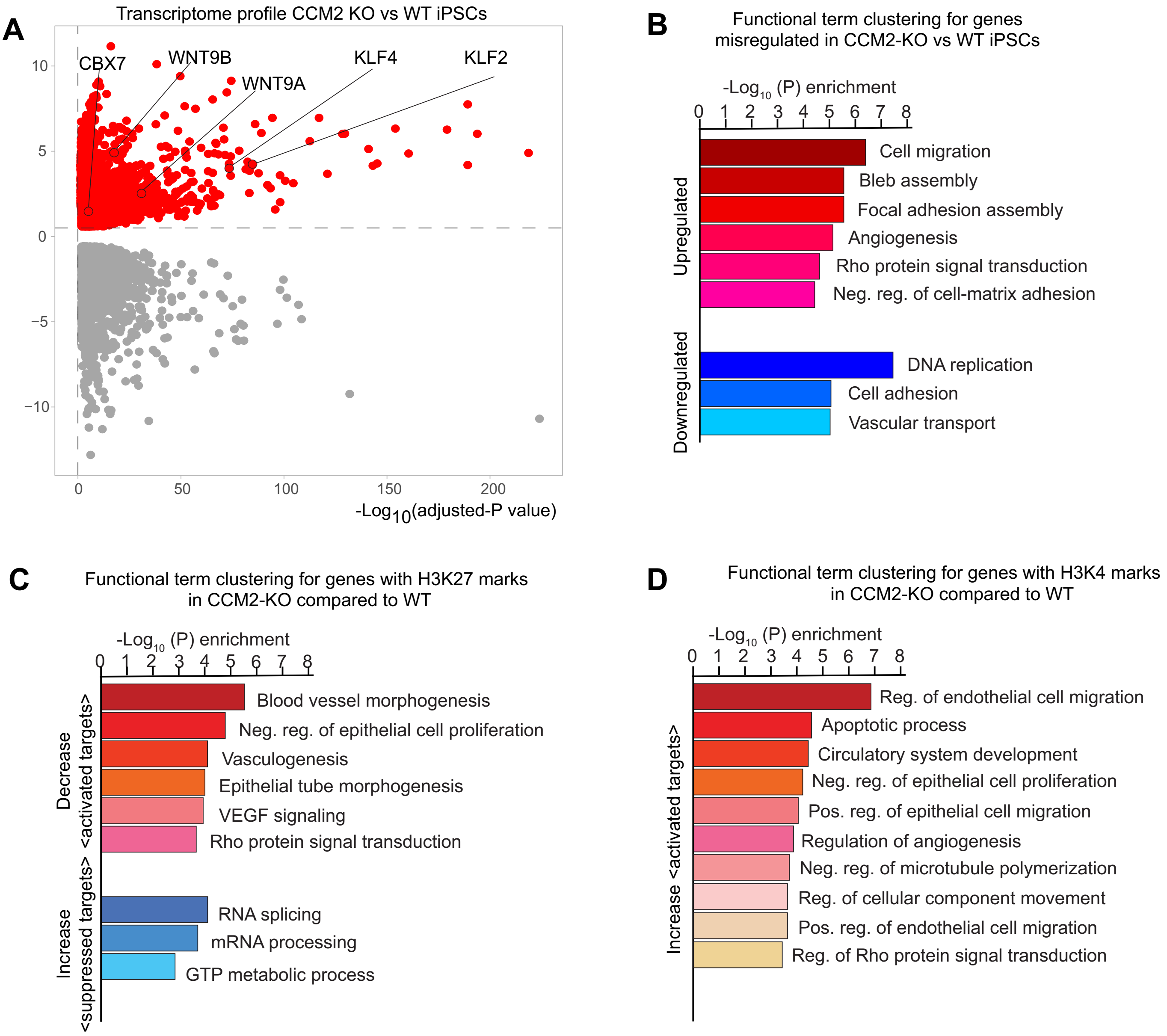

Figure EV5. Transcriptional and epigenetic regulation in *CCM2* knock out (KO) iPSC-derived ECs.

(**A**) Volcano plot of RNA-seq data of differentially-expressed genes (calculated by Wald test under DESeq2 package (v1.26.0), adjusted *p* value <0.05 and fold change >1) in CCM2-depleted iPCS-derived ECs compared to wild-type. Indicated is the upregulation of the CCM hallmark marker genes *KLF2* and *KLF4* as well as *CBX7*, *WNT9A*, and *WNT9B*. (**B**) Functional clustering of gene ontology terms for genes misregulated in *CCM2*-KO as compared to WT. (**C**, **D**) Functional clustering of gene ontology terms for inactivating H3K27me3 mark target genes (both increased and decreased targets) (**C**) and increased activating H3K4me3 mark gene targets (**D**) in CUT&RUN-seq data from *CCM2*-KO and WT (differentially bound peaks were calculated by Wald test under DESeq2 associated with Diffbind (v.3.4.3) package). Source data are available online for this figure.

© The Author(s)
